# B(C_6_F_5_)_3_‐Catalyzed *E*‐Selective Isomerization of Alkenes

**DOI:** 10.1002/chem.202202454

**Published:** 2022-09-01

**Authors:** Betty A. Kustiana, Salma A. Elsherbeni, Thomas G. Linford‐Wood, Rebecca L. Melen, Matthew N. Grayson, Louis C. Morrill

**Affiliations:** ^1^ Cardiff Catalysis Institute School of Chemistry Cardiff University Main Building Park Place Cardiff CF10 3AT UK; ^2^ Department of Chemistry University of Bath Bath BA2 7AY UK

**Keywords:** alkenes, borane catalysis, isomerization

## Abstract

Herein, we report the B(C_6_F_5_)_3_‐catalyzed *E*‐selective isomerization of alkenes. The transition‐metal‐free method is applicable across a diverse array of readily accessible substrates, giving access to a broad range of synthetically useful products containing versatile stereodefined internal alkenes. The reaction mechanism was investigated by using synthetic and computational methods.

## Graphical abstract

**Move along**!: Herein, we report the B(C_6_F_5_)_3_‐catalyzed *E*‐selective isomerization of alkenes. The transition‐metal‐free method is applicable across a diverse array of readily accessible substrates, accessing a broad range of synthetically useful products containing versatile stereodefined internal alkenes. Synthetic and computational mechanistic studies indicate that the isomerization proceeds along competing 1,2‐hydride shift and hydride abstraction pathways.

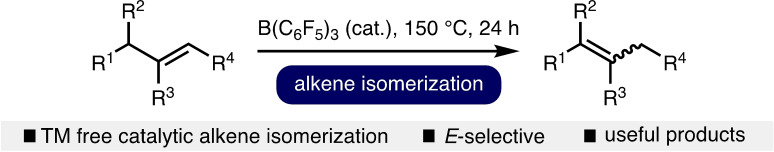

## Introduction

Main‐group chemistry is a topical area of research that has undergone a renaissance in the past 20 years.^[1]^ This can partly be attributed to the unique chemistry and reactivity exhibited by main group compounds, which can offer complementarity to transition metal catalysis.^[2]^ Furthermore, there is an increasing global challenge to develop catalytic methods for the production of chemicals for society that provide alternatives to catalysts based on precious metals. As such, the development of novel methodologies that employ main group catalysts, diversifying their reactivity profile, is an important and timely pursuit. One of the widely explored classes of main group compounds are Lewis acidic boranes, such as the archetypal tris(pentafluorophenyl)borane, B(C_6_F_5_)_3_.^[3]^ These versatile species participate in a diverse range of processes including borylation, hydrosilylation, Lewis acid catalysis, and frustrated Lewis pair (FLP) chemistry.^[4]^

**Scheme 1:**
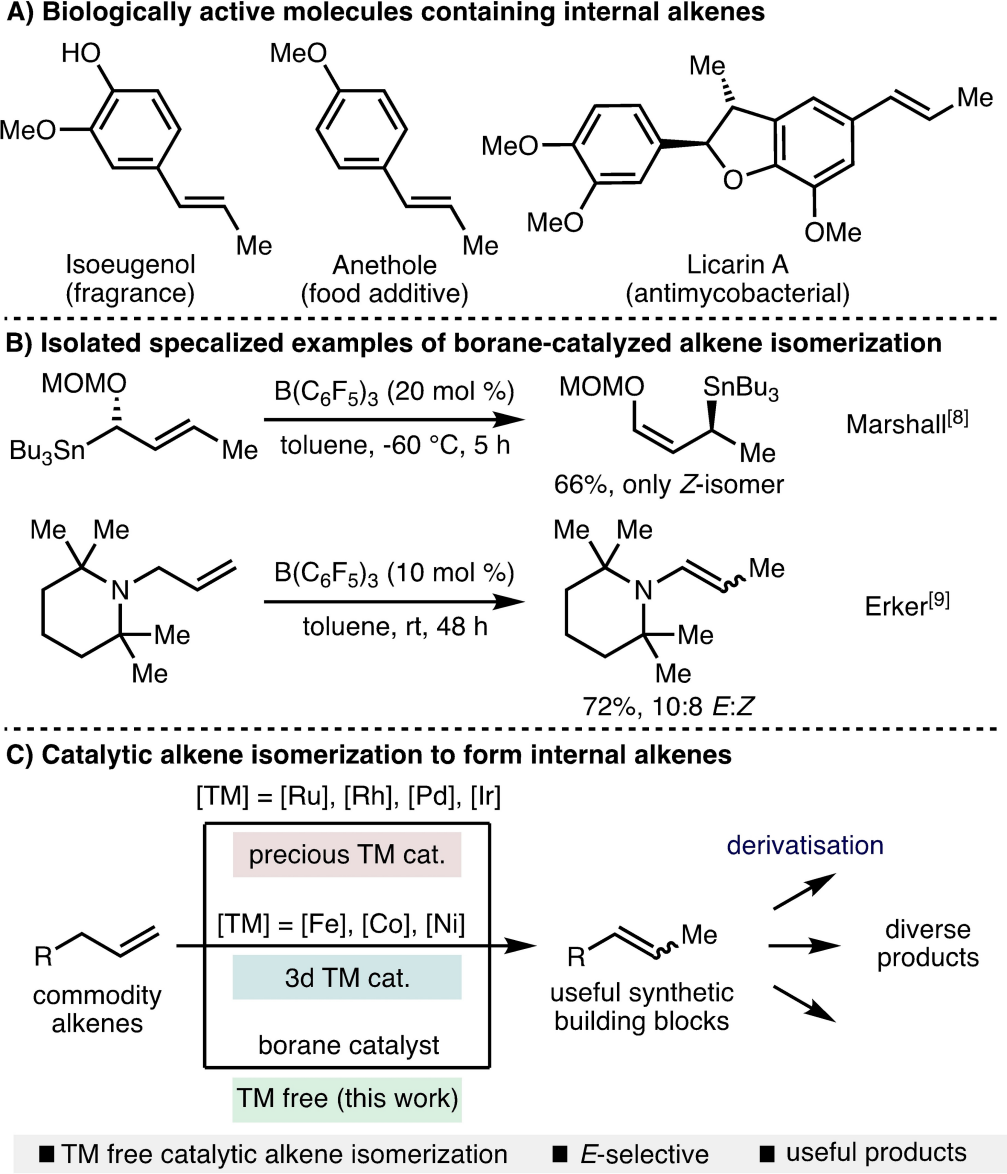
Context and outline of this work. TM=transition metal, MOM=methoxymethyl.

Alkene‐containing compounds are ubiquitous throughout chemistry. Isoeugenol (fragrance), Anethole (food additive), and Licarin A (antimycobacterial) are examples of biologically active molecules that contain internal alkenes (Scheme 1A). An attractive approach for the formation of internal alkenes is through the isomerization of terminal alkenes, due to their relative ease of synthesis and greater commercial availability.^[5]^ Catalytic approaches to alkene isomerization have been developed that employ a broad range of catalysts based on precious transition metals (e. g., Ru, Rh, Pd, Ir),^[6]^ and more recently Earth‐abundant first‐row transition metals (e. g., Fe, Co, Ni).^[7]^ With respect to borane catalysis, there exists only sporadic reports of alkene isomerization within specialized systems. Marshall and Gill reported the B(C_6_F_5_)_3_‐catalyzed isomerization of α‐ to γ‐oxygenated allylic stannanes (Scheme 1B),^[8]^ whereas Erker and co‐workers found that *N*‐allyl tetramethylpiperidine isomerized to the corresponding enamine in the presence of catalytic quantities of B(C_6_F_5_)_3_.^[9]^ As part of our ongoing interest in expanding the utility of borane catalysts in synthesis,^[10]^ we envisaged the development of a general borane‐catalyzed protocol for the *E*‐selective isomerization of alkene‐containing compounds. Herein, we report the successful realization of this approach, which permits access to a broad range of synthetically useful internal alkene products (Scheme 1C).

## Results and Discussion

To commence our studies, the isomerization of allylbenzene **1** to form prop‐1‐en‐1‐ylbenzene (**2**) was selected for reaction optimization (Table 1).^[11]^ Employing B(C_6_F_5_)_3_ (10 mol %) as catalyst and toluene as solvent ([**1**]=0.5 M) in a sealed tube at 150 °C for 24 h under Ar, gave **2** in 97 % NMR spectroscopic yield with a 94 : 6 *E : Z* isomeric ratio (entry 1). No conversion occurs in the absence of B(C_6_F_5_)_3_ (entry 2). It was found that employing xylenes as the reaction solvent gave **2** in a comparable 94 % NMR spectroscopic yield (entry 3), however, employing anisole, chlorobenzene or bromobenzene as solvent all resulted in lower conversion to **2** (entries 4–6). Lowering the catalyst loading, concentration, temperature, and reaction time had a detrimental impact upon product formation (entries 7–10).

**Table 1:** Optimization of the isomerization process.^[a]^

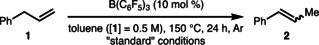			
	Variation from “standard” conditions	Yield^[b]^ [%]	E : Z ratio^[b]^
**1**	**none**	**97**	**94 : 6**
2	no B(C_6_F_5_)_3_	<2	n.d.
3	xylenes as solvent	94	94 : 6
4	anisole as solvent	60	97 : 3
5	chlorobenzene as solvent	56	95 : 5
6	bromobenzene as solvent	70	90 : 10
7	B(C_6_F_5_)_3_ (5 mol %)	54	>98:<2
8^[c]^	[**1**]=0.25 M	34	97 : 3
9	140 °C	10	90 : 10
10	reaction time=16 h	47	98 : 2

[a] Reactions performed using 0.2 mmol of **1**. [b] As determined by ^1^H NMR analysis of the crude reaction mixture with 1,3,5‐trimethylbenzene as the internal standard. [c] 0.1 mmol of **1**. n.d.: not determined.

**Scheme 2:**
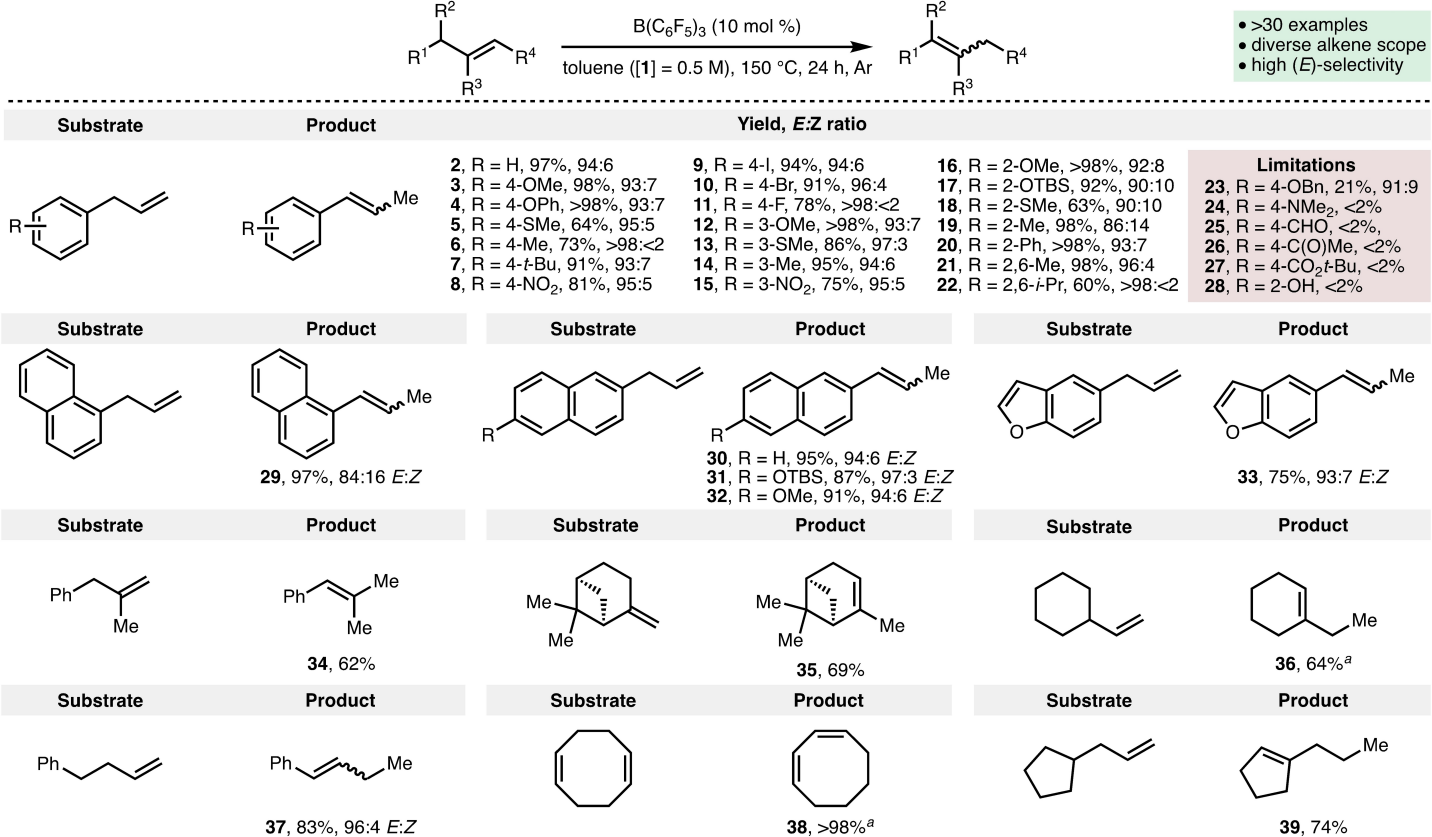
Substrate scope. Reactions performed by using 0.2 mmol of alkene with yields and *E : Z* ratios determined by ^1^H NMR analysis of the crude reaction mixture with 1,3,5‐trimethybenzene as the internal standard. [a] Reaction time=48 h.

With the optimized reaction conditions in hand, the full scope of the B(C_6_F_5_)‐catalyzed alkene isomerization process was explored (Scheme 2). It was found that a variety of electron‐releasing (e. g., OMe, SMe, *t*Bu), electron‐withdrawing (e. g., NO_2_), and halogen substituents could be present at the *ortho*, *meta*, and *para* positions relative to the allyl functionality within the allylbenzene scaffold; this enabled access to the corresponding substituted styrene derivatives **3–22** in high yields and with a high selectivity for the *E*‐alkene isomer. The isomerization process was found to proceed efficiently employing sterically encumbered allylbenzenes containing *ortho*‐substituted aromatics (e. g., products **16**–**22**), with product **22**, containing bulky isopropyl groups at the 2‐ and 6‐positions, being formed in 60 % yield using optimized reaction conditions. When the aromatic unit was substituted with 4‐OBn and 4‐NMe_2_ groups, 21 % and <2 % conversion to the corresponding styrene derivatives (**23** and **24**) was observed, respectively, which may be attributed towards competing coordination of these basic functionalities to B(C_6_F_5_) and/or competing C−H hydride abstraction processes.^[12]^ It was also found that the presence of aromatic aldehyde, ketone, ester and alcohol functionalities within the allylbenzene substrates resulted in no observable conversion to the corresponding styrene derivatives (**25**–**28**), presumably due to catalyst poisoning by substrate coordination. However, TBS‐protected phenol derivative **17** was formed in 92 % yield with 90 : 10 *E : Z* selectivity. Substrates containing substituted naphthyl and benzofuranyl motifs were converted into products **29**–**33** in high yields. It was found that 1,1‐disubstituted alkenes also undergo efficient isomerization, with product **34** formed in 62 % yield. There is no requirement for an aromatic group within the substrates, with β‐pinene converted to α‐pinene **35** in 69 % yield. Furthermore, alkene isomerization proceeds until the thermodynamic product is formed (e. g., products **36**–**39**).

**Scheme 3:**
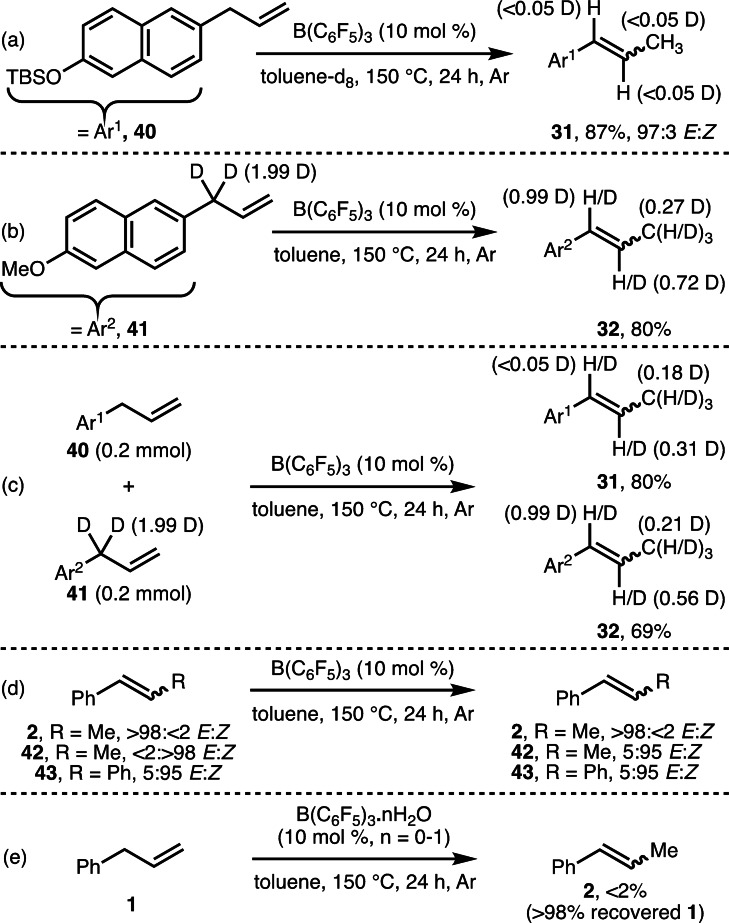
Mechanistic studies. Yields, deuterium incorporation and *E : Z* ratios determined by ^1^H NMR analysis of the crude reaction mixture with 1,3,5‐trimethybenzene as the internal standard.

**Scheme 4:**
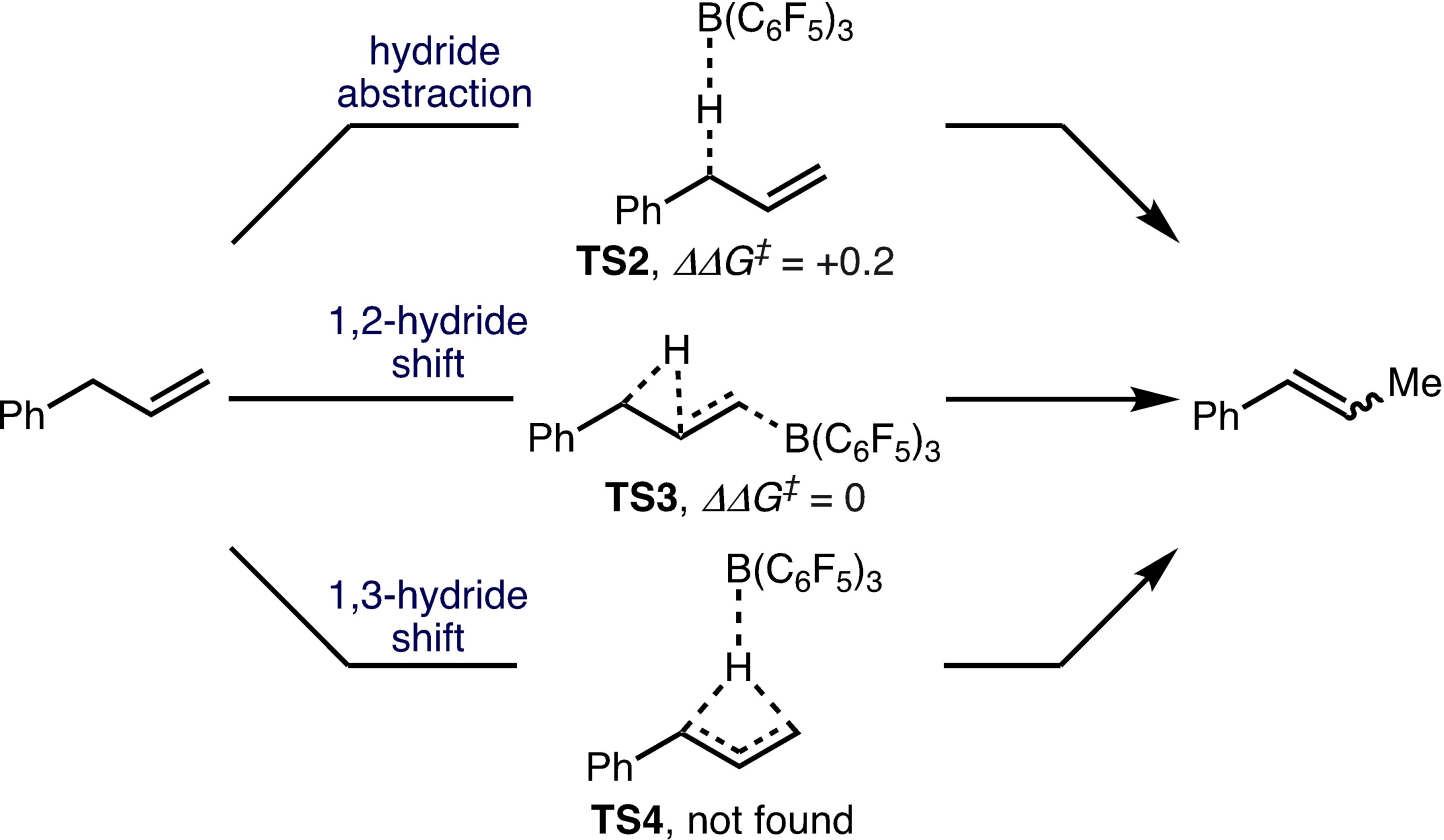
Proposed mechanistic pathways and ΔΔ*G* ^≠^ for borane‐catalyzed hydride abstraction (TS2) and 1,2‐hydride shift (TS3), TSs calculated at the M06‐2X/def2‐TZVPP/IEF‐PCM(Tol)//AM1/IEF‐PCM(Tol) level of theory and given in kcal mol^−1^.

A selection of experiments was performed to gain insight into the reaction mechanism (Scheme 3). Firstly, no deuterium incorporation within product **31** was observed when [D_8_]toluene‐was employed as solvent (Scheme 3a). When deuterated substrate **41** was subjected to the “standard” reaction conditions, deuterium incorporation was observed at all positions within the allyl group of **32** (Scheme 3b), which indicated the possibility of competing 1,2‐ and 1,3‐hydride shift pathways. A cross‐over experiment involving substrates **40** and **41** (Scheme 3c) resulted in deuterium incorporation within product **31**, which confirmed the presence of intermolecular hydride shifts. Resubjecting *E*‐alkene **2** (>98:<2 *E : Z*) to the “standard” reaction conditions resulted in no observable reaction (Scheme 3d), whereas the corresponding *Z*‐alkene (<2:>98 *E : Z*) was isomerized to a small degree (5 : 95 *E : Z*). (*Z*)‐Stilbene (5 : 95 *E : Z*) did not undergo *Z*‐to‐*E* isomerization, which indicated that the allyl group is required for this process. Finally, employing commercially supplied B(C_6_F_5_)_3_ **⋅** *n* H_2_O (10 mol %, *n*=0, 1), without purification by sublimation, resulted in no observable product formation (Scheme 3e), which reduces the likelihood of Brønsted acid catalysis being operative.^[13]^ Based on these synthetic studies, it can be postulated that the B(C_6_F_5_)_3_‐catalyzed isomerization of alkenes proceeds according to various pathways, including: i) hydride abstraction; ii) 1,2‐hydride shift; iii) 1,3‐hydride shift (Scheme 4). To gain further mechanistic insight, transition states (TSs) were located for the proposed hydride abstraction and 1,2‐hydride shift (TS2 and TS3, respectively; Scheme 4) at the M06‐2X/def2‐TZVPP/IEF‐PCM(Tol)//AM1/IEF‐PCM(Tol) level of theory.^[11]^ For these pathways, hydride migration proceeds with a small ΔΔ*G* ^≠^ of +0.2 kcal mol^−1^, respectively. The small ΔΔ*G* ^≠^ observed for TS2 and TS3 concur with experimentally observed deuterium scrambling (Scheme 3b and c), suggesting that multiple mechanisms are operative. A TS for the direct B(C_6_F_5_)_3_‐catalyzed 1,3‐hydride migration could not be located despite extensive efforts using various methods.^[11]^ Thus, the 1,3‐hydride migration cannot be discounted as a plausible mechanism following computational investigation.

## Conclusion

In conclusion, we have developed a new transition‐metal‐free approach for the isomerization of alkenes. The protocol is selective for the *E* isomer of the alkene product, employs B(C_6_F_5_)_3_ as a catalyst, and can be applied across a broad range of substrates to access useful products containing internal alkenes. Synthetic and computational studies suggest that multiple competing reaction mechanisms might be operative.

## Experimental Section

**General alkene isomerization procedure**: In the glovebox under Ar, an oven‐dried 10 mL microwave vial equipped with a magnetic stirrer bar was charged with B(C_6_F_5_)_3_ (10 mol %), alkene (0.2 mmol), and toluene (0.4 mL). The vial was sealed with an aluminium crimp cap and stirred at 150 °C for 24 h. The reaction mixture was cooled to RT, and 1,3,5‐trimethylbenzene (30 μL, 0.2 mmol) was added prior to analysis by ^1^H NMR. For product isolation, sat. aq. NaCl (0.4 mL) was added, and the organic phase was separated, dried over MgSO4, filtered, and concentrated in vacuo to give the crude product, which was purified by silica gel chromatography using the eluent stated in each case.

## Conflict of interest

The authors declare no conflict of interests.

1

## Supporting information

As a service to our authors and readers, this journal provides supporting information supplied by the authors. Such materials are peer reviewed and may be re‐organized for online delivery, but are not copy‐edited or typeset. Technical support issues arising from supporting information (other than missing files) should be addressed to the authors.

Supporting Information

## Data Availability

The data that support the findings of this study are openly available in Cardiff University data catalogue at https://doi.org/10.17035/d.2022.0216695569.
