# Supplementary material for: B(C6F5)3‐Catalyzed E‐Selective Isomerization of Alkenes
Source: Chemistry. 2022 Sep 1;28(63):e202202454. doi: 10.1002/chem.202202454 (PMC9804281; doi:10.1002/chem.202202454)
Supplement: Supplementary file 1 — Supporting Information [file CHEM-28-0-s001.pdf]

Chemistry–A European Journal

Supporting Information

$B(C_6F_5)_3$-Catalyzed *E*-Selective Isomerization of Alkenes

Betty A. Kustiana, Salma A. Elsherbeni, Thomas G. Linford-Wood, Rebecca L. Melen, Matthew N. Grayson, and Louis C. Morrill*

Contents

1. General information ... 2

2. Experimental and characterization data ... 3

2.1. Synthesis of substrates ... 3

2.1.1. General procedure [1] ... 3

2.1.2. General procedure 2 [15] ... 35

2.1.3. Deuterated substrates synthesis[21,22] ... 47

2.2. Optimization of the allyl isomerization ... 57

2.3. Substrate scope ... 58

2.3.1. General procedure 3 ... 58

2.3.2. Characterization of isomerization products ... 58

2.4. Mechanistic study ... 97

2.4.1. Isotopic solvent effect ... 97

2.4.2. Hydrogen isotope effect ... 98

2.4.3. *E*-/*Z*- products equilibrium ... 100

2.4.4. Crossover experiment ... 105

2.4.5. $B(C_6F_5)_3$•H_2O-catalyzed isomerization attempt ... 108

2.4.6. Computational method ... 109

3. References ... 118

1. General information

Unless stated otherwise, reactions were performed using oven-dried 10 mL microwave vials sealed with an aluminium crimp cap and were stirred with Teflon-coated magnetic stirrer bars. Dry toluene was obtained after previously degassed solvent with Schlenk technique through activated alumina columns (Mbraun, SPS-800). All other solvents and commercial reagents were kept with activated molecular sieves and used without further purification unless stated otherwise. All $B(C_6F_5)_3$-catalyzed isomerization reactions were prepared in the glovebox under argon atmosphere.

Room temperature (rt) refers to 20–25 ˚C. All reactions involving heating were conducted using DrySyn blocks and a contact thermometer. *In vacuo* refers to reduced pressure of rotary evaporator. $B(C_6F_5)_3$ was obtained commercially from Acros and purified by sublimation three times before use.

Analytical thin layer chromatography was performed using silica coated aluminium plates (Kieselgel 60 F254 silica) and visualization was obtained using ultraviolet light (254 nm). Flash chromatography used Kieselgel 60 silica with the eluent stated.

Melting points were obtained on a Gallenkamp melting point apparatus and corrected by linear interpolation of melting points standards benzophenone (47–49 ˚C), and benzoic acid (121–123 ˚C).

^{1}H, and ^{13}C NMR spectra were obtained on either a Bruker Avance 300 (300 MHz ^{1}H, 75 MHz ^{13}C) or a Bruker Avance 400 (400 MHz ^{1}H, 101 MHz ^{13}C) or a Bruker Avance 500 (500 MHz ^{1}H, 126 MHz ^{13}C) spectrometer at rt in the solvent stated. Chemical shifts are reported in parts per million (ppm) relative to the residual solvent signal. All coupling constants, *J*, are quoted in Hz. Multiplicities are reported as the following symbols: s = singlet, d = doublet, t = triplet, q = quartet, m = multiplet and multiples thereof. Resolved signals were obtained from the crude NMR spectra.

High resolution mass spectrometry (HRMS, m/z) data were obtained at Cardiff University on a Micromass LCT spectrometer. The NMR yields and the *E*/*Z* ratio were determined by integration of suitable baseline separated ^{1}H NMR signals.

2. Experimental and characterization data

2.1. Synthesis of substrates

2.1.1. General procedure [1]

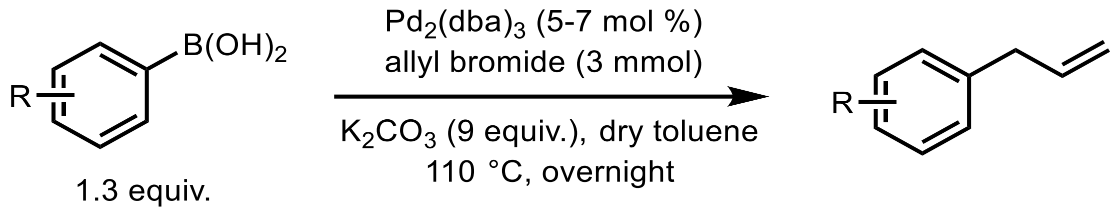

Under nitrogen, an oven-dried 250 mL three-necked round-bottomed flask with a stirrer bar and a condenser was charged with $Pd_2(dba)_3$ (5–7 mol %), K_2CO_3 (27 mmol), boronic acid (4 mmol), and dry toluene (56 mL), followed by the portionwise addition of allyl bromide (3 mmol) at rt with stirring. The reaction was heated up to 110 ˚C and left to stir overnight. The reaction was quenched with water (20 mL), the organic phase was separated. The aqueous phase was then extracted with Et_2O (2 x 10 mL). The combined organic phase was washed with brine (2 x 10 mL), dried over $MgSO_4$, filtered, and concentrated *in vacuo*.

1-Allyl-2-methoxybenzene[2]

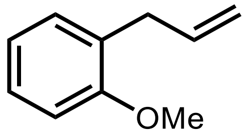

The title compound was prepared according to general procedure 1 using 2-methoxyphenylboronic acid (4 mmol). Purification by flash silica chromatography (eluent = 10% EtOAc in hexanes) gave the title compound as pale yellow liquid (66 mg, 15%); R_f: 0.67 (eluent = 10% EtOAc in hexanes); **^{1}H NMR (300 MHz, Chloroform-*d*)** δ 7.42 – 7.29 (m, 2H), 7.12 – 6.99 (m, 2H), 6.18 (ddt, *J* = 16.9, 10.3, 6.6 Hz, 1H), 5.29 – 5.16 (m, 2H), 3.99 (s, 3H), 3.57 (dt, *J* = 6.6, 1.5 Hz, 2H); **^{13}C NMR (75 MHz, Chloroform-*d*)** δ 157.3, 137.1, 129.9, 128.7, 127.4, 120.6, 115.4, 110.4, 55.4, 34.4; HRMS (EI$^+$) calculated $[C_{10}H_{12}O]^+$ $(M)^+$: m/z 148.0882, found 148.0882.

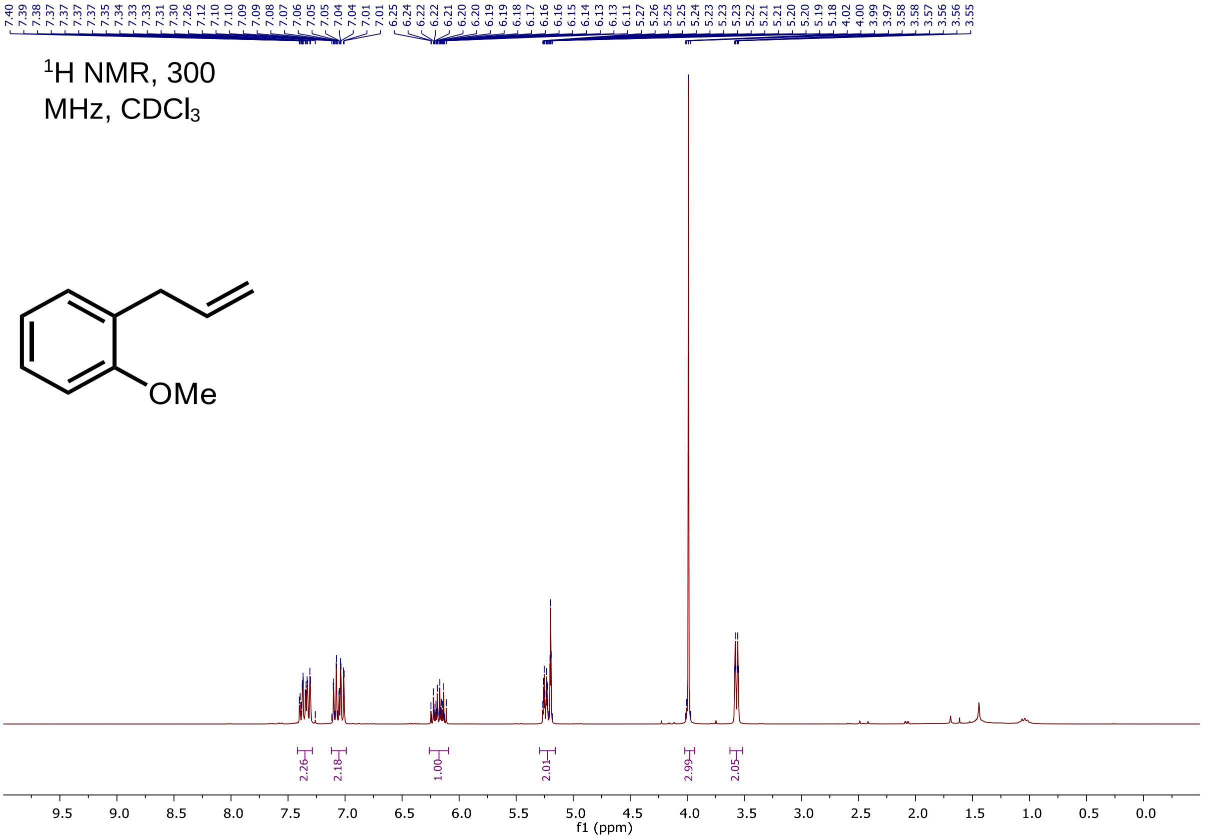
^{1}H NMR, 300 MHz, $CDCl_3$
OMe
7.40 7.39 7.38 7.37 7.37 7.37 7.35 7.34 7.33 7.33 7.31 7.30 7.26 7.12 7.10 7.10 7.09 7.09 7.08 7.07 7.06 7.05 7.05 7.04 7.04 7.01 7.01 6.25 6.24 6.22 6.22 6.21 6.20 6.20 6.19 6.19 6.18 6.17 6.16 6.16 6.15 6.14 6.13 6.13 6.11 5.27 5.26 5.25 5.25 5.24 5.23 5.23 5.23 5.22 5.21 5.21 5.20 5.20 5.19 5.18 4.02 4.00 3.99 3.97 3.58 3.58 3.57 3.56 3.56 3.55
2.26 2.18 1.00 2.01 2.99 2.05
9.5 9.0 8.5 8.0 7.5 7.0 6.5 6.0 5.5 5.0 4.5 4.0 3.5 3.0 2.5 2.0 1.5 1.0 0.5 0.0
f1 (ppm)

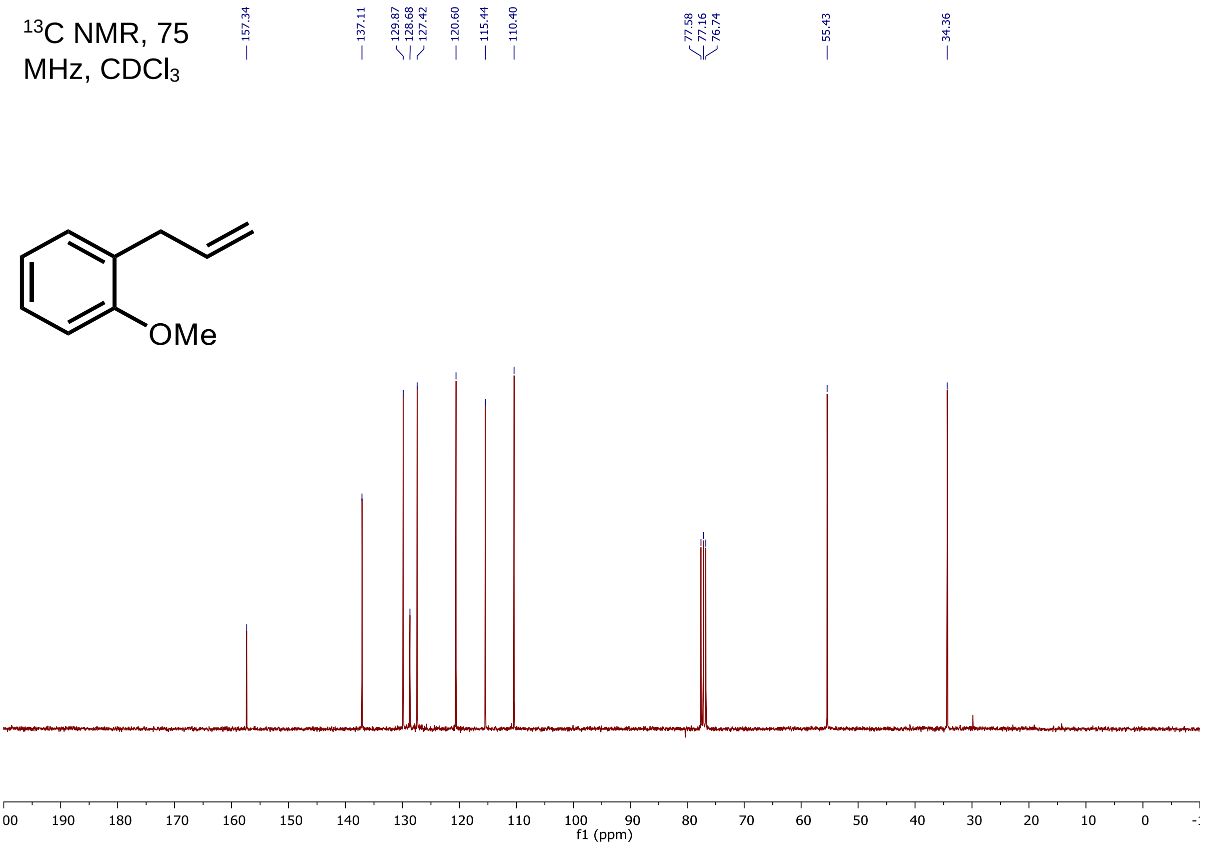
^{13}C NMR, 75 MHz, $CDCl_3$
OMe
157.34 137.11 129.87 128.68 127.42 120.60 115.44 110.40 77.58 77.16 76.74 55.43 34.36
00 190 180 170 160 150 140 130 120 110 100 90 80 70 60 50 40 30 20 10 0
f1 (ppm)

1-Allyl-3-methoxybenzene[3]

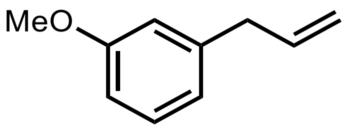

The title compound was prepared according to general procedure 1 using 3-methoxyphenylboronic acid (4 mmol). Purification by flash silica chromatography (eluent = 10% EtOAc in hexanes) gave the title compound as colourless liquid (0.24 g, 55%); R_f: 0.61 (eluent = 10% EtOAc in hexanes); **^{1}H NMR (500 MHz, Chloroform-*d*)** δ 7.27 – 7.22 (m, 1H), 6.85 – 6.77 (m, 3H), 6.00 (ddt, *J* = 16.8, 10.0, 6.7 Hz, 1H), 5.17 – 5.08 (m, 2H), 3.82 (s, 3H), 3.42 – 3.38 (m, 2H); **^{13}C NMR (126 MHz, Chloroform-*d*)** δ 159.8, 141.8, 137.4, 129.5, 121.1, 116.0, 114.4, 111.6, 55.3, 40.4; HRMS (EI^+) calculated $[C_{10}H_{12}O]^+$ $(M)^+$: m/z 148.0882, found 148.0881.

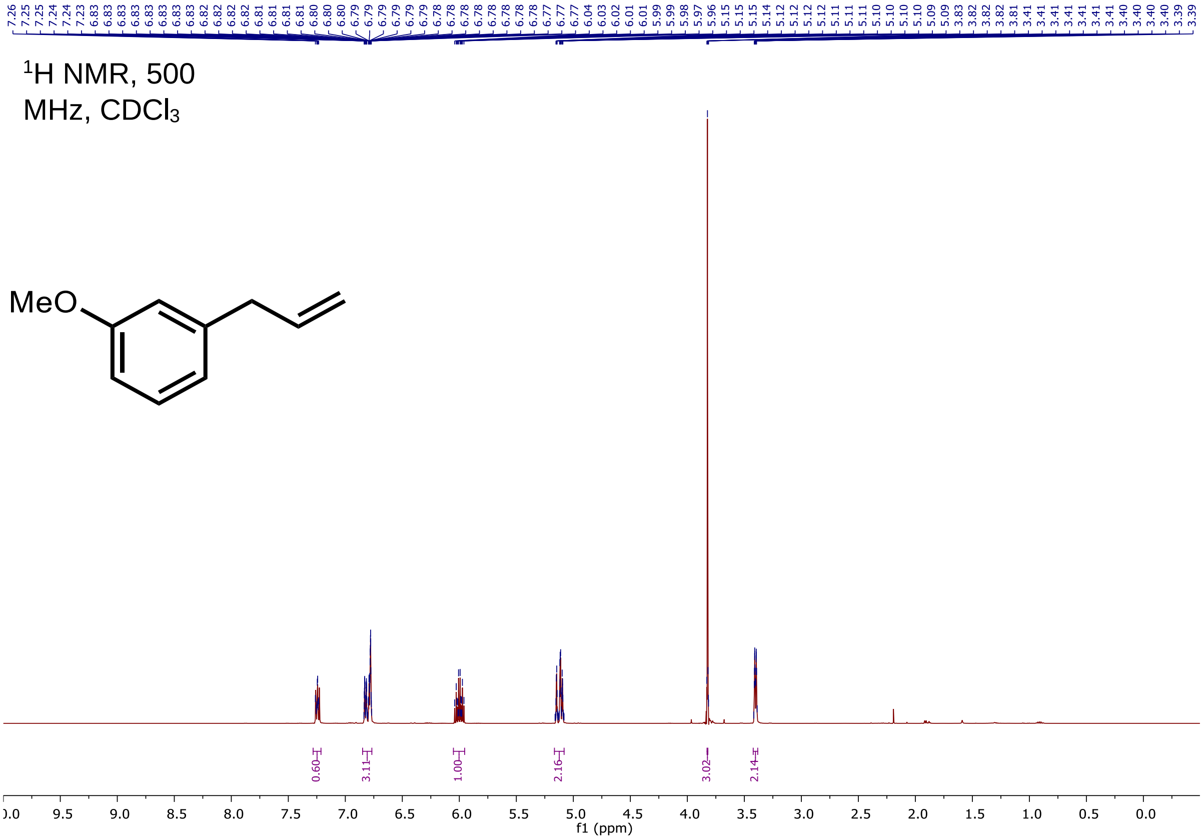

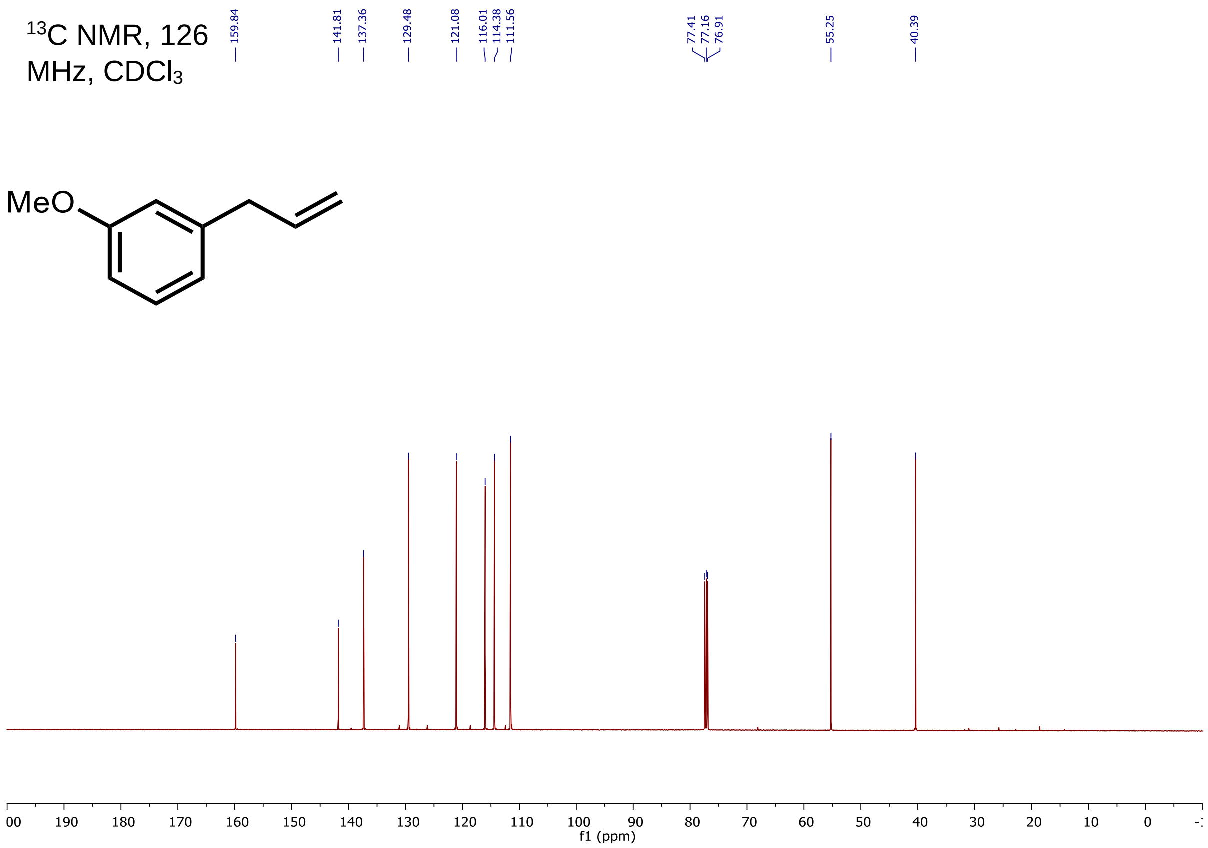

1-Allyl-4-(phenoxy)benzene[4]

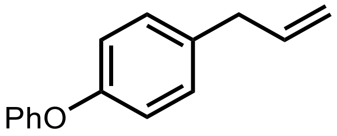

The title compound was prepared according to general procedure 1 using (4-(phenoxy)phenyl)lboronic acid (4 mmol). Purification by flash silica chromatography (eluent = 5% EtOAc in PE) gave the title compound as colourless liquid (0.51 g, 81%); R_f: 0.69 (eluent = 5% EtOAc in PE); **^{1}H NMR (300 MHz, Chloroform-*d*)** δ 7.36 – 7.31 (m, 2H), 7.21 – 7.14 (m, 2H), 7.13 – 7.06 (m, 1H), 7.06 – 6.99 (m, 2H), 6.99 – 6.96 (m, 2H), 6.00 (ddt, J = 15.8, 10.5, 6.7 Hz, 1H), 5.16 – 5.06 (m, 2H), 3.39 (dt, J = 6.8, 1.4 Hz, 2H); **^{13}C NMR (75 MHz, Chloroform-*d*)** δ 157.7, 155.5, 137.6, 135.1, 129.9, 129.8, 123.1, 119.2, 118.7, 115.9, 39.6; HRMS (CI$^+$) calculated $[C_{15}H_{14}O]^+$ (M)$^+$: m/z 210.1039, found 210.1040.

^{1}H NMR, 300 MHz, $CDCl_3$

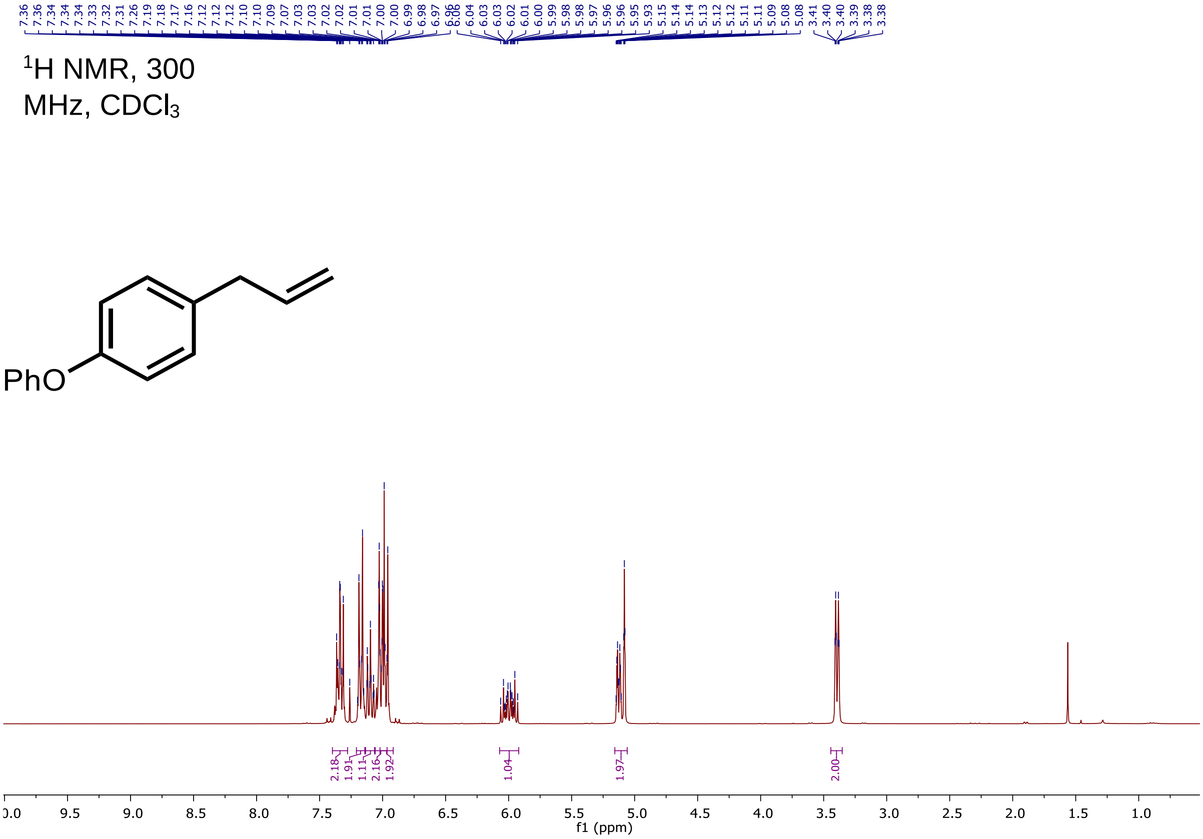

^{13}C NMR, 75 MHz, $CDCl_3$

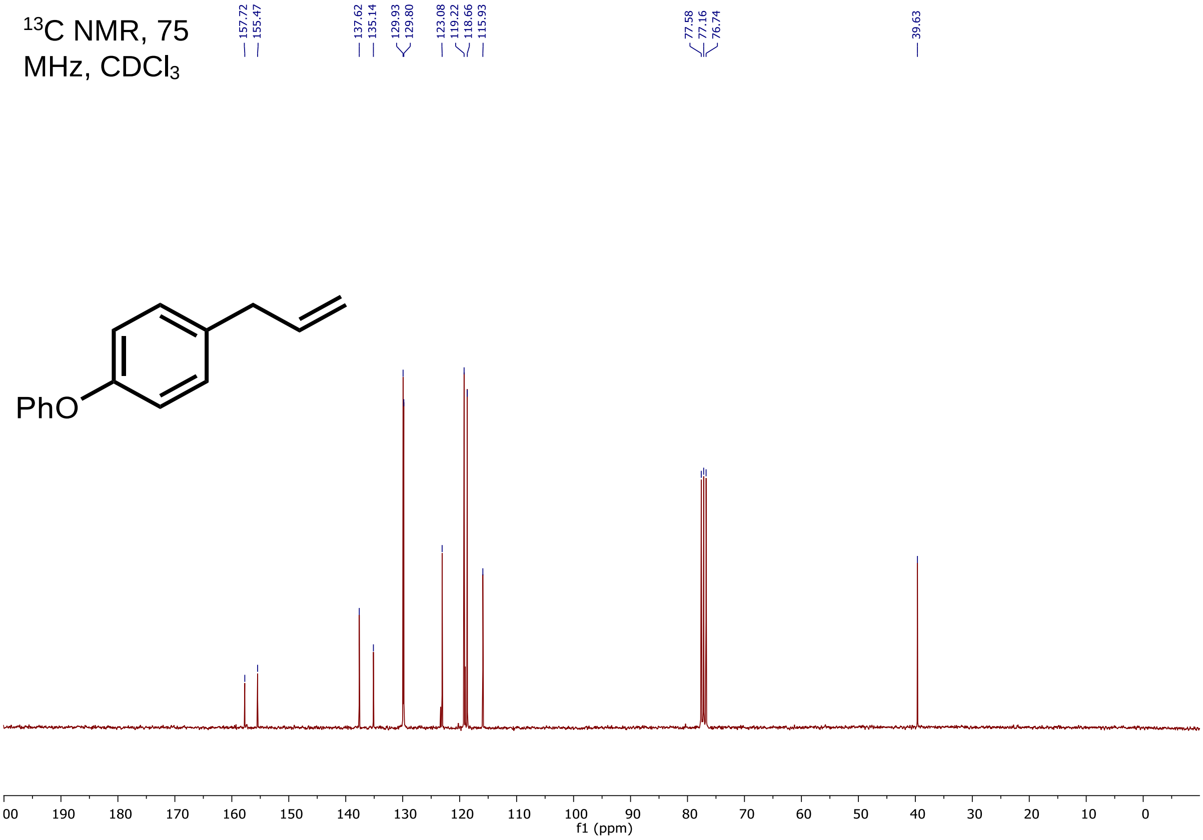

1-Allyl-4-(benzyloxy)benzene[5]

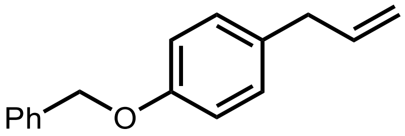

The title compound was prepared according to general procedure 1 using (4-(benzyloxy)phenyl)lboronic acid (4 mmol). Purification by flash silica chromatography (eluent = 10% EtOAc in hexanes) gave the title compound as colourless liquid (0.37 g, 55%); R_f: 0.63 (eluent = 10% EtOAc in hexanes); **^{1}H NMR (300 MHz, Chloroform-*d*)** δ 7.55 – 7.33 (m, 5H), 7.22 – 7.14 (m, 2H), 7.02 – 6.96 (m, 2H), 6.03 (ddt, J = 16.9, 10.3, 6.7 Hz, 1H), 5.21 – 5.10 (m, 2H), 5.10 (s, 2H), 3.40 (dt, J = 6.8, 1.5 Hz, 2H); **^{13}C NMR (75 MHz, Chloroform-*d*)** δ 157.3, 137.9, 137.3, 132.5, 129.6, 128.7, 127.9, 127.6, 115.6, 114.9, 70.1, 39.5; HRMS (EI$^+$) calculated $[C_{16}H_{16}O]^+$ (M)$^+$: m/z 224.1195, found 224.1198.

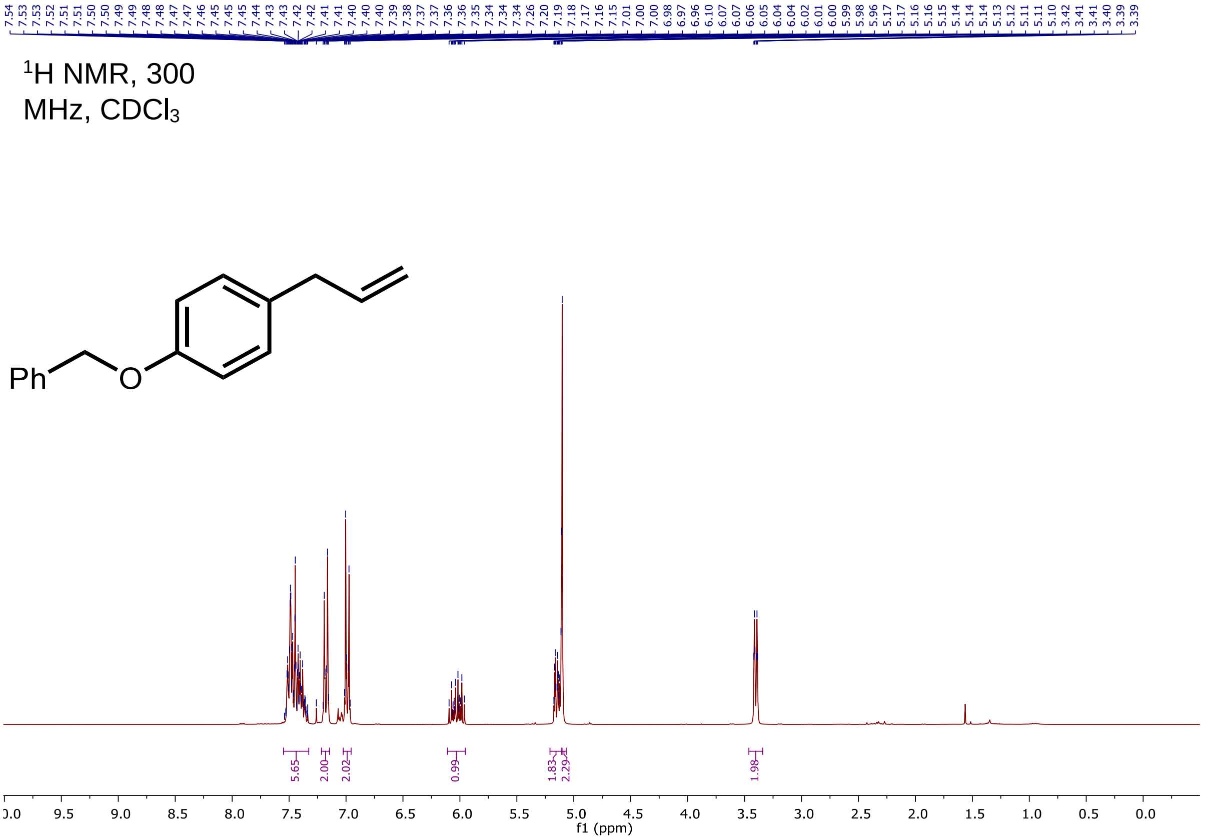

^{13}C NMR, 75 MHz, $CDCl_3$

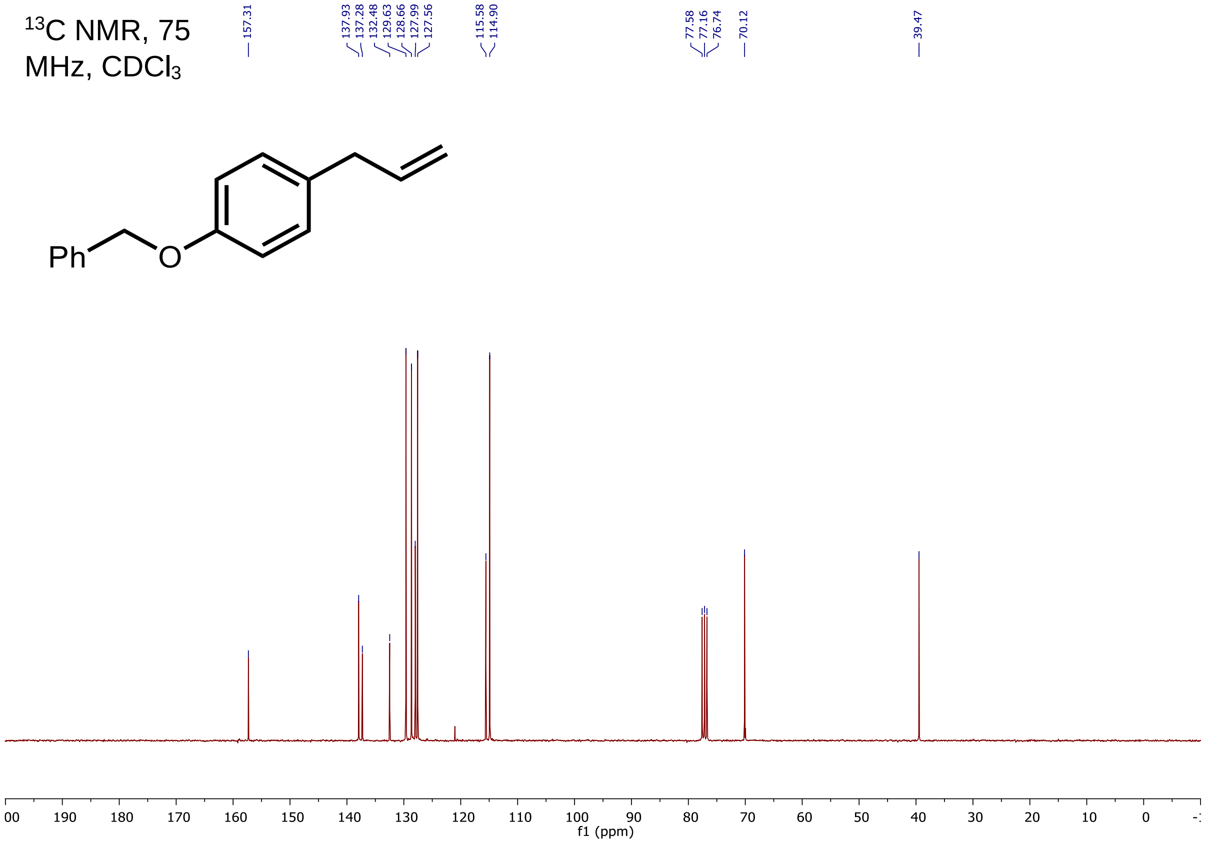

(2-Allylphenyl)methyl)sulfane[6]

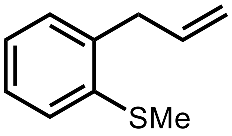

The title compound was prepared according to general procedure 1 using 2-(methylthio)phenylboronic acid (4 mmol). Purification by flash silica chromatography (eluent = 10% EtOAc in hexanes) gave the title compound as pale yellow liquid (0.38 g, 77%); R_f: 0.67 (eluent = 10% EtOAc in hexanes); **^{1}H NMR (300 MHz, Chloroform-*d*)** δ 7.25 – 7.21 (m, 2H), 7.18 – 7.09 (m, 2H), 6.00 (ddt, J = 16.8, 10.3, 6.5 Hz, 1H), 5.16 – 5.02 (m, 2H), 3.50 (dt, J = 6.6, 1.7 Hz, 2H), 2.47 (s, 3H); **^{13}C NMR (75 MHz, Chloroform-*d*)** δ 138.0, 137.5, 136.2, 129.3, 127.1, 125.7, 125.1, 116.4, 37.8, 15.9; HRMS (EI^+) calculated $[C_{10}H_{12}S]^+$ $(M)^+$: m/z 164.0654, found 164.0653.

^{1}H NMR, 300 MHz, $CDCl_3$

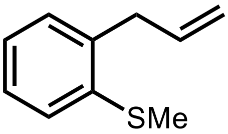

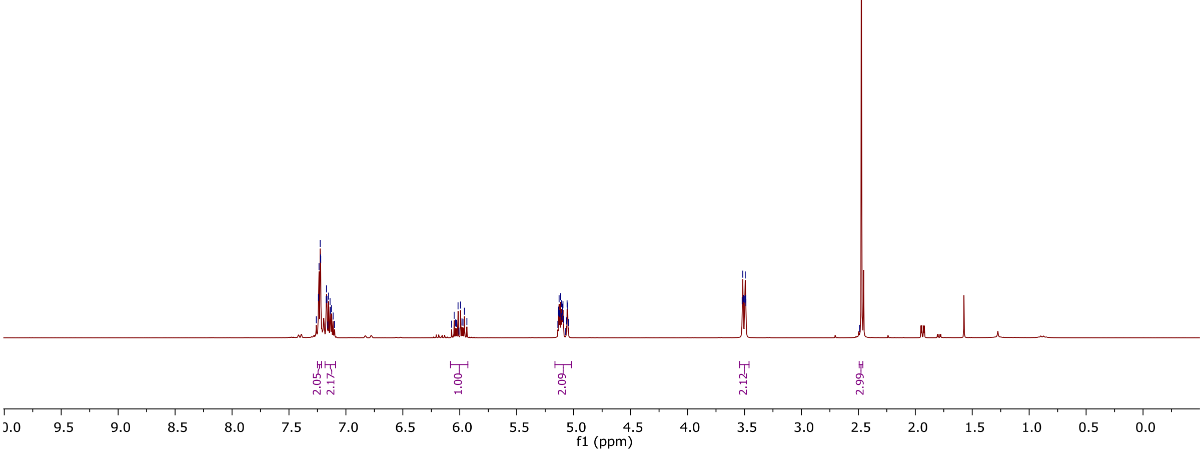

^{13}C NMR, 75 MHz, $CDCl_3$

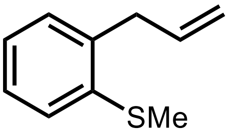

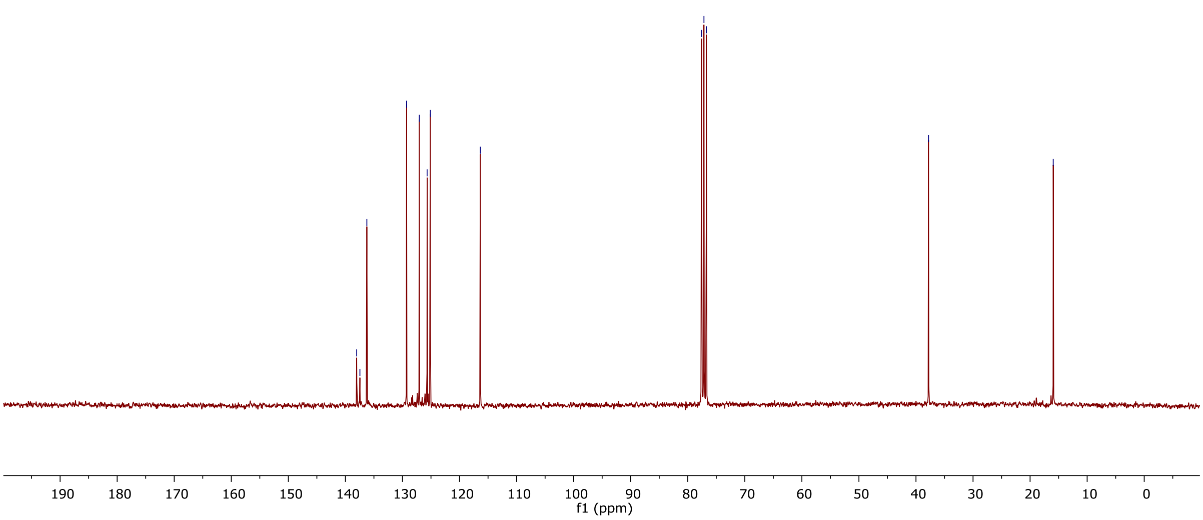

(3-(Allylphenyl)methyl)sulfane[7]

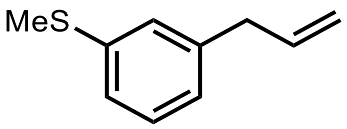

The title compound was prepared according to general procedure 1 using 3-(methylthio)phenylboronic acid (4 mmol). Purification by flash silica chromatography (eluent = 10% EtOAc in hexanes) gave the title compound as pale yellow liquid (0.44 g, 90%); R_f: 0.71 (eluent = 10% EtOAc in hexanes); ν_{max} / cm^{-1} (film) 2976, 2918, 1638, 1589, 1570, 1474, 1422, 1086, 993; **^{1}H NMR (500 MHz, Chloroform-*d*)** δ 7.32 – 7.25 (m, 1H), 7.21 – 7.14 (m, 2H), 7.04 (ddt, *J* = 7.6, 1.7, 1.2, 1H), 6.03 (ddt, *J* = 16.9, 10.2, 6.7 Hz, 1H), 5.21 – 5.11 (m, 2H), 3.48 – 3.39 (m, 2H), 2.54 (s, 3H); **^{13}C NMR (126 MHz, Chloroform-*d*)** δ 140.8, 138.5, 137.1, 128.9, 126.9, 125.5, 124.4, 116.2, 40.2, 15.9; HRMS (EI^+) calculated $[C_{10}H_{12}S]^+$ $(M)^+$: m/z 164.0654, found 164.0653. The compound was previously reported, but not spectroscopically characterized.

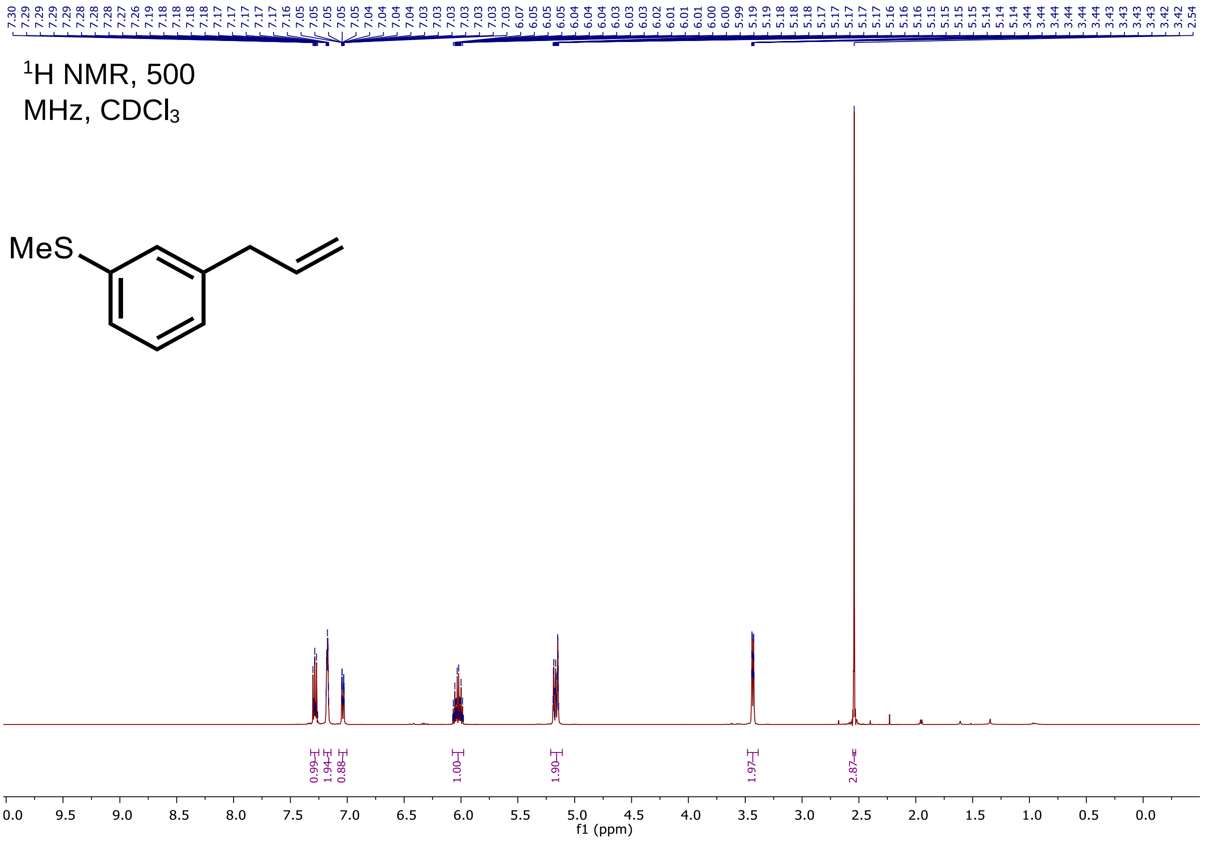

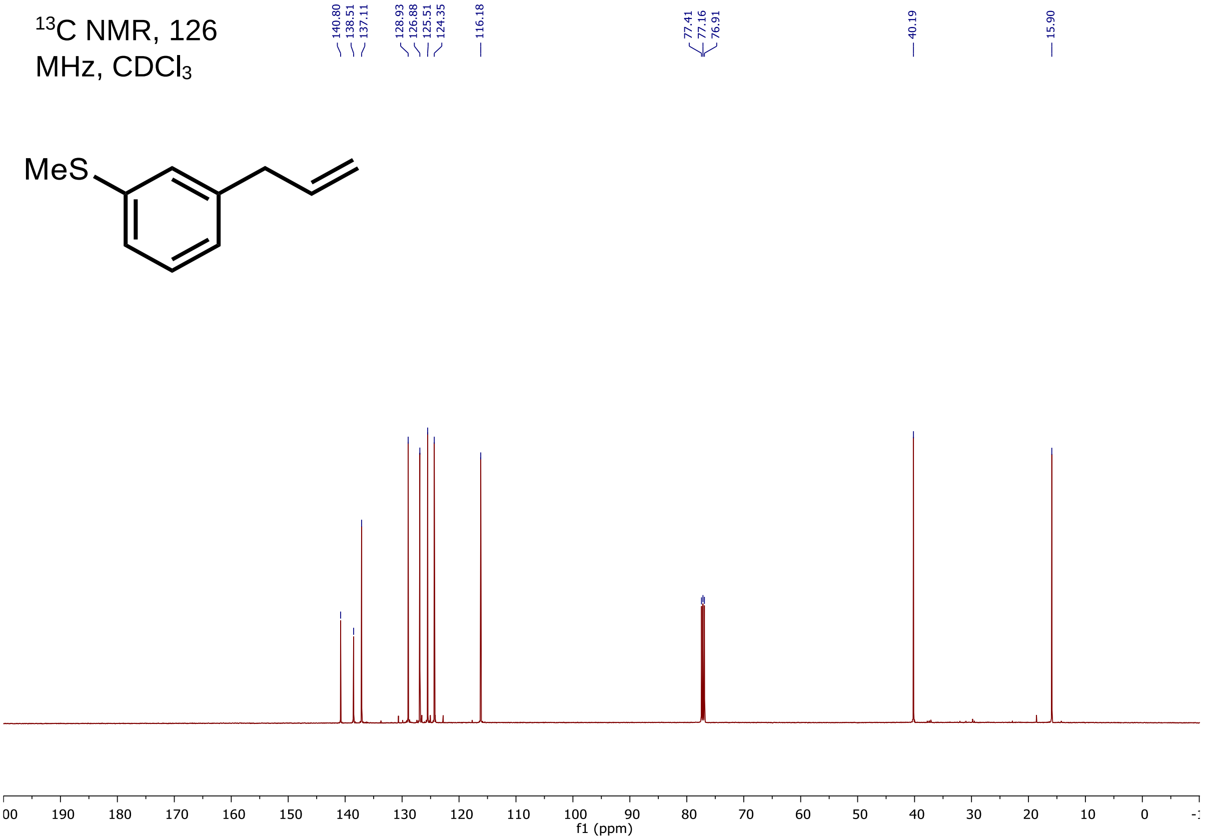

(4-(Allylphenyl)methyl)sulfane[8]

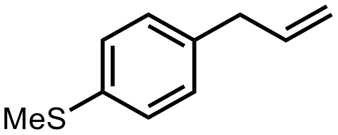

The title compound was prepared according to general procedure 1 using 4-(methylthio)phenylboronic acid (4 mmol). Purification by flash silica chromatography (eluent = 10% EtOAc in hexanes) gave the title compound as yellow liquid (0.32 g, 65%); R_f: 0.71 (eluent = 10% EtOAc in hexanes); **^{1}H NMR (500 MHz, Chloroform-*d*)** δ 7.25 – 7.21 (m, 2H), 7.16 – 7.12 (m, 2H), 6.03 – 5.91 (m, 1H), 5.13 – 5.05 (m, 2H), 3.41 – 3.34 (m, 2H), 2.48 (s, 3H); **^{13}C NMR (126 MHz, Chloroform-*d*)** δ 137.4, 137.2, 135.8, 129.2, 127.3, 115.9, 39.8, 16.4; HRMS (EI^+) calculated $[C_{10}H_{12}S]^+$ $(M)^+$: m/z 164.0654, found 164.0653.

^{1}H NMR, 500 MHz, $CDCl_3$

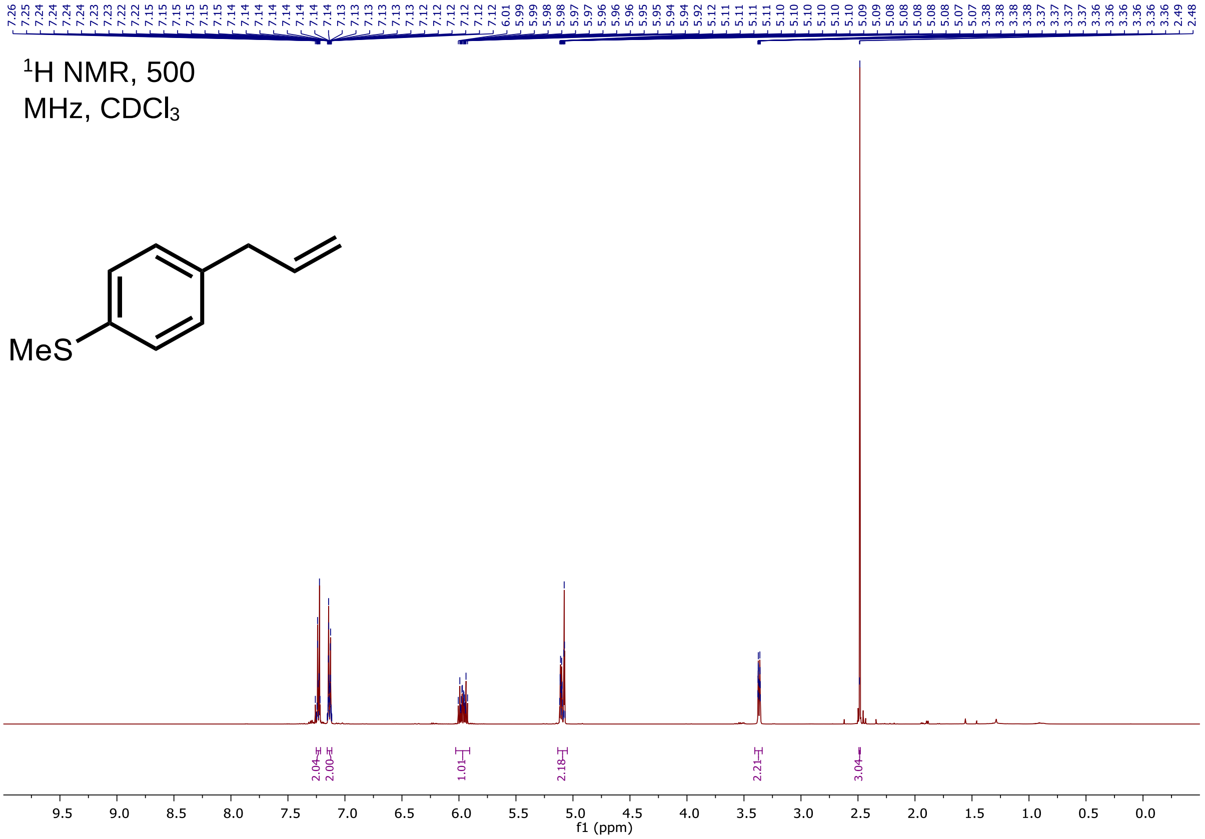

^{13}C NMR, 126 MHz, $CDCl_3$

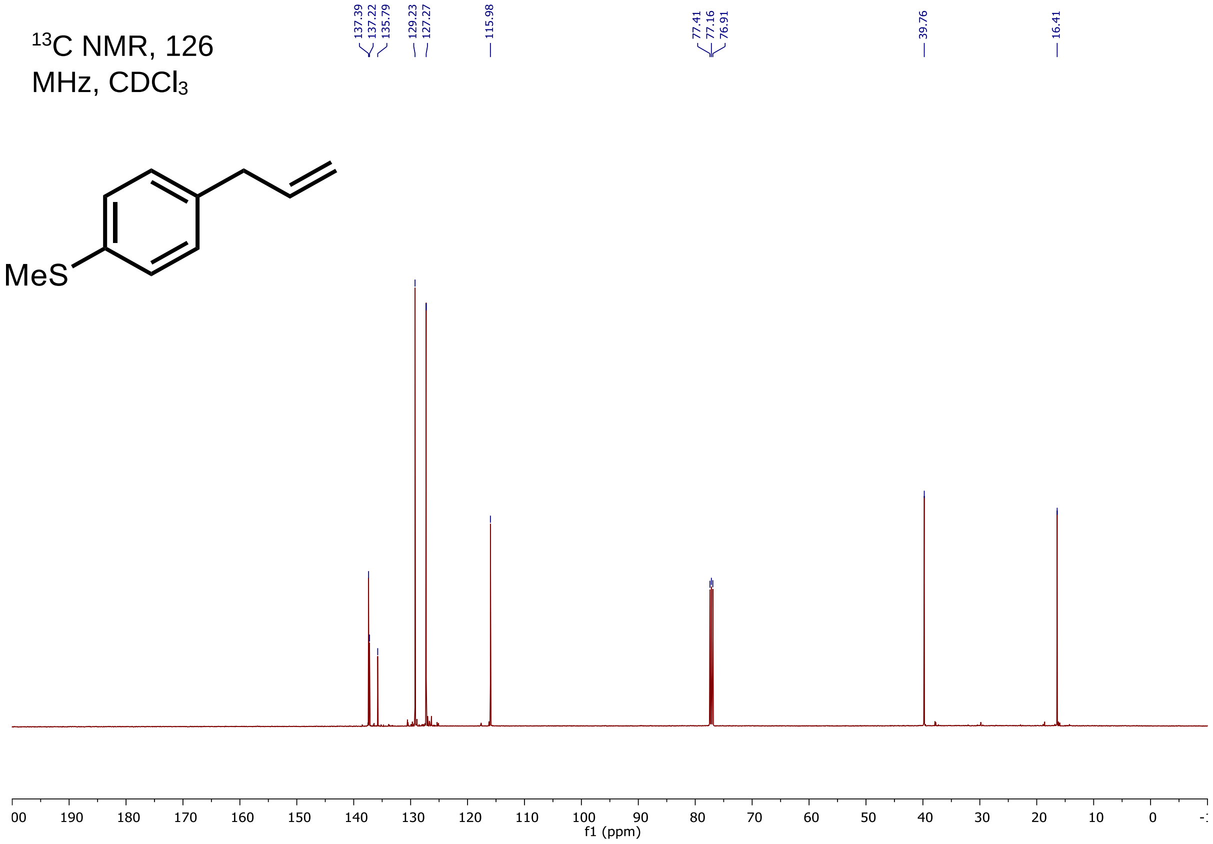

(2-Allylphenoxy)(*tert*-butyl)dimethylsilane[3]

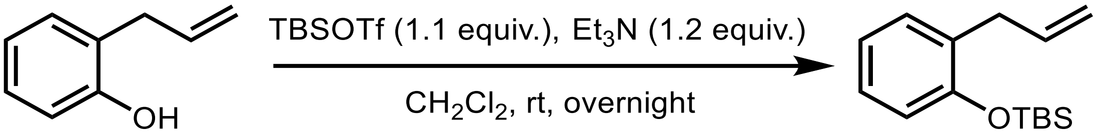

Under nitrogen, an oven-dried three-necked round-bottomed flask with a stirrer bar was charged with 2-allylphenol (5 mmol), Et_3N (6 mmol), and CH_2Cl_2 (10 mL). The mixture was stirred at rt for 2 h. TBSOTf (5.5 mmol) was then added dropwise at rt then the reaction was left stirred at rt for overnight. The reaction was quenched with sat. NH_4Cl (20 mL), extracted with Et_2O (3 x 10 mL), dried over $MgSO_4$, filtered, and concentrated *in vacuo*. Purification by deactivated flash silica chromatography (eluent = 100% PE) gave the title compound as colourless liquid (1.09 g, 88%); R_f: 0.62 (eluent = 100% PE); **^{1}H NMR (500 MHz, Chloroform-*d*)** δ 7.19 (m, 1H), 7.16 – 7.11 (m, 1H), 6.95 (td, *J* = 7.4, 1.2 Hz, 1H), 6.85 (dd, *J* = 8.0, 1.2 Hz, 1H), 6.09 – 5.98 (m, 1H), 5.13 – 5.06 (m, 2H), 3.43 (dt, *J* = 6.5, 1.6 Hz, 2H), 1.08 (s, 9H), 0.29 (s, 6H); **^{13}C NMR (126 MHz, Chloroform-*d*)** δ 153.5, 137.2, 130.8, 130.3, 127.2, 121.2, 118.6, 115.6, 34.6, 25.9, 18.4, -3.9; HRMS (EI$^+$) calculated $[C_{15}H_{24}OSi]^+$ $(M)^+$: m/z 248.1591, found 248.1591.

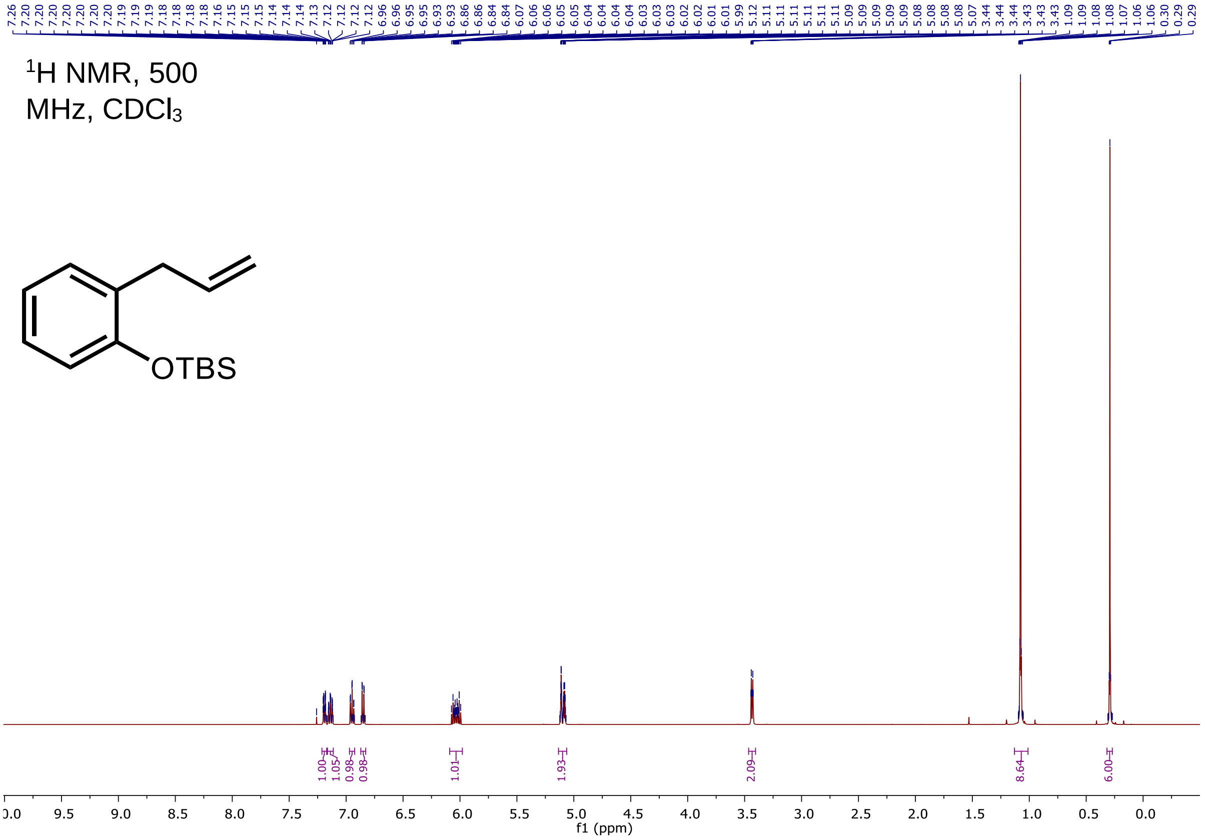

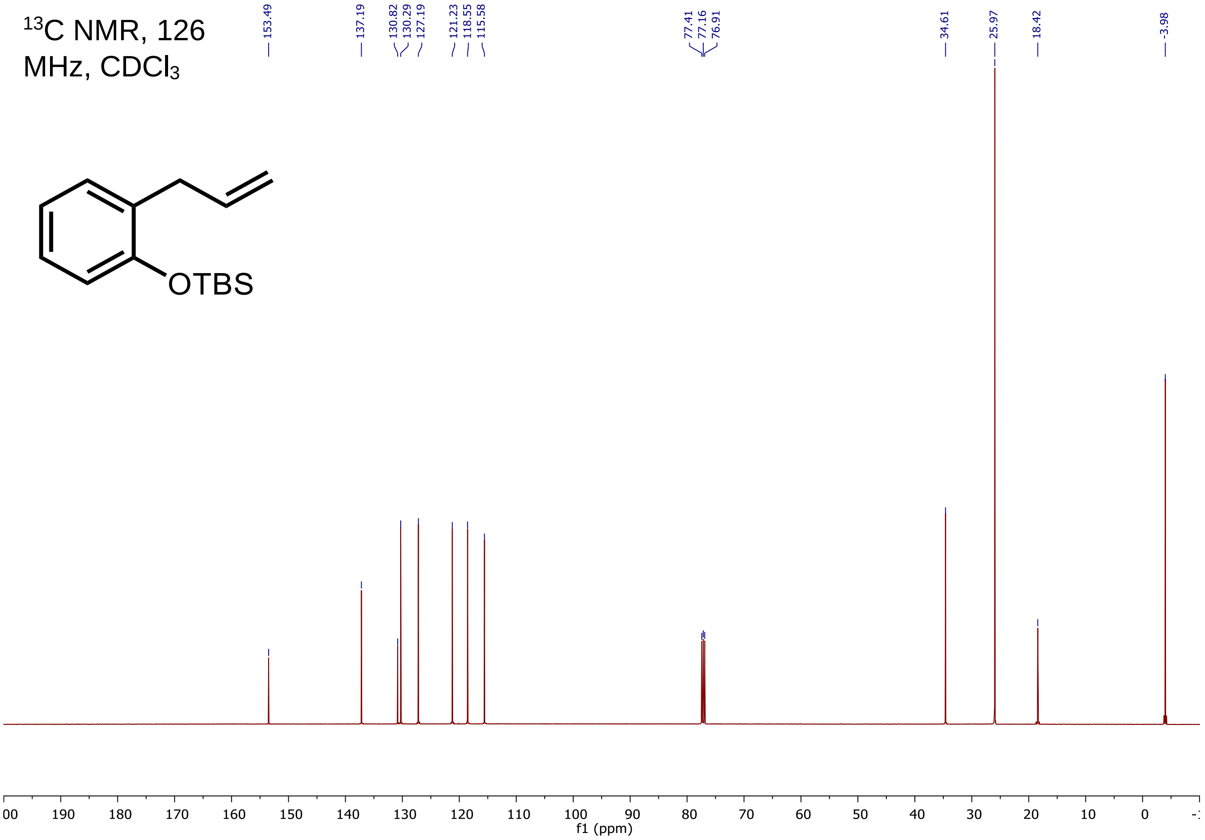

1-Allyl-3-methylbenzene[9]

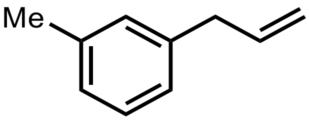

The title compound was prepared according to general procedure 1 using 3-methylbenzeneboronic acid (4 mmol). Purification by flash silica chromatography (eluent = 10% EtOAc in hexanes) gave the title compound as colourless liquid (0.20 g, 51%); R_f: 0.86 (eluent = 10% EtOAc in hexanes); **^{1}H NMR (500 MHz, Chloroform-*d*)** δ 7.24 – 7.19 (m, 1H), 7.06 – 7.00 (m, 3H), 6.08 – 5.93 (m, 1H), 5.18 – 5.04 (m, 2H), 3.38 (dt, *J* = 6.8, 1.4 Hz, 2H), 2.36 (d, *J* = 0.8 Hz, 3H); **^{13}C NMR (126 MHz, Chloroform-*d*)** δ 140.1, 138.1, 137.7, 129.5, 128.5, 126.9, 125.7, 115.8, 40.4, 21.5; HRMS (EI^+) calculated $[C_{10}H_{12}]^+$ $(M)^+$: m/z 132.0933, found 132.0934.

^{1}H NMR, 500 MHz, CDCl$_3$

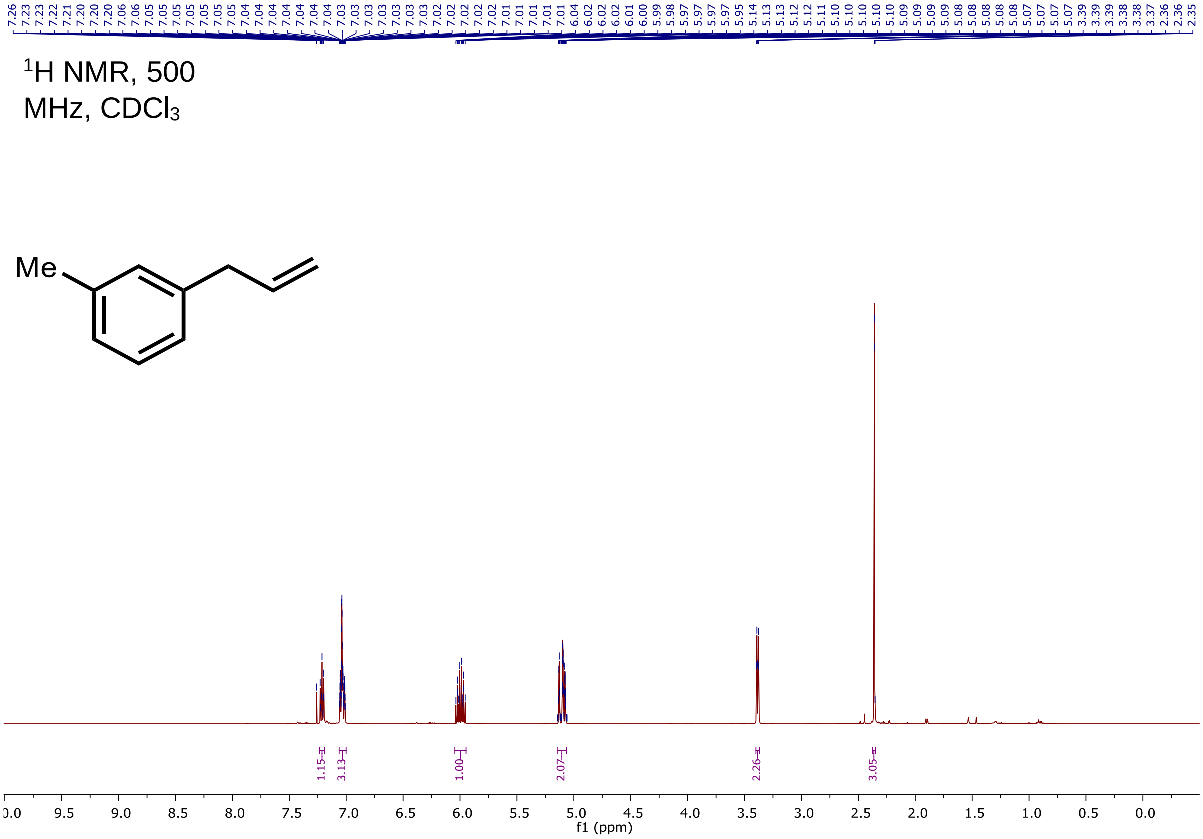

^{13}C NMR, 126 MHz, CDCl$_3$

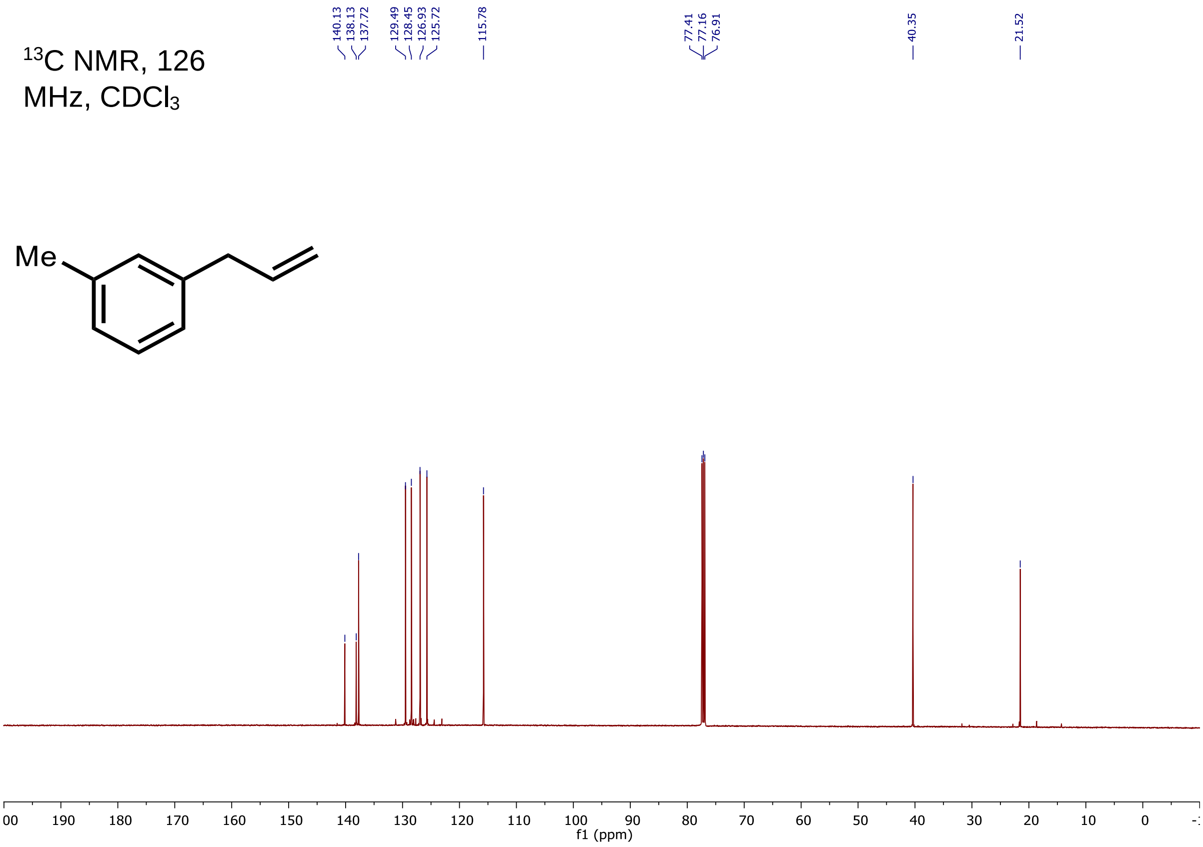

1-Allyl-4-methylbenzene[9]

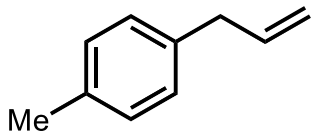

The title compound was prepared according to general procedure 1 using 4-methylphenylboronic acid (4 mmol). Purification by flash silica chromatography (eluent = 10% EtOAc in hexanes) gave the title compound as colourless liquid (0.20 g, 50%); R_f: 0.72 (eluent = 10% EtOAc in hexanes); **^{1}H NMR (500 MHz, Chloroform-*d*)** δ 7.18 – 7.06 (m, 4H), 5.98 (ddt, *J* = 16.8, 10.0, 6.7, 1H), 5.15 – 5.02 (m, 2H), 3.43 – 3.32 (m, 2H), 2.34 (d, *J* = 1.7 Hz, 3H)); **^{13}C NMR (126 MHz, Chloroform-*d*)** δ 137.9, 137.1, 135.7, 129.2, 128.6, 115.7, 39.9, 21.2; HRMS (EI$^+$) calculated $[C_{10}H_{12}]^+$ (M)$^+$: m/z 132.0933, found 132.0934.

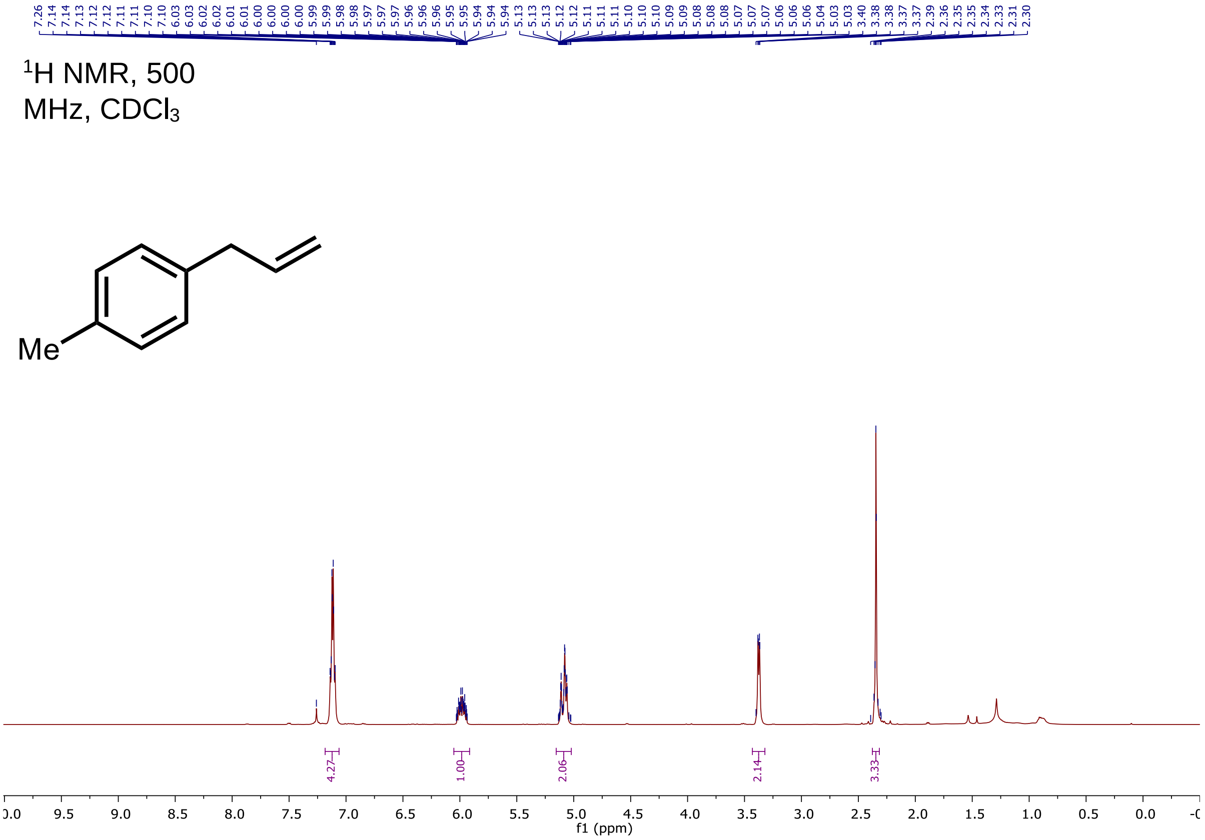

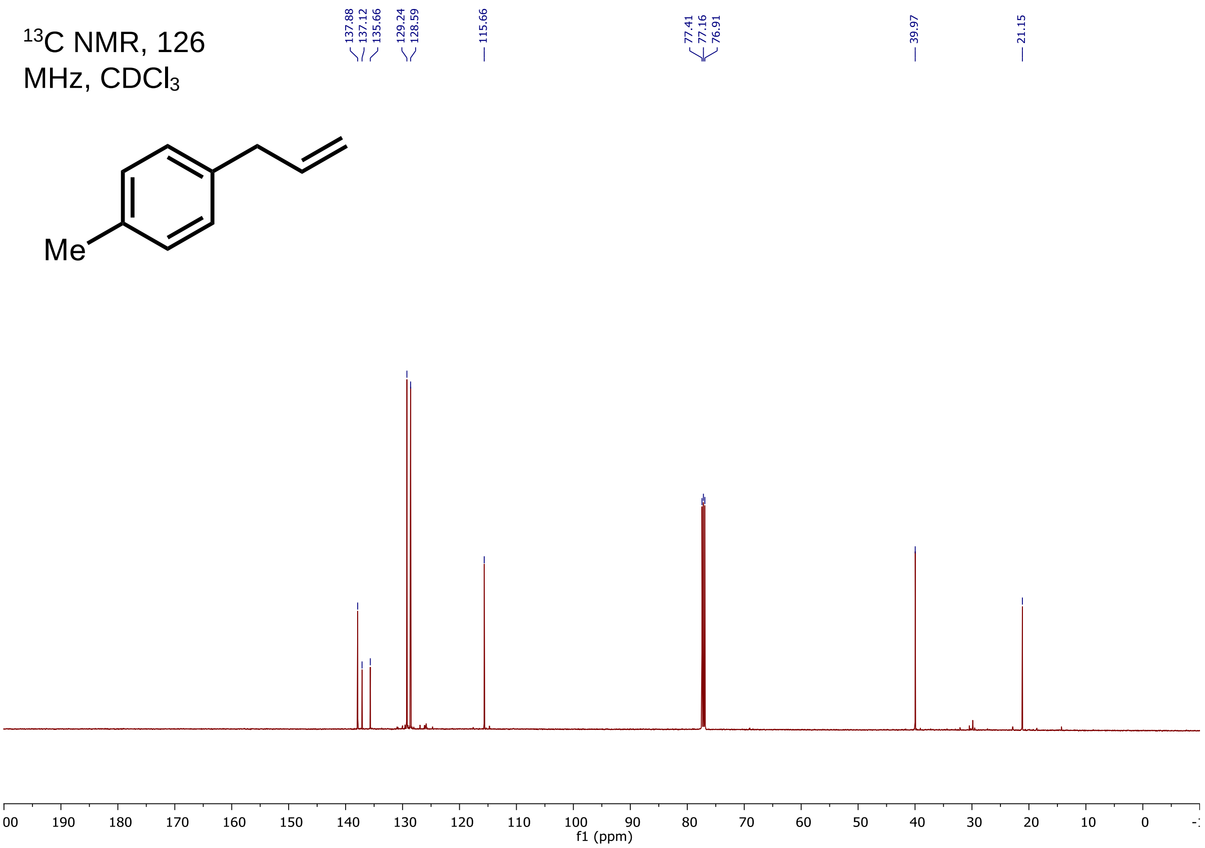

2-Allyl-1,3-dimethylbenzene[10]

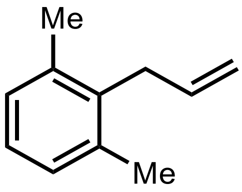

The title compound was prepared according to general procedure 1 using 2,6-dimethylphenylboronic acid (4 mmol). Purification by flash silica chromatography (eluent = 10% EtOAc in hexanes) gave the title compound as colourless liquid (0.26 g, 60%); R_f: 0.58 (eluent = 10% EtOAc in hexanes); **^{1}H NMR (500 MHz, Chloroform-*d*)** δ 7.09 – 7.04 (m, 3H), 5.94 (ddt, *J* = 17.1, 10.2, 5.7 Hz, 1H), 5.03 (dq, *J* = 10.2, 1.8 Hz, 1H), 4.89 (dq, *J* = 17.1, 1.9 Hz, 1H), 3.43 (dt, *J* = 5.7, 1.9 Hz, 2H), 2.33 (d, *J* = 0.8 Hz, 6H); **^{13}C NMR (126 MHz, Chloroform-*d*)** δ 136.8, 136.2, 135.4, 128.1, 126.2, 114.9, 33.8, 19.9; HRMS (EI^+) calculated $[C_{11}H_{14}]^+$ $(M)^+$: m/z 146.1090, found 146.1091.

^{1}H NMR, 500 MHz, $CDCl_3$

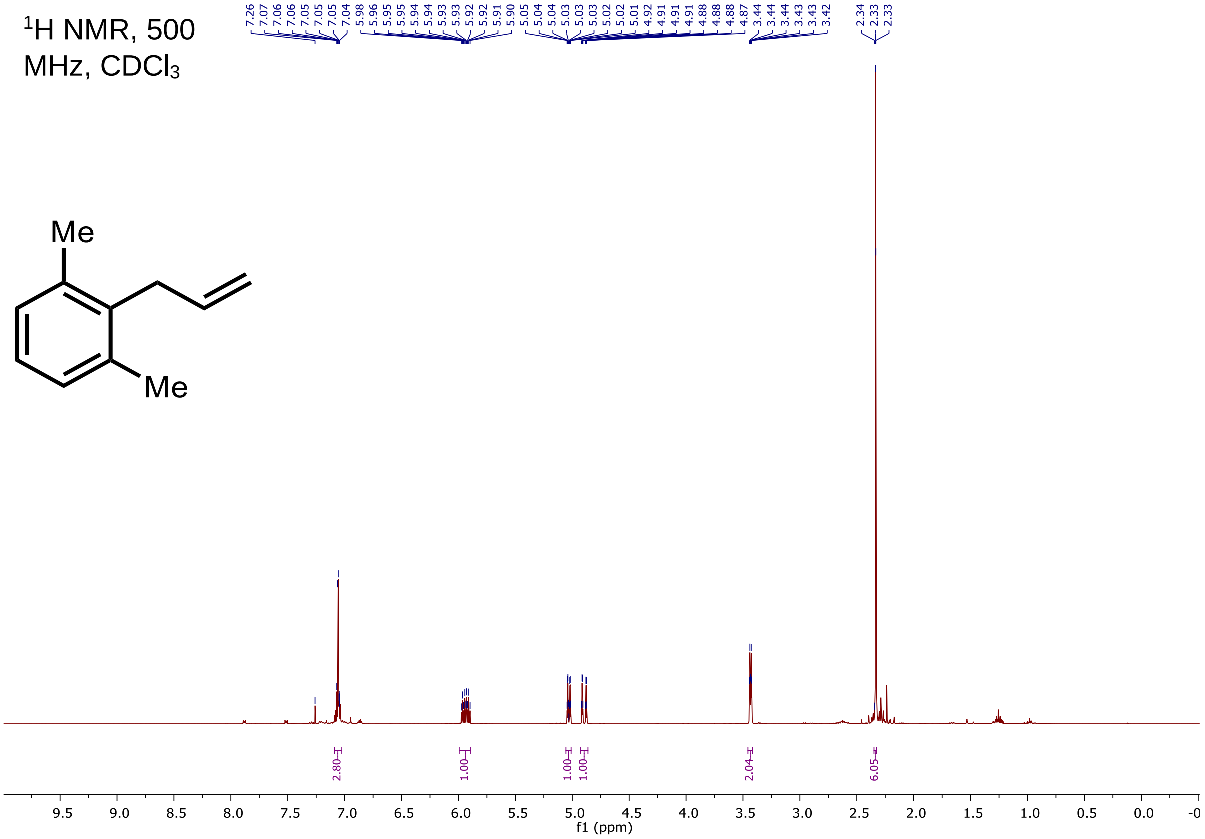

^{13}C NMR, 126 MHz, $CDCl_3$

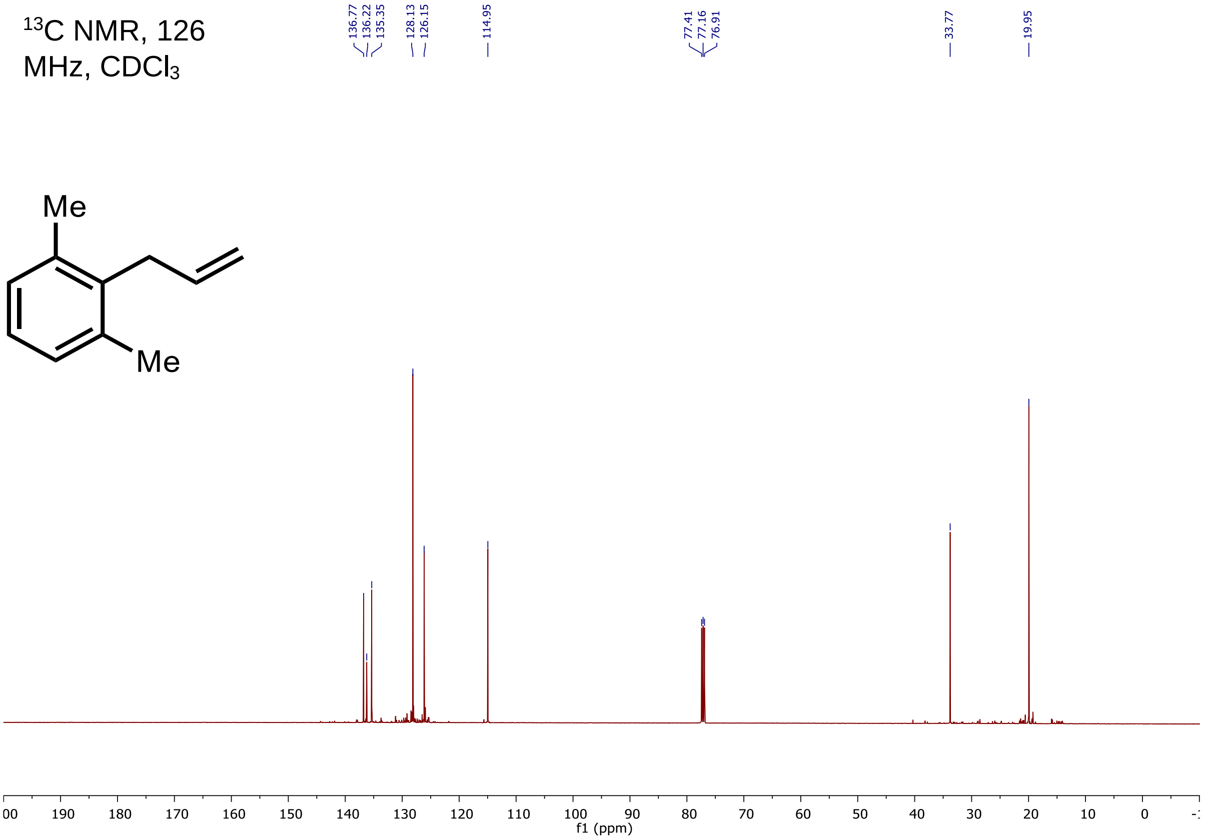

2-Allyl-1,3-diisopropylbenzene[11]

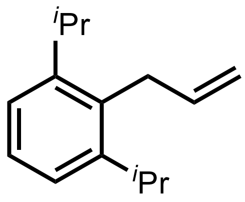

The title compound was prepared according to general procedure 1 using 2,6-isopropylphenylboronic acid (4 mmol). Purification by flash silica chromatography (eluent = 10% EtOAc in hexanes) gave the title compound as colourless liquid (0.24 g, 40%); R_f: 0.81 (eluent = 10% EtOAc in hexanes); ν_{max} / cm^{-1} (film) 2961, 2926, 2868, 1636, 1582, 1462, 1383, 1362, 1055, 910; **^{1}H NMR (500 MHz, Chloroform-*d*)** δ 7.31 (dd, *J* = 8.5, 6.9 Hz, 1H), 7.25 – 7.21 (m, 2H), 6.09 (ddt, *J* = 17.2, 10.3, 5.2 Hz, 1H), 5.10 (dq, *J* = 10.2, 1.9 Hz, 1H), 4.89 (dq, *J* = 17.2, 2.0 Hz, 1H), 3.58 (dt, *J* = 5.2, 2.0 Hz, 2H), 3.22 (hept, *J* = 6.8 Hz, 2H), 1.30 (dd, *J* = 6.9, 0.6 Hz, 12H); **^{13}C NMR (126 MHz, Chloroform-*d*)** δ 147.5, 137.7, 133.1, 126.8, 122.9, 115.2, 31.7, 29.4, 24.4. LRMS (EI) [$C_{15}H_{21}$] $(M-H)^{-}$: m/z 201.16. The compound was previously reported, but not spectroscopically characterized.

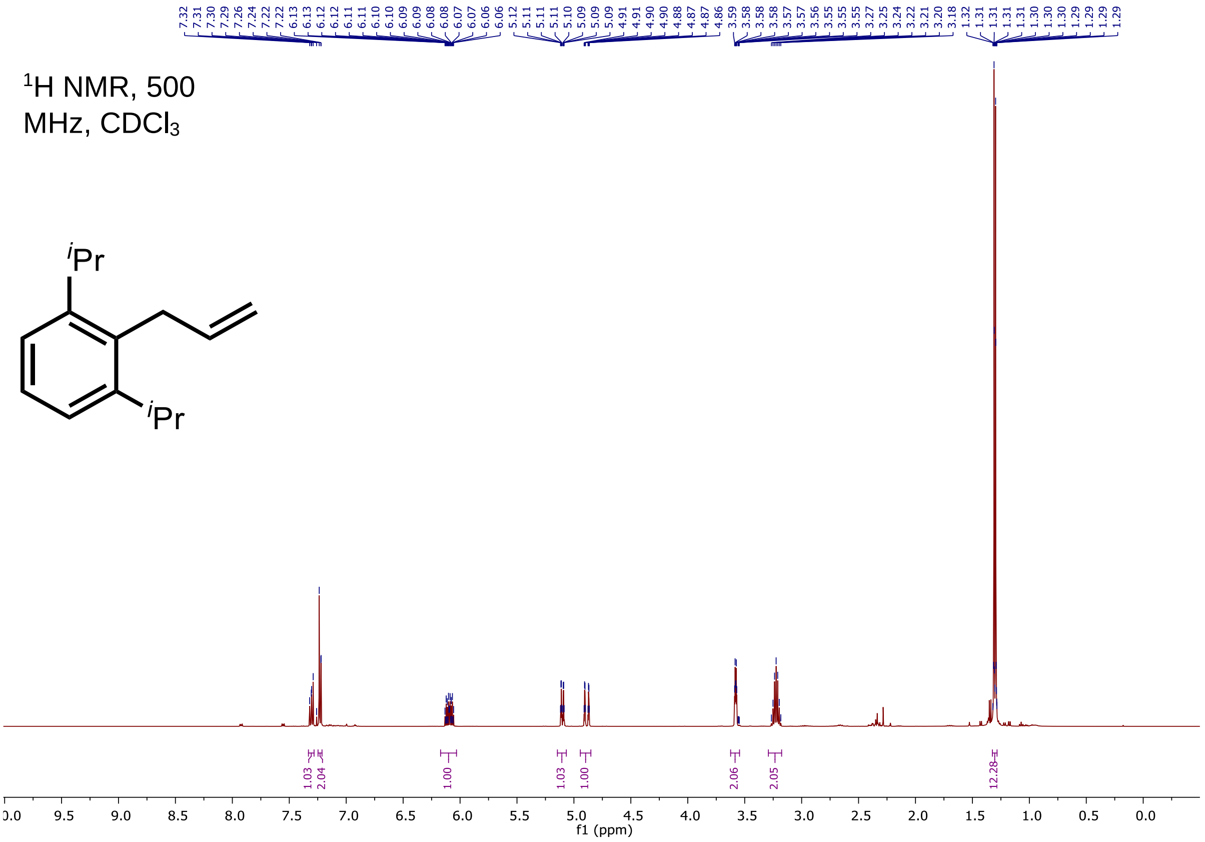

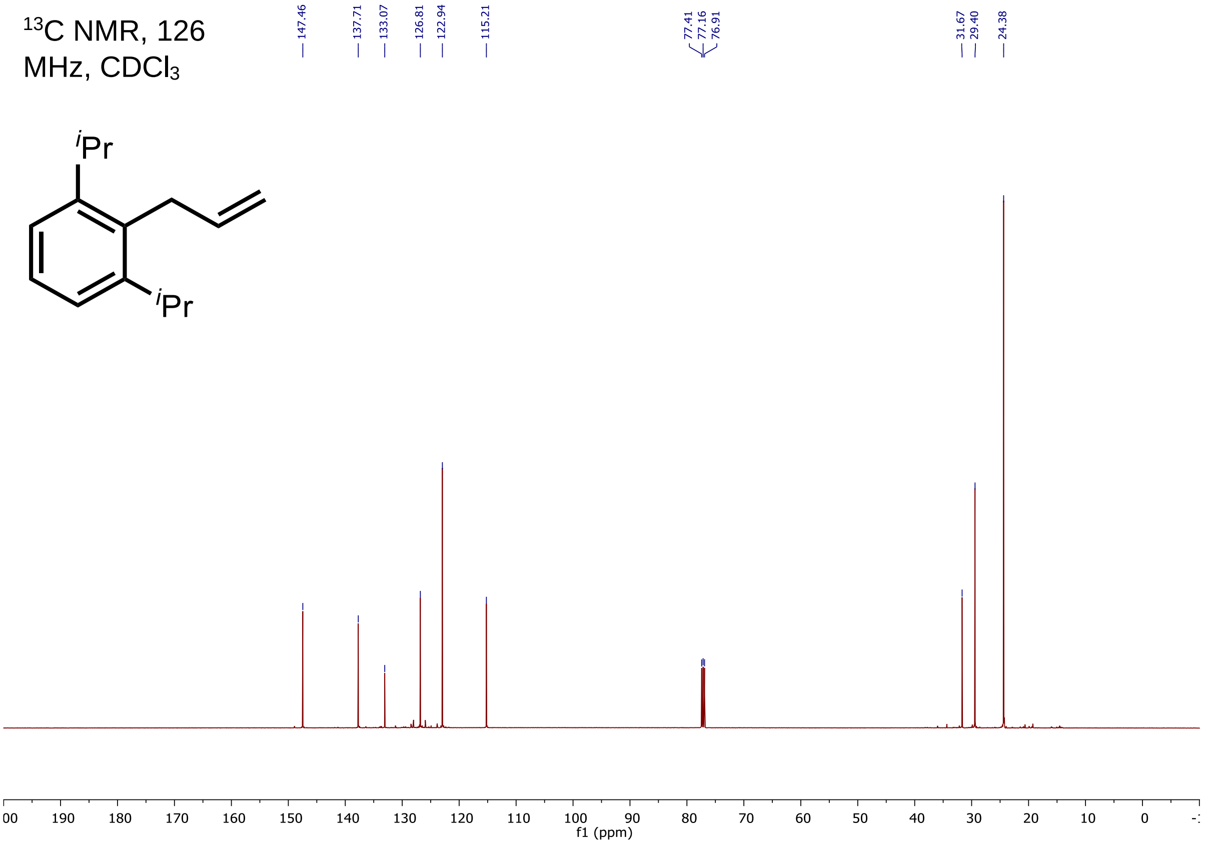

2-Allylnaphthalene[2]

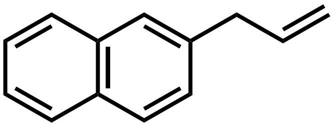

The title compound was prepared according to general procedure 1 using 2-naphthylboronic acid (2 mmol). Purification by flash silica chromatography (eluent = 10% EtOAc in hexanes) gave the title compound as colourless liquid (0.20 g, 64%); R_f: 0.70 (eluent = 10% EtOAc in hexanes); **^{1}H NMR (500 MHz, Chloroform-*d*)** δ 7.86 – 7.81 (m, 3H), 7.69 – 7.66 (m, 1H), 7.51 – 7.45 (m, 2H), 7.38 (dd, J = 8.4, 1.8 Hz, 1H), 6.10 (ddt, J = 16.8, 10.1, 6.7 Hz, 1H), 5.22 – 5.15 (m, 2H), 3.60 (dq, J = 6.7, 1.3 Hz, 2H); **^{13}C NMR (126 MHz, Chloroform-*d*)** δ 137.7, 137.5, 133.8, 132.3, 128.1, 127.8, 127.6, 127.5, 126.8, 126.1, 125.4, 116.2, 40.5; HRMS (EI$^+$) calculated $[C_{13}H_{12}]^+$ (M)$^+$: m/z 168.0933, found 168.0930.

^{1}H NMR, 500 MHz, $CDCl_3$

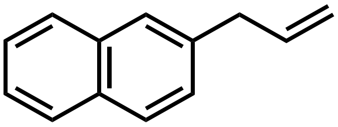

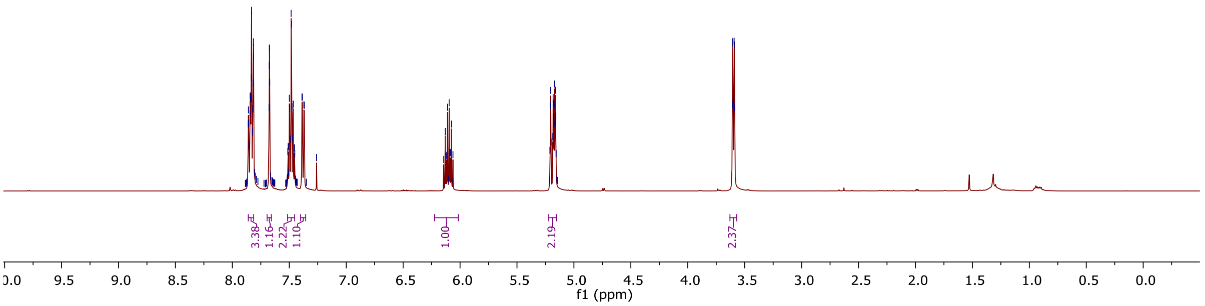

^{13}C NMR, 126 MHz, $CDCl_3$

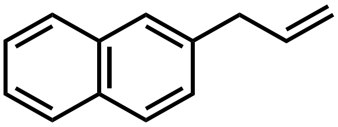

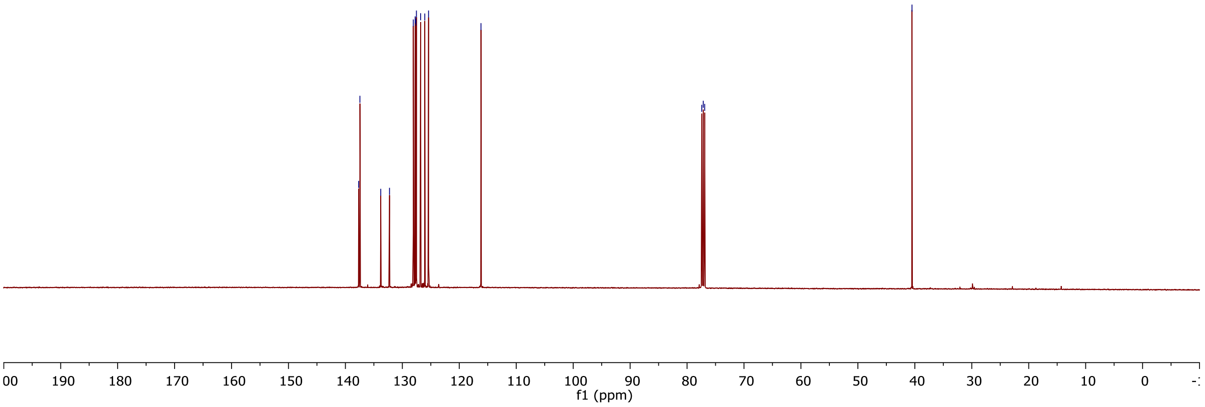

1-Allylnaphthalene[2]

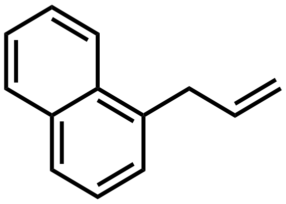

The title compound was prepared according to general procedure 1 using 1-naphthylboronic acid (4 mmol). Purification by flash silica chromatography (eluent = 10% EtOAc in hexanes) gave the title compound as colourless liquid (0.46 g, 90%); R_f: 0.75 (eluent = 10% EtOAc in hexanes); **^{1}H NMR (500 MHz, Chloroform-*d*)** δ 8.09 (dq, *J* = 8.4, 0.9 Hz, 1H), 7.93 – 7.89 (m, 1H), 7.79 (dt, *J* = 8.2, 1.0 Hz, 1H), 7.59 – 7.50 (m, 2H), 7.47 (dd, *J* = 8.2, 7.0 Hz, 1H), 7.40 (dt, *J* = 7.0, 1.0 Hz, 1H), 6.18 (ddt, *J* = 16.7, 10.3, 6.3 Hz, 1H), 5.26 – 5.07 (m, 2H), 3.90 (dt, *J* = 6.4, 1.7 Hz, 2H); **^{13}C NMR (126 MHz, Chloroform-*d*)** δ 137.1, 136.2, 133.9, 132.1, 128.8, 127.1, 126.4, 125.9, 125.8, 125.7, 124.2, 116.3, 37.4; HRMS (EI$^+$) calculated $[C_{13}H_{12}]^+$ $(M)^+$: m/z 168.0933, found 168.0929.

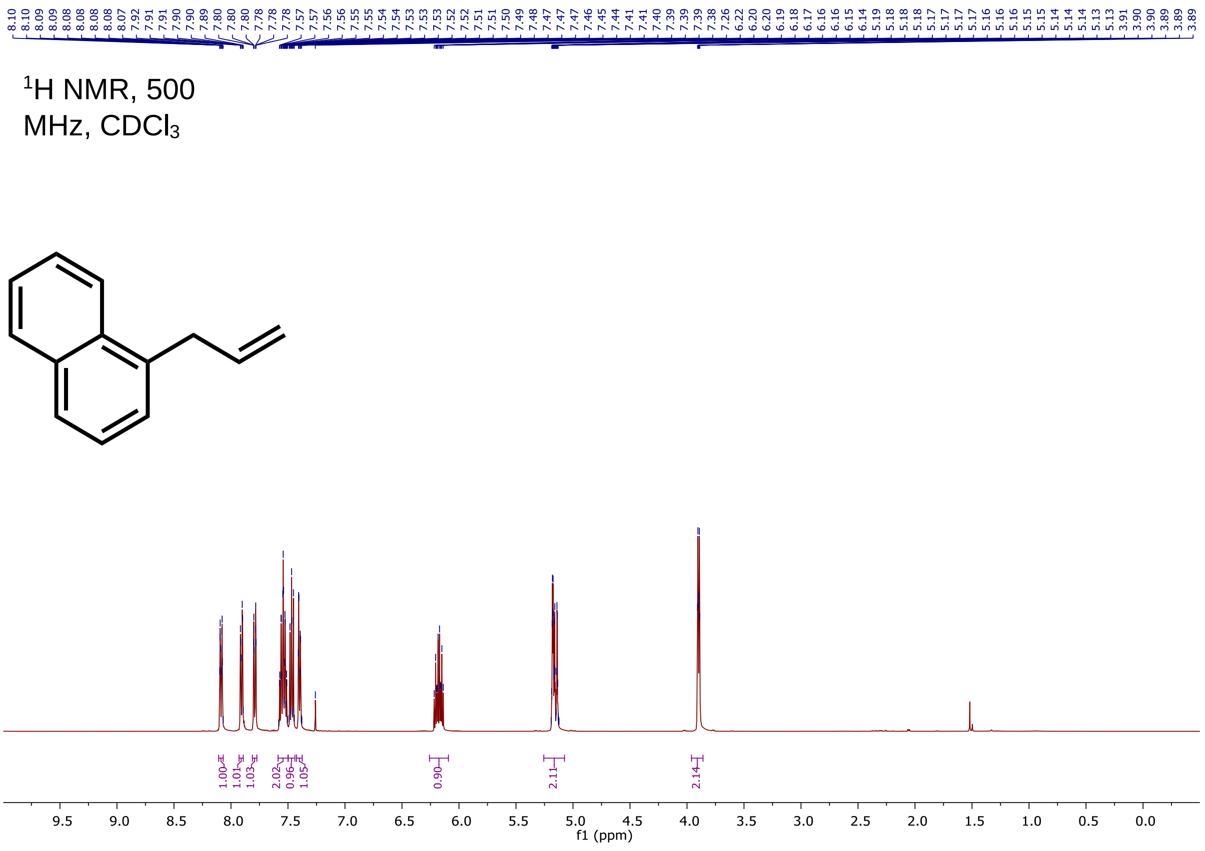

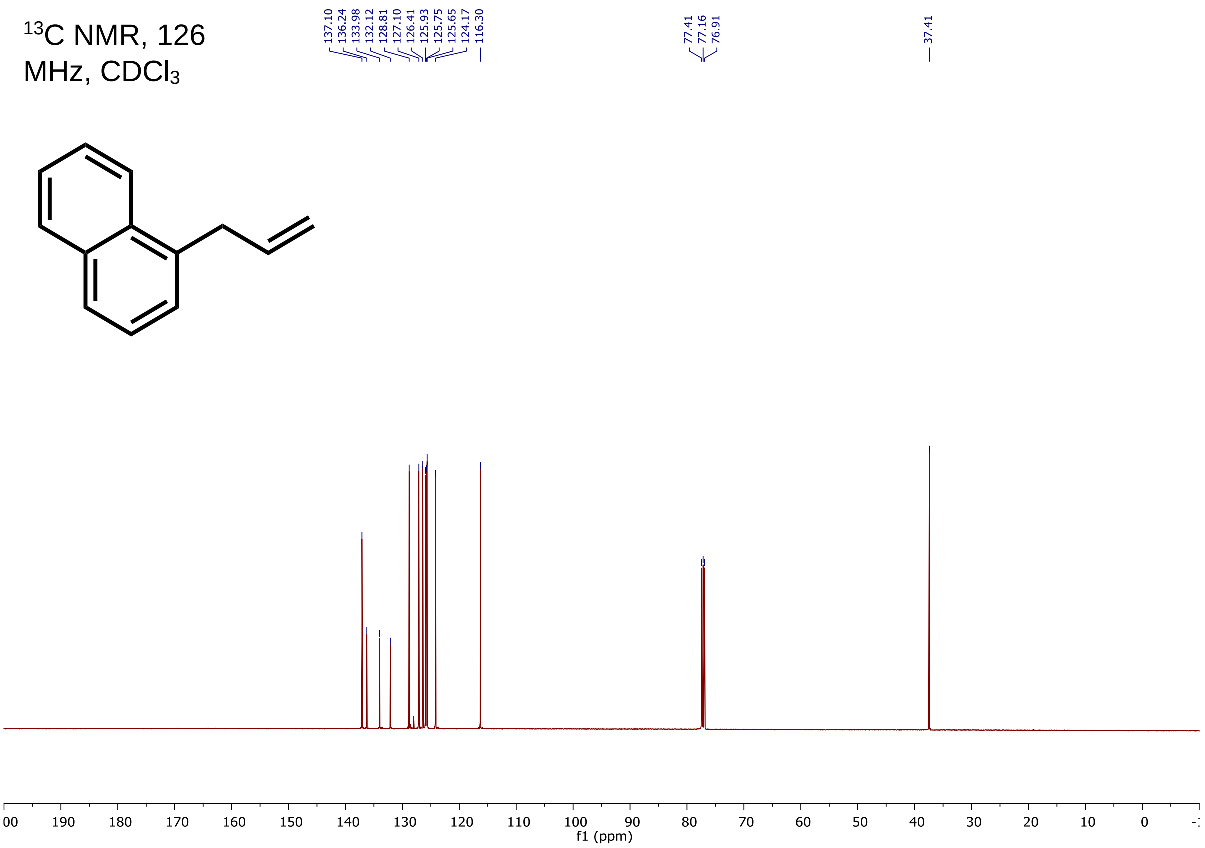

2-Allyl-1,1’-biphenyl[12]

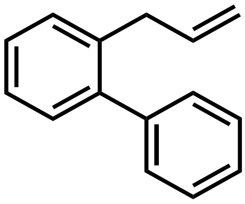

The title compound was prepared according to general procedure 1 using 2-biphenylboronic acid (4 mmol). Purification by flash silica chromatography (eluent = 10% EtOAc in hexanes) gave the title compound as colourless liquid (0.56 g, 97%); R_f: 0.78 (eluent = 10% EtOAc in hexanes); **^{1}H NMR (500 MHz, Chloroform-*d*)** δ 7.51 – 7.44 (m, 2H), 7.46 – 7.37 (m, 5H), 7.37 – 7.31 (m, 2H), 6.04 – 5.92 (m, 1H), 5.10 (ddt, *J* = 10.1, 2.0, 1.4 Hz, 1H), 5.01 (dq, *J* = 17.0, 1.7 Hz, 1H), 3.43 (dt, *J* = 6.4, 1.6 Hz, 2H); **^{13}C NMR (126 MHz, Chloroform-*d*)** δ 142.1, 141.8, 137.9, 137.3, 130.2, 129.8, 129.4, 128.1, 127.5, 127.0, 126.2, 115.9, 37.6; HRMS (EI$^+$) calculated $[C_{15}H_{14}]^+$ $(M)^+$: m/z 194.1090, found 194.1087.

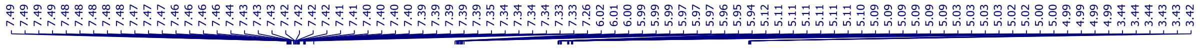

^{1}H NMR, 500 MHz, $CDCl_3$

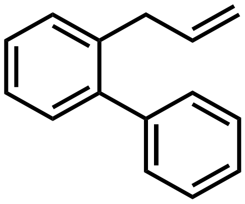

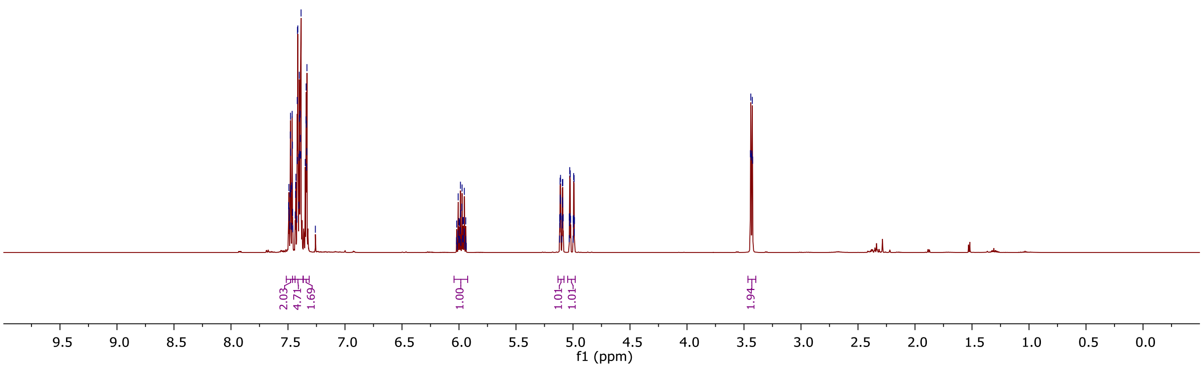

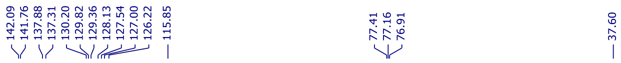

^{13}C NMR, 126 MHz, $CDCl_3$

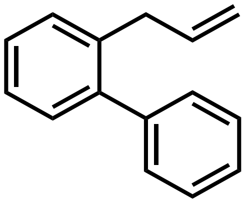

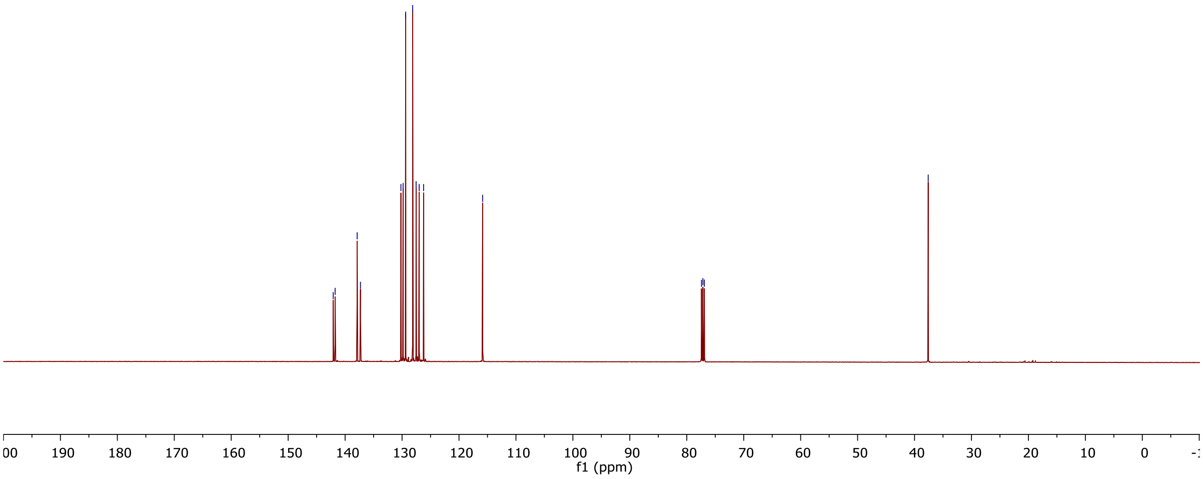

1-(4-Allylphenyl)ethan-1-one[13]

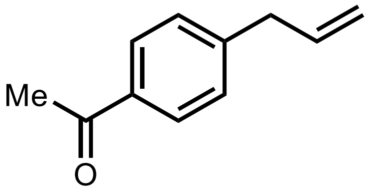

The title compound was prepared according to general procedure 1 using 4-acethoxyphenylboronic acid (2.78 mmol). Purification by flash silica chromatography (eluent = 10% EtOAc in PE) gave the title compound as colourless liquid (0.20 g, 62%); R_f: 0.59 (eluent = 10% EtOAc in PE); **^{1}H NMR (300 MHz, Chloroform-*d*)** δ 7.23 – 7.17 (m, 2H), 7.04 – 6.98 (m, 2H), 5.96 (ddt, *J* = 16.1, 10.8, 6.7 Hz, 1H), 5.14 – 5.05 (m, 2H), 3.39 (dt, *J* = 6.8, 1.4 Hz, 2H), 2.29 (s, 3H); **^{13}C NMR (75 MHz, Chloroform-*d*)** δ 169.8, 149.1, 137.7, 137.2, 129.6, 121.5, 116.2, 39.7, 21.3.

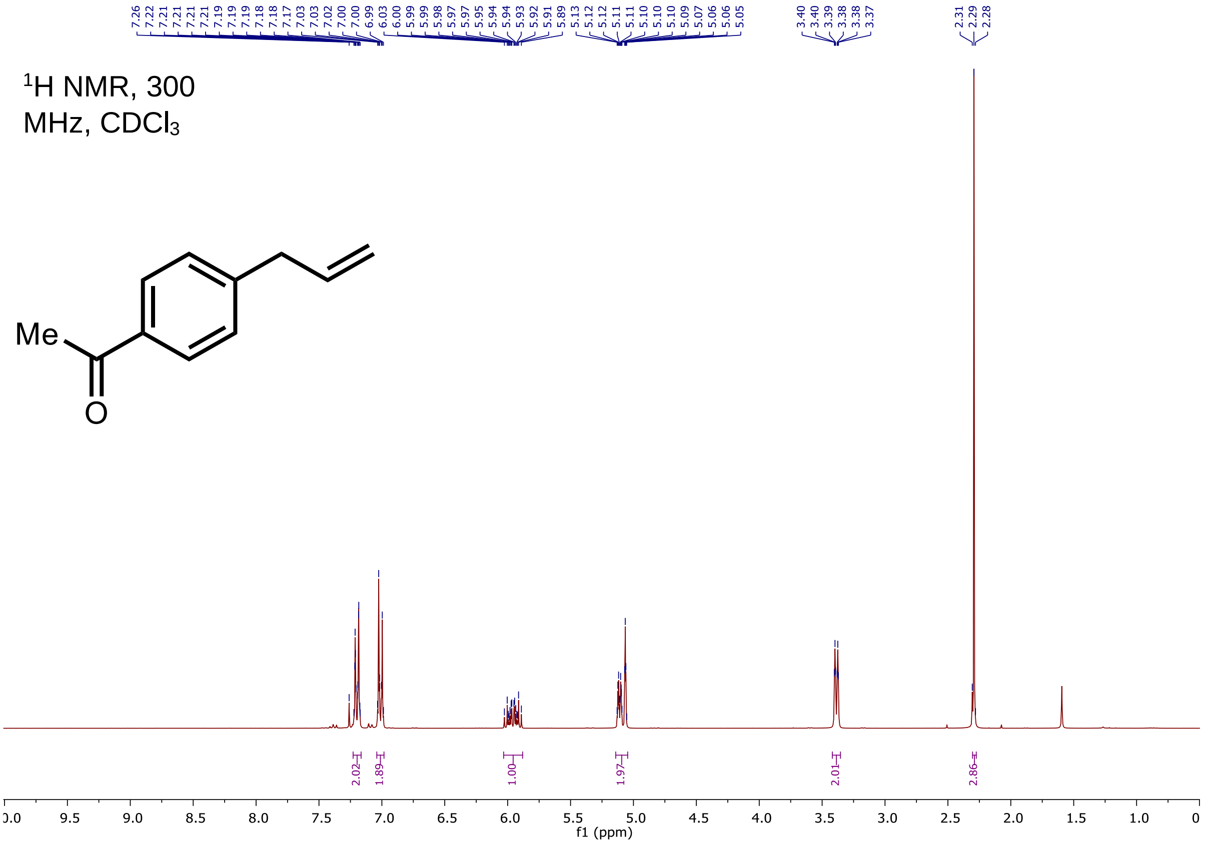

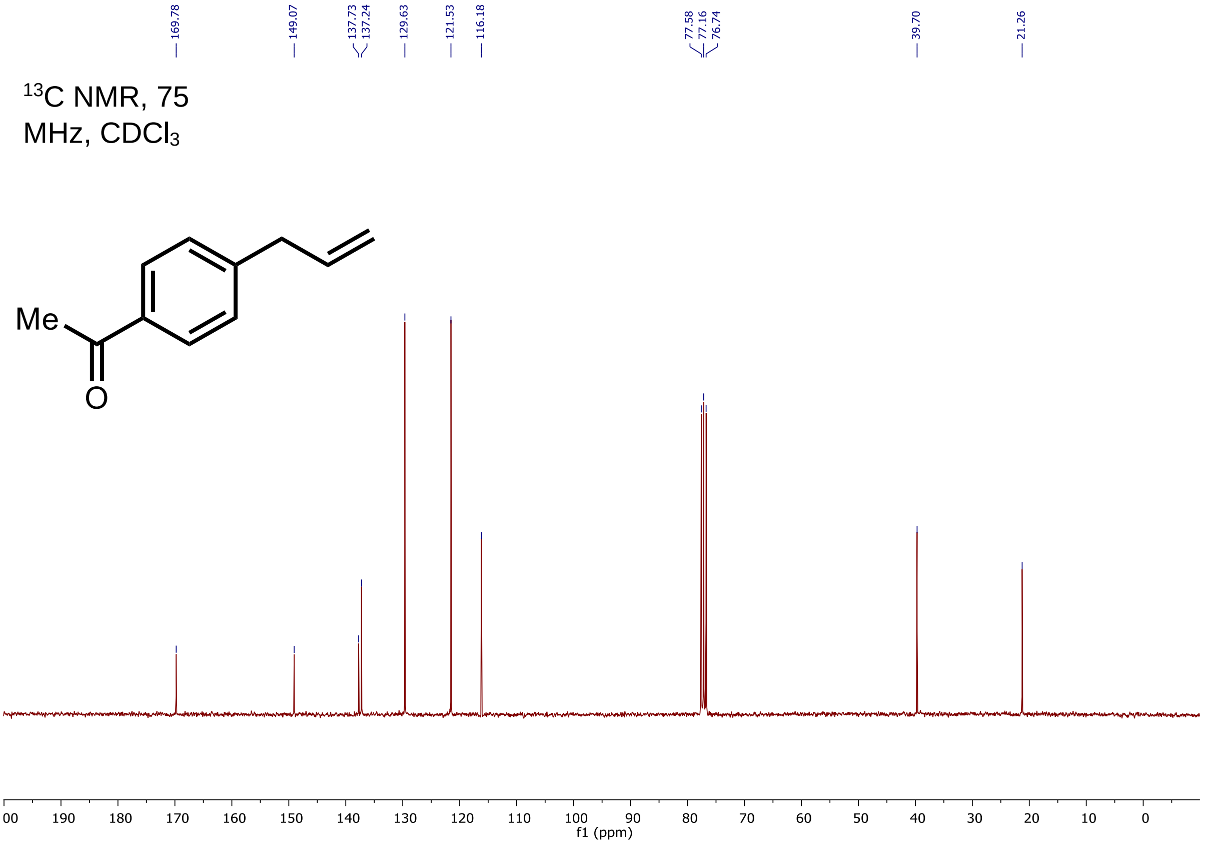

tert-butyl-4-allylbenzoate[14]

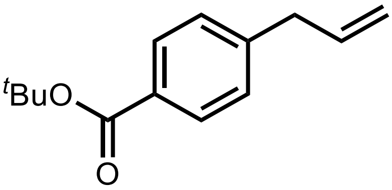

The title compound was prepared according to general procedure 1 using 4-*tert*-butoxyphenylboronic acid (4 mmol). Purification by flash silica chromatography (eluent = 20% EtOAc in PE) gave the title compound as colourless liquid (0.43 g, 65%); R_f: 0.76 (eluent = 20% EtOAc in PE); **^{1}H NMR (300 MHz, Chloroform-*d*)** δ 7.97 – 7.86 (m, 2H), 7.25 – 7.20 (m, 2H), 6.07 – 5.85 (m, 1H), 5.18 – 5.01 (m, 2H), 3.43 (dt, *J* = 6.8, 1.5 Hz, 2H), 1.59 (s, 9H); **^{13}C NMR (75 MHz, Chloroform-*d*)** δ 165.9, 145.0, 136.7, 130.1, 129.7, 128.6, 116.6, 80.9, 40.3, 28.4; HRMS (CI$^+$) calculated $[C_{14}H_{19}O_2]^+$ (M+H)$^+$: m/z 219.1379, found 219.1380.

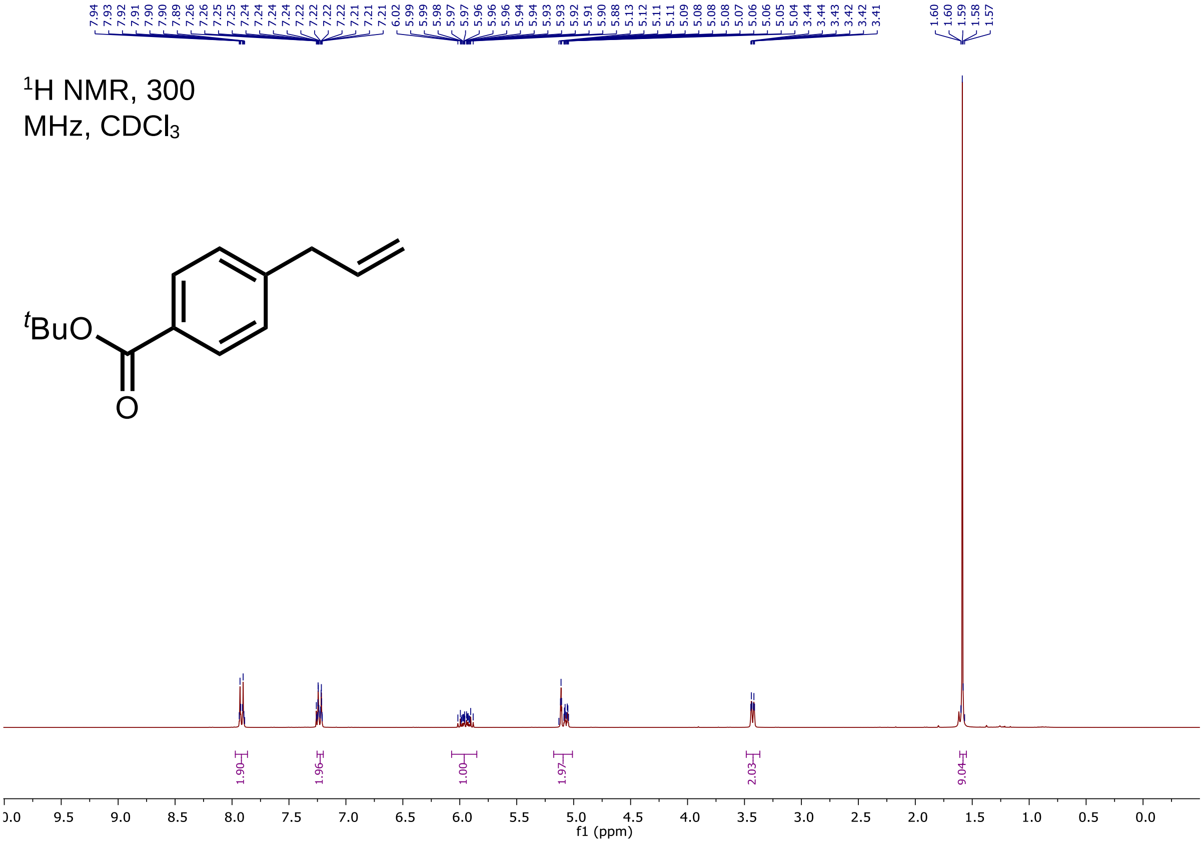

^{1}H NMR, 300 MHz, $CDCl_3$
^{t}BuO
O
f1 (ppm)

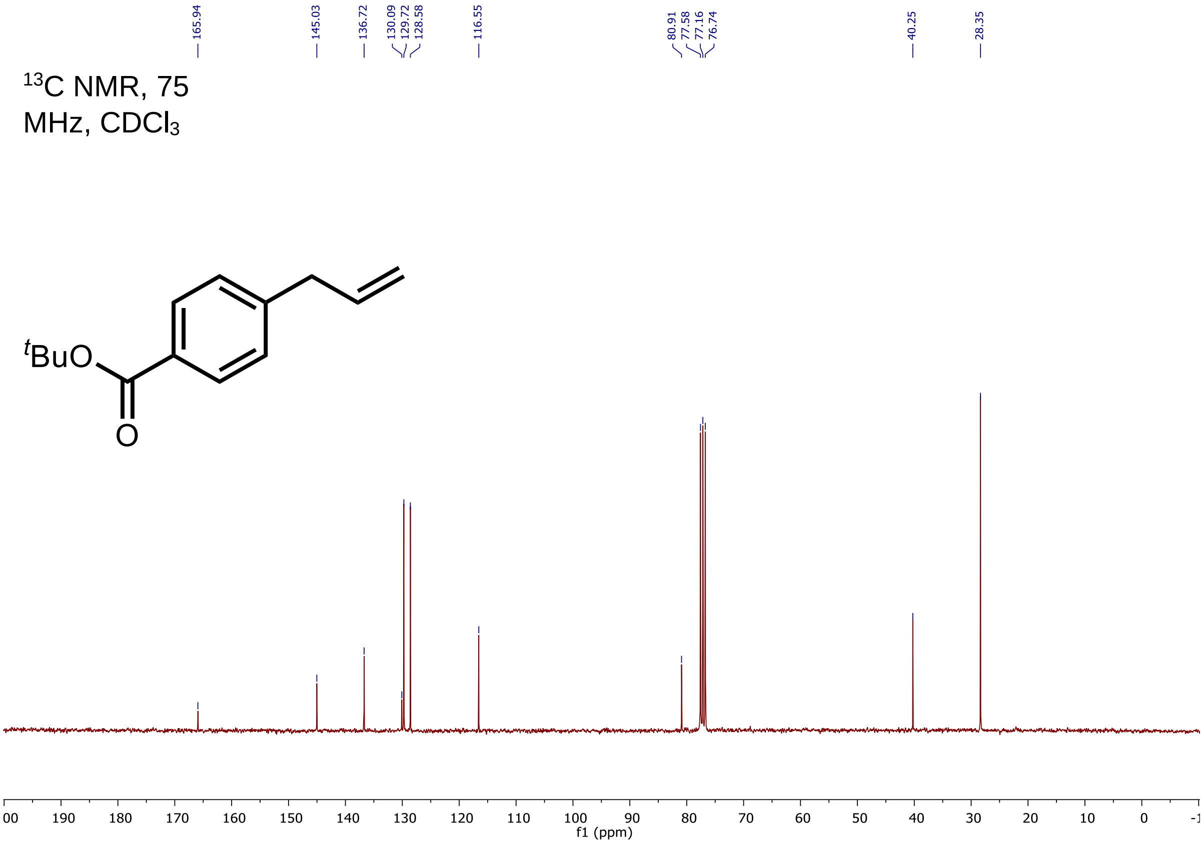

^{13}C NMR, 75 MHz, $CDCl_3$
^{t}BuO
O
165.94
145.03
136.72
130.09
129.72
128.58
116.55
80.91
77.58
77.16
76.74
40.25
28.35
f1 (ppm)

1-Allyl-3-nitrobenzene[15]

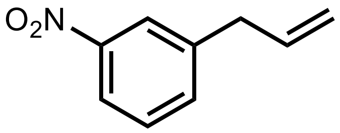

The title compound was prepared according to general procedure 1 using 3-nitrophenylboronic acid (2 mmol). Purification by flash silica chromatography (eluent = 5% EtOAc in hexanes) gave the title compound as pale yellow liquid (0.12 g, 47%); R_f: 0.51 (eluent = 5% EtOAc in hexanes); **^{1}H NMR (400 MHz, Chloroform-*d*)** δ 8.12 – 8.03 (m, 2H), 7.58 – 7.41 (m, 2H), 5.96 (ddt, *J* = 16.8, 10.1, 6.7, 1H), 5.20 – 5.08 (m, 2H), 3.53 – 3.46 (m, 2H); **^{13}C NMR (101 MHz, Chloroform-*d*)** δ 148.5, 142.2, 135.8, 134.9, 129.4, 123.6, 121.4, 117.5, 39.8; HRMS (EI$^+$) calculated $[C_9H_9O_2N]^+$ (M)$^+$: m/z 163.0627, found 163.0627.

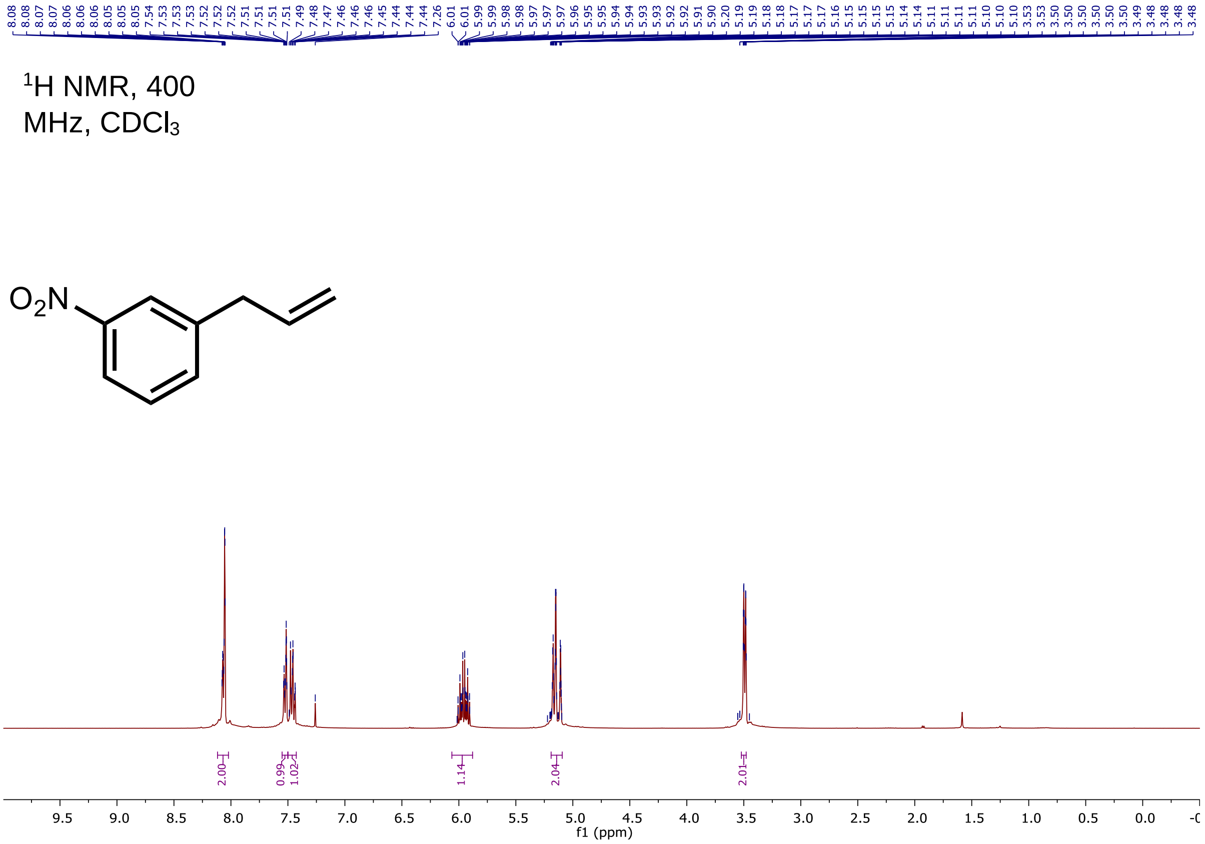

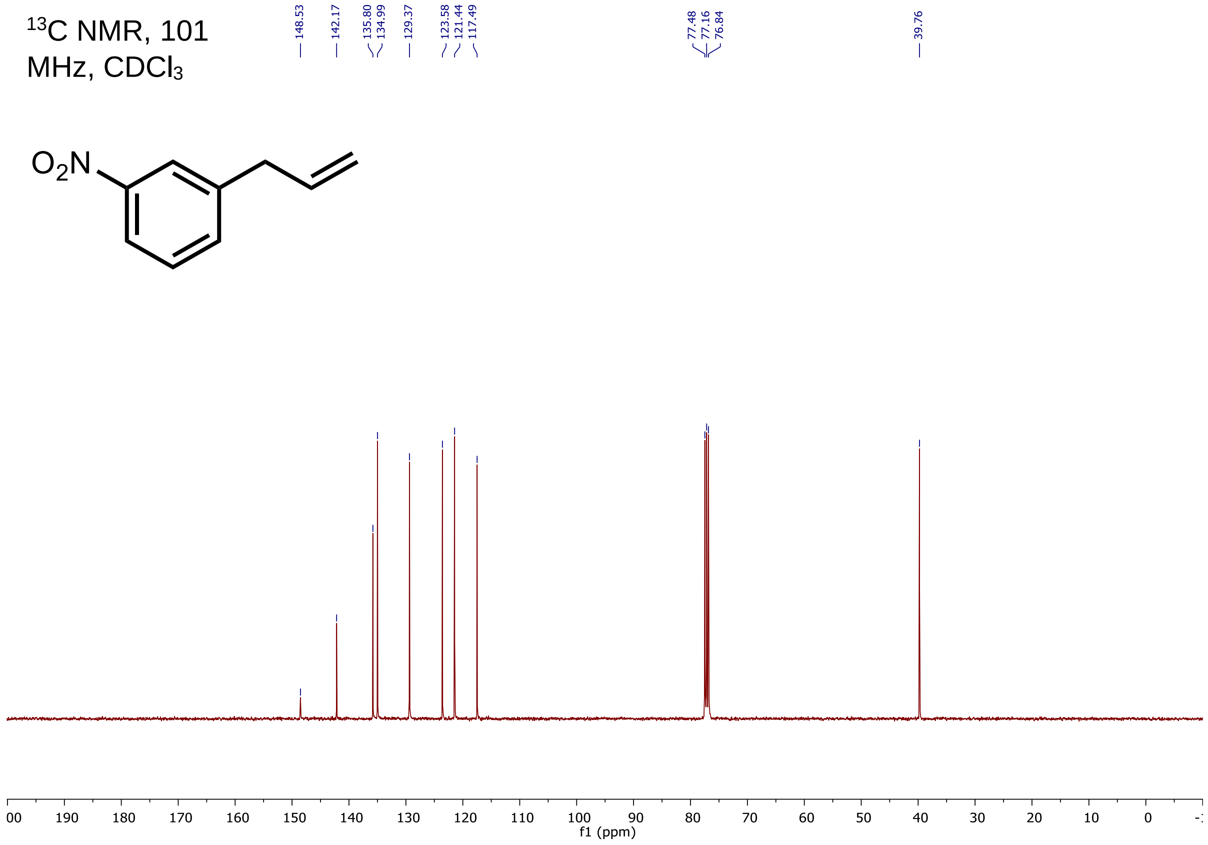

1-Allyl-4-nitrobenzene[16]

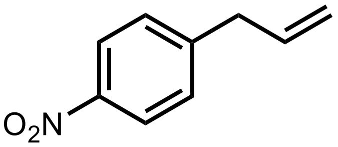

The title compound was prepared according to general procedure 1 using 4-nitrophenylboronic acid (4 mmol). Purification by flash silica chromatography (eluent = 20% EtOAc in hexanes) gave the title compound as pale yellow liquid (0.17 g, 35%); R_f: 0.74 (eluent = 20% EtOAc in hexanes); **^{1}H NMR (500 MHz, Chloroform-*d*)** δ 8.21 – 8.12 (m, 2H), 7.40 – 7.31 (m, 2H), 5.94 (ddt, *J* = 16.8, 10.1, 6.7 Hz, 1H), 5.23 – 5.07 (m, 2H), 3.49 (dt, *J* = 6.8, 1.5 Hz, 2H); **^{13}C NMR (126 MHz, Chloroform-*d*)** δ 147.9, 146.7, 135.6, 129.5, 123.8, 117.6, 40.0; HRMS (ASAP) calculated $[C_9H_{10}O_2N]^+$ $(M+H)^+$: m/z 164.0712, found 164.0708.

^{1}H NMR, 500 MHz, $CDCl_3$

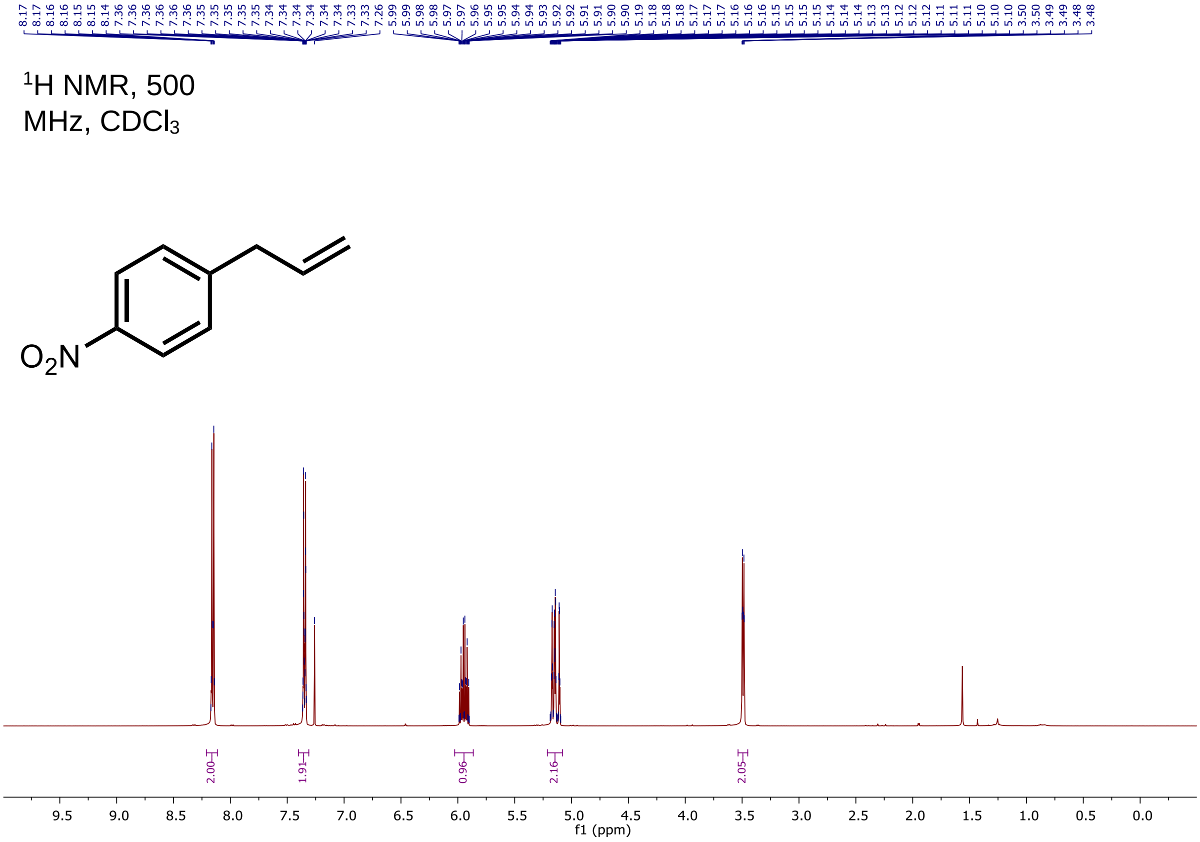

^{13}C NMR, 126 MHz, $CDCl_3$

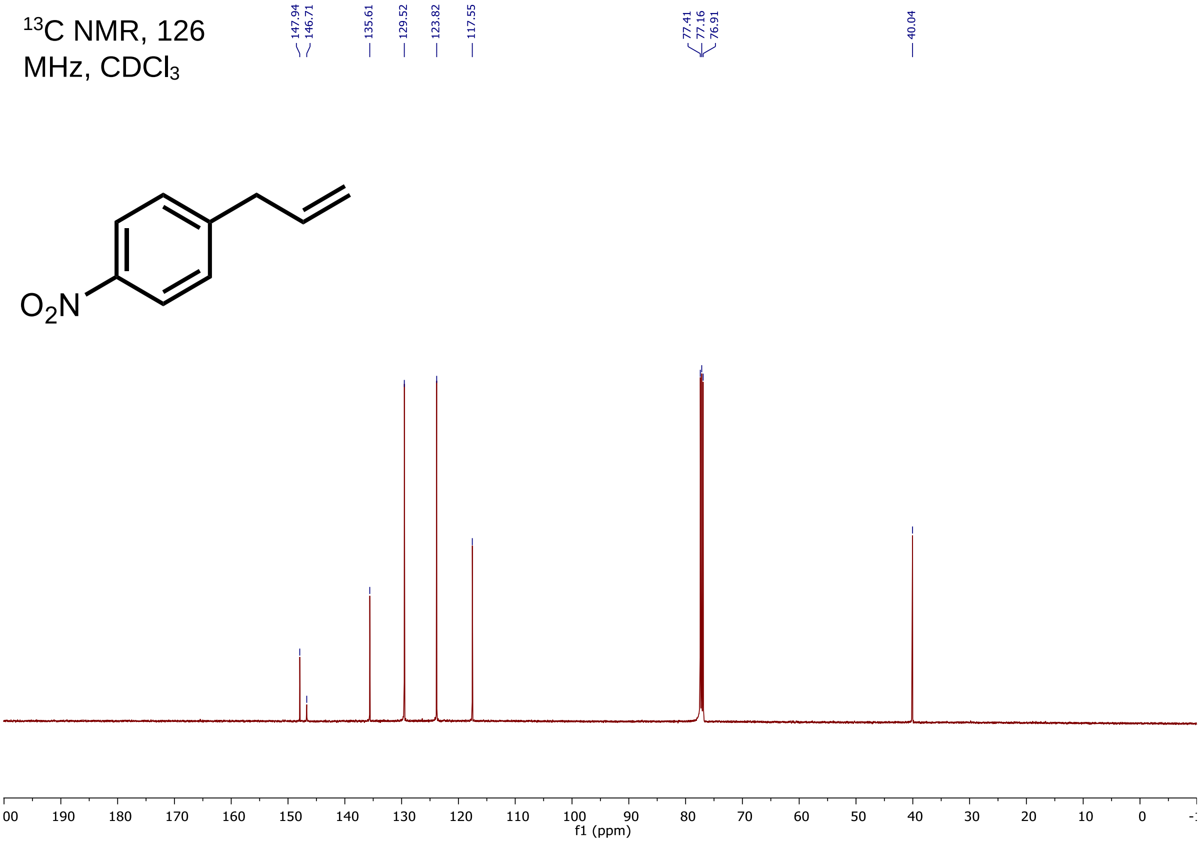

((6-Allylnaphthalen-2-yl)oxy)(*tert*-butyl)dimethylsilane (40)[17]

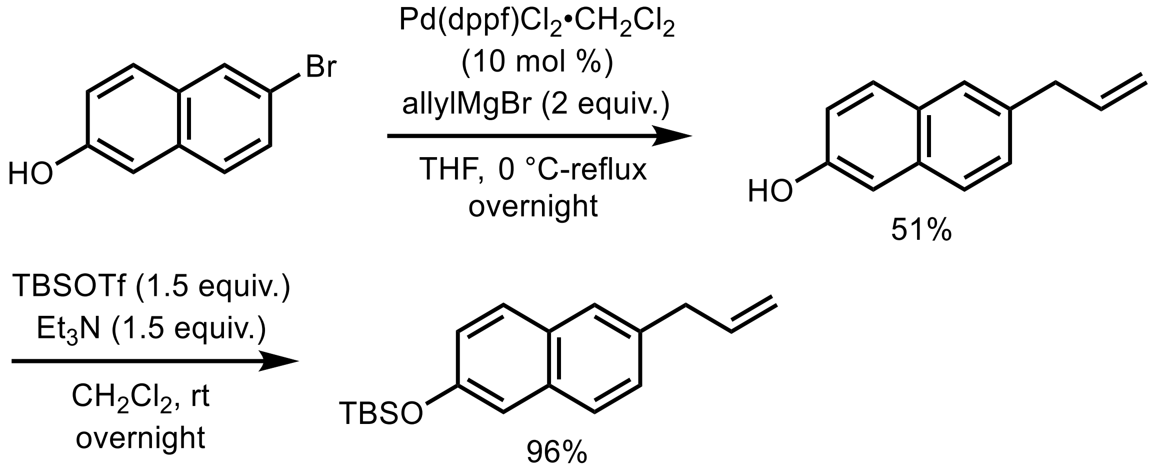

6-Bromonaphthalen-2-ol (1.12 g, 5 mmol) and $Pd(dppf)Cl_2.CH_2Cl_2$ (0.41 g, 10 mol %) were added into dry THF (30 mL). Allylmagnesium bromide (10 mL, 10 mmol) was added dropwise to the mixture at 0 ˚C. The solution was refluxed for overnight. The reaction was quenched by sat. NH_4Cl then extracted with EtOAc, washed with brine (3 x 10 mL), dried over $MgSO_4$, and the solvent was removed *in vacuo*. The crude compound was purified by flash silica chromatography (eluent = EtOAc:PE 1:1) to give 6-allylnaphthalene-2-ol as off-white solid (0.47 g, 51%); mp 89–92 ˚C (lit. 90 ˚C)[17]; R_f = 0.80 (eluent = EtOAc:PE 1:1). **^{1}H NMR (300 MHz, Chloroform-*d*)** δ 7.69 (dq, *J* = 8.8, 0.6 Hz, 1H), 7.62 (d, *J* = 8.5 Hz, 1H), 7.56 (d, *J* = 1.5 Hz, 1H), 7.29 (dd, *J* = 8.4, 1.8 Hz, 1H), 7.14 – 7.05 (m, 2H), 6.04 (ddt, *J* = 16.8, 10.1, 6.7 Hz, 1H), 5.19 – 5.06 (m, 2H), 4.90 (s, 1H), 3.54 – 3.48 (m, 2H). **^{13}C NMR (75 MHz, Chloroform-*d*)** δ 153.0, 137.6, 135.4, 133.3, 129.5, 129.2, 128.2, 126.7, 126.6, 117.9, 116.1, 109.5, 40.3; HRMS (CI) calculated $[C_{13}H_{12}O]^+$ $(M)^+$: m/z 184.0882, found 184.0882.

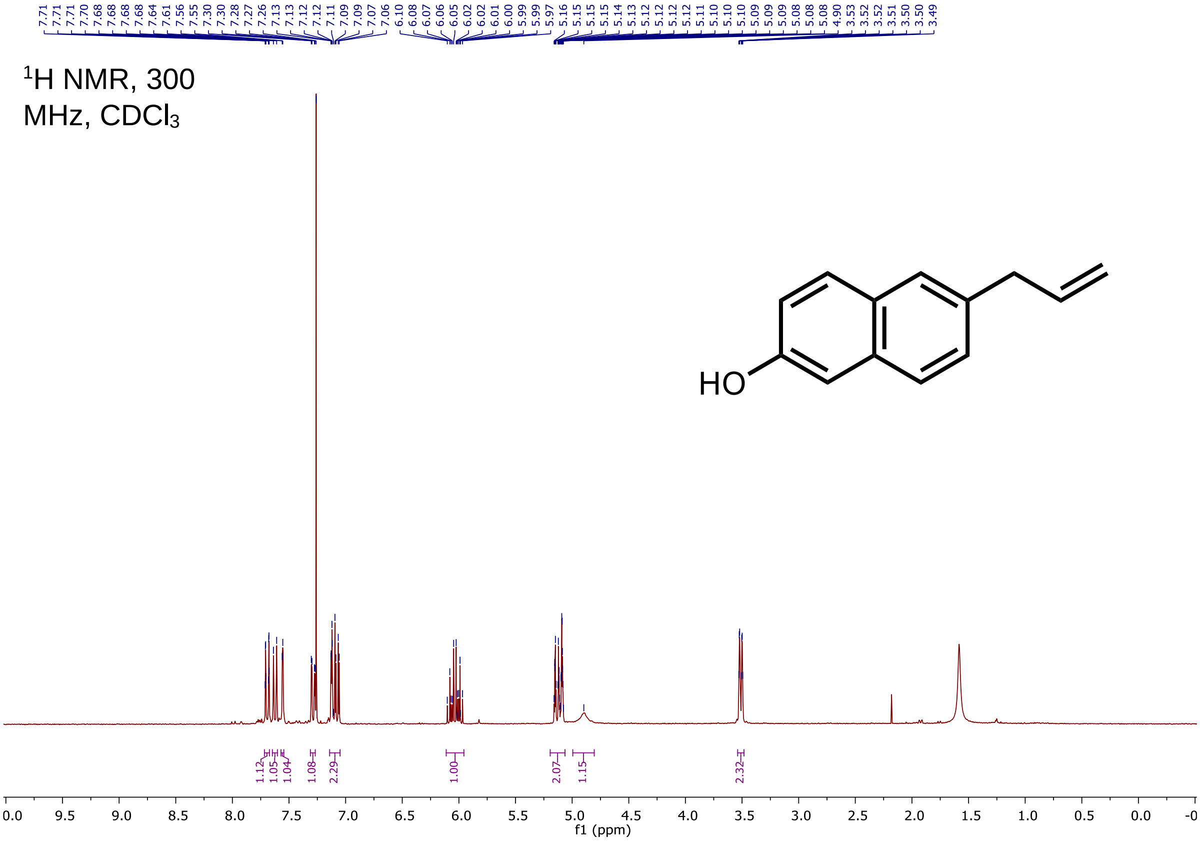

1H NMR, 300 MHz, CDCl3
HO
f1 (ppm)

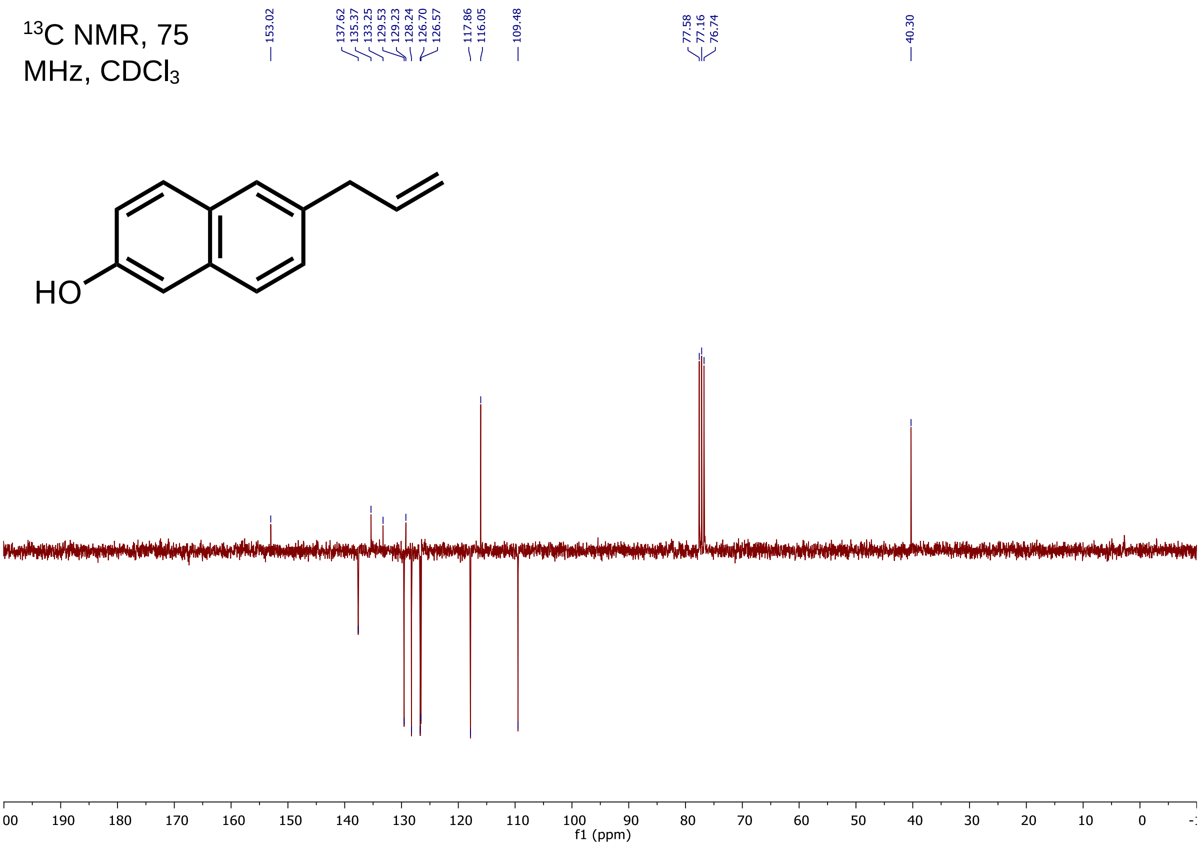

13C NMR, 75 MHz, CDCl3
153.02
137.62
135.37
133.25
129.53
129.23
128.24
126.70
126.57
117.86
116.05
109.48
77.58
77.16
76.74
40.30
HO
f1 (ppm)

6-allylnaphthalene-2-ol (0.37 g, 2 mmol) and Et_3N (0.42 mL, 3 mmol) were added to dry CH_2Cl_2 (4 mL). TBSOTf (0.7 mL, 3 mmol) was added to the mixture dropwise at rt. The solution was stirred at rt for overnight. The solution was quenched by sat. NH_4Cl then extracted with EtOAc, washed with brine (1 x 10 mL), dried over $MgSO_4$, and the solvent was removed *in vacuo*. The crude was purified by deactivated silica chromatography (eluent = 100% PE) to give the title compound as colourless liquid (0.57 g, 96%); R_f = 0.61 (eluent = 100% PE); ν_{max} / cm^{-1} (film) 2953, 2928, 2887, 2857, 1638, 1603, 1477, 1362, 1258, 1234, 1153, 1121, 974; **^{1}H NMR (300 MHz, Chloroform-*d*)** δ 7.70 – 7.62 (m, 2H), 7.56 (dd, *J* = 1.8, 0.9 Hz, 1H), 7.28 (dd, *J* = 8.4, 1.8 Hz, 1H), 7.19 – 7.16 (m, 1H), 7.06 (dd, *J* = 8.7, 2.4 Hz, 1H), 6.04 (ddt, *J* = 16.8, 10.0, 6.7 Hz, 1H), 5.20 – 5.06 (m, 2H), 3.56 – 3.48 (m, 2H), 1.02 (s, 9H), 0.24 (s, 6H). **^{13}C NMR (75 MHz, Chloroform-*d*)** δ 153.2, 137.7, 135.5, 133.3, 129.6, 128.9, 127.8, 126.9, 126.6, 122.3, 116.0, 114.9, 40.4, 25.9, -4.2; HRMS (CI) calculated $[C_{19}H_{26}OSi]^+$ $(M)^+$: m/z 298.1747, found 298.1748.

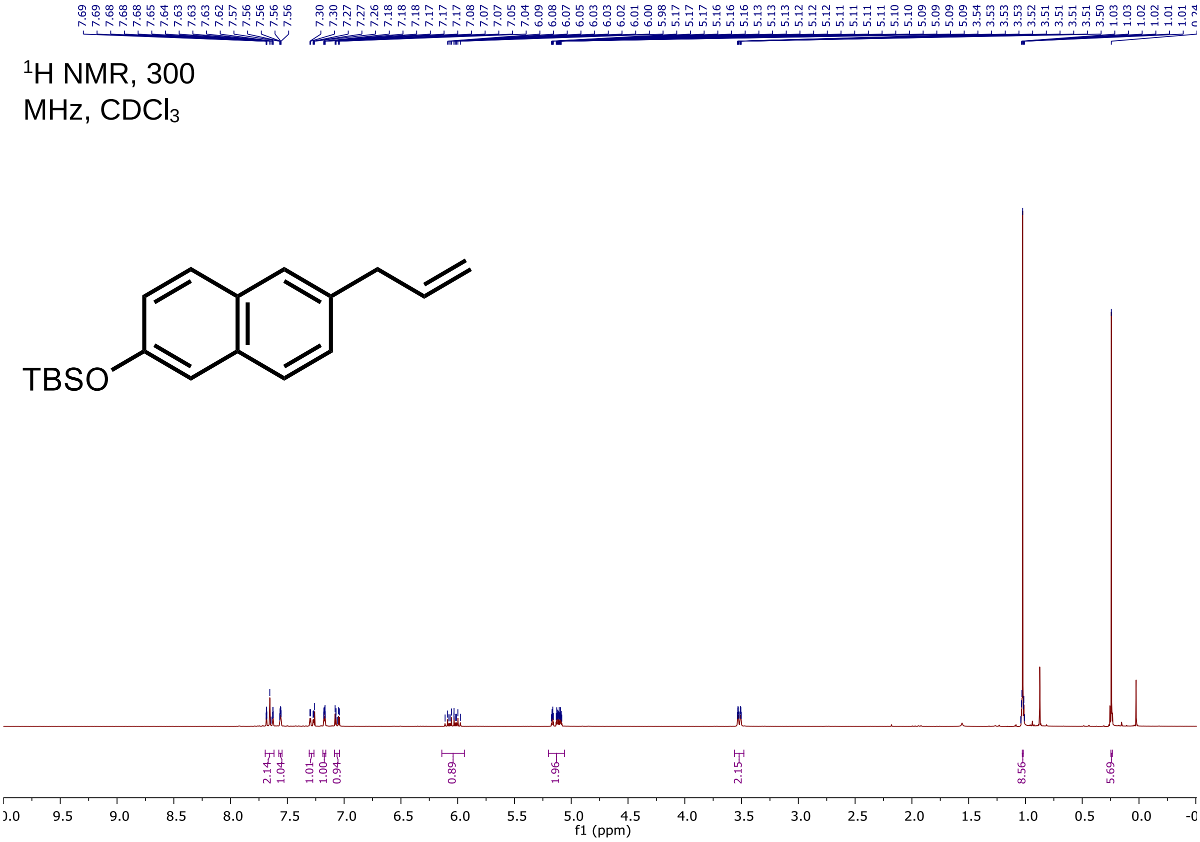

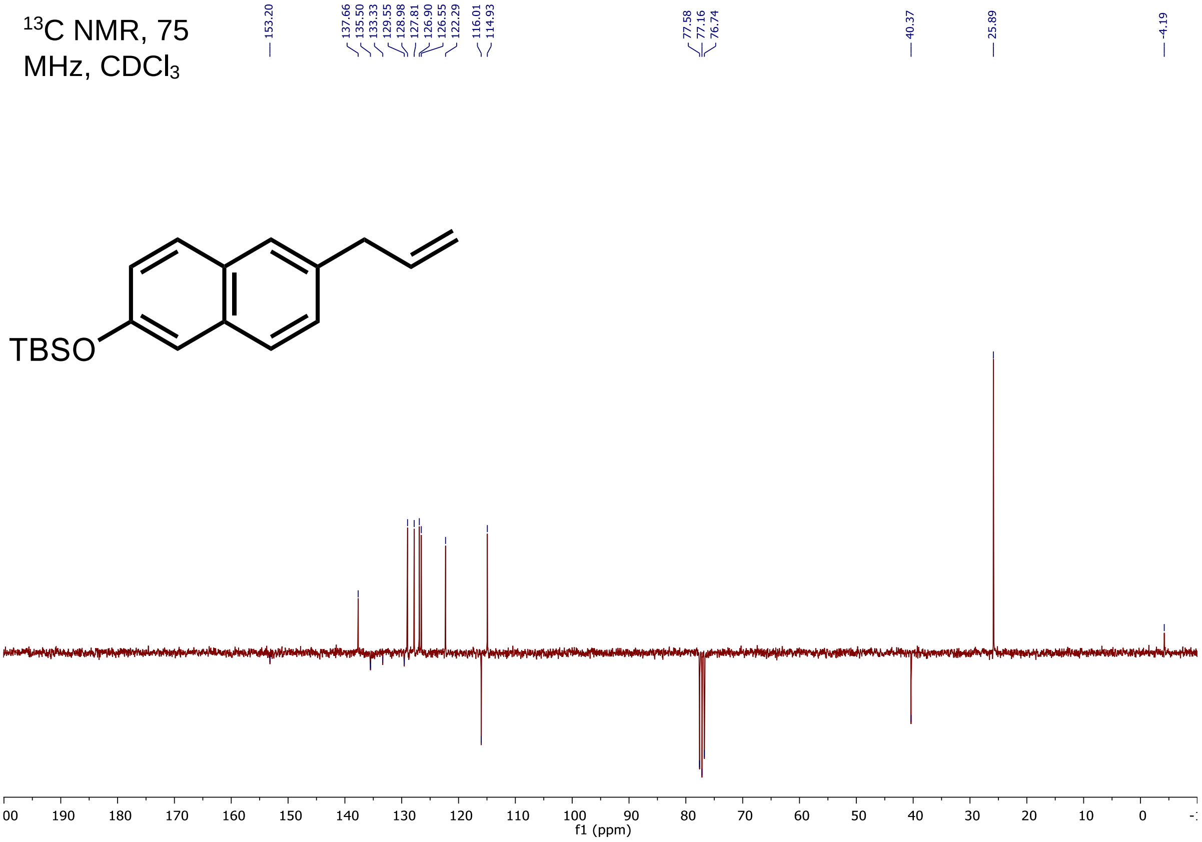

2.1.2. General procedure 2 [15]

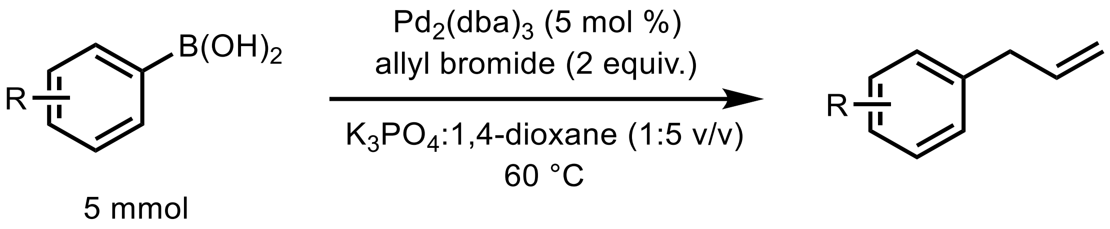

Under nitrogen, to an oven dried 100 ml three-necked round bottomed flask with a stirrer bar was added boronic acid (5 mmol) and Pd_2dba_3 (5 mol %). An aq. 3 M solution of K_3PO_4 (1:1 ratio for every mmol of boronic acid used) was added along with allyl bromide (2 equiv.) and dioxane (5:1 v/v ratio to K_3PO_4) to the mixture. The reaction was stirred at 60 ˚C and monitored by TLC. The reaction mixture was allowed to cool to rt and quenched with 1 M NaOH, extracted with Et_2O (3 x 10 mL). The combined organic layer was dried over $MgSO_4$, filtered, and concentrated *in vacuo*.

4-Allyl-*N,N*-dimethylaniline[2]

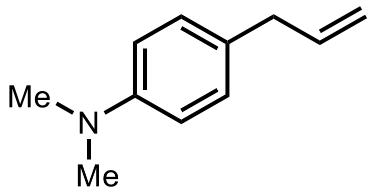

The title compound was prepared according to general procedure 2 using 4-(dimethylamino)phenylboronic acid (1.5 mmol). Purification by flash silica chromatography (eluent = 10% EtOAc in hexanes) gave the title compound as a pale yellow oil (59 mg, 37%); R_f: 0.54 (eluent = 10% EtOAc in hexanes); **^{1}H NMR (500 MHz, Chloroform-d)** δ 7.11 – 7.03 (m, 2H), 6.75 – 6.69 (m, 2H), 5.98 – 5.88 (m, 1H), 5.12 – 5.00 (m, 2H), 3.30 (dddd, *J* = 6.7, 2.1, 1.4, 0.6 Hz, 2H), 2.92 (s, 6H). **^{13}C NMR (126 MHz, Chloroform-d)** δ 149.3, 138.5, 129.3, 128.3, 115.1, 113.2, 41.1, 39.4. HRMS (EI$^+$) calculated $[C_{11}H_{15}N]^+$ (M)$^+$: m/z 161.1199, found 161.1196.

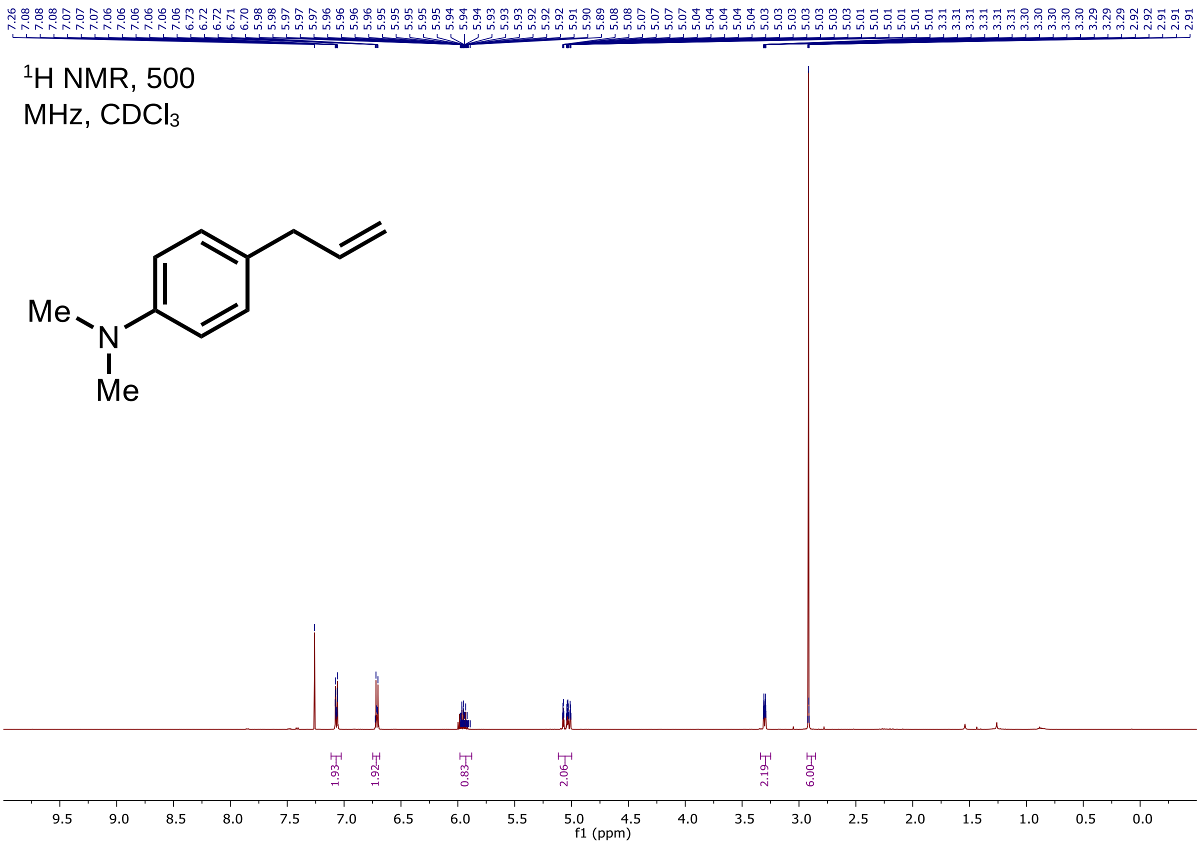

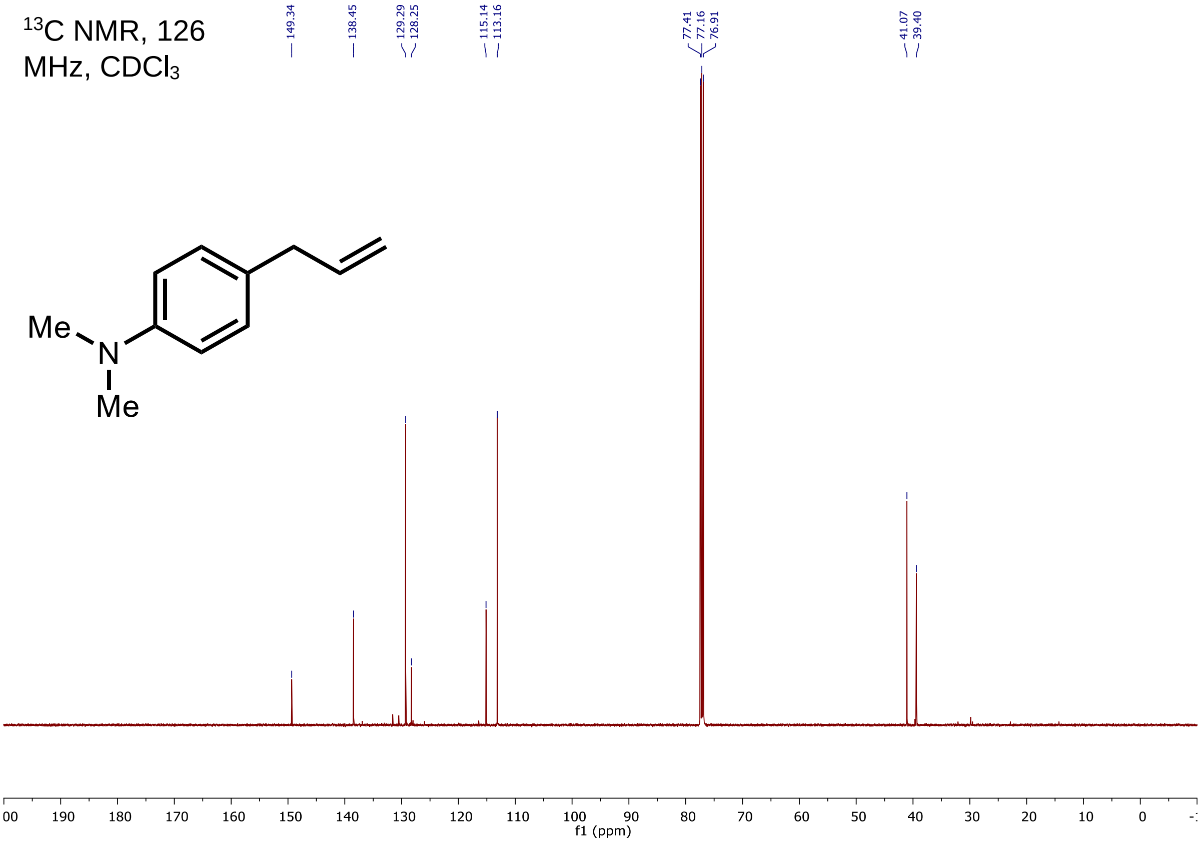

1-Allyl-4-(*tert*-butyl)benzene[2]

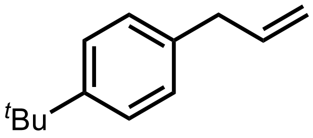

The title compound was prepared according to general procedure 2 using (4-(*tert*-butyl)phenyl)boronic acid (5 mmol). Purification by flash silica chromatography (eluent = 10% EtOAc in hexanes) gave the title compound as a pale yellow oil (0.52 g, 60%); R_f: 0.85 (eluent = 10% EtOAc in hexanes); **^{1}H NMR (500 MHz, Chloroform-d)** δ 7.33 (d, *J* = 8.3 Hz, 2H), 7.17 – 7.11 (m, 2H), 5.99 (ddt, *J* = 16.9, 10.0, 6.8 Hz, 1H), 5.13 – 5.04 (m, 2H), 3.41 – 3.35 (m, 2H), 1.32 (d, *J* = 0.9 Hz, 9H). **^{13}C NMR (126 MHz, Chloroform-d)** δ 149.0, 137.8, 137.2, 128.3, 125.5, 115.8, 39.9, 34.5, 31.6. HRMS (EI$^+$) calculated $[C_{13}H_{18}]^+$ (M)$^+$: m/z 174.1403, found 174.1403.

^{1}H NMR, 500 MHz, $CDCl_3$

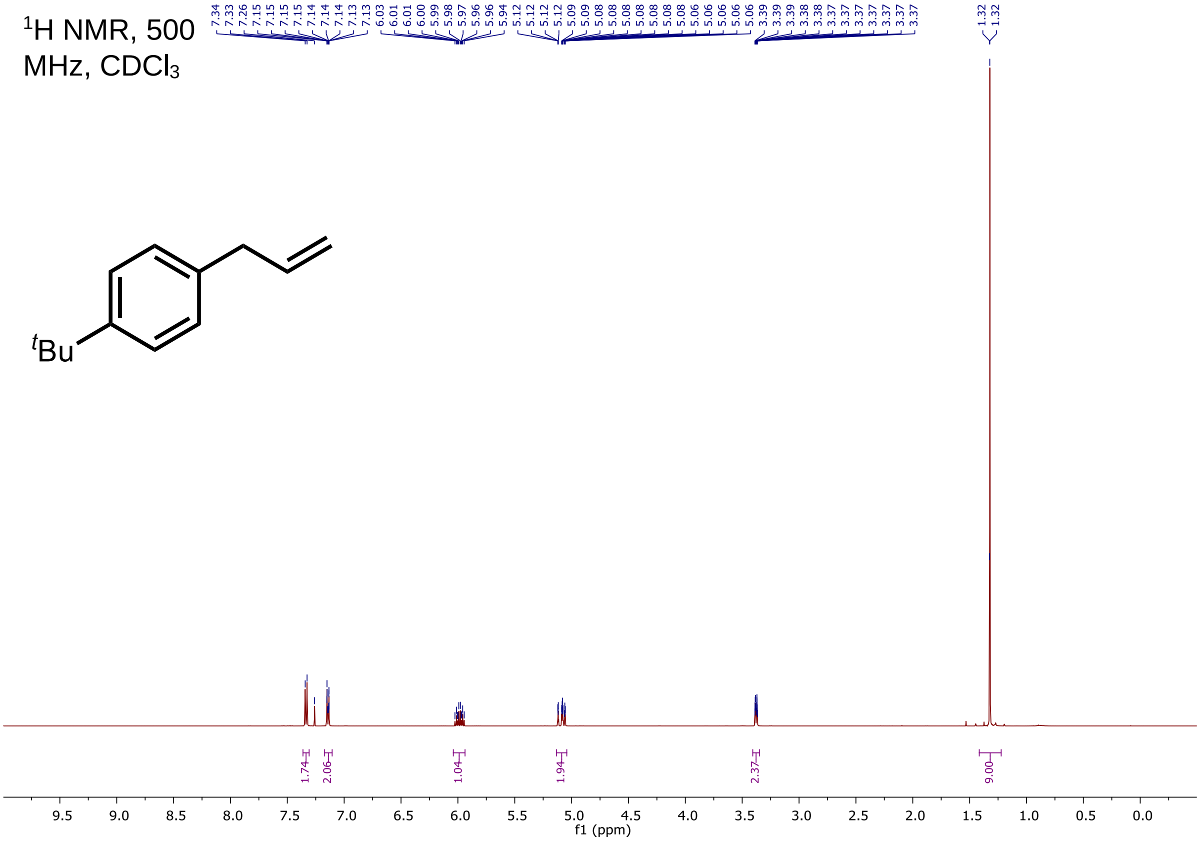

^{13}C NMR, 126 MHz, $CDCl_3$

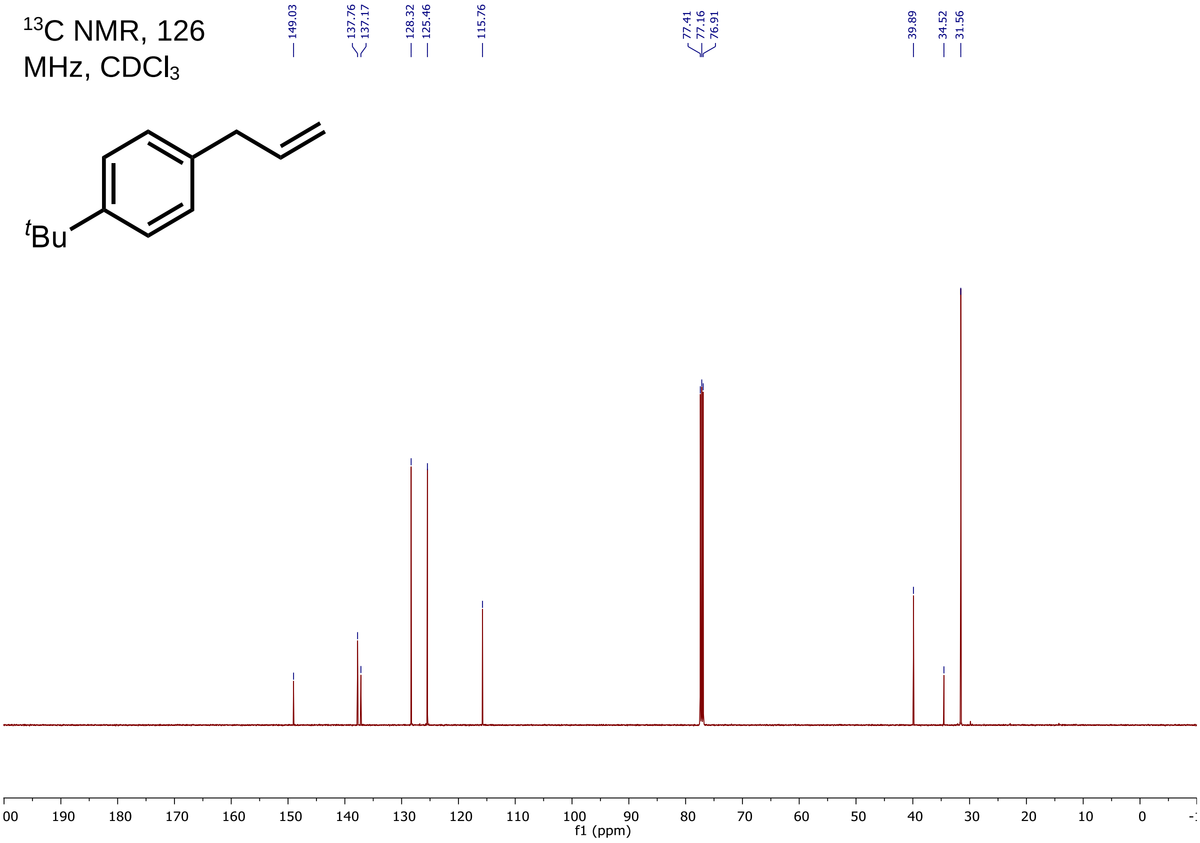

2-Allyl-6-methoxynaphthalene[2]

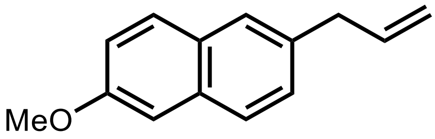

The title compound was prepared according to general procedure 2 using (6-methoxynaphthalen-2-yl)boronic acid (1 mmol). Purification by flash silica chromatography (eluent = 10% EtOAc in hexanes) gave the title compound as white solid (0.91 g, 46%); R_f: 0.48 (eluent = 10% EtOAc in hexanes); mp: 56–58 ˚C (Lit. 54–55 ˚C).[2] **^{1}H NMR (500 MHz, Chloroform-d)** δ 7.73 – 7.65 (m, 2H), 7.37 – 7.24 (m, 2H), 7.18 – 7.06 (m, 2H), 6.05 (ddt, *J* = 16.8, 10.1, 6.7 Hz, 1H), 5.20 – 4.99 (m, 2H), 3.92 (s, 3H), 3.62 – 3.37 (m, 2H); **^{13}C NMR (126 MHz, Chloroform-d)** δ 157.4, 137.7, 135.3, 133.3, 129.3, 129.1, 128.0, 127.0, 126.7, 118.9, 116.0, 105.8, 55.4, 40.3; HRMS (EI$^+$) calculated $[C_{14}H_{14}O]^+$ (M)$^+$: m/z 198.1039, found 198.1040.

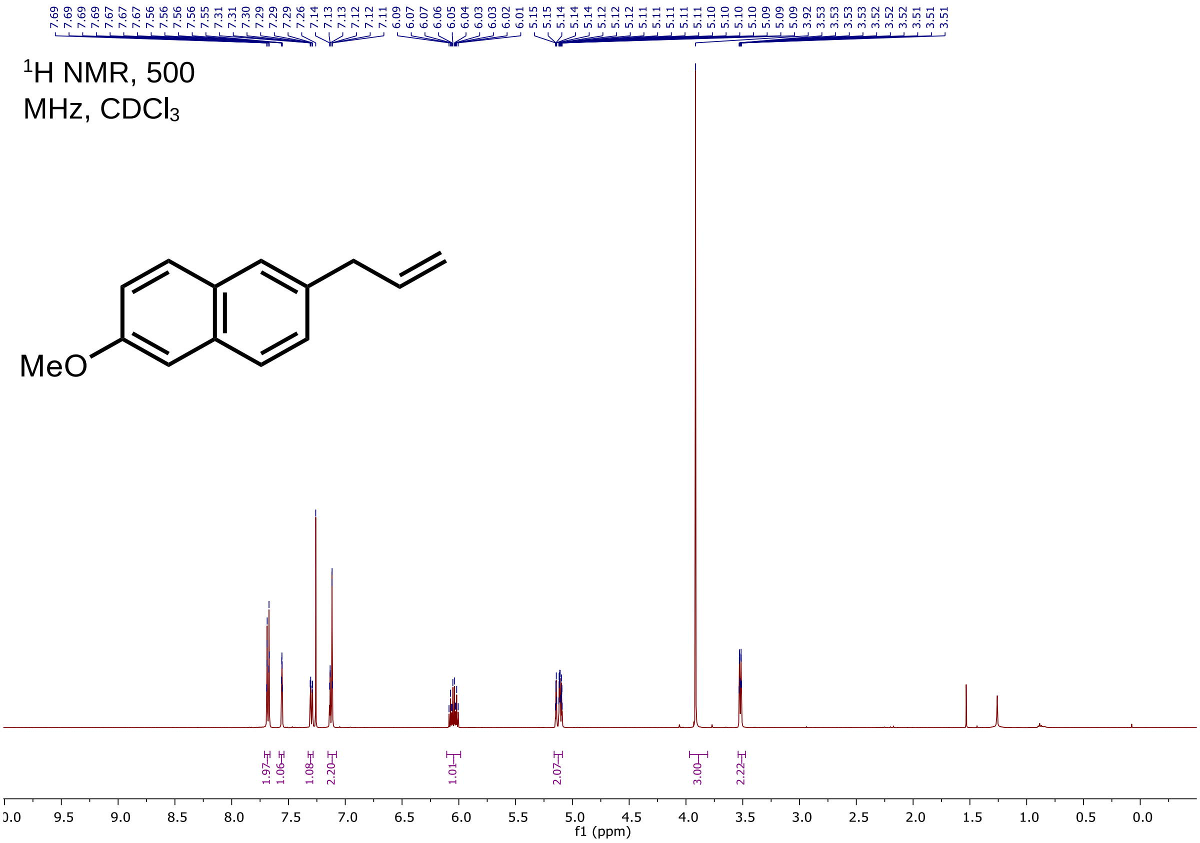

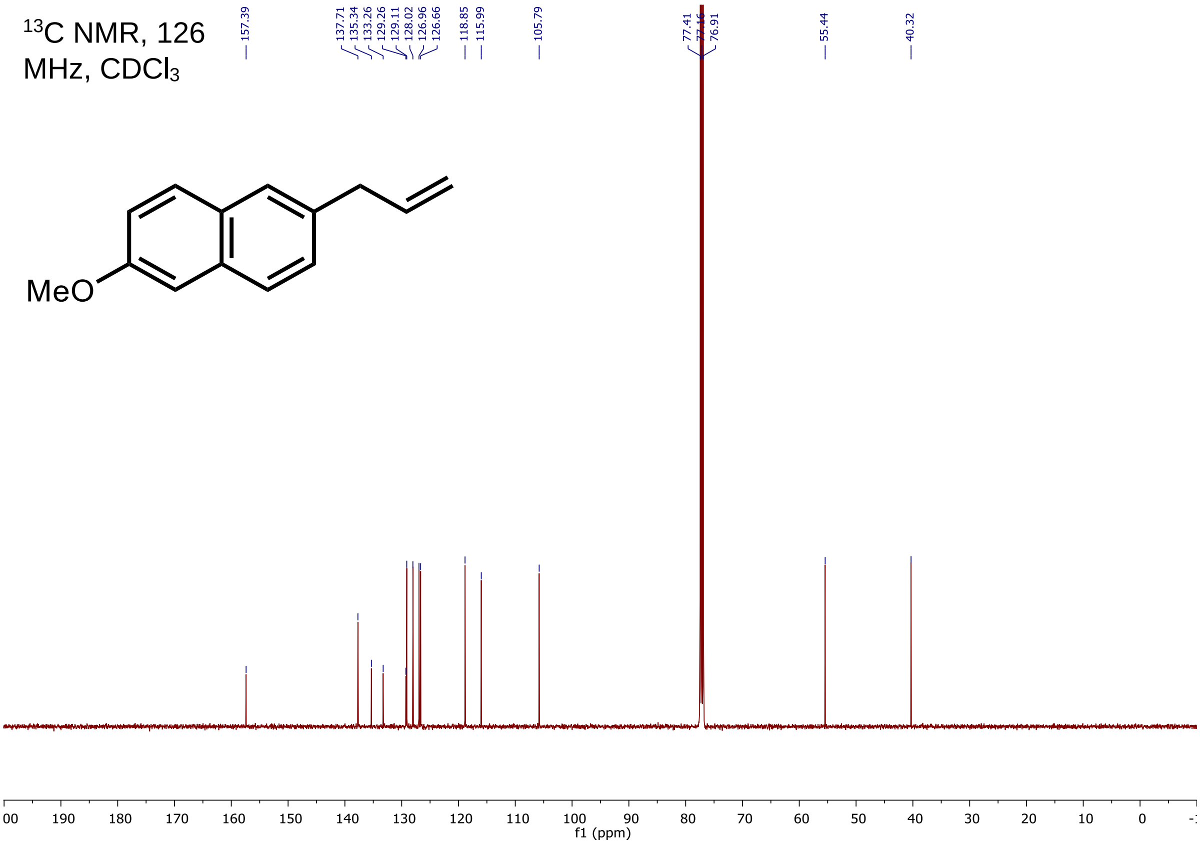

4-Allylbenzaldehyde[18]

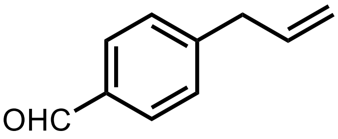

The title compound was prepared according to general procedure 2 using (4-formylphenyl)boronic acid (5 mmol). Purification by flash silica chromatography (eluent = 10% EtOAc in hexanes) gave the title compound as yellow oil (291 mg, 40%); R_f: 0.48 (eluent = 10% EtOAc in hexanes); **^{1}H NMR (500 MHz, Chloroform-d)** δ 9.97 (s, 1H), 7.85 – 7.75 (m, 2H), 7.39 – 7.29 (m, 2H), 5.95 (ddt, *J* = 16.9, 10.2, 6.7 Hz, 1H), 5.16 – 5.03 (m, 2H), 3.53 – 3.40 (m, 2H). **^{13}C NMR (126 MHz, Chloroform-d)** δ 192.1, 147.4, 136.0, 134.7, 130.0, 129.3, 116.9, 40.3; HRMS (EI$^-$) calculated $[C_{10}H_9O]^-$ (M-H)$^-$: m/z 145.0679, found 145.0645.

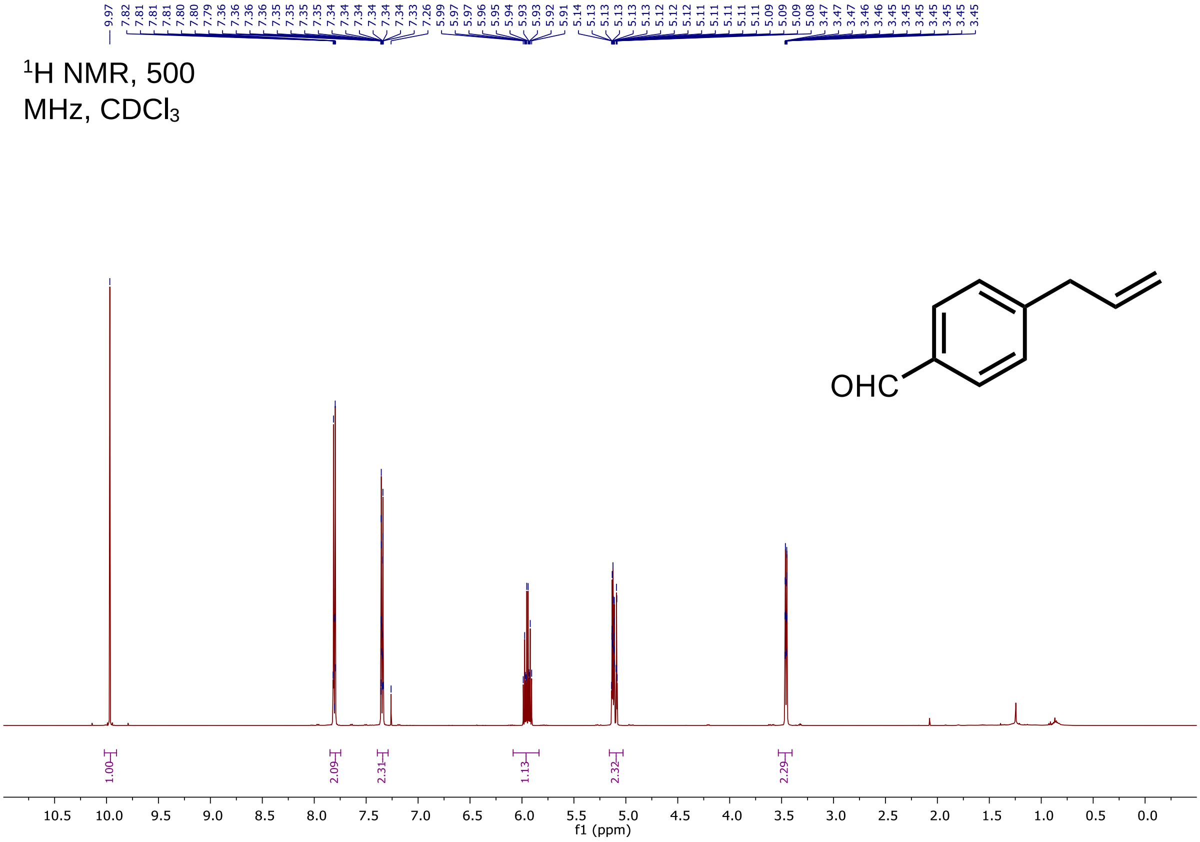
9.97
7.82
7.81
7.81
7.80
7.80
7.79
7.36
7.36
7.36
7.35
7.35
7.35
7.35
7.34
7.34
7.34
7.34
7.34
7.34
7.33
7.26
5.99
5.97
5.97
5.96
5.95
5.94
5.93
5.93
5.93
5.92
5.91
5.14
5.13
5.13
5.13
5.13
5.13
5.12
5.12
5.11
5.11
5.11
5.11
5.11
5.09
5.09
5.08
3.47
3.47
3.47
3.46
3.45
3.45
3.45
3.45
3.45
3.45
1H NMR, 500 MHz, CDCl3
OHC
1.00
2.09
2.31
1.13
2.32
2.29
f1 (ppm)

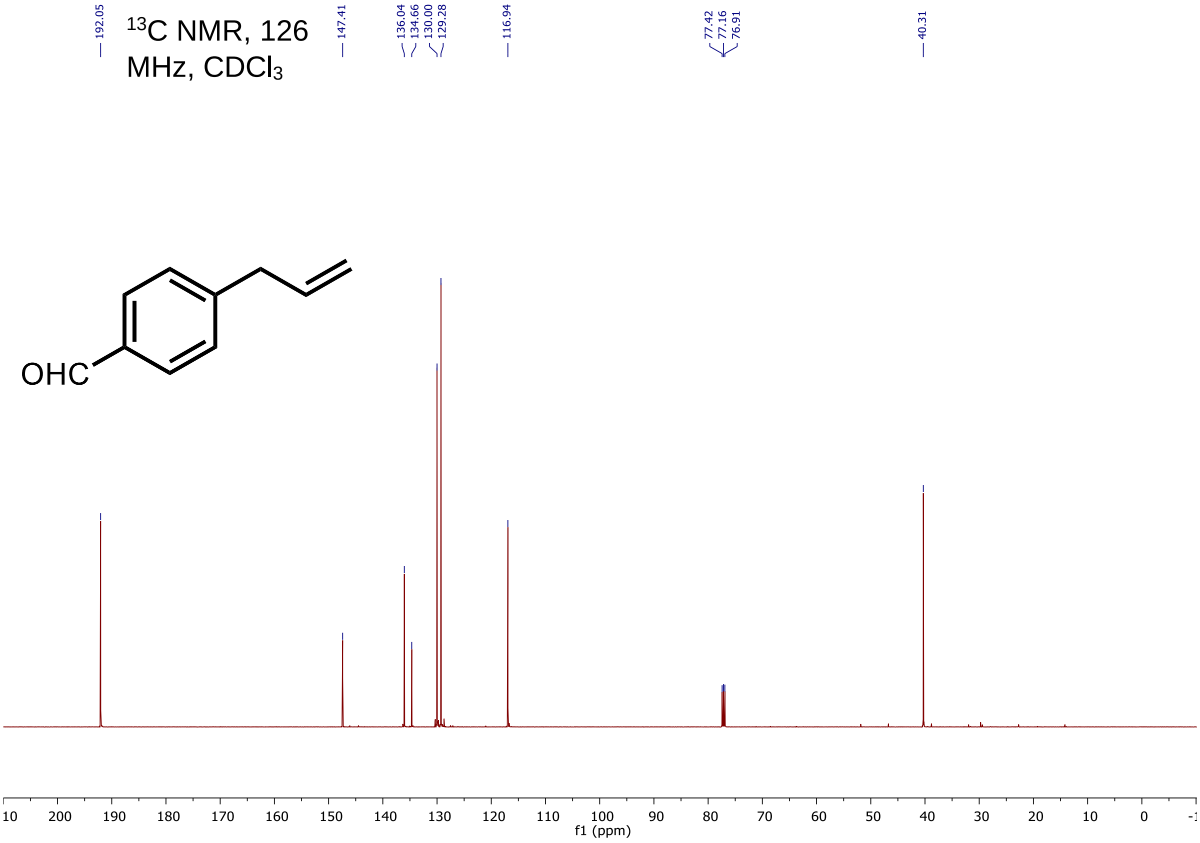
192.05
13C NMR, 126 MHz, CDCl3
147.41
136.04
134.66
130.00
129.28
116.94
77.42
77.16
76.91
40.31
OHC
f1 (ppm)

1-Allyl-4-bromobenzene[9]

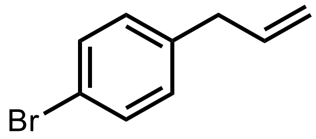

The title compound was prepared according to general procedure 2 using (4-bromophenyl)boronic acid (4 mmol). Purification by flash silica chromatography (eluent = 10% EtOAc in hexanes) gave the title compound as a pale yellow oil (154 mg, 20%); R_f: 0.69 (eluent = 10% EtOAc in hexanes); **^{1}H NMR (500 MHz, Chloroform-d)** δ 7.46 – 7.37 (m, 2H), 7.15 – 7.02 (m, 2H), 5.93 (ddt, *J* = 16.9, 10.3, 6.7 Hz, 1H), 5.11 – 5.05 (m, 2H), 3.34 (dt, *J* = 6.7, 1.5 Hz, 2H). **^{13}C NMR (126 MHz, Chloroform-d)** δ 139.1, 136.9, 131.6, 130.5, 120.0, 116.4, 39.7; HRMS (EI$^+$) calculated $[C_9H_9Br]^+$ (M)$^+$: m/z 195.9882, found 195.9882.

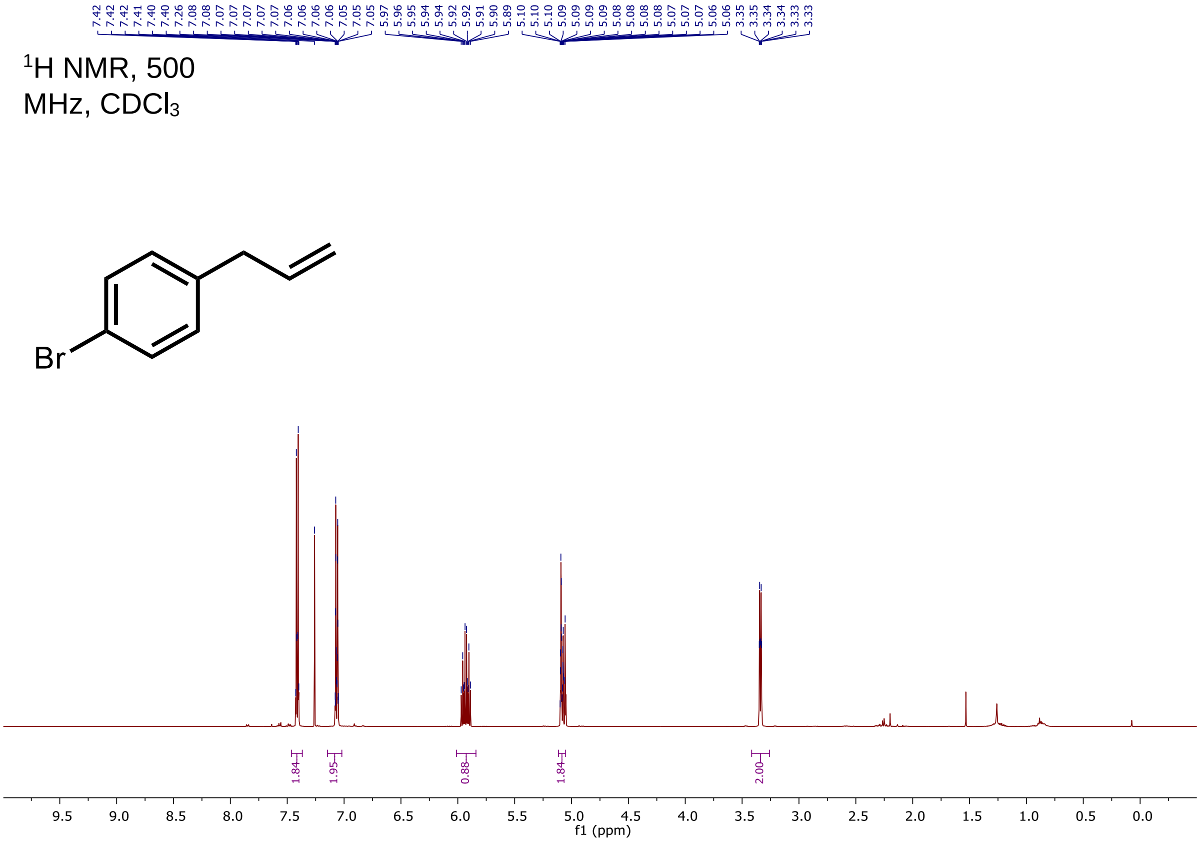

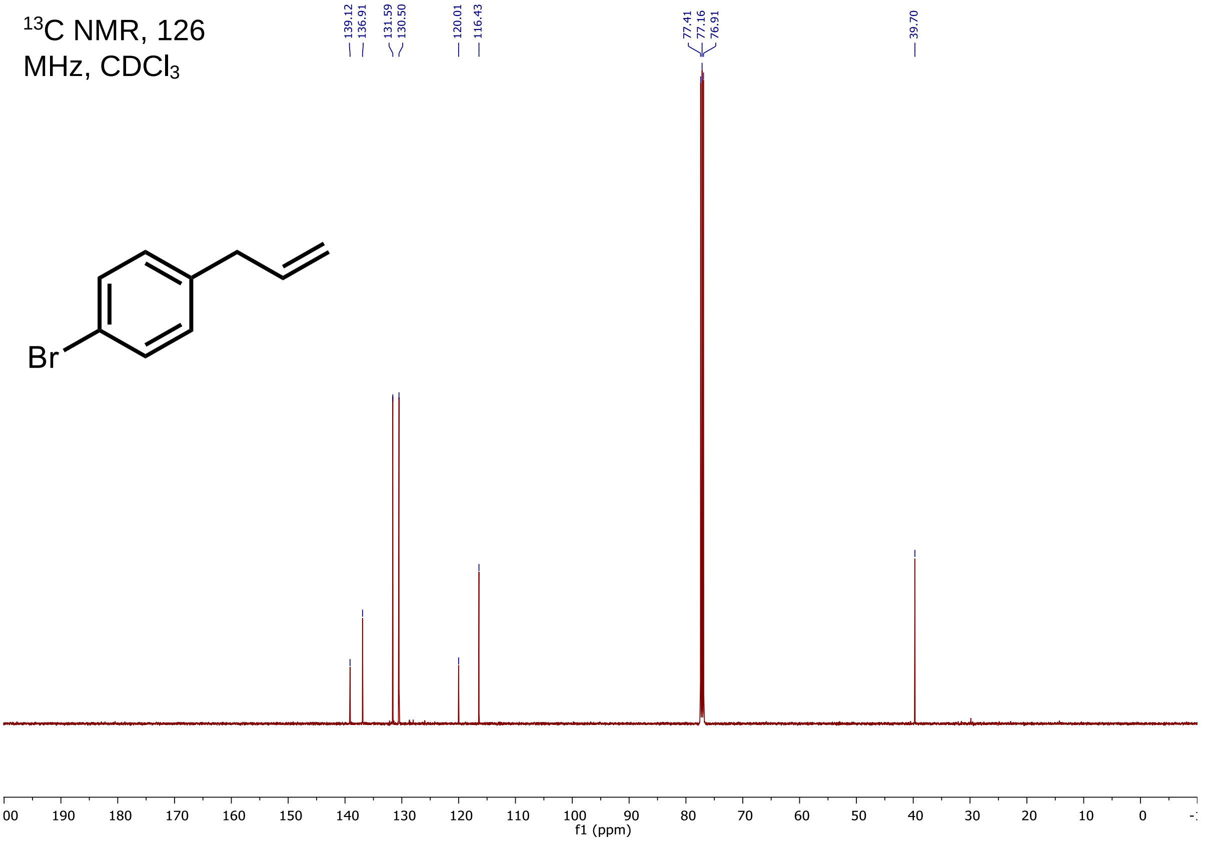

1-Allyl-4-iodobenzene[19]

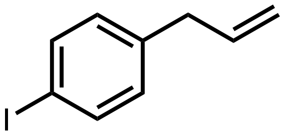

The title compound was prepared according to general procedure 2 using (4-iodophenyl)boronic acid (5 mmol). Purification by flash silica chromatography (eluent = 10% EtOAc in hexanes) gave the title compound as a pale yellow oil (396 mg, 32%); R_f: 0.80 (eluent = 10% EtOAc in hexanes**)**; **^{1}H NMR (500 MHz, Chloroform-d) δ** 7.65 – 7.55 (m, 2H), 6.99 – 6.89 (m, 2H), 5.92 (ddt, *J* = 16.9, 10.4, 6.7 Hz, 1H), 5.13 – 5.02 (m, 2H), 3.37 – 3.27 (m, 2H); **^{13}C NMR (126 MHz, Chloroform-d)** δ 139.8, 137.5, 136.9, 130.9, 116.5, 91.4, 39.8; HRMS (EI$^+$) calculated $[C_9H_9I]^+$ (M)$^+$: m/z 243.9743, found 243.9745.

^{1}H NMR, 500 MHz, $CDCl_3$

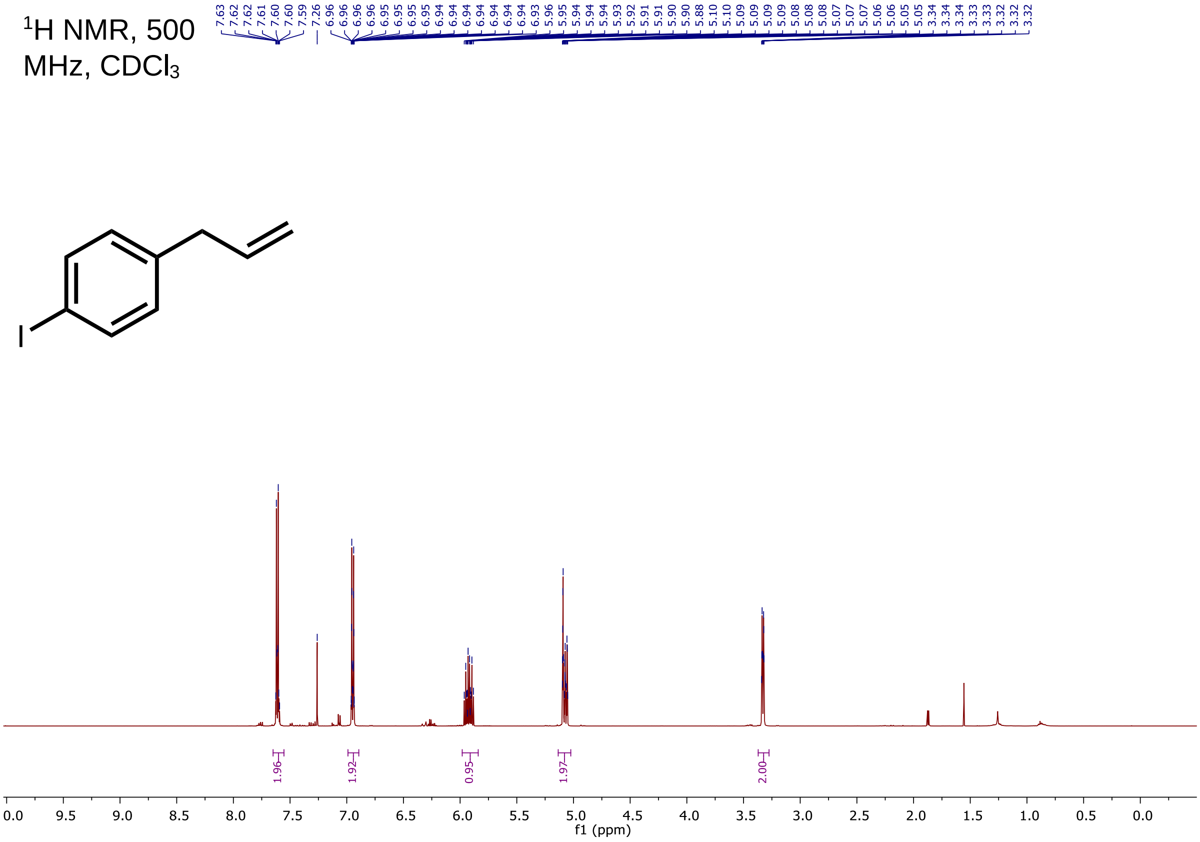

^{13}C NMR, 126 MHz, $CDCl_3$

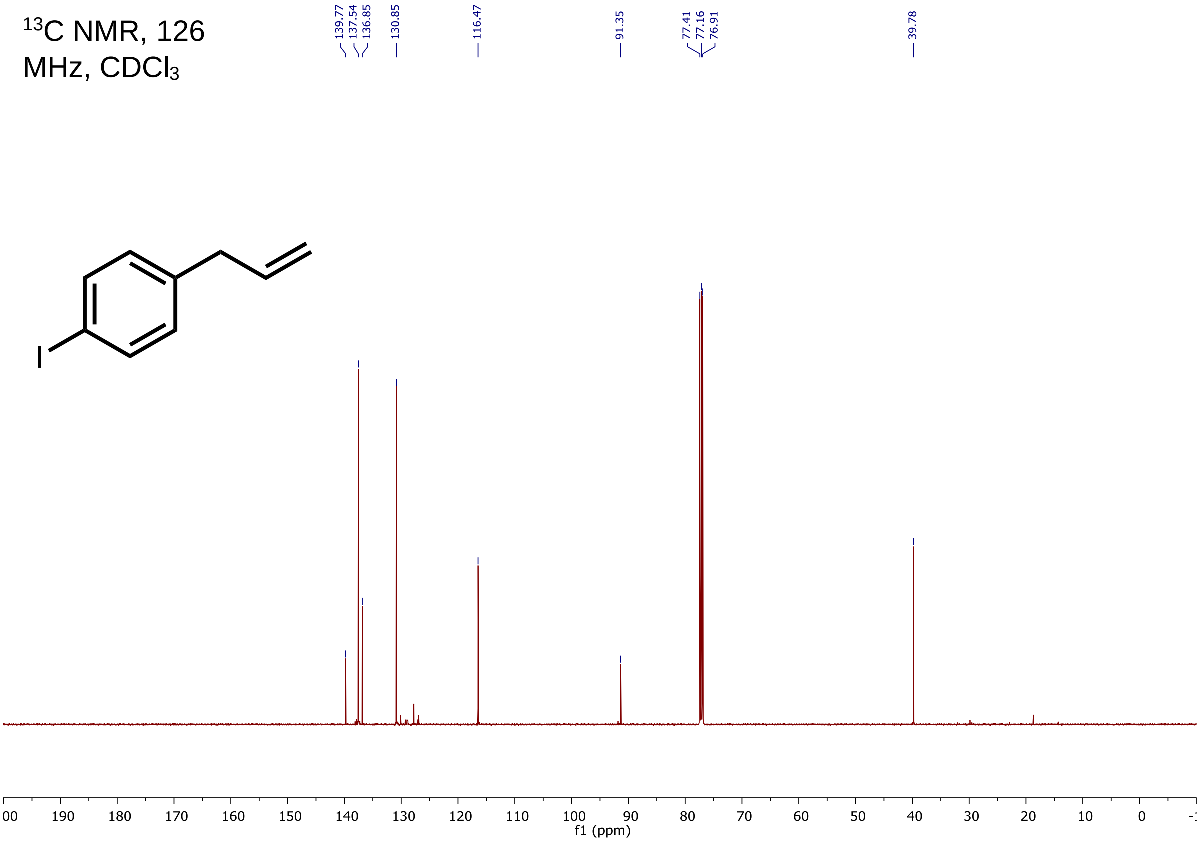

5-Allylbenzofuran[20]

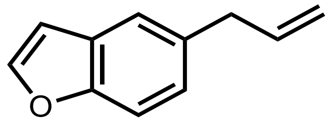

The title compound was prepared according to general procedure 2 using benzofuran-5-ylboronic acid (3 mmol). Purification by flash silica chromatography (eluent = 10% EtOAc in hexanes) gave the title compound as a pale yellow oil (304 mg, 62%); R_f: 0.58 (eluent = 10% EtOAc in hexanes); **^{1}H NMR (500 MHz, Chloroform-d) δ** 7.60 (d, *J* = 2.2 Hz, 1H), 7.44 – 7.40 (m, 2H), 7.15 – 7.11 (m, 1H), 6.72 (dd, *J* = 2.2, 1.0 Hz, 1H), 6.02 (ddt, *J* = 16.8, 10.1, 6.7 Hz, 1H), 5.12 – 5.06 (m, 2H), 3.49 (dtd, *J* = 6.7, 1.5, 0.7 Hz, 2H); **^{13}C NMR (126 MHz, Chloroform-d)** δ 153.9, 145.3, 138.2, 134.6, 127.7, 125.2, 120.8, 115.7, 111.2, 106.6, 40.3; HRMS (CI$^+$) calculated $[C_{11}H_{10}O]^+$ (M)$^+$: m/z 158.0726, found 158.0723.

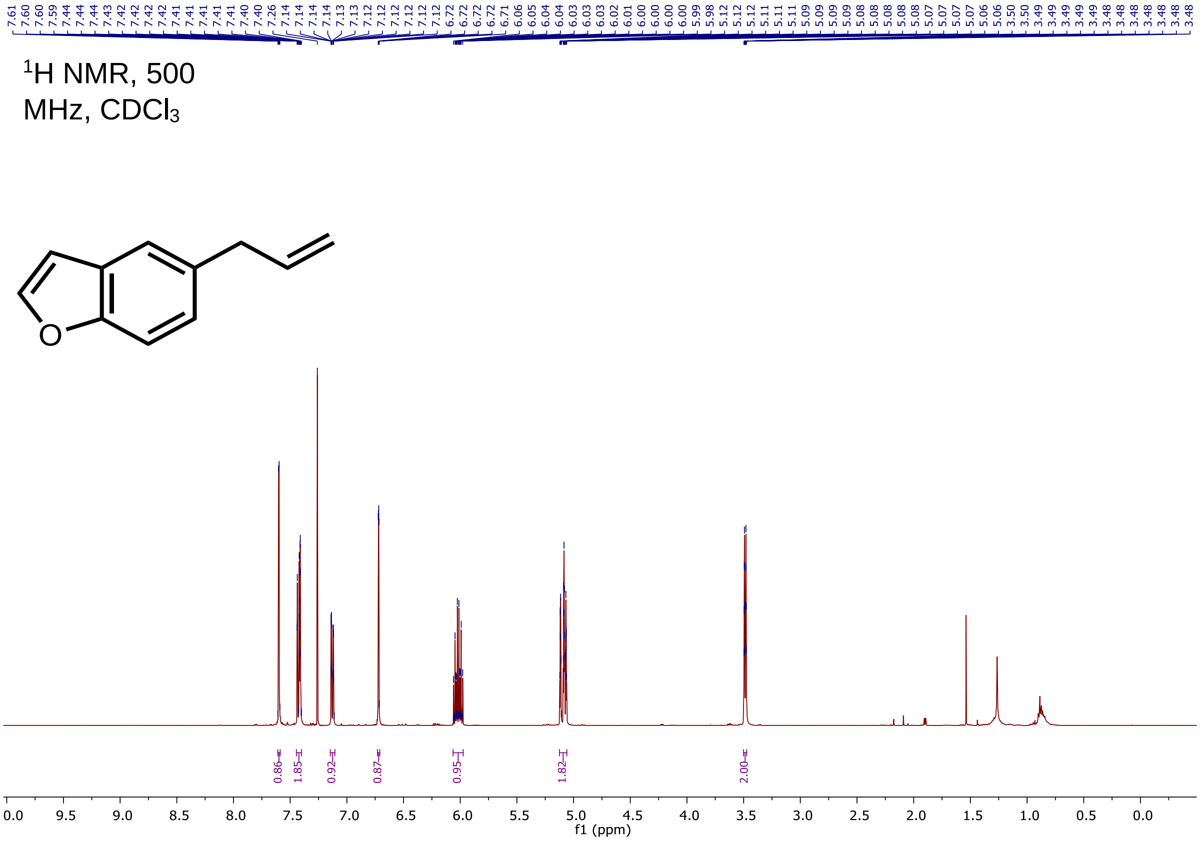

^{13}C NMR, 126 MHz, $CDCl_3$

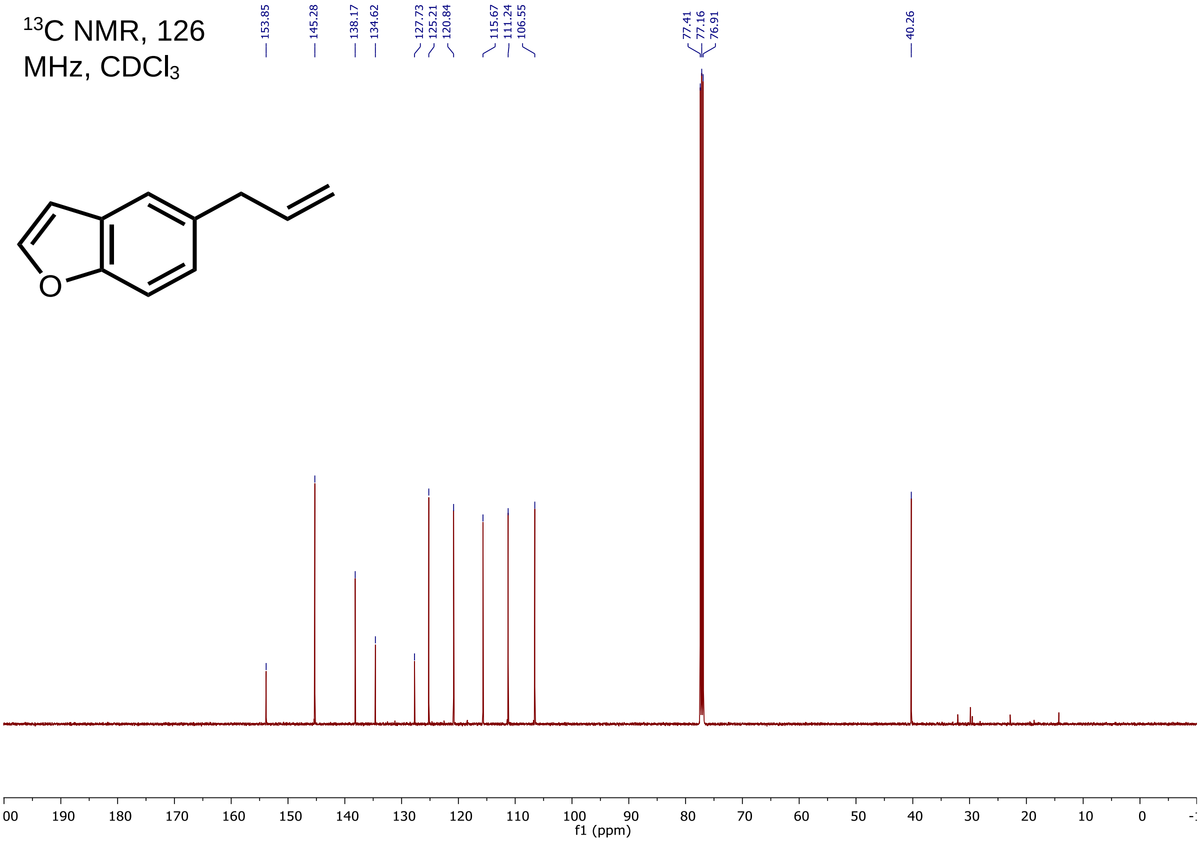

2.1.3. Deuterated substrates synthesis[21,22]

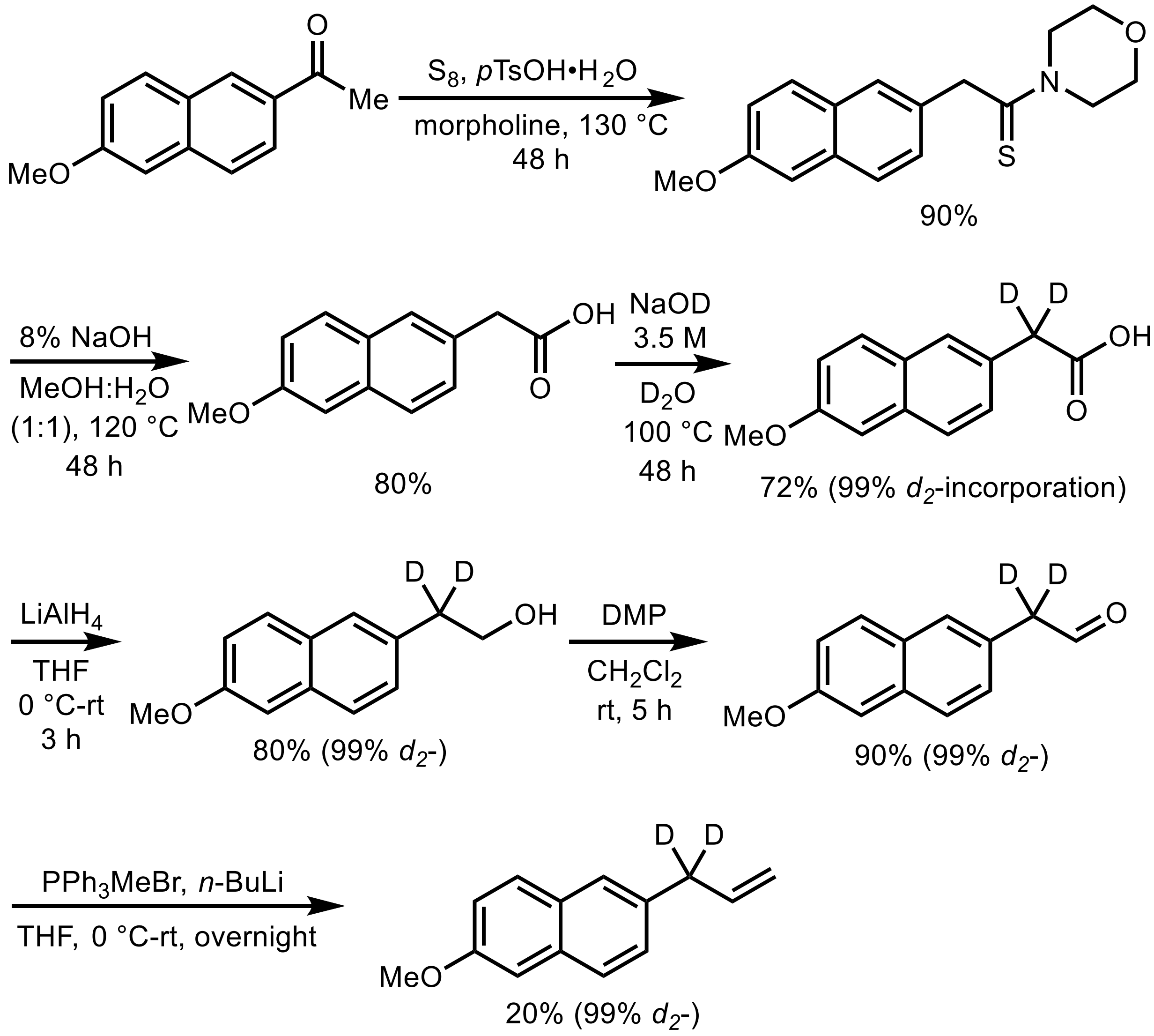

2-(6-methoxynaphthalen-2-yl)-1-morpholinoethanethione[22]

6'-methoxy-2'-acetonaphthone (4.00 g, 20 mmol), sulfur (1.28 g, 40 mmol), and *p*-toluenesulfonic acid monohydrate (57 mg, 0.3 mmol) were dissolved in morpholine (5.2 mL, 60 mmol). The mixture was refluxed at 130 ˚C which subsequently formed a deep red solution. The reaction was continued for 48 h. The reaction mixture was cooled to rt, then diluted with CH_2Cl_2 (10 mL), and washed with $NaHCO_3$ (3 x 10 mL) then brine (1 x 10 mL). The organic layer was dried over $MgSO_4$, concentrated *in vacuo*, and purified by column chromatography (eluent = EtOAc:PE 1:1) to give the title compound as a yellow solid (5.42 g, 90%); mp 128–130 ˚C (lit. 134–135 ˚C)[23]; R_f: 0.50 (eluent = EtOAc:PE 1:1); **^{1}H NMR (500 MHz, Chloroform-*d*)** δ 7.70 (dd, *J* = 14.0, 8.7 Hz, 2H), 7.66 – 7.64 (m, 1H), 7.44 (dd, *J* = 8.4, 1.8 Hz, 1H), 7.18 – 7.10 (m, 2H), 4.48 (d, *J* = 1.0 Hz, 2H), 4.42 – 4.35 (m, 2H), 3.92 (s, 3H), 3.77 – 3.73 (m, 2H), 3.69 – 3.63 (m, 2H), 3.40 – 3.33 (m, 2H); **^{13}C NMR (126 MHz, Chloroform-*d*)** δ 200.3, 157.9, 133.7, 130.9, 129.3, 129.2, 127.7, 126.6, 126.3, 119.4, 105.8, 66.5, 66.3, 55.5, 50.9, 50.8, 50.4; HRMS (CI^+) calculated [$C_{17}H_{20}NO_2S$] $(M+H)^+$: m/z 302.1215, found 302.1215.

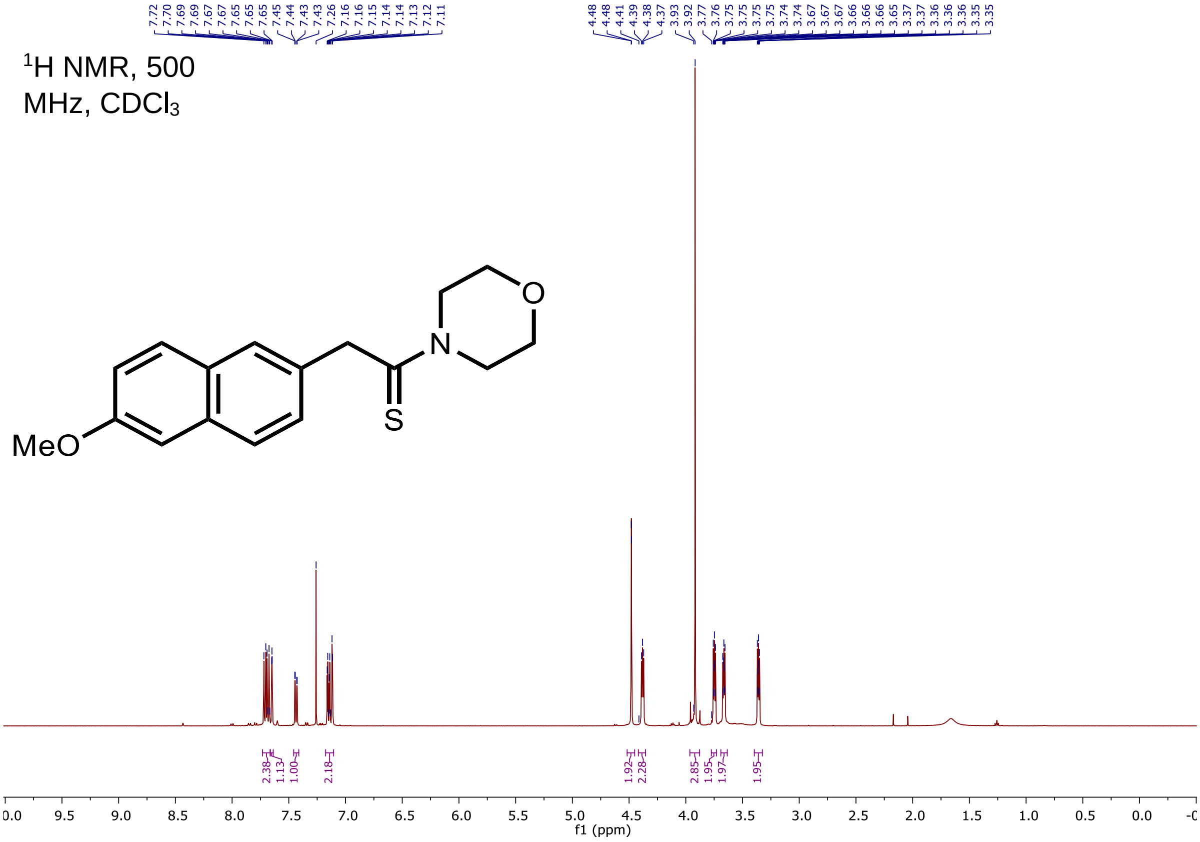

7.72
7.70
7.69
7.69
7.67
7.67
7.65
7.65
7.65
7.45
7.44
7.43
7.43
7.26
7.16
7.16
7.15
7.14
7.14
7.13
7.12
7.11
4.48
4.48
4.41
4.39
4.38
4.37
3.93
3.92
3.77
3.76
3.75
3.75
3.75
3.75
3.74
3.74
3.67
3.67
3.67
3.66
3.66
3.66
3.65
3.37
3.37
3.36
3.36
3.36
3.35
3.35
^{1}H NMR, 500 MHz, $CDCl_3$
MeO
N
O
S
2.38
1.13
1.00
2.18
1.92
2.28
2.85
1.95
1.97
1.95
f1 (ppm)

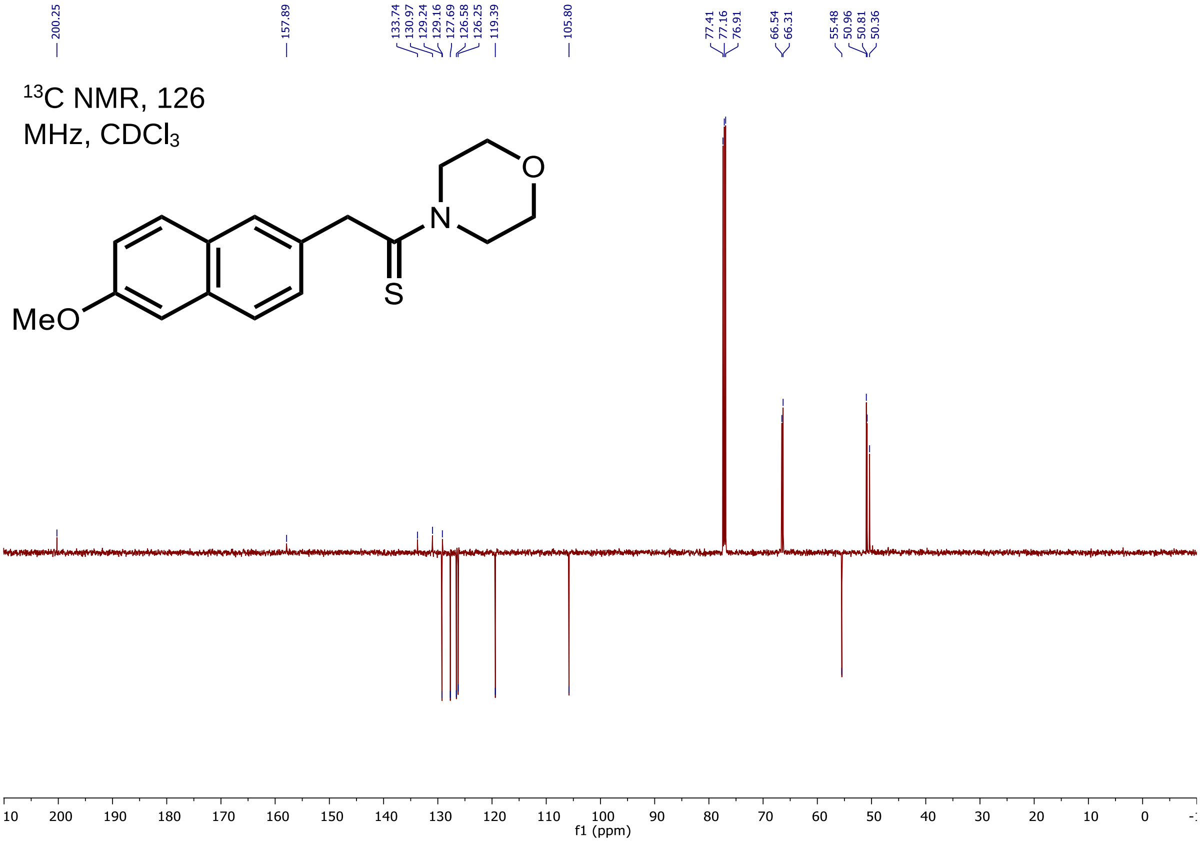

200.25
157.89
133.74
130.97
129.24
129.16
127.69
126.58
126.25
119.39
105.80
77.41
77.16
76.91
66.54
66.31
55.48
50.96
50.81
50.36
^{13}C NMR, 126 MHz, $CDCl_3$
MeO
N
O
S
f1 (ppm)

2-(6-methoxynaphthalen-2-yl)acetic acid[22]

2-(6-methoxynaphthalen-2-yl)-1-morpholinoethanethione (3.62 g, 12 mmol) was dissolved in 8% NaOH in MeOH:H_2O 1:1 (40 mL, 70 mmol). The mixture was refluxed at 120 ˚C for 48 h. Upon completion, the reaction was cooled to rt then diluted with CH_2Cl_2 (10 mL). The aqueous solution was acidified with 1 M HCl until pH 2. The aqueous solution then extracted with CH_2Cl_2 (5 x 50 mL), dried over $MgSO_4$, concentrated *in vacuo*, then rinsed with hexanes to give the title compound as yellow solid (2.07 g, 80%); mp 168–170 ˚C (lit. 170 ˚C)[23]; R_f: 0.00 (eluent = 20% EtOAc in PE); **^{1}H NMR (500 MHz, Chloroform-*d*)** δ 7.73 – 7.68 (m, 2H), 7.68 – 7.65 (m, 1H), 7.37 (dd, *J* = 8.4, 1.8 Hz, 1H), 7.16 – 7.10 (m, 2H), 3.92 (s, 3H), 3.78 (d, *J* = 0.7 Hz, 2H); **^{13}C NMR (126 MHz, Chloroform-*d*)** δ 176.7, 157.9, 133.9, 129.3, 129.1, 128.5, 128.1, 127.9, 127.4, 119.2, 105.8, 55.5, 40.9; HRMS (CI$^+$) calculated [$C_{13}H_{12}O_3$] (M)$^+$: m/z 216.0786, found 216.0786.

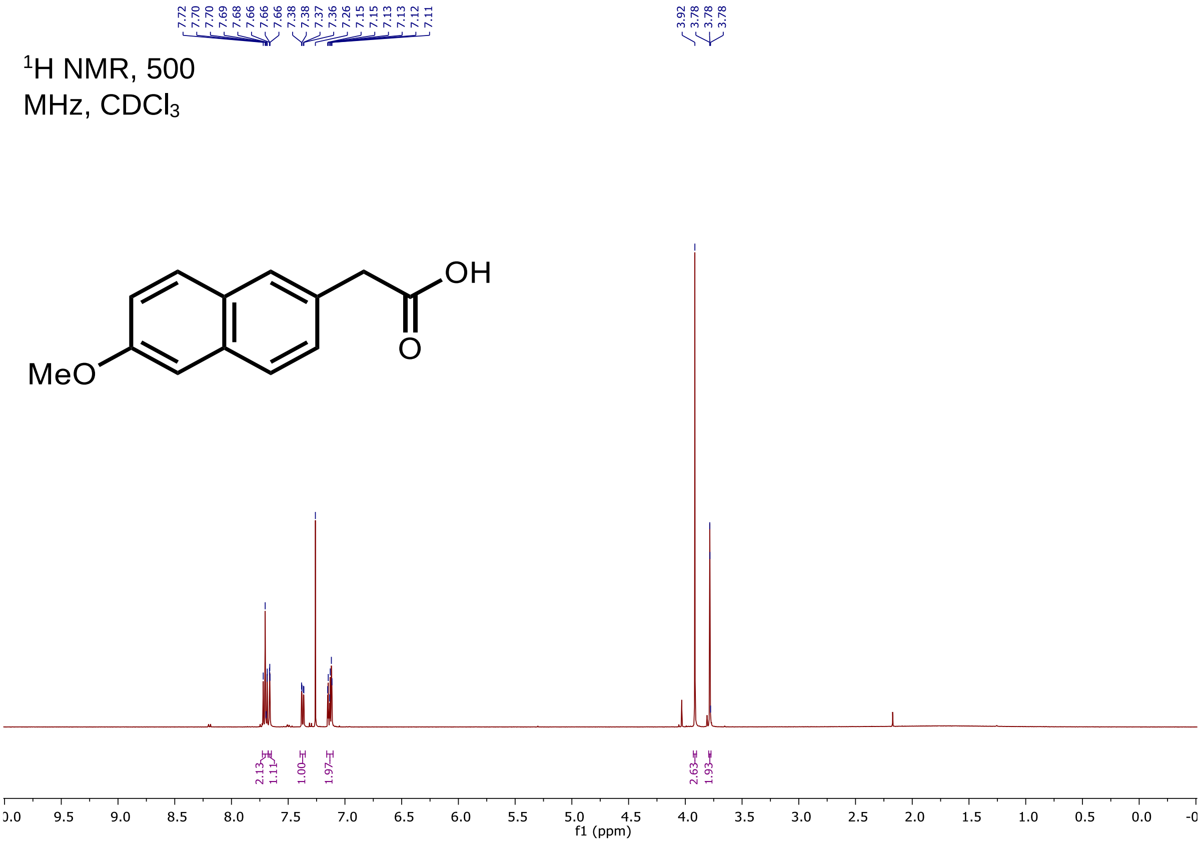

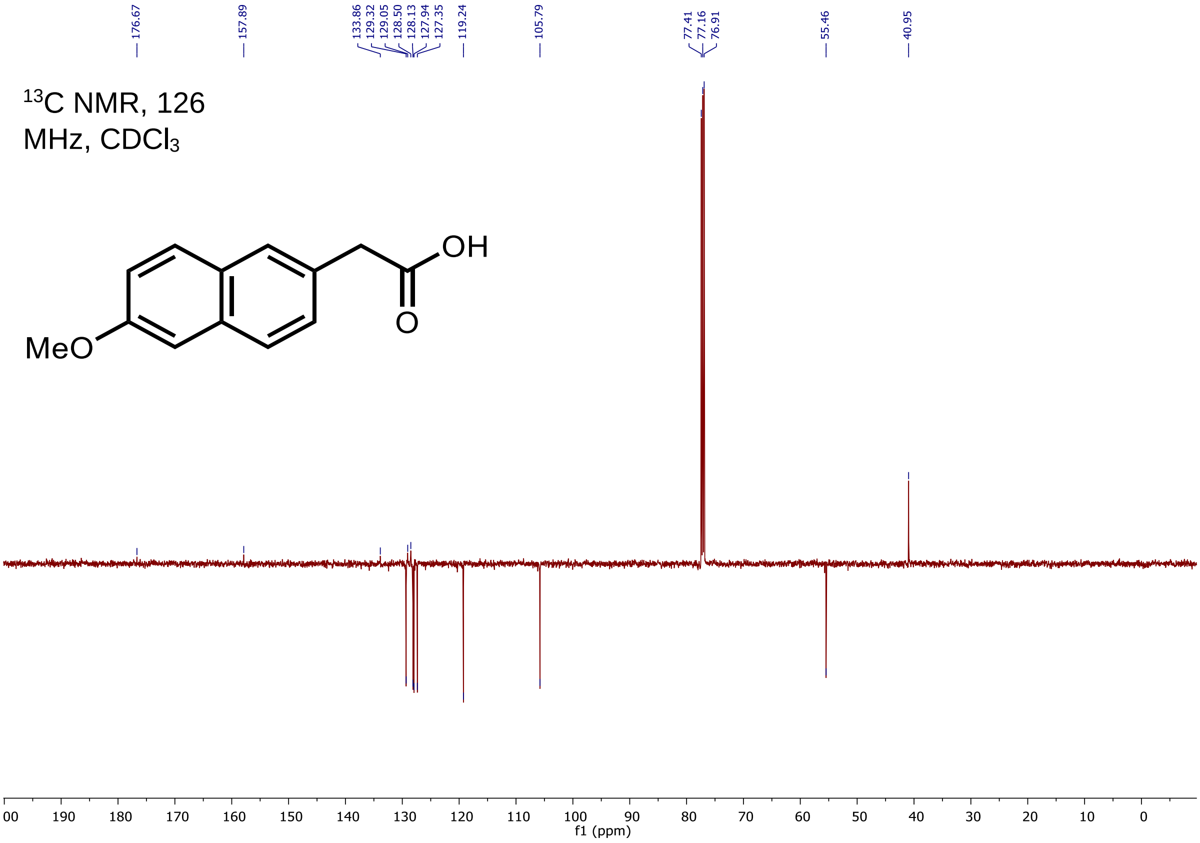

2-(6-methoxynaphthalen-2-yl)acetic-2,2-d_2 acid[21]

2-(6-methoxynaphthalen-2-yl)acetic acid (1.99 g, 9 mmol) was dissolved in NaOD 3.5 M in D_2O (5.3 mL, 18 mmol) in oven-dried J-Young-Schlenk flask. The flask was sealed and refluxed at 100 ˚C for 48 h. The reaction was cooled to rt then 1 M HCl was added until pH 2. The mixture was extracted with CH_2Cl_2 (5 x 30 mL), dried over $MgSO_4$, filtered, and concentrated *in vacuo* to give the title compound as a yellow solid and used without further purification (1.41 g, 72% with 99% d_2- incorporation); $ν_{max}$ / cm^{-1} (film) 2937, 2889, 1694, 1607, 1487, 1265, 1242, 1161, 1028, 849, 816; **^{1}H NMR (500 MHz, Chloroform-*d*)** δ 7.75 – 7.64 (m, 3H), 7.41 – 7.35 (m, 1H), 7.13 (ddt, *J* = 10.0, 4.9, 2.3 Hz, 2H), 3.96 – 3.88 (m, 3H); **^{13}C NMR (126 MHz, Chloroform-*d*)** δ 176.6, 157.9, 133.9, 129.3, 129.1, 128.4, 128.1, 127.9, 127.4, 119.2, 105.8, 55.5, 40.5; HRMS (CI$^+$) calculated [$C_{13}H_{10}D_2O_3$] $(M)^+$: m/z 218.0912, found 218.0912.

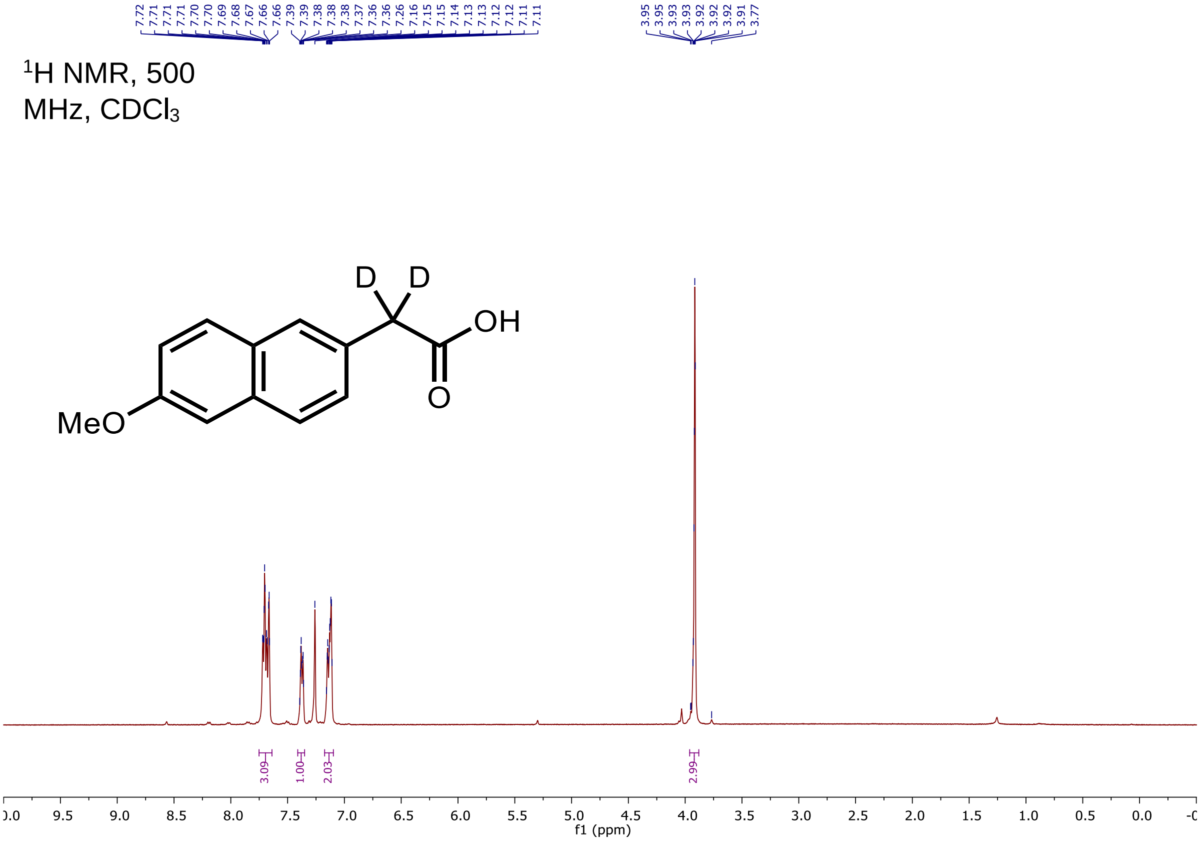
7.72
7.71
7.71
7.70
7.70
7.69
7.68
7.67
7.66
7.66
7.39
7.39
7.38
7.38
7.37
7.36
7.36
7.26
7.16
7.15
7.15
7.14
7.13
7.13
7.12
7.11
7.11
3.95
3.95
3.93
3.93
3.92
3.92
3.92
3.91
3.77
1H NMR, 500 MHz, CDCl3
D
D
OH
O
MeO
3.09
1.00
2.03
2.99
f1 (ppm)

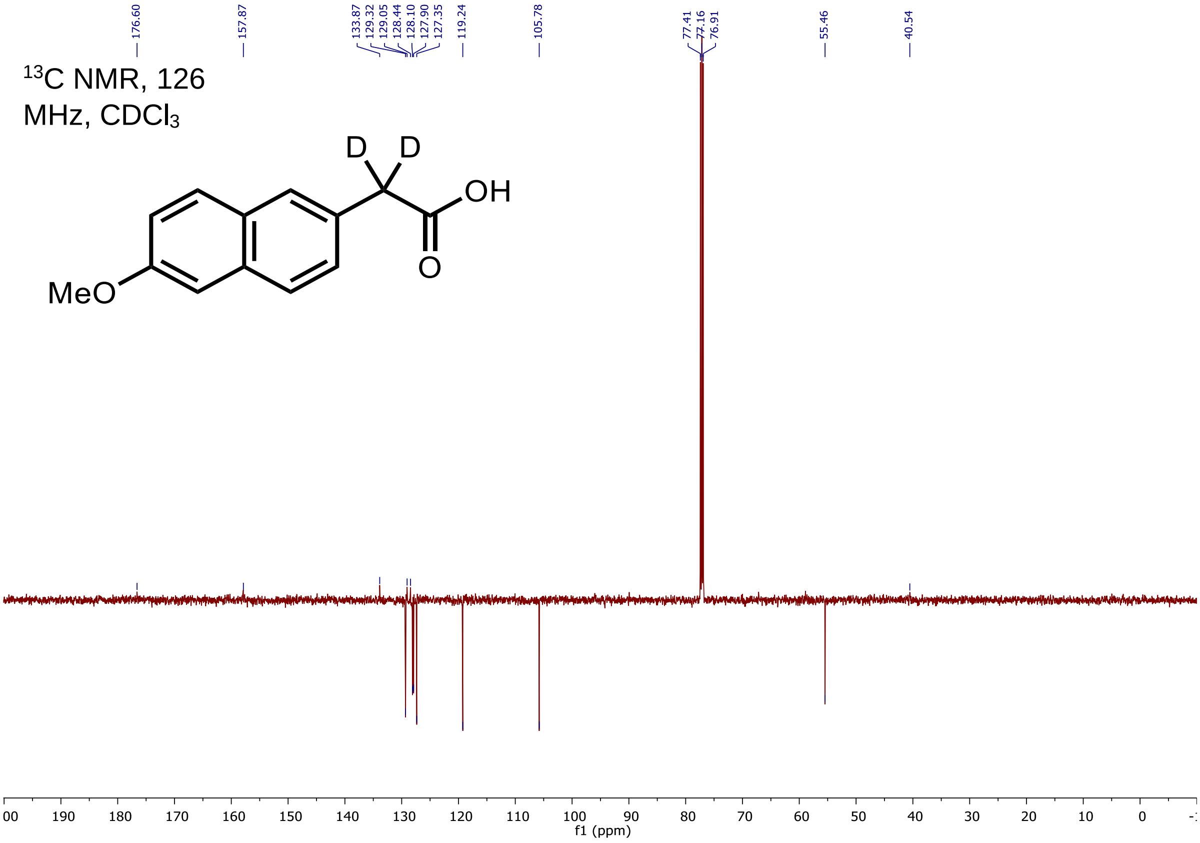
176.60
157.87
133.87
129.32
129.05
128.44
128.10
127.90
127.35
119.24
105.78
77.41
77.16
76.91
55.46
40.54
13C NMR, 126 MHz, CDCl3
D
D
OH
O
MeO
f1 (ppm)

2-(6-methoxynaphthalen-2-yl)ethan-2,2-d_2-1-ol[21]

$LiAlH_4$ (0.20 g, 5.2 mmol) was added to dry THF (5 mL) and cooled to 0 ˚C. Then a solution of 2-(6-methoxynaphthalen-2-yl)acetic-2,2-d_2 acid (1.09 g, 5 mmol) in dry THF (6 mL) was added dropwise. The reaction was stirred for 3 h. After completion, the mixture was quenched with 0.5 M HCl, filtered, washed with EtOAc. The organic phase was washed with brine, dried over $MgSO_4$, and concentrated *in vacuo*. Purification by flash silica chromatography (eluent = EtOAc:PE 1:1) gave the title compound as white solid (0.82 g, 80% with 99% d_2-incorporation); mp 117–119 ˚C; R_f: 0.49 (eluent = EtOAc:PE 1:1); ν_{max} / cm^{-1} (film) 3291, 2880, 1697, 1449, 1391, 1236, 1061, 1049; **^{1}H NMR (500 MHz, Chloroform-*d*)** δ 7.72 – 7.68 (m, 2H), 7.61 (dt, *J* = 1.9, 0.5 Hz, 1H), 7.33 (dd, *J* = 8.4, 1.8 Hz, 1H), 7.17 – 7.11 (m, 2H), 3.92 (d, *J* = 4.2 Hz, 5H); **^{13}C NMR (126 MHz, Chloroform-*d*)** δ 157.5, 133.6, 133.5, 129.2, 129.1, 128.0, 127.5, 127.3, 119.1, 105.8, 63.7, 55.5, 39.3; HRMS (CI^+) calculated $[C_{13}H_{12}D_2O_2]^+$ $(M)^+$: m/z 204.1113, found 204.1114.

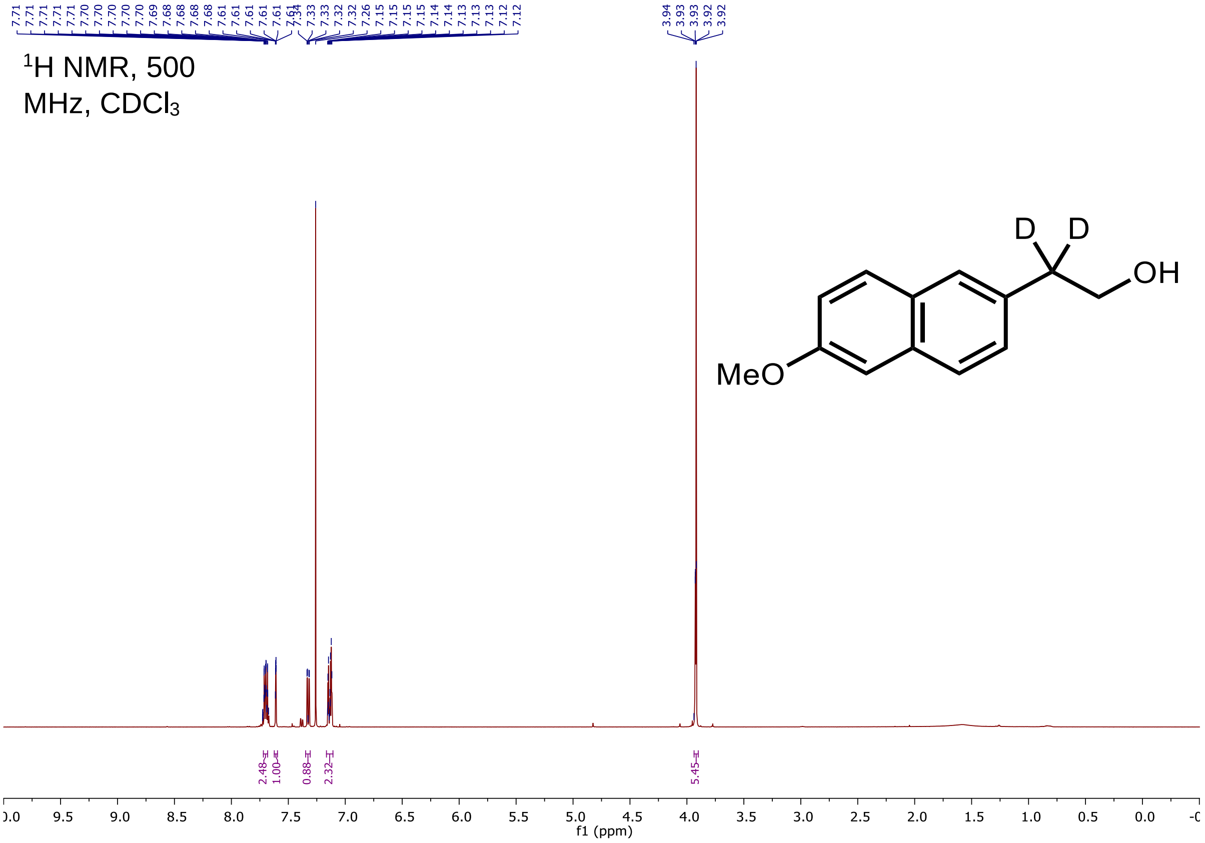

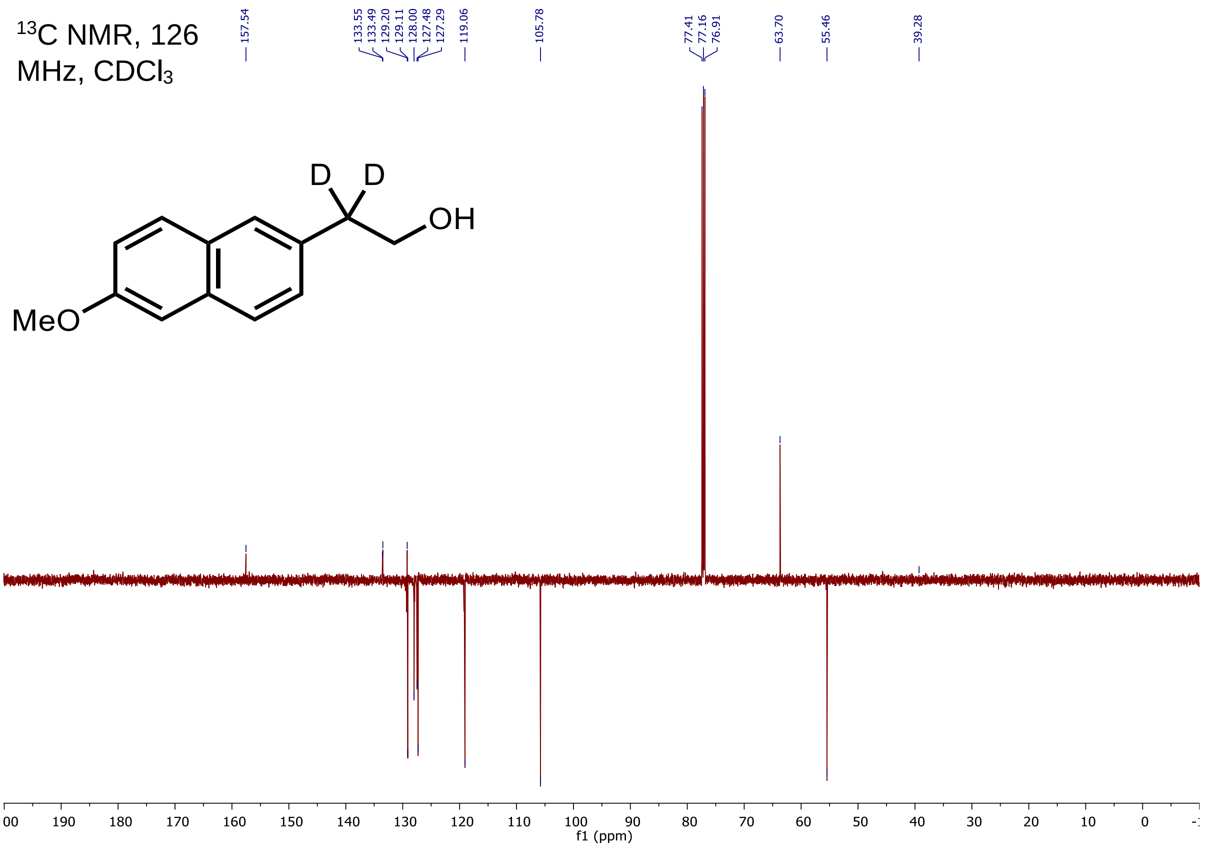

2-(6-methoxynaphthalen-2-yl)acetaldehyde-2,2-d$_2$[21]

Dess-Martin periodinane (0.64 g, 1.5 mmol) was diluted in CH_2Cl_2 (2 mL). Then 2-(6-methoxynaphthalen-2-yl)ethan-2,2-d$_2$-1-ol solution (0.26 g, 1.25 mmol) in CH_2Cl_2 (3 mL) was added to the mixture and stirred at rt for 5 h. The solution was filtered with silica and celite plug, dried over $MgSO_4$, concentrated *in vacuo*, and used without further purification to give a pale yellow solid (0.23 g, 90% with 99% d$_2$-incorporation); R_f: 0.38 (eluent = 10% EtOAc in PE); **^{1}H NMR (300 MHz, Chloroform-*d*)** δ 9.81 (s, 1H), 7.71 (dq, *J* = 8.3, 0.6 Hz, 2H), 7.63 – 7.61 (m, 1H), 7.28 (dd, *J* = 8.4, 1.8 Hz, 1H), 7.13 (dt, *J* = 4.1, 2.0 Hz, 2H), 3.92 (s, 3H); **^{13}C NMR (75 MHz, Chloroform-*d*)** δ 206.1, 156.1, 133.0, 131.2, 131.1, 129.5, 129.2, 128.5, 128.1, 119.5, 105.7, 55.5, 44.8; HRMS (CI) calculated $[C_{13}H_{10}D_2O_2]^+$ $(M)^+$: m/z 202.0957, found 202.0958.

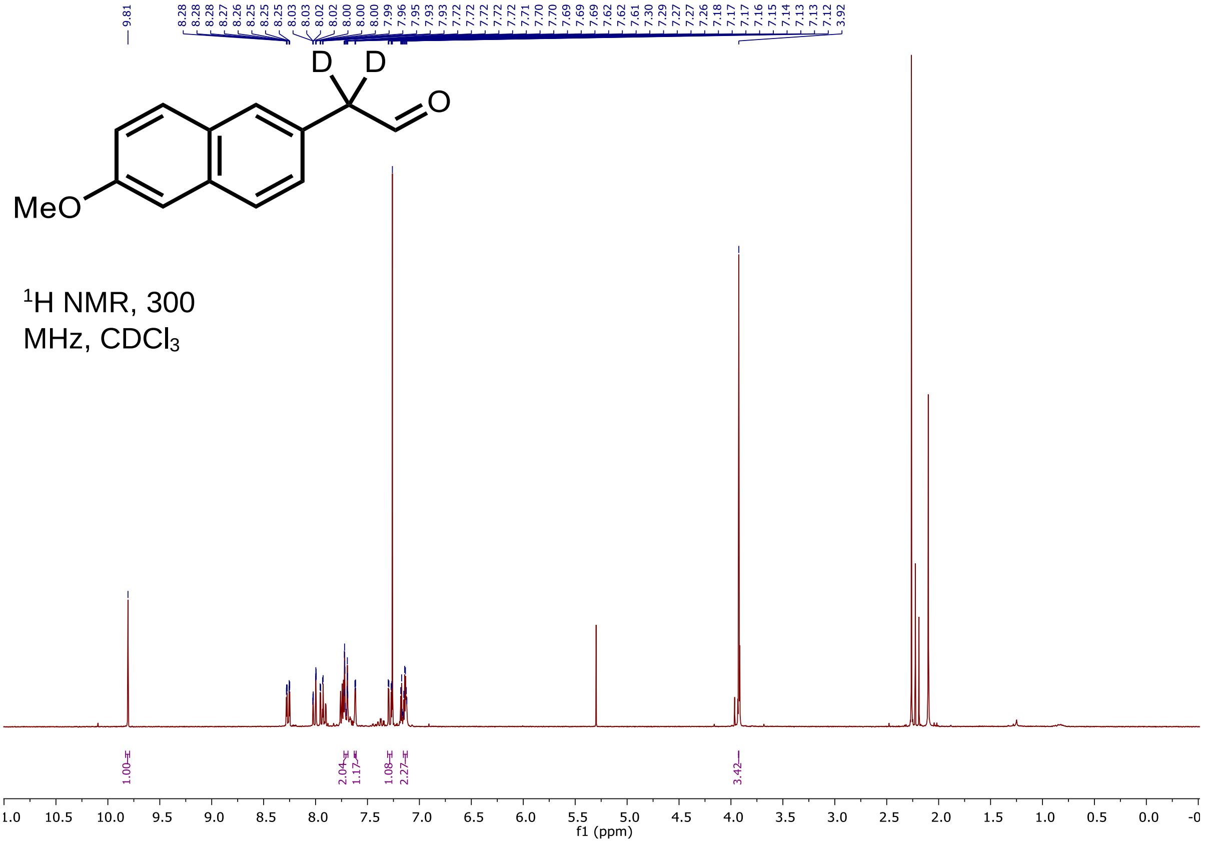
9.81
8.28 8.28 8.28 8.27 8.26 8.25 8.25 8.25 8.03 8.03 8.02 8.02 8.00 8.00 8.00 7.99 7.96 7.95 7.93 7.93 7.72 7.72 7.72 7.72 7.72 7.71 7.70 7.70 7.69 7.69 7.69 7.62 7.62 7.61 7.30 7.29 7.27 7.27 7.26 7.18 7.17 7.17 7.16 7.15 7.14 7.13 7.13 7.12 3.92
D D
O
MeO
1H NMR, 300 MHz, CDCl3
1.00
2.04
1.17
1.08
2.27
3.42
f1 (ppm)

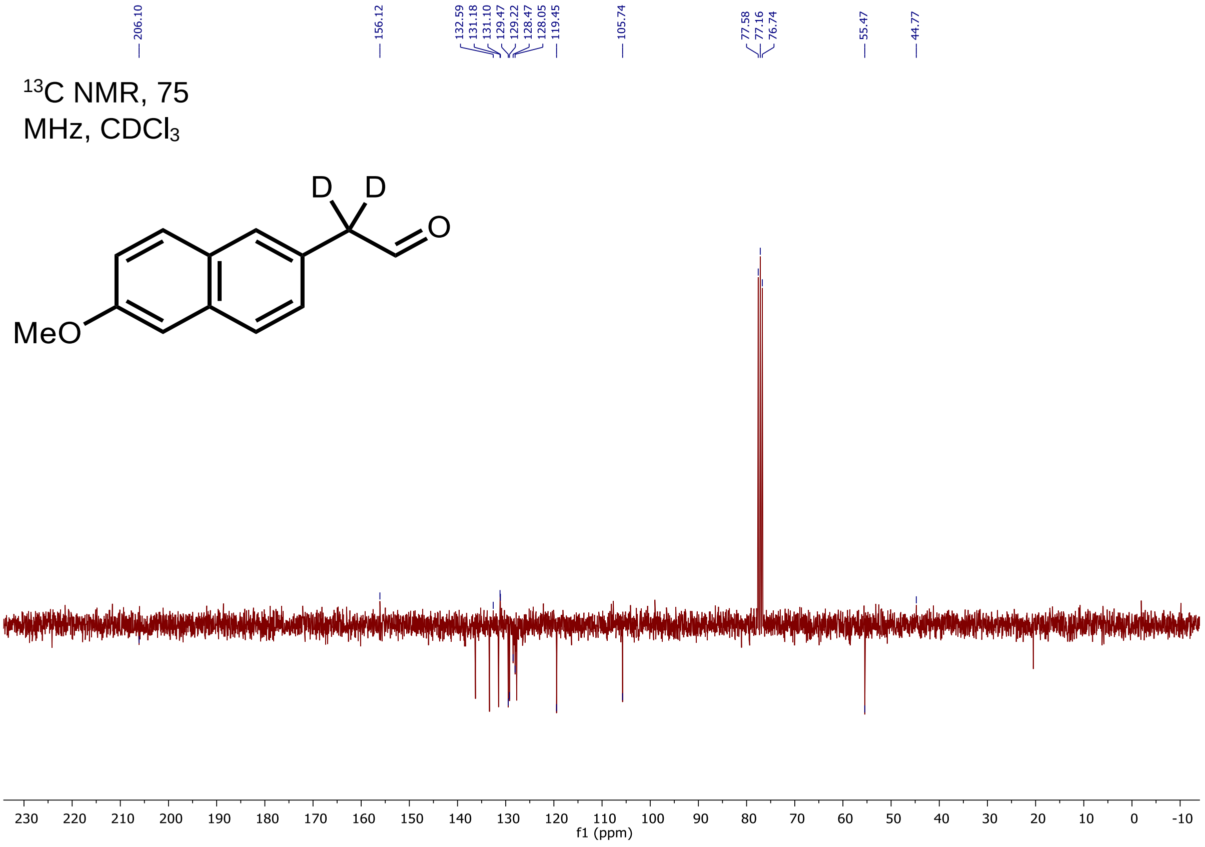
206.10
156.12
132.59 131.18 131.10 129.47 129.22 128.47 128.05 119.45
105.74
77.58 77.16 76.74
55.47
44.77
13C NMR, 75 MHz, CDCl3
D D
O
MeO
f1 (ppm)

2-(Allyl-1,1-d_2)-6-methoxynaphthalene (41)[21]

A solution of *n*-buLi (1.5 mmol, 2.5 M in hexane) was added to a stirred solution of methyltriphenylphosphonium bromide (0.54 g, 1.5 mmol) in dry THF (25 mL) under nitrogen. The solution was then cooled to 0 ˚C and crude 2-(6-methoxynaphthalen-2-yl)acetaldehyde-2,2-d_2 (0.20 g, 1.0 mmol) was added. The reaction was allowed to warm to rt and stirred for 12 h. Upon completion, 0.5 M HCl was added, and the mixture was extracted with diethyl ether then water, dried over $MgSO_4$, filtered, and concentrated *in vacuo*. Purification by flash silica chromatography (eluent = 10% EtOAc in hexanes) gave the title compound as pale yellow solid (0.04 g, 20% with 99% d_2-incorporation); mp 47–50 ˚C; R_f: 0.55 (eluent = 10% EtOAc in hexanes); ν_{max} / cm^{-1} (film) 3059, 3007, 2963, 2940, 2905, 2837, 1634, 1603, 1504, 1483, 1462, 1437, 1391, 1261, 1159, 1030; **^{1}H NMR (300 MHz, Chloroform-*d*)** δ 7.68 (d, *J* = 8.9 Hz, 2H), 7.56 (d, *J* = 1.6 Hz, 1H), 7.30 (dd, *J* = 8.4, 1.7 Hz, 1H), 7.16 – 7.09 (m, 2H), 6.03 (dd, *J* = 17.0, 10.1 Hz, 1H), 5.19 – 5.06 (m, 2H), 3.92 (s, 3H); **^{13}C NMR (75 MHz, Chloroform-*d*)** δ 155.1, 137.6, 136.2, 133.7, 129.1, 128.6, 128.0, 126.9, 126.7, 118.9, 116.0, 105.7, 55.4, 42.0; HRMS (CI^+) calculated $[C_{14}H_{12}D_2O]^+$ $(M)^+$: m/z 200.1180, found 200.1179.

^{1}H NMR, 300 MHz, $CDCl_3$

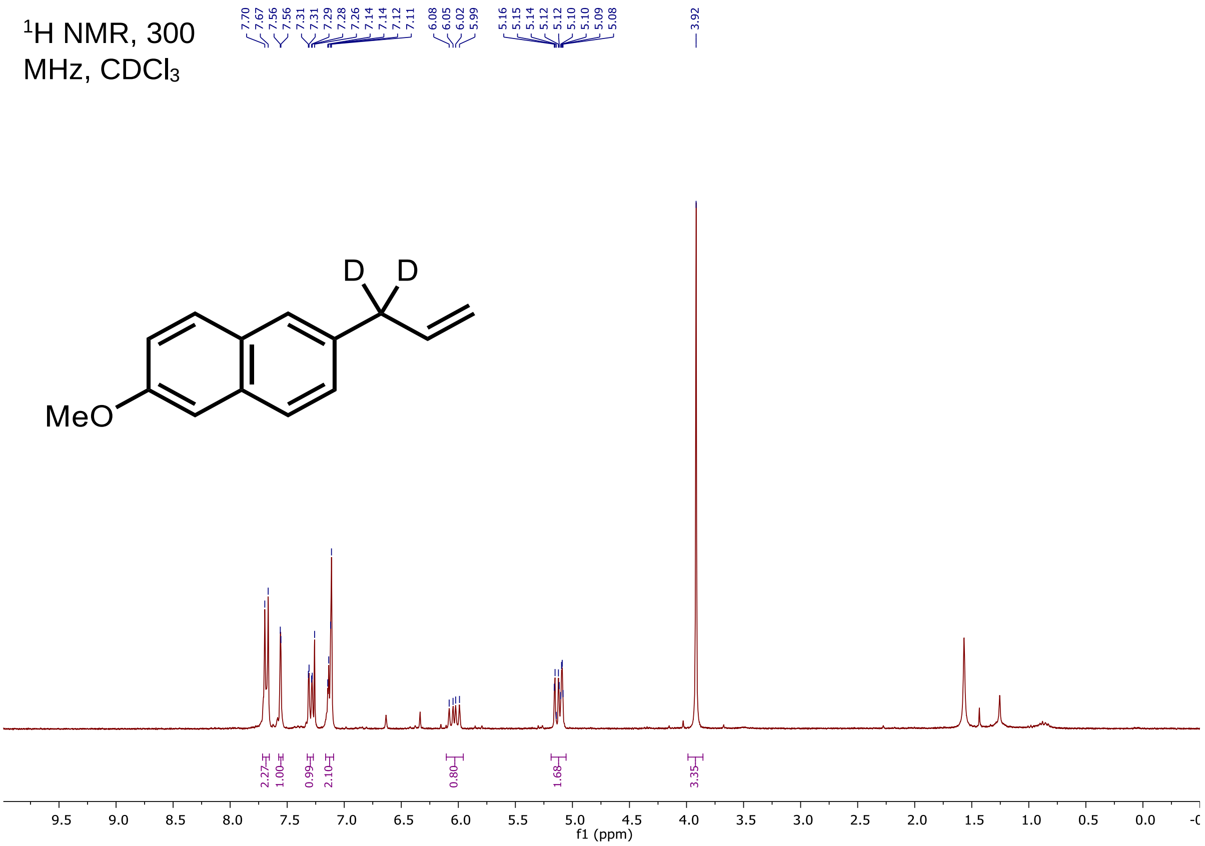

^{13}C NMR, 75 MHz, $CDCl_3$

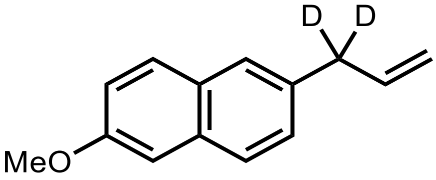

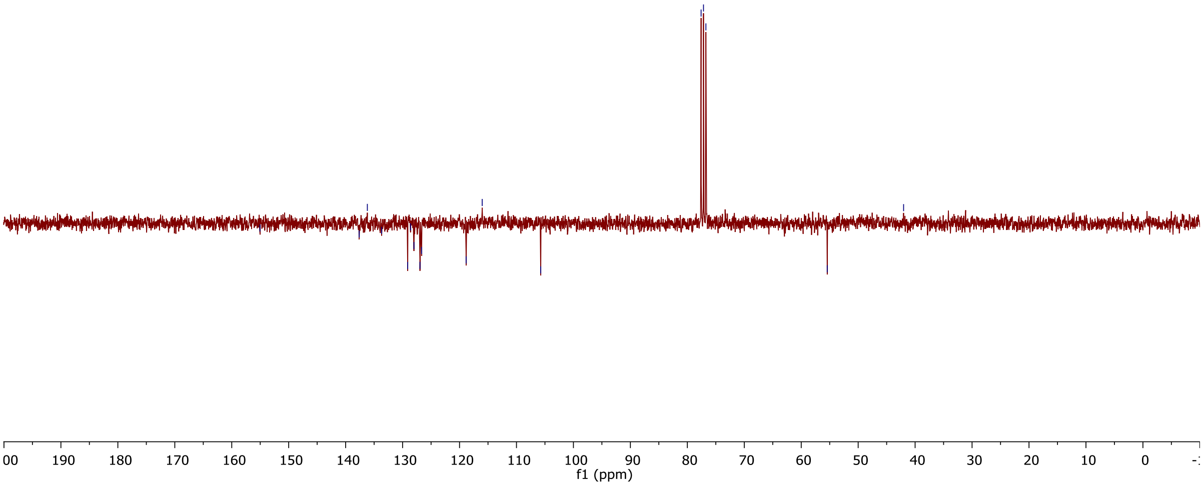

2.2. Optimization of the allyl isomerization

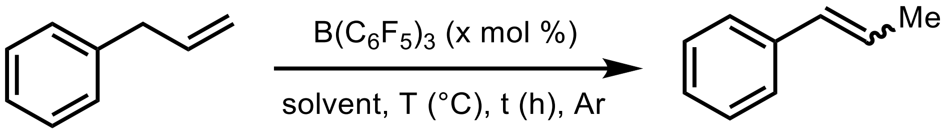

In the glovebox under Ar, an oven-dried 10 mL microwave vial equipped with a magnetic stirrer bar was charged with $B(C_6F_5)_3$ (x mol%), allyl benzene (0.2 mmol), and solvent (0.4 mL). The vial was sealed with an aluminium crimp cap and removed from the glovebox then stirred at the stated temperature (˚C) and time (h). It was then cooled to rt, 1,3,5-trimethylbenzene (30 μL, 0.2 mmol) and wet solvent (0.2 mL) were added. The mixture was stirred for 5 minutes and analysed using ^{1}H NMR.

Entry	SM (mmol)	BCF (mol%)	Solvent	T (˚C)	t (h)	*E*:*Z*:SM[a]	*E*:*Z*	Yield (%)[a]
1	0.2	10	toluene	100	24	0:0:>95	N.D	<2
2	0.2	10	xylene	100	24	0:0:>95	N.D	<2
3	0.2	10	toluene	140	24	9:<1:90	90:10	10
4	0.2	10	toluene	150	24	91:6:<1	94:6	97
5	0.2	10	xylene	150	24	88:6:6	94:6	94
6	0.2	10	anisole	150	24	58:2:23	97:3	60
7	0.2	10	bromobenzene	150	24	63:7:23	90:10	70
8	0.2	10	chlorobenzene	150	24	53:3:38	95:5	56
9	0.2	10	nitrobenzene	150	24	3:0.1:70	>98:<2	3
10	0.2	0	toluene	150	24	0:0:>95	N.D	<2
11	0.2	5	toluene	150	24	54:0:43	>98:<2	54
12	0.2	20	toluene	150	24	90:9:<1	91:9	>98
13	0.1 [0.25M]	10	toluene	150	24	33:<1:66	97:3	34
14	0.2	10	toluene	150	20	67:2:27	97:3	69
15	0.2	10	toluene	150	16	46:1:46	98:2	47
16	0.2	10	toluene	150	14	32:<1:66	98:2	33
17	0.2	10	toluene	150	12	24:<1:64	98:2	25
18	0.2	10	toluene	150	10	17:<1:83	98:2	18
19	0.2	10	toluene	150	6	10:<1:70	97:3	11
20	0.2	10	toluene	150	3	6:<1:84	97:3	7
21	0.2	10	toluene	150	1	3:0:>95	>98:<2	3

[a]determined by crude NMR yield with 1,3,5-trimethylbenzene as internal standard; all reactions were performed in **0.5 M** of substrate except otherwise stated; all reactions were repeated at least twice; SM = starting material; N.D. = not determined.

2.3. Substrate scope

2.3.1. General procedure 3

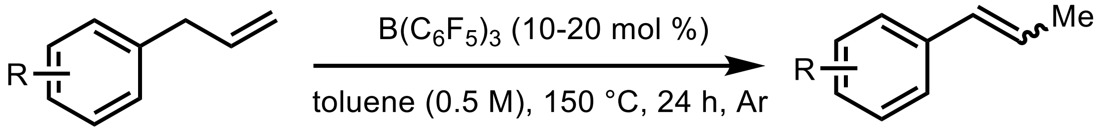

In the glovebox under Ar, an oven-dried 10 mL microwave vial equipped with a magnetic stirrer bar was charged with $B(C_6F_5)_3$ (10-20 mol %), allyl benzene (0.2 mmol), and toluene (0.4 mL). The vial was sealed with an aluminium crimp cap and stirred at 150 ˚C for 24 h. It was cooled to rt, 1,3,5-trimethylbenzene (30 μL, 0.2 mmol) was added and analysed using ^{1}H NMR. Purification was done by quenching with brine (0.4 mL). The organic phase was separated, dried over $MgSO_4$, filtered, and concentrated *in vacuo*.

2.3.2. Characterization of isomerization products

(*E*)-Prop-1-en-1-ylbenzene (2)[24]

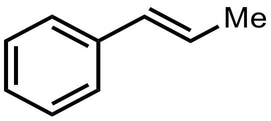

The title compound was prepared according to general procedure 3 using allyl benzene (0.2 mmol). Yield determined by crude ^{1}H NMR using 1,3,5-trimethylbenzene as internal standard: 97% (*E*:*Z* = 94:6).

Resolved signals of the major isomer (*E*)-prop-1-en-1-ylbenzene:

^{1}H NMR (500 MHz, Chloroform-*d*) δ 6.66 (dq, *J* = 15.7, 1.7 Hz, 1H), 6.48 (dq, *J* = 15.7, 6.6 Hz, 1H), 2.13 (dd, *J* = 6.6, 1.6 Hz, 3H).

Resolved signals of the minor isomer (*Z*)-prop-1-en-1-ylbenzene:[25]

^{1}H NMR (500 MHz, Chloroform-*d*) δ 6.9 (dq, *J* = 11.5, 1.9 Hz, 1H), 6.2 (dq, *J* = 11.6, 7.2 Hz, 1H), 2.3 (dd, *J* = 7.2, 1.9 Hz, 3H).

NMR yield determination:

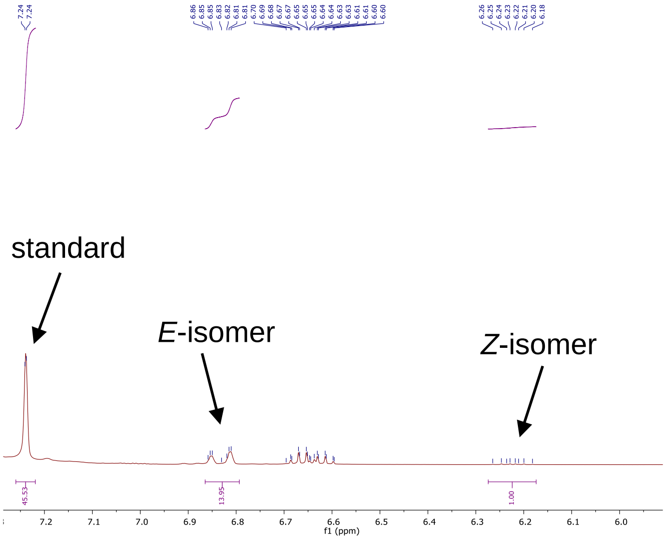

$$\%\ \text{yield} = \frac{cmp\ integral}{std\ integral} \times \frac{std\ proton}{cmp\ proton} \times 100$$

$$E\text{-isomer} = \frac{13.95}{45.53} \times \frac{3}{1} \times 100 = 91\%$$

$$Z\text{-isomer} = \frac{1.00}{45.53} \times \frac{3}{1} \times 100 = 6\%$$

cmp = compound

std = standard (1,3,5-trimethylbenzene)

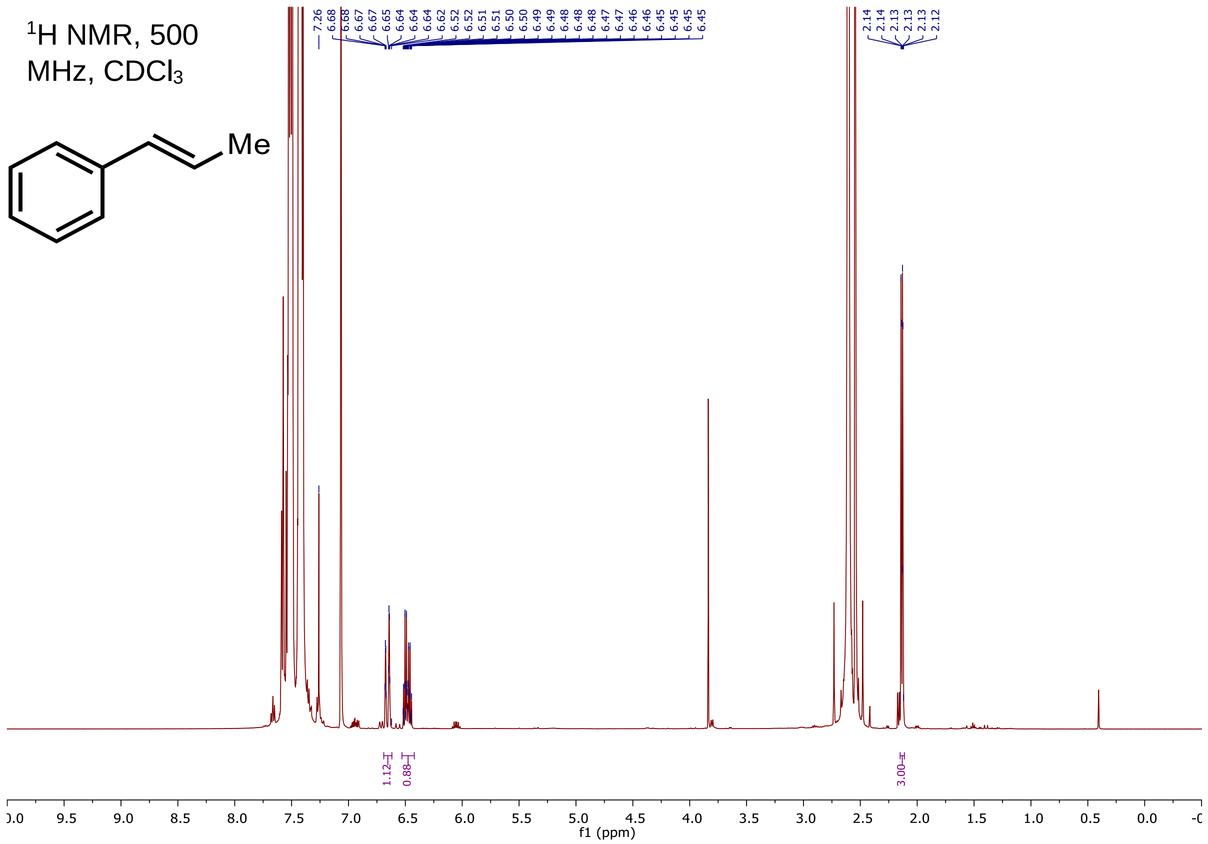

(*E*)-1-Methoxy-4-(prop-1-en-1-yl)benzene (3)[26]

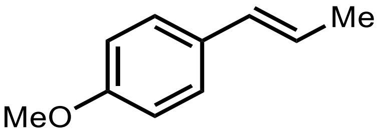

The title compound was prepared according to general procedure 3 using 4-allyl-methoxybenzene (0.2 mmol). Yield determined by crude ^{1}H NMR using 1,3,5-trimethylbenzene as internal standard: 98% (*E*:*Z* = 93:7).

<u>Resolved signals of the major isomer (*E*)-1-methoxy-4-(prop-1-en-1-yl)benzene:</u>

^{1}H NMR (500 MHz, Chloroform-*d*) δ 7.01 – 6.98 (m, 2H), 6.52 (dq, *J* = 15.7, 1.8 Hz, 1H), 6.25 (dq, *J* = 15.7, 6.6 Hz, 1H), 3.92 (s, 3H), 2.03 (dd, *J* = 6.6, 1.7 Hz, 3H).

<u>Resolved signals of the minor isomer (*Z*)-1-methoxy-4-(prop-1-en-1-yl)benzene:</u>[26]

^{1}H NMR (500 MHz, Chloroform-*d*) δ 7.05 – 7.03 (m, 2H), 5.92 – 5.84 (m, 1H), 3.94 (s, 3H), 2.07 (dd, *J* = 7.2, 1.8 Hz, 3H).

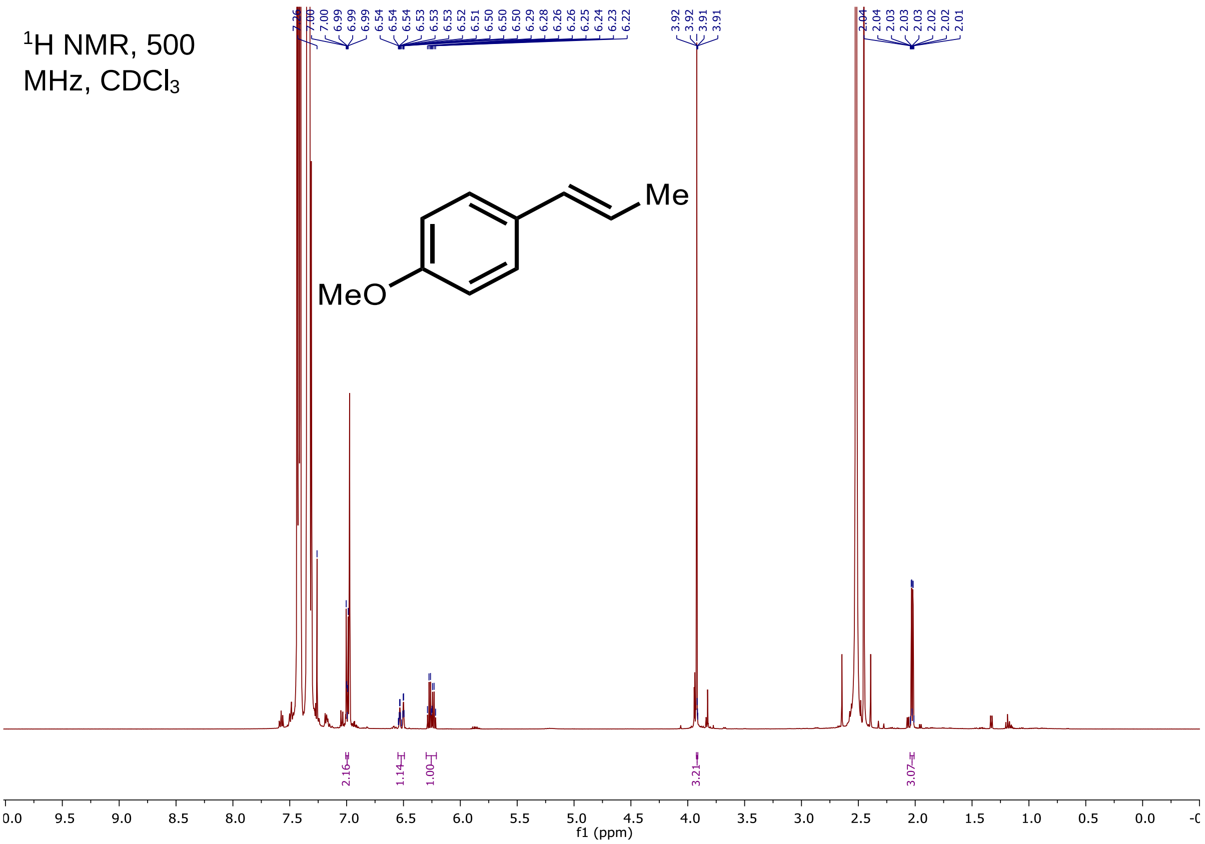

(*E*)-1-Phenoxy-4-(prop-1-en-1-yl)benzene (4)[27]

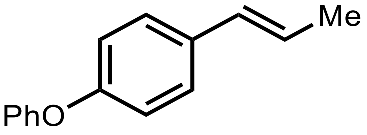

The title compound was prepared according to general procedure 3 using 1-allyl-4-(phenoxy)benzene (0.2 mmol). Purification by flash silica chromatography (eluent = 10% EtOAc in PE) gave the title compound as a pale yellow liquid (41 mg, 98%) as a mixture of *E*/*Z* products with grease impurity; R_f = 0.66 (eluent = 10% EtOAc in PE). NMR yield = >98% (*E*:*Z* = 93:7).

Signals of the major isomer (*E*)-1-phenoxy-4-(prop-1-en-1-yl)benzene:

^{1}H NMR (500 MHz, Chloroform-*d*) δ 7.36 – 7.29 (m, 4H), 7.10 (dq, *J* = 7.6, 1.0 Hz, 1H), 7.02 (dq, *J* = 7.5, 1.1 Hz, 2H), 6.97 – 6.94 (m, 2H), 6.39 (dd, *J* = 15.7, 1.8 Hz, 1H), 6.17 (dq, *J* = 15.7, 6.6, 0.9 Hz, 1H), 1.89 (dt, *J* = 6.6, 1.3 Hz, 3H).

^{13}C NMR (126 MHz, Chloroform-*d*) δ 157.5, 156.1, 133.5, 130.3, 129.8, 127.2, 124.9, 123.2, 119.2, 118.8, 18.6.

Resolved signals of the minor isomer (*Z*)-1-phenoxy-4-(prop-1-en-1-yl)benzene:[27]

^{1}H NMR (500 MHz, Chloroform-*d*) δ 5.77 (dq, *J* = 11.6, 7.2, 0.9 Hz, 1H), 1.92 (ddd, *J* = 7.2, 1.9, 0.9 Hz, 3H).

^{13}C NMR (126 MHz, Chloroform-*d*) δ 157.4, 130.3, 129.9, 123.3, 119.0, 118.7, 14.8.

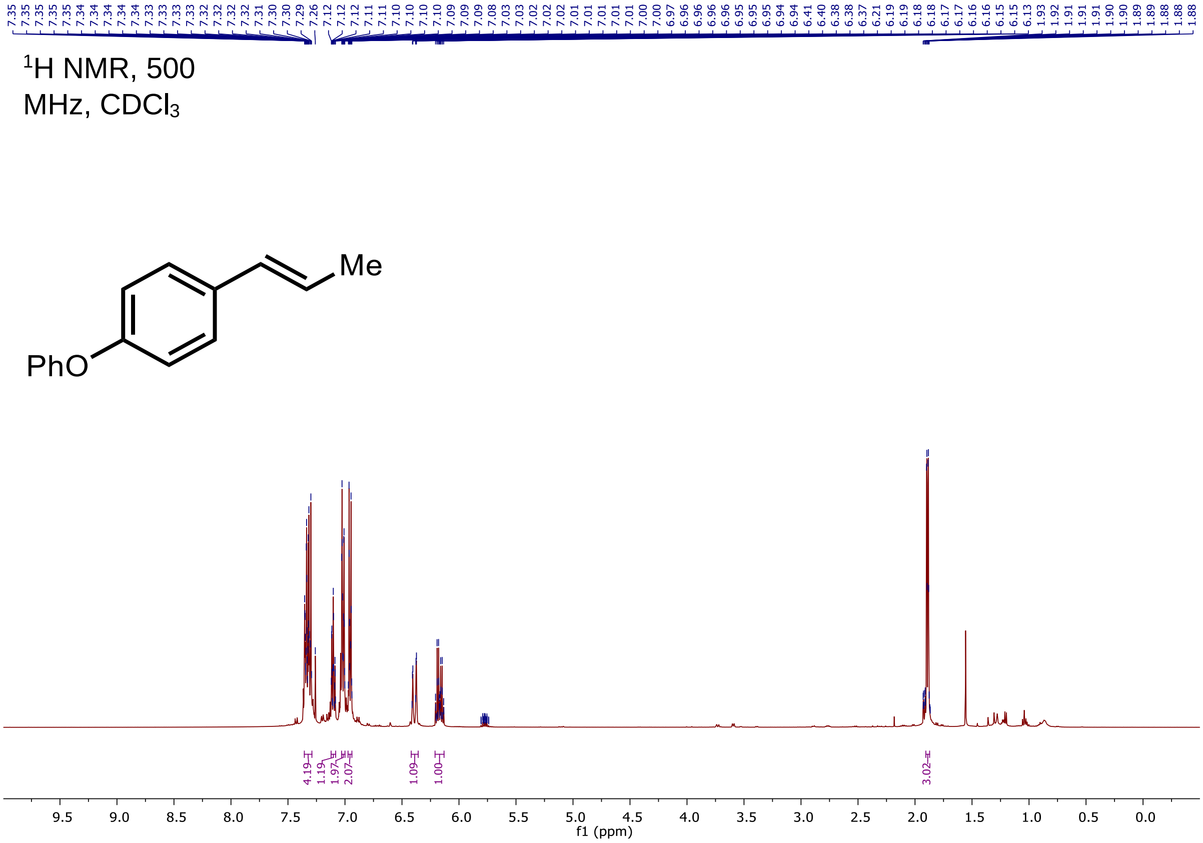

1H NMR, 500 MHz, CDCl3
Me
PhO
4.19
1.19
1.97
2.07
1.09
1.00
3.02
f1 (ppm)

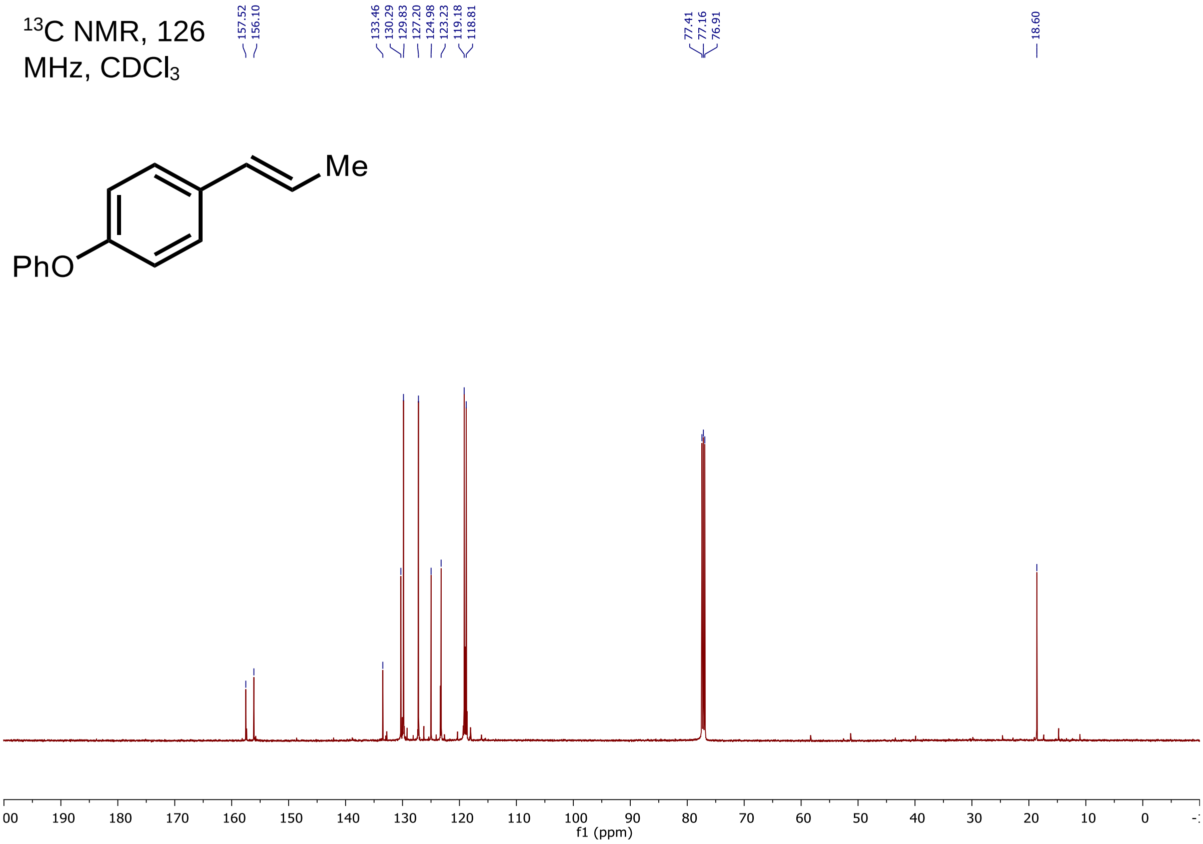

13C NMR, 126 MHz, CDCl3
157.52
156.10
133.46
130.29
129.83
127.20
124.98
123.23
119.18
118.81
77.41
77.16
76.91
18.60
Me
PhO
f1 (ppm)

(*E*)-Methyl(4-(prop-1-en-1-yl)phenyl)sulfane (5)[26]

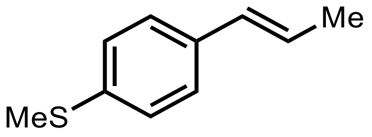

The title compound was prepared according to general procedure 3 using (4-(allylphenyl)methyl)sulfane (0.2 mmol). Yield determined by crude ^{1}H NMR using 1,3,5-trimethylbenzene as internal standard: 64% (*E*:*Z* = 95:5).

<u>Resolved signals of the major isomer (*E*)-methyl(4-prop-1-en-1-yl)phenyl)sulfane:</u>

^{1}H NMR (500 MHz, Chloroform-*d*) δ 6.74 (dq, *J* = 15.7, 1.8 Hz, 1H), 6.62 – 6.52 (m, 1H), 2.26 (dd, *J* = 6.6, 1.7 Hz, 3H).

<u>Resolved signals of the minor isomer (*Z*)-methyl(4-prop-1-en-1-yl)phenyl)sulfane:</u>[25]

^{1}H NMR (500 MHz, Chloroform-*d*) δ 6.80 (dq, *J* = 11.4, 1.8 Hz, 1H), 6.22 – 6.11 (m, 1H), 2.30 (dd, *J* = 7.5, 1.9 Hz, 3H).

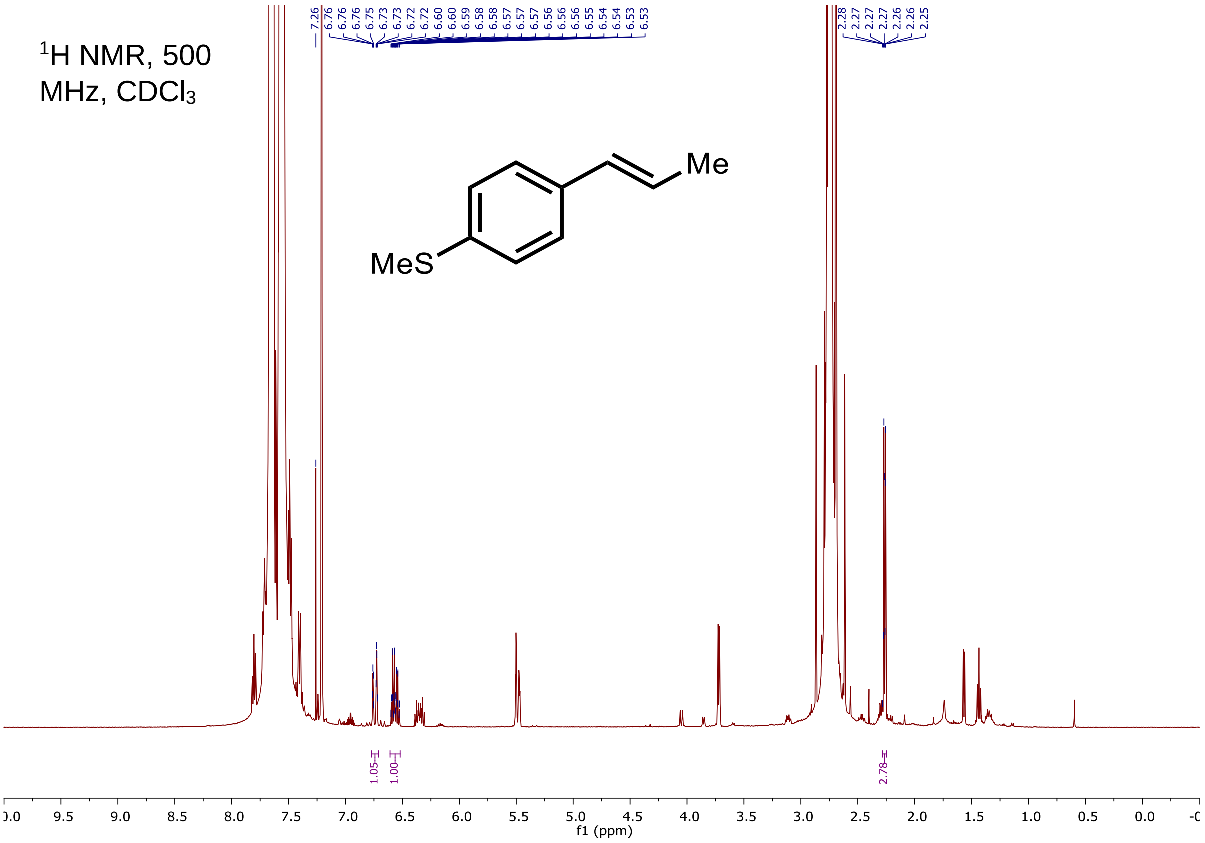

(*E*)-1-Methyl-4-(prop-1-en-1-yl)benzene (6)[26]

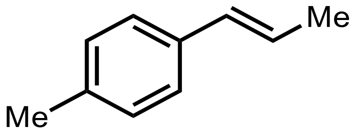

The title compound was prepared according to general procedure 3 using 4-allyl-methylbenzene (0.2 mmol). Yield determined by crude ^{1}H NMR using 1,3,5-trimethylbenzene as internal standard: 73% (*E*:*Z* = >98:<2).

<u>Resolved signals of the major isomer (*E*)-1-methyl-4-(prop-1-en-1-yl)benzene:</u>

^{1}H NMR (500 MHz, Chloroform-*d*) δ 6.85 – 6.78 (m, 1H), 6.59 (dq, *J* = 15.6, 6.6 Hz, 1H), 2.29 (dd, *J* = 6.7, 1.6 Hz, 3H).

<u>Resolved signals of the minor isomer (*Z*)-1-methyl-4-(prop-1-en-1-yl)benzene:</u>[28]

^{1}H NMR (500 MHz, Chloroform-*d*) δ 6.88 (d, *J* = 11.1 Hz, 1H), 6.18 (dd, *J* = 11.6, 7.1 Hz, 1H), 2.34 (dd, *J* = 7.2, 1.8 Hz, 3H).

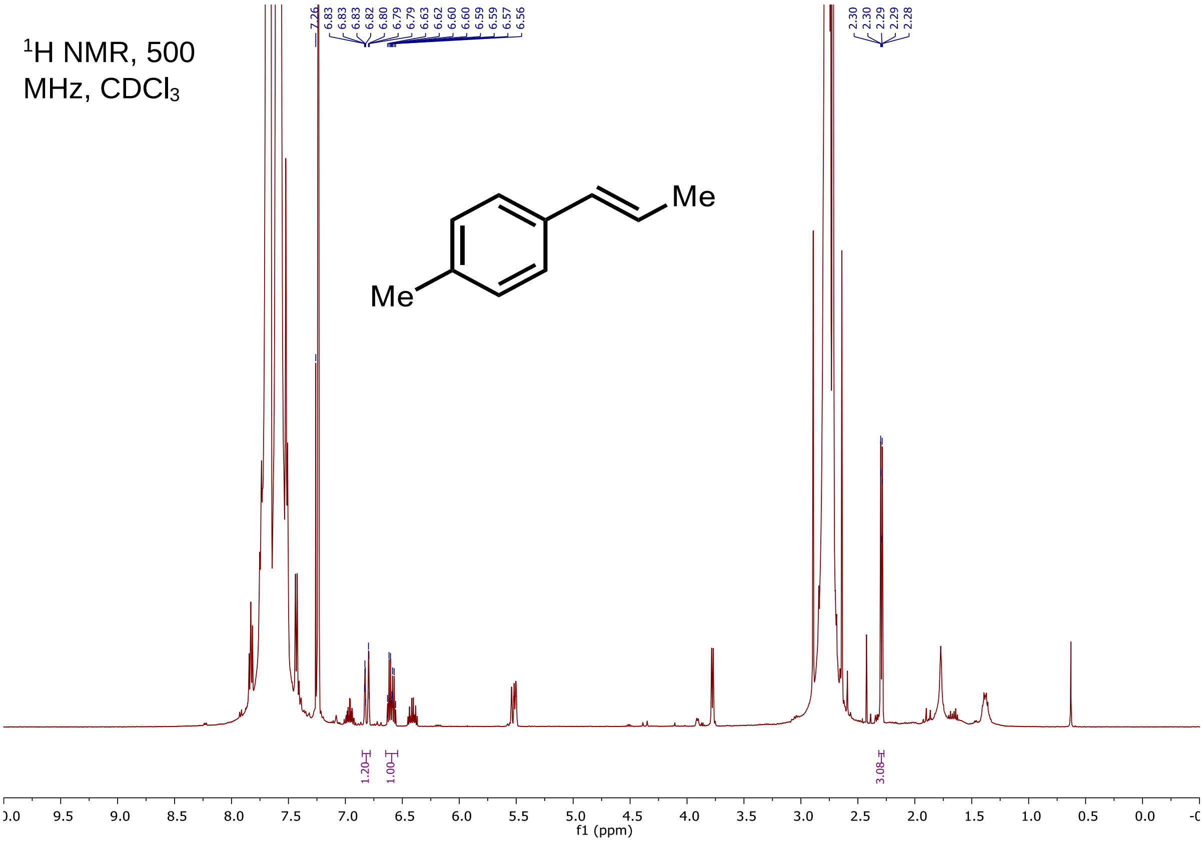

(*E*)-1-*tert*-butyl-4-(prop-1-en-1-yl)benzene (7)[29]

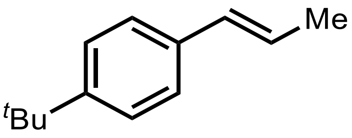

The title compound was prepared according to general procedure 3 using 1-allyl-4-(*tert*-butyl)benzene (0.2 mmol). Yield determined by crude ^{1}H NMR using 1,3,5-trimethylbenzene as internal standard: 91% (*E*:*Z* = 93:7).

<u>Signals of the major isomer (*E*)-1-(*tert*-butyl)-4-(prop-1-en-1-yl)benzene:</u>

^{1}H NMR (500 MHz, Chloroform-*d*) δ 7.36 – 7.34 (m, 2H), 7.33 – 7.30 (m, 2H), 6.40 – 6.35 (m, 1H), 6.19 (dq, J = 15.7, 6.6 Hz, 1H), 1.87 (dd, J = 6.6, 1.7 Hz, 3H), 1.31 (s, 9H).

^{13}C NMR (126 MHz, Chloroform-*d*) δ 149.8, 135.3, 130.8, 125.5, 125.4, 124.9, 34.6, 31.5, 18.7.

Resolved signals of the minor isomer (*Z*)-1-(tert-butyl)-4-(prop-1-en-1-yl)benzene:[30]

^{1}H NMR (500 MHz, Chloroform-*d*) δ 5.77 (ddd, *J* = 11.6, 7.2, 0.9 Hz, 1H), 1.94 (ddd, *J* = 7.2, 1.9, 0.9 Hz, 3H).

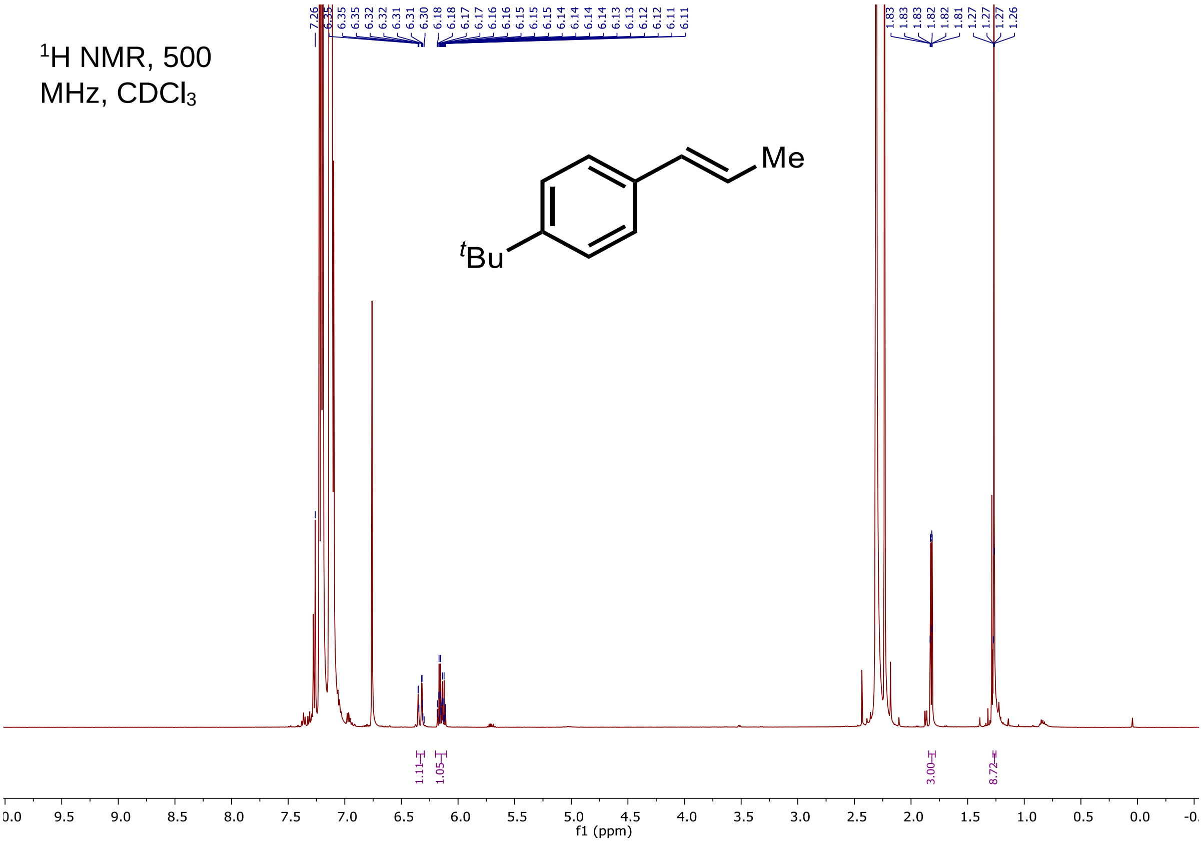

(*E*)-1-Nitro-4-(prop-1-en-1-yl)benzene (8)[31]

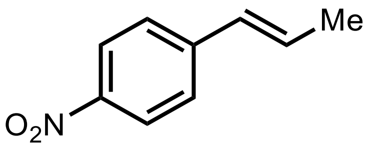

The title compound was prepared according to general procedure 3 using 1-allyl-4-nitrobenzene (0.2 mmol). Yield determined by crude ^{1}H NMR using 1,3,5-trimethylbenzene as internal standard: 81% (*E*:*Z* = 95:5).

Resolved signals of the major isomer (*E*)-1-nitro-4-(prop-1-en-1-yl)benzene:

^{1}H NMR (500 MHz, Chloroform-*d*) δ 8.41 – 8.37 (m, 2H), 6.67 – 6.60 (m, 2H), 2.21 (d, *J* = 5.3 Hz, 3H).

Resolved signals of the minor isomer (*Z*)-1-nitro-4-(prop-1-en-1-yl)benzene:[32]

^{1}H NMR (500 MHz, Chloroform-*d*) δ 8.46 – 8.43 (m, 2H), 6.31 – 6.25 (m, 1H).

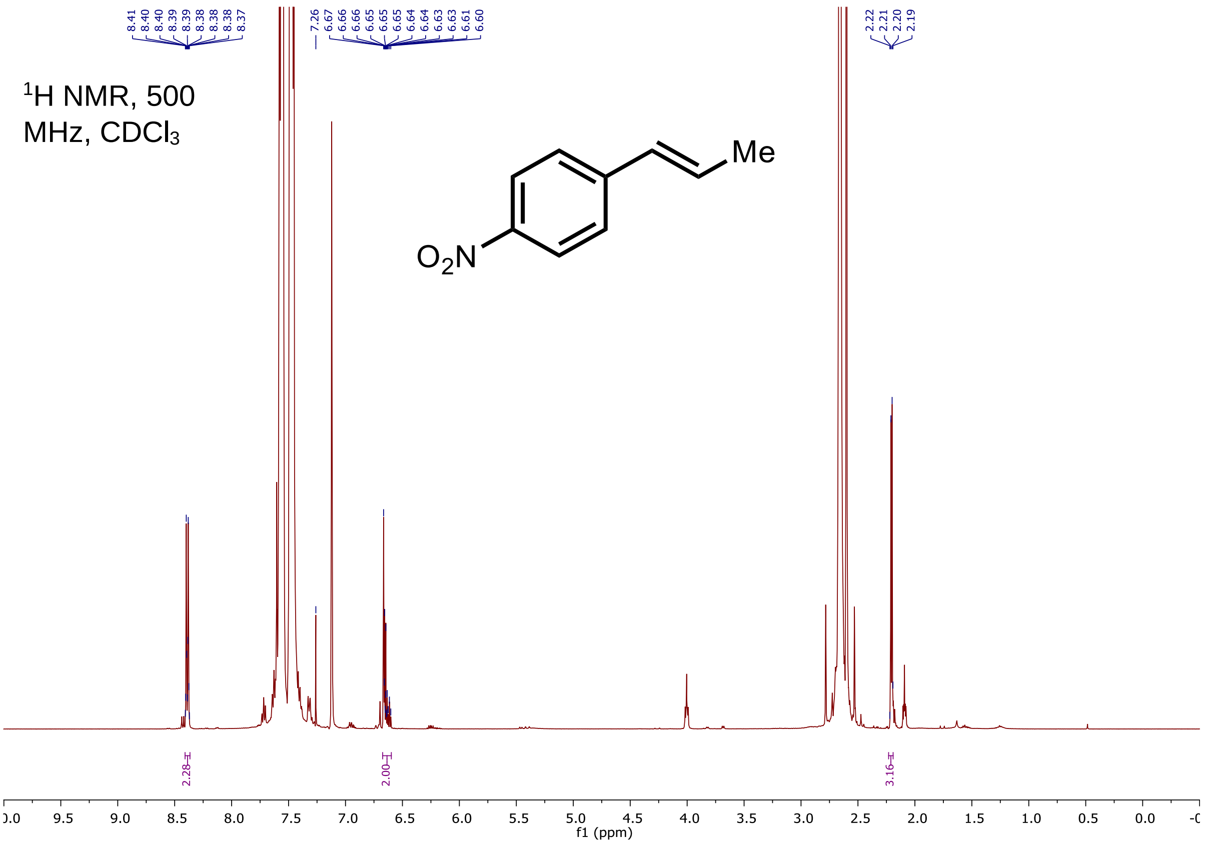

(*E*)-1-iodo-4-(prop-1-en-1-yl)benzene (9)[33]

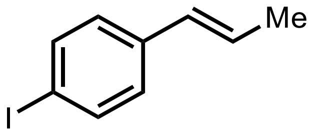

The title compound was prepared according to general procedure 3 using 1-allyl-4-iodobenzene (0.2 mmol). Purification by flash silica chromatography (eluent = 10% EtOAc in hexanes) gave the title compound as a white solid with grease impurity (44 mg, 91%). NMR yield = 94% (*E*:*Z* = 94:6).

<u>Signals of the minor isomer (*E*)-1-iodo-4-(prop-1-en-1-yl)benzene:</u>

^{1}H NMR (500 MHz, Chloroform-d) δ 7.62 – 7.57 (m, 2H), 7.08 – 7.04 (m, 2H), 6.32 (dd, *J* = 15.8, 1.5 Hz, 1H), 6.24 (dq, *J* = 15.7, 6.3 Hz, 1H), 1.87 (dd, *J* = 6.3, 1.4 Hz, 3H).

^{13}C NMR (126 MHz, Chloroform-d) δ 137.6, 137.5, 130.1, 127.8, 126.9, 91.8, 18.7.

<u>Resolved signals of the minor isomer (*Z*)-1-iodo-4-(prop-1-en-1-yl)benzene:</u>

^{1}H NMR (500 MHz, Chloroform-d) δ 5.82 (dq, *J* = 11.7, 7.3 Hz, 1H), 1.91 (dd, *J* = 6.6, 1.6 Hz, 3H).

^{13}C NMR (126 MHz, Chloroform-d) δ 14.3.

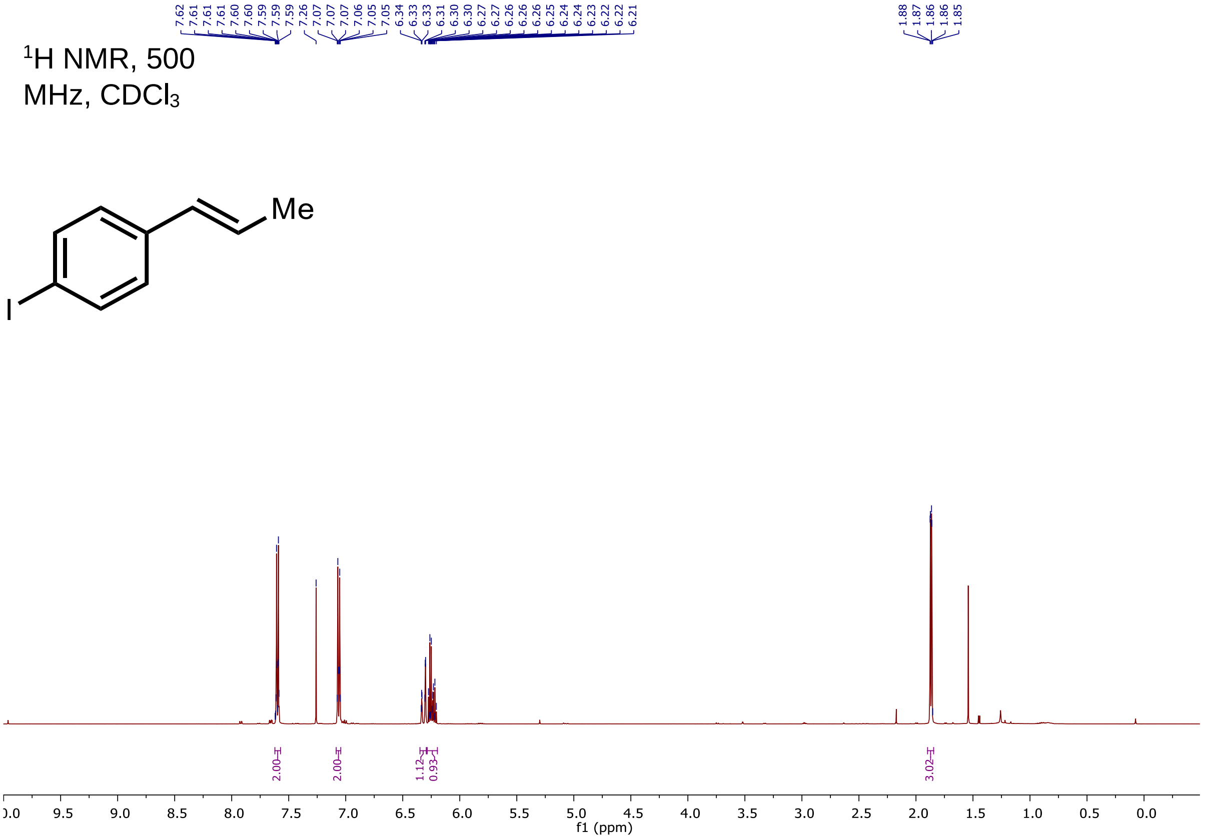

^{1}H NMR, 500 MHz, CDCl$_3$
Me
I
7.62 7.61 7.61 7.61 7.60 7.60 7.59 7.59 7.59 7.26 7.07 7.07 7.07 7.06 7.05 7.05 6.34 6.33 6.33 6.31 6.30 6.30 6.27 6.27 6.26 6.26 6.26 6.25 6.24 6.24 6.23 6.22 6.22 6.21
1.88 1.87 1.86 1.86 1.85
2.00 2.00 1.12 0.93 3.02
f1 (ppm)

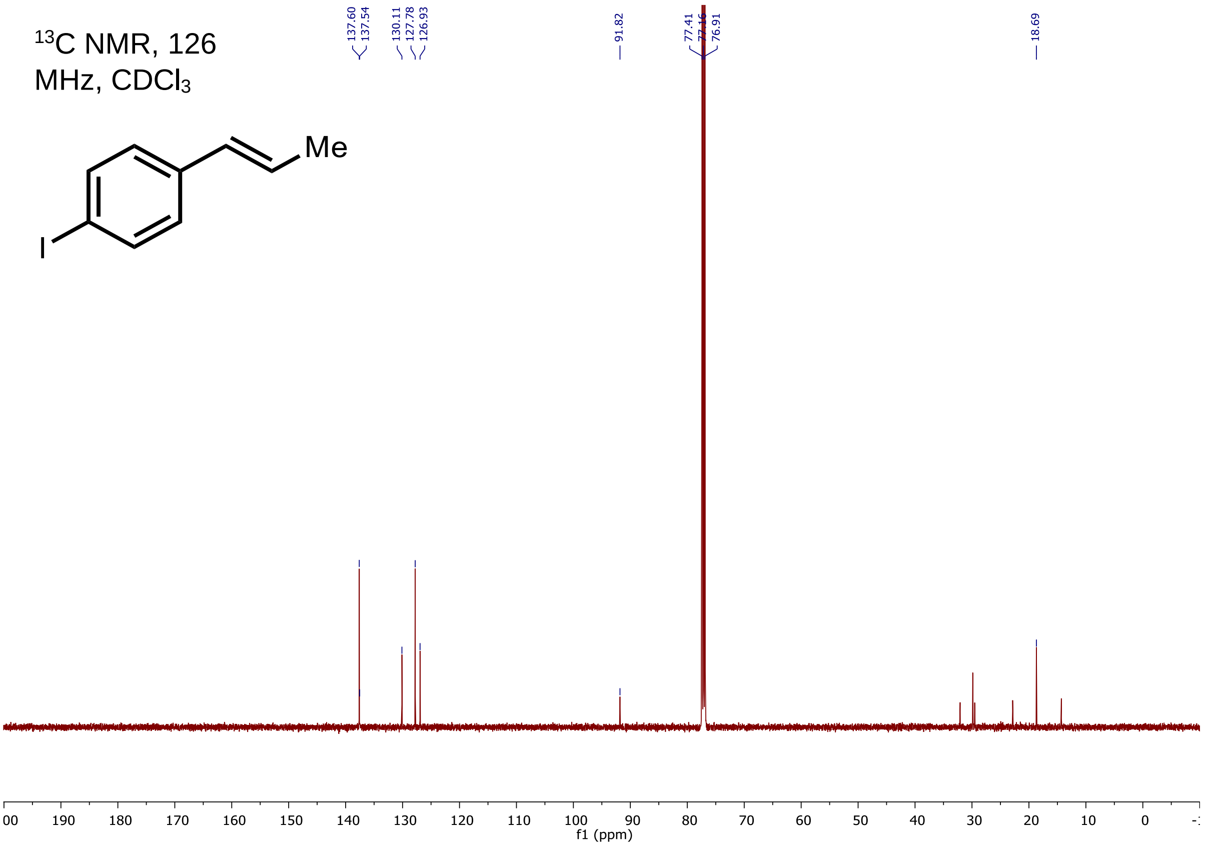

^{13}C NMR, 126 MHz, CDCl$_3$
Me
I
137.60 137.54 130.11 127.78 126.93
91.82
77.41 77.16 76.91
18.69
f1 (ppm)

(*E*)-1-bromo-4-(prop-1-en-1-yl)benzene (10)[29]

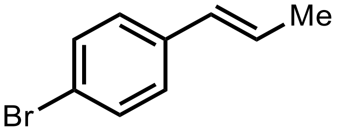

The title compound was prepared according to general procedure 3 using 1-allyl-4-bromobenzene (0.2 mmol). Purification by flash silica chromatography (eluent = 10% EtOAc in hexanes) gave the title compound as colourless liquid (25 mg, 63%) as a mixture of *E*/*Z* products and starting material with grease impurity. NMR yield = 91% (*E*:*Z* = 96:4).

Signals of the major isomer (*E*)-1-bromo-4-(prop-1-en-1-yl)benzene:

^{1}H NMR (500 MHz, Chloroform-d) δ 7.42 – 7.38 (m, 2H), 7.21 – 7.16 (m, 2H), 6.33 (dq, *J* = 15.7, 1.6 Hz, 1H), 6.23 (dq, *J* = 15.8, 6.5 Hz, 1H), 1.87 (dd, *J* = 6.5, 1.6 Hz, 3H).

^{13}C NMR (126 MHz, Chloroform-d) δ 136.9, 131.7, 130.1, 127.5, 126.8, 120.5, 18.7.

Resolved signals of the minor isomer (*Z*)-1-bromo-4-(prop-1-en-1-yl)benzene:[34]

^{1}H NMR (500 MHz, Chloroform-*d*) δ 5.85 – 5.79 (m, 1H).

^{13}C NMR (126 MHz, Chloroform-*d*) δ 14.3.

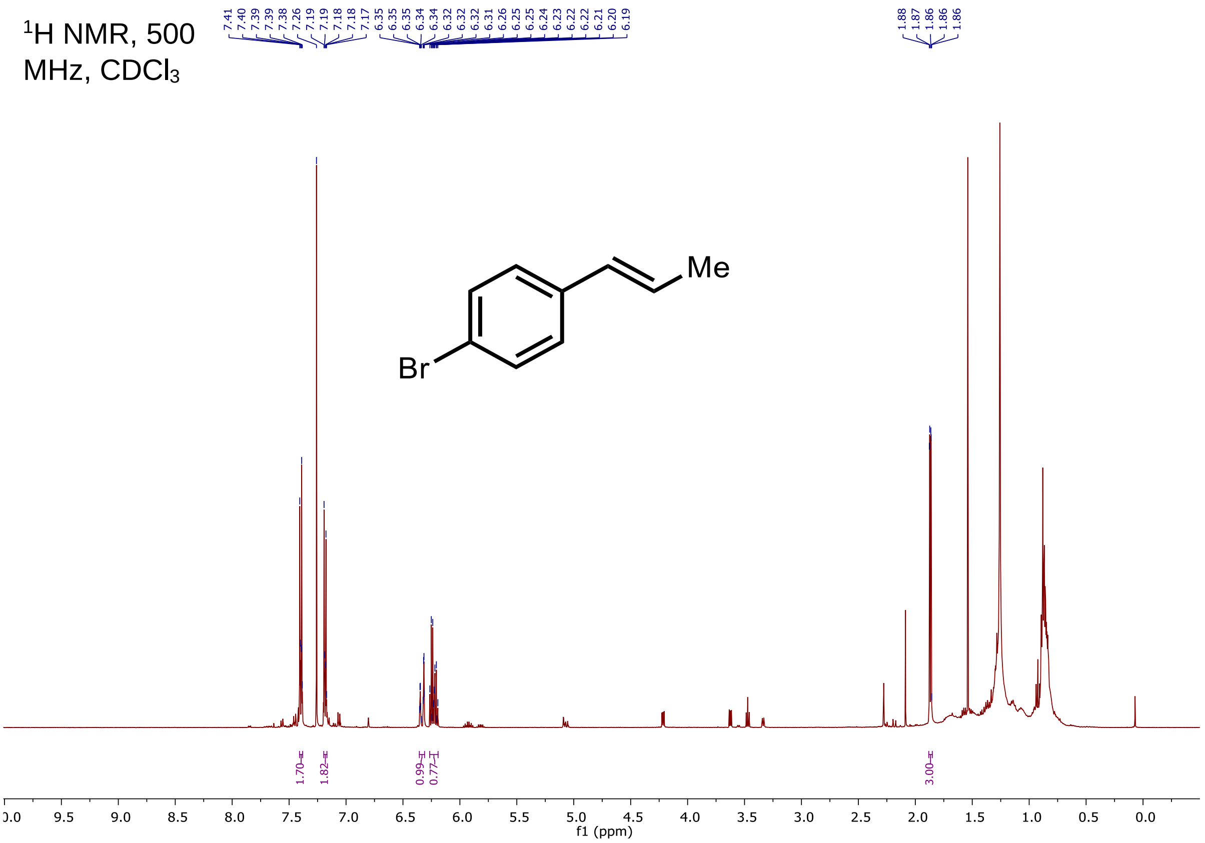

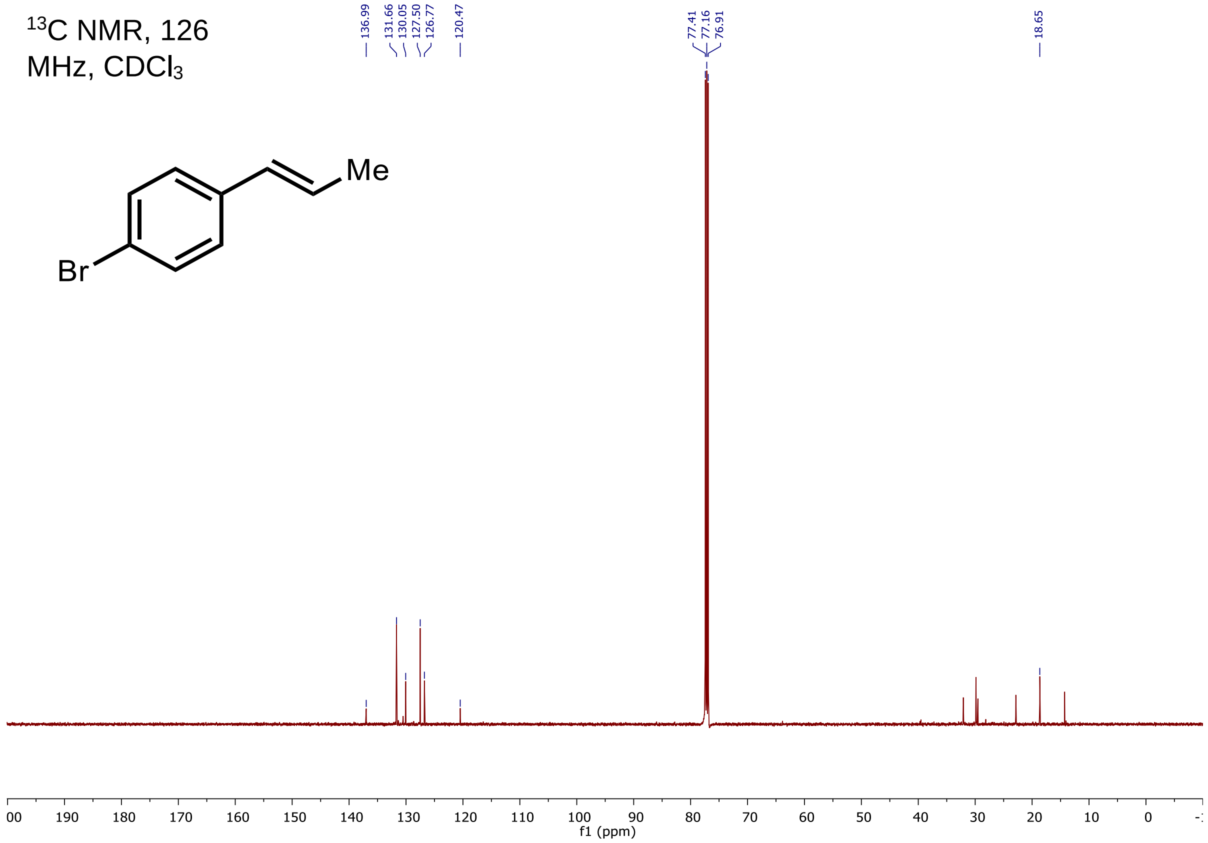

(*E*)-1-Fluoro-4-(prop-1-en-1-yl)benzene (11)[29]

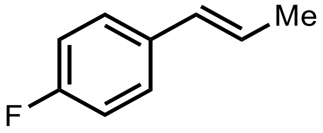

The title compound was prepared according to general procedure 3 using 1-allyl-4-fluorobenzene (0.2 mmol). Yield determined by crude ^{1}H NMR using 1,3,5-trimethylbenzene as internal standard: 78% (*E*:*Z* = >98:<2).

<u>Resolved signals of the major isomer (*E*)-1-fluoro-4-(prop-1-en-1-yl)benzene:</u>

^{1}H NMR (300 MHz, Chloroform-*d*) δ 6.70 (dq, *J* = 15.7, 1.8 Hz, 1H), 6.46 (dq, *J* = 15.1, 6.6, 1.4 Hz, 1H), 2.23 (dt, *J* = 6.6, 1.7 Hz, 3H).

<u>Resolved signals of the major isomer (*Z*)-1-fluoro-4-(prop-1-en-1-yl)benzene:</u>[25]

^{1}H NMR (300 MHz, Chloroform-*d*) δ 6.76 – 6.72 (m, 1H), 6.16 – 6.08 (m, 1H).

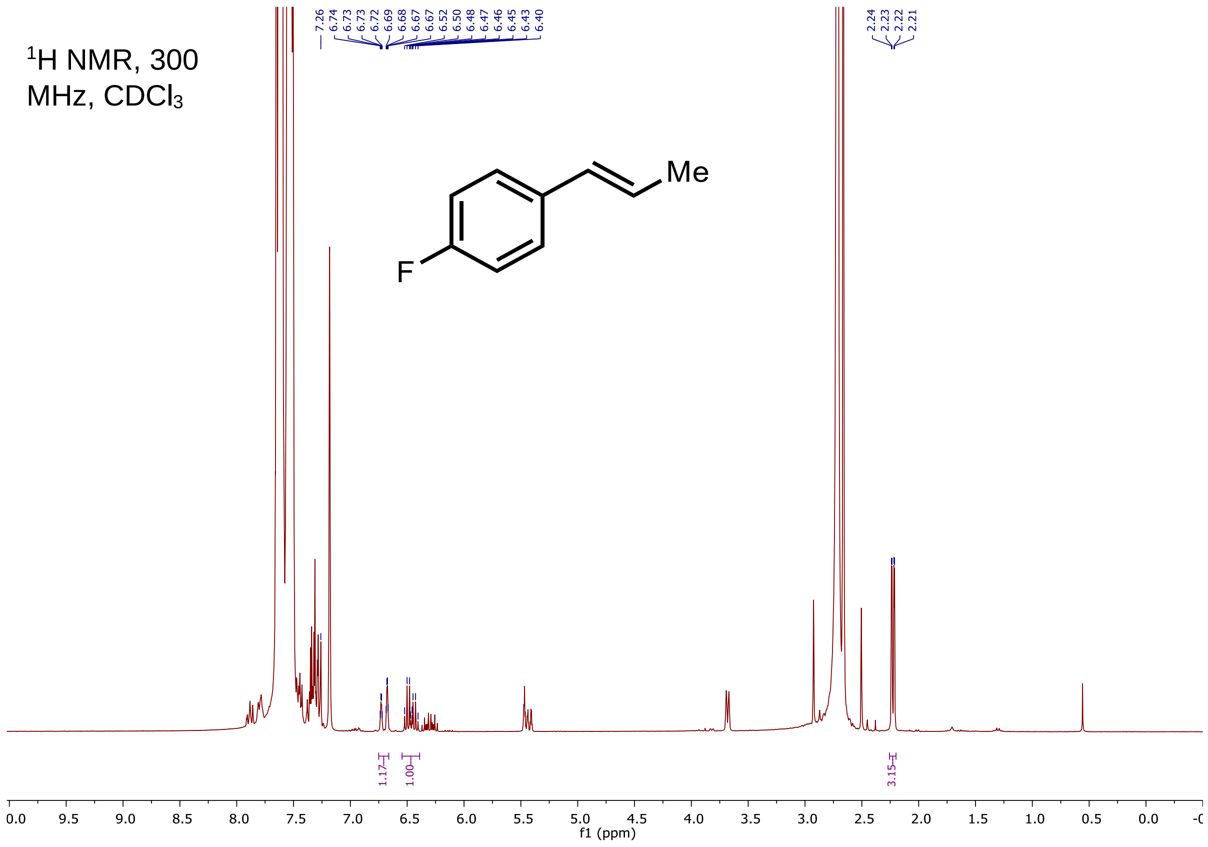

(*E*)-1-Methoxy-3-(prop-1-en-1-yl)benzene (12)[25,26]

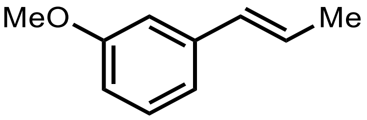

The title compound was prepared according to general procedure 3 using 3-allyl-methoxybenzene (0.2 mmol). Yield determined by crude ^{1}H NMR using 1,3,5-trimethylbenzene as internal standard: >98% (*E*:*Z* = 93:7).

<u>Resolved signals of the major isomer (*E*)-1-methoxy-3-(prop-1-en-1-yl)benzene:</u>

^{1}H NMR (500 MHz, Chloroform-*d*) δ 7.28 (dt, *J* = 7.7, 1.2 Hz, 1H), 7.24 (dd, *J* = 2.6, 1.6 Hz, 1H), 7.09 (ddd, *J* = 8.2, 2.6, 1.0 Hz, 1H), 6.72 (dq, *J* = 15.7, 1.7 Hz, 1H), 6.61 – 6.52 (m, 1H), 4.07 (d, *J* = 0.9 Hz, 3H), 2.22 (dt, *J* = 6.6, 1.5 Hz, 3H).

<u>Resolved signals of the minor isomer (*Z*)-1-methoxy-3-(prop-1-en-1-yl)benzene:</u>[25]

^{1}H NMR (500 MHz, Chloroform-*d*) δ 7.23 (d, *J* = 2.3 Hz, 1H), 7.14 – 7.11 (m, 1H), 6.79 (dq, *J* = 11.6, 1.9 Hz, 1H), 6.18 – 6.10 (m, 1H), 2.26 (dt, *J* = 7.2, 1.6 Hz, 3H).

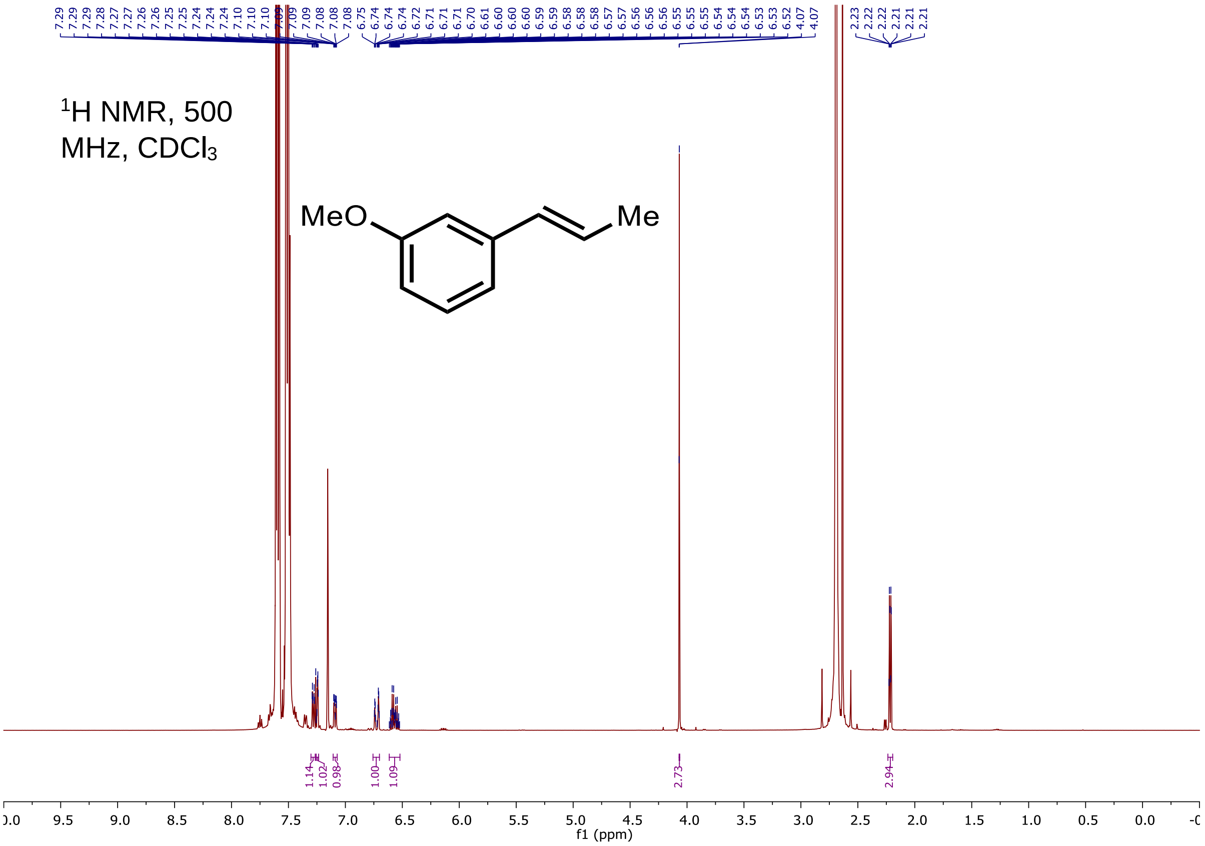

(*E*)-Methyl(3-(prop-1-en-1-yl)phenyl)sulfane (13)

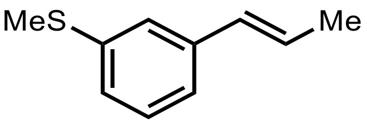

The title compound was prepared according to general procedure 3 using (3-(allylphenyl)methyl)sulfane (0.2 mmol). Purification by flash silica chromatography (eluent = 100% hexane) gave the title compound as a yellow liquid (21 mg, 64%) as a mixture of *E*/*Z* products and starting material with grease impurity. NMR yield = 86% (*E*:*Z* = 97:3).

<u>Signals of the major isomer (*E*)-methyl(3-(prop-1-en-1-yl)phenyl)sulfane:</u>

^{1}H NMR (500 MHz, Chloroform-*d*) δ 7.22 (t, *J* = 1.9 Hz, 1H), 7.13 – 7.07 (m, 3H), 6.39 – 6.34 (m, 1H), 6.25 (dq, *J* = 15.7, 6.5 Hz, 1H), 2.49 (s, 3H), 1.89 (dd, *J* = 6.5, 1.6 Hz, 3H).

^{13}C NMR (126 MHz, Chloroform-*d*) δ 138.6, 138.6, 130.6, 129.0, 126.6, 124.9, 124.1, 122.8, 18.7, 15.9.

IR (film, ν_{max} / cm^{-1}) 2920, 2851, 1699, 1587, 1497, 1437, 1198, 964.

<u>Resolved signals of the minor isomer (*Z*)-methyl(3-(prop-1-en-1-yl)phenyl)sulfane:</u>

^{1}H NMR (500 MHz, Chloroform-*d*) δ 2.73 (s, 3H), 1.92 (dd, *J* = 6.4, 1.4 Hz, 3H).

^{1}H NMR, 500 MHz, $CDCl_3$

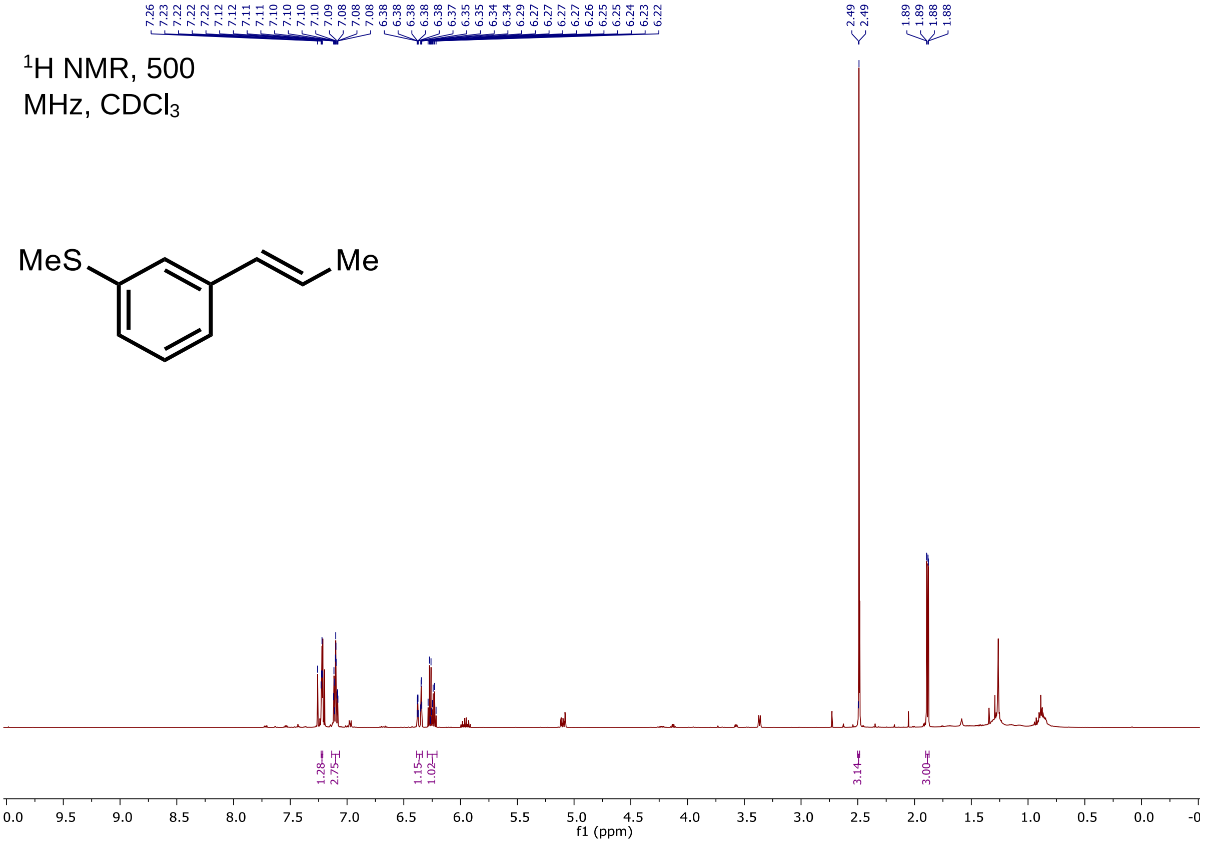

^{13}C NMR, 126 MHz, $CDCl_3$

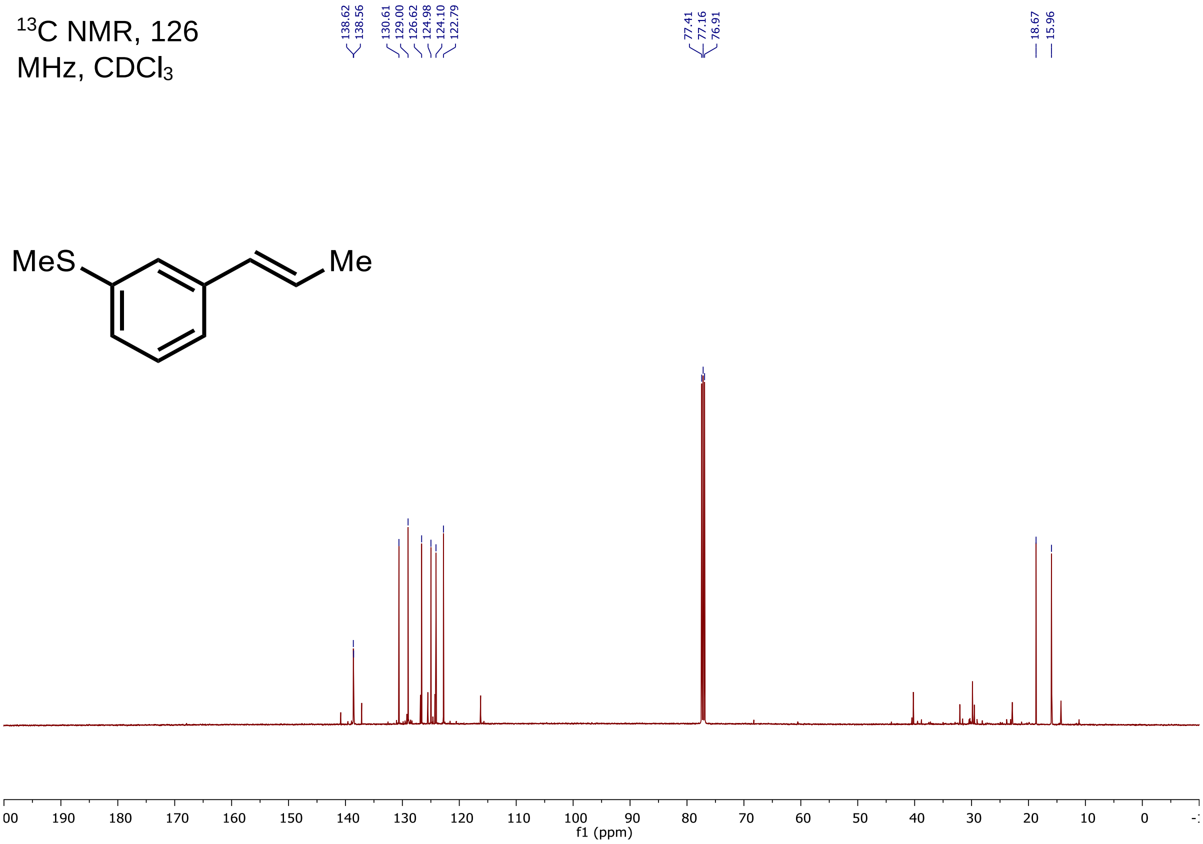

(*E*)-1-Methyl-3-(prop-1-en-1-yl)benzene (14)[26]

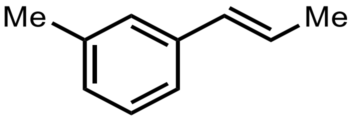

The title compound was prepared according to general procedure 3 using 2-allyl-methylbenzene (0.2 mmol). Yield determined by crude ^{1}H NMR using 1,3,5-trimethylbenzene as internal standard: 95% (*E*:*Z* = 94:6).

<u>Resolved signals of the major isomer (*E*)-1-methyl-3-(prop-1-en-1-yl)benzene:</u>

^{1}H NMR (500 MHz, Chloroform-*d*) δ 6.69 (dq, *J* = 15.8, 1.6 Hz, 1H), 6.56 – 6.48 (m, 1H), 2.18 (dt, *J* = 6.6, 1.4 Hz, 3H).

<u>Resolved signals of the minor isomer (*Z*)-1-methyl-3-(prop-1-en-1-yl)benzene:</u>[34]

^{1}H NMR (500 MHz, Chloroform-*d*) δ 6.77 – 6.72 (m, 1H), 6.08 (dq, *J* = 9.9, 7.2, 1.2 Hz, 1H), 2.22 (dq, *J* = 7.2, 1.6 Hz, 3H).

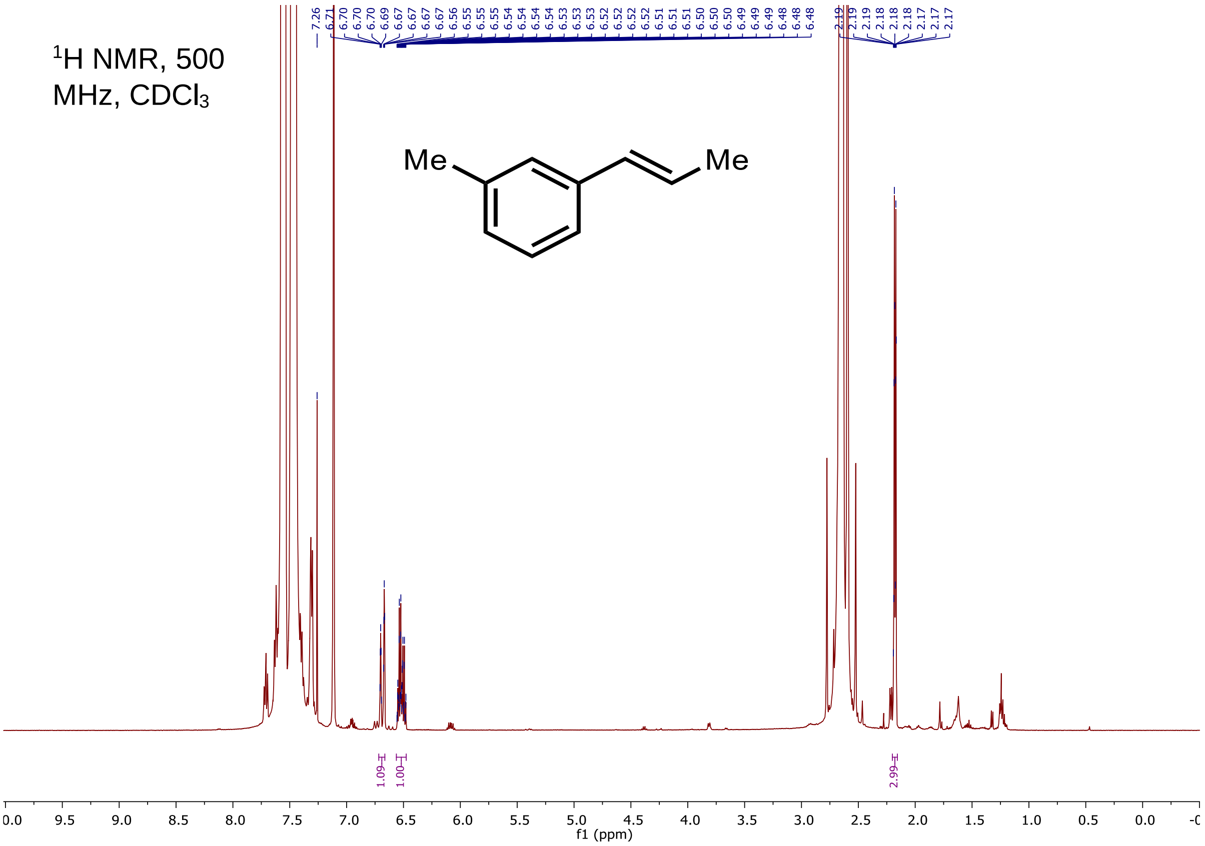

(*E*)-1-Nitro-3-(prop-1-en-1-yl)benzene (15)[31]

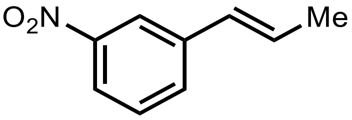

The title compound was prepared according to general procedure 3 using 1-allyl-3-nitrobenzene (0.2 mmol). Yield determined by crude ^{1}H NMR using 1,3,5-trimethylbenzene as internal standard: 75% (*E*:*Z* = 95:5).

<u>Resolved signals of the major isomer (*E*)-1-nitro-3-(prop-1-en-1-yl)benzene:</u>

^{1}H NMR (500 MHz, Chloroform-*d*) δ 8.43 (t, *J* = 2.0 Hz, 1H), 8.26 (ddd, *J* = 8.2, 2.3, 1.0 Hz, 1H), 7.77 (dt, *J* = 7.8, 1.4 Hz, 1H), 6.65 (dd, *J* = 15.8, 1.5 Hz, 1H), 6.58 (dq, *J* = 15.7, 6.2 Hz, 1H), 2.18 (dd, *J* = 6.2, 1.3 Hz, 3H).

<u>Resolved signals of the minor isomer (*Z*)-1-nitro-3-(prop-1-en-1-yl)benzene:</u>[35]

^{1}H NMR (500 MHz, Chloroform-*d*) δ 6.70 (dq, *J* = 11.5, 2.0 Hz, 1H).

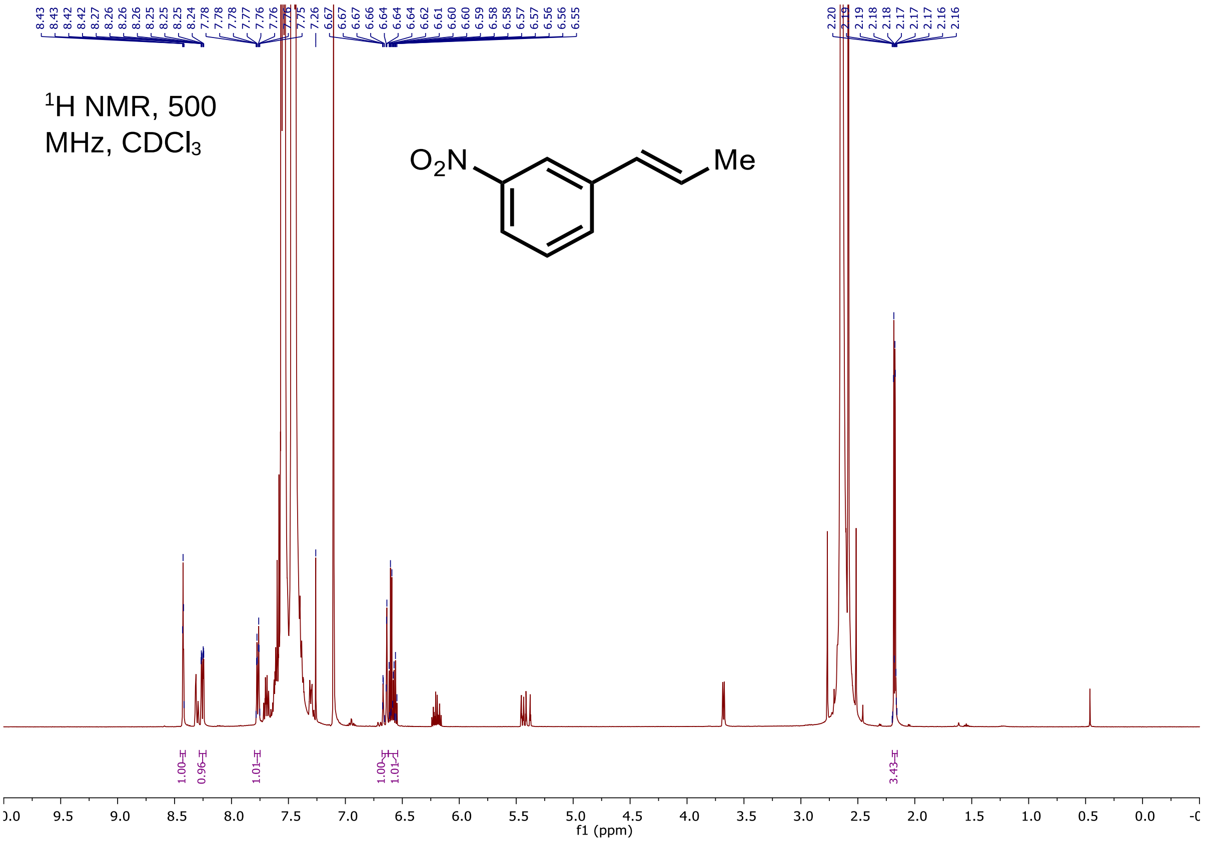

(*E*)-1-Methoxy-2-(prop-1-en-1-yl)benzene (16)[26]

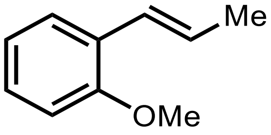

The title compound was prepared according to general procedure 3 using 2-allyl-methoxybenzene (0.2 mmol). Yield determined by crude ^{1}H NMR using 1,3,5-trimethylbenzene as internal standard: >98% (*E*:*Z* = 92:8).

<u>Resolved signals of the major isomer (*E*)-1-methoxy-2-(prop-1-en-1-yl)benzene:</u>

^{1}H NMR (500 MHz, Chloroform-*d*) δ 7.03 (q, *J* = 1.8 Hz, 1H), 6.49 (dq, *J* = 15.8, 6.6 Hz, 1H), 4.04 (s, 3H), 2.17 (dd, *J* = 6.6, 1.8 Hz, 3H).

Resolved signals of the minor isomer (*Z*)-1-methoxy-2-(prop-1-en-1-yl)benzene:[25]

^{1}H NMR (500 MHz, Chloroform-*d*) δ 6.59 (dt, *J* = 16.3, 1.6 Hz, 1H), 6.15 (dq, *J* = 11.5, 7.0 Hz, 1H), 4.08 (s, 3H), 2.13 (dd, *J* = 7.1, 1.9 Hz, 3H).

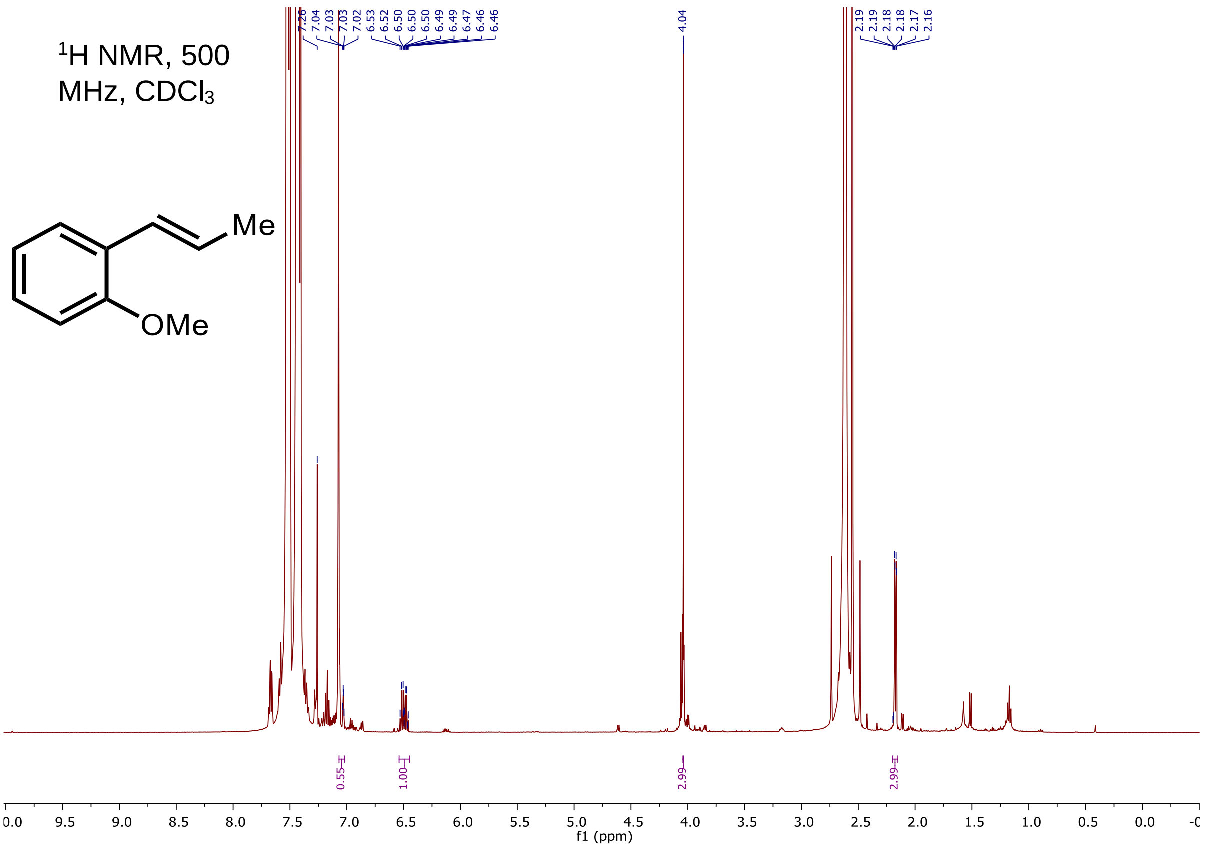

(*E*)-*tert*-butyldimethyl(2-(prop-1-en-1-yl)phenoxy)silane (17)[36]

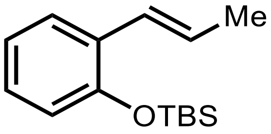

The title compound was prepared according to general procedure 3 using ((2-allylphenoxy)(*tert*-butyl)dimethylsilane (0.2 mmol). Purification by deactivated flash silica chromatography (eluent = 100% PE) gave the title compound as a colourless liquid (35.2 mg, 71%) as a mixture of *E*/*Z* products; R_f = 0.37 (eluent = 100% PE). NMR yield = 92% (*E*:*Z* = 90:10).

Signals of the major isomer (*E*)-*tert*-butyldimethyl(2-(prop-1-en-1-yl)phenoxy)silane:

^{1}H NMR (300 MHz, Chloroform-*d*) δ 7.42 (dd, *J* = 7.7, 1.8 Hz, 1H), 7.08 (ddd, *J* = 9.1, 7.4, 1.8 Hz, 1H), 6.94 – 6.87 (m, 1H), 6.78 (dd, *J* = 8.0, 1.3 Hz, 1H), 6.72 (dq, *J* = 15.9, 1.8 Hz, 1H), 6.16 (dq, *J* = 15.9, 6.6 Hz, 1H), 1.89 (dd, *J* = 6.6, 1.7 Hz, 3H), 1.04 (s, 9H), 0.21 (s, 6H).

^{13}C NMR (75 MHz, Chloroform-*d*) δ 152.4, 129.6, 127.6, 126.4, 126.2, 125.7, 121.5, 119.7, 25.9, 18.9, 18.5, -4.1.

Resolved signals of the minor isomer (*Z*)-*tert*-butyldimethyl(2-(prop-1-en-1-yl)phenoxy)silane:

^{1}H NMR (300 MHz, Chloroform-*d*) δ 5.79 (dq, *J* = 11.6, 7.0 Hz, 1H), 1.83 (dd, *J* = 7.1, 3H).

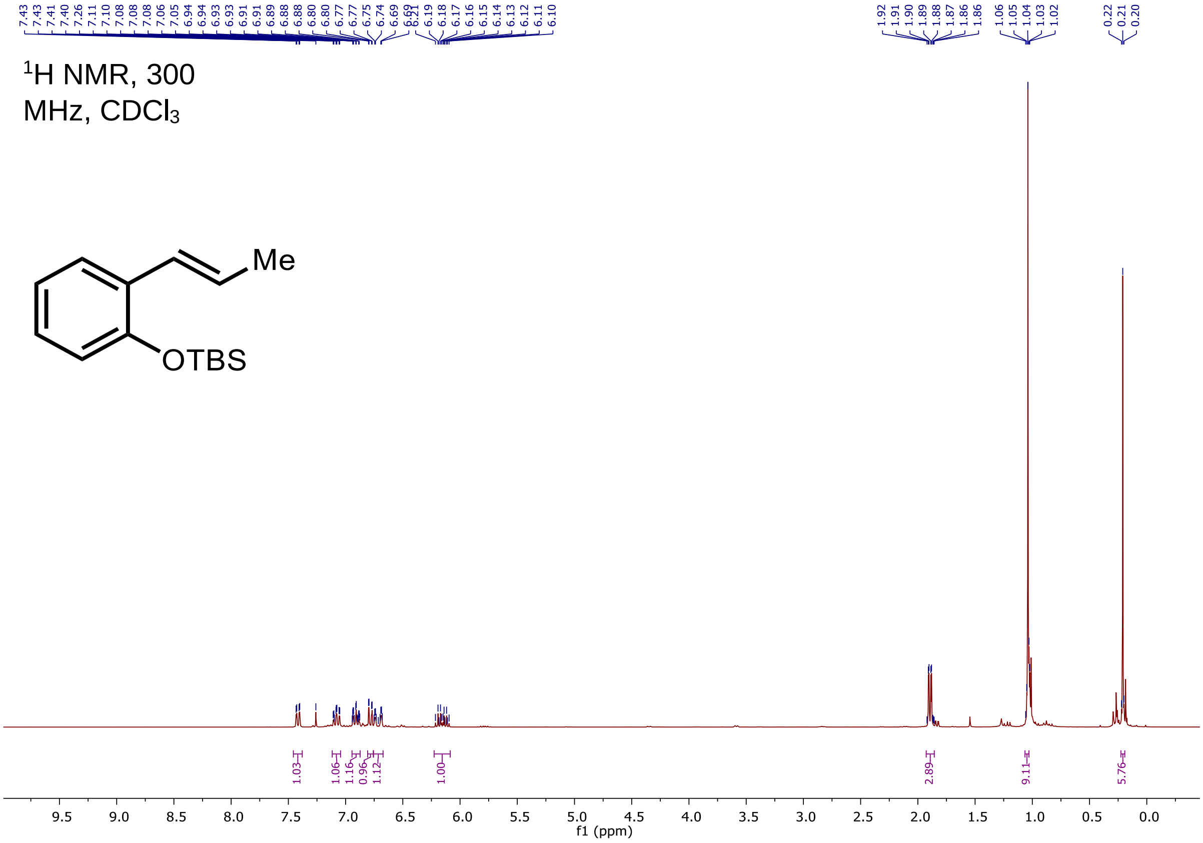

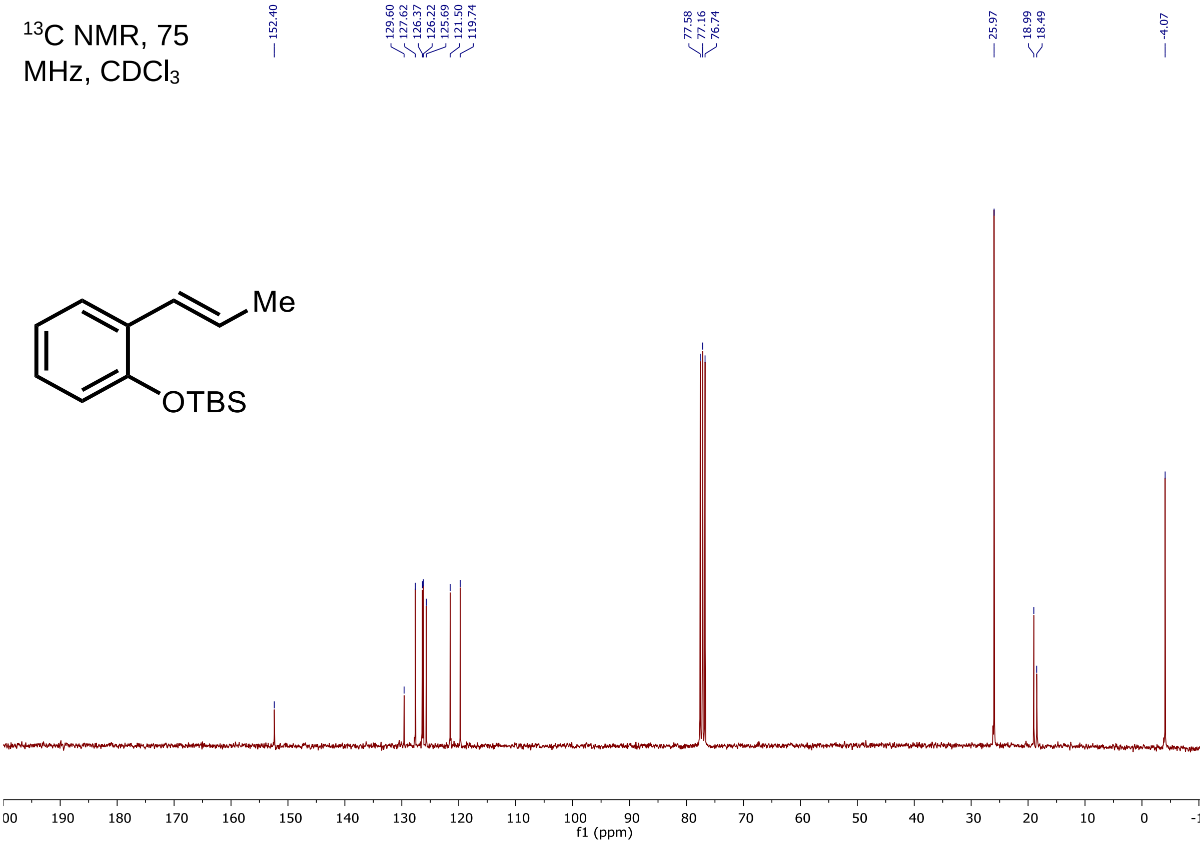

(*E*)-methyl(2-(prop-1-en-1-yl)phenyl)sulfane (18)[37]

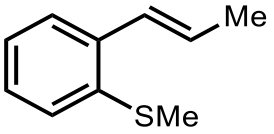

The title compound was prepared according to general procedure 3 using (2-(allylphenyl)methyl)sulfane (0.2 mmol). Purification by flash silica chromatography (eluent = 5% EtOAc in PE) gave the title compound as a yellow liquid (13 mg, 40%) as a mixture of *E*/*Z* products and starting material with grease impurity; R_f = 0.57 (eluent = 5% EtOAc in PE). NMR yield = 63% (*E*:*Z* = 90:10).

<u>Signals of the major isomer (*E*)-methyl(2-(prop-1-en-1-yl)phenyl)sulfane:</u>

^{1}H NMR (300 MHz, Chloroform-*d*) δ 7.39 (dd, *J* = 7.4, 1.5 Hz, 1H), 7.23 – 7.11 (m, 3H), 6.79 (dq, *J* = 15.5, 1.8 Hz, 1H), 6.16 (dq, *J* = 15.6, 6.6 Hz, 1H), 2.45 (s, 3H), 1.93 (dd, *J* = 6.6, 1.8 Hz, 3H).

^{13}C NMR (75 MHz, Chloroform-*d*) δ 137.3, 128.4, 128.3, 127.4, 126.6, 126.1, 125.5, 18.9, 16.4.

<u>Resolved signals of the minor isomer (*Z*)-methyl(2-(prop-1-en-1-yl)phenyl)sulfane:</u>[37]

^{1}H NMR (300 MHz, Chloroform-*d*) δ 6.53 (d, *J* = 11.0 Hz, 1H), 1.78 (dd, *J* = 7.0, 1.8 Hz, 3H).

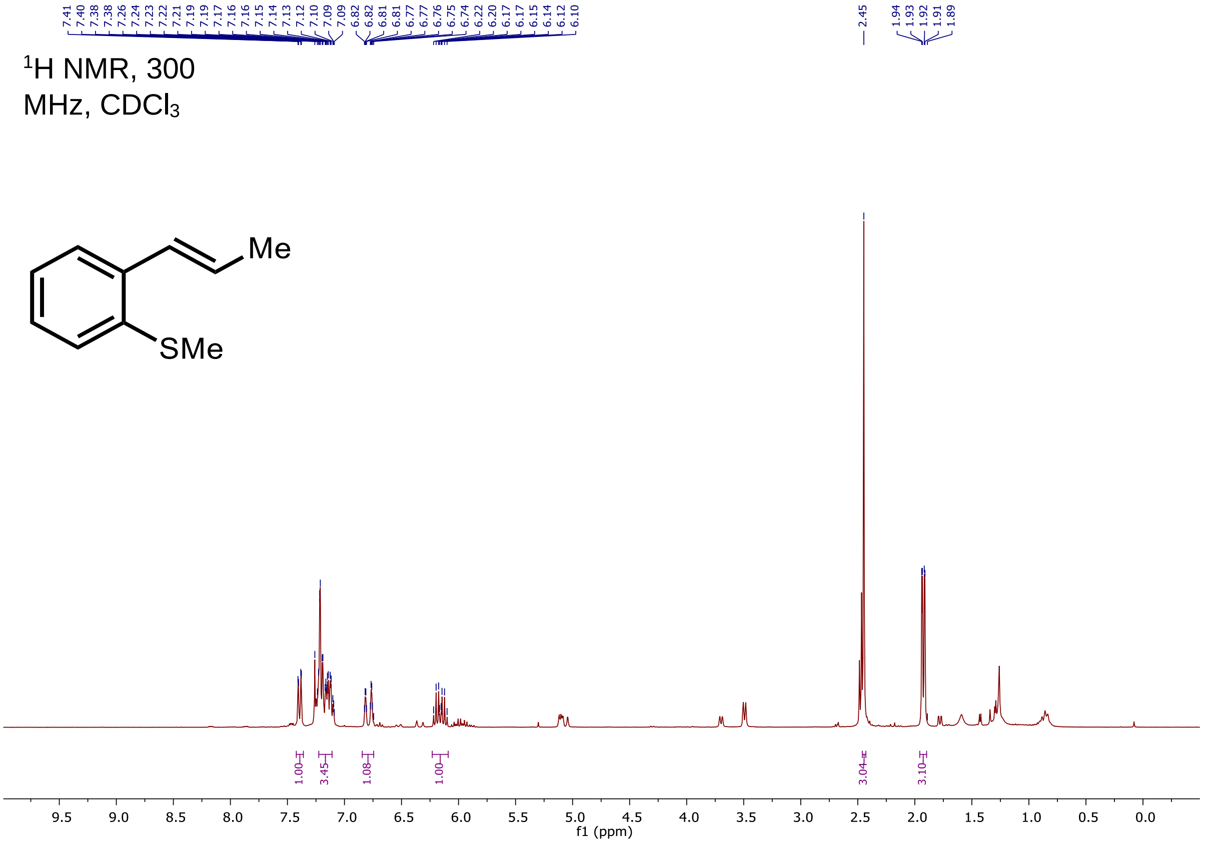

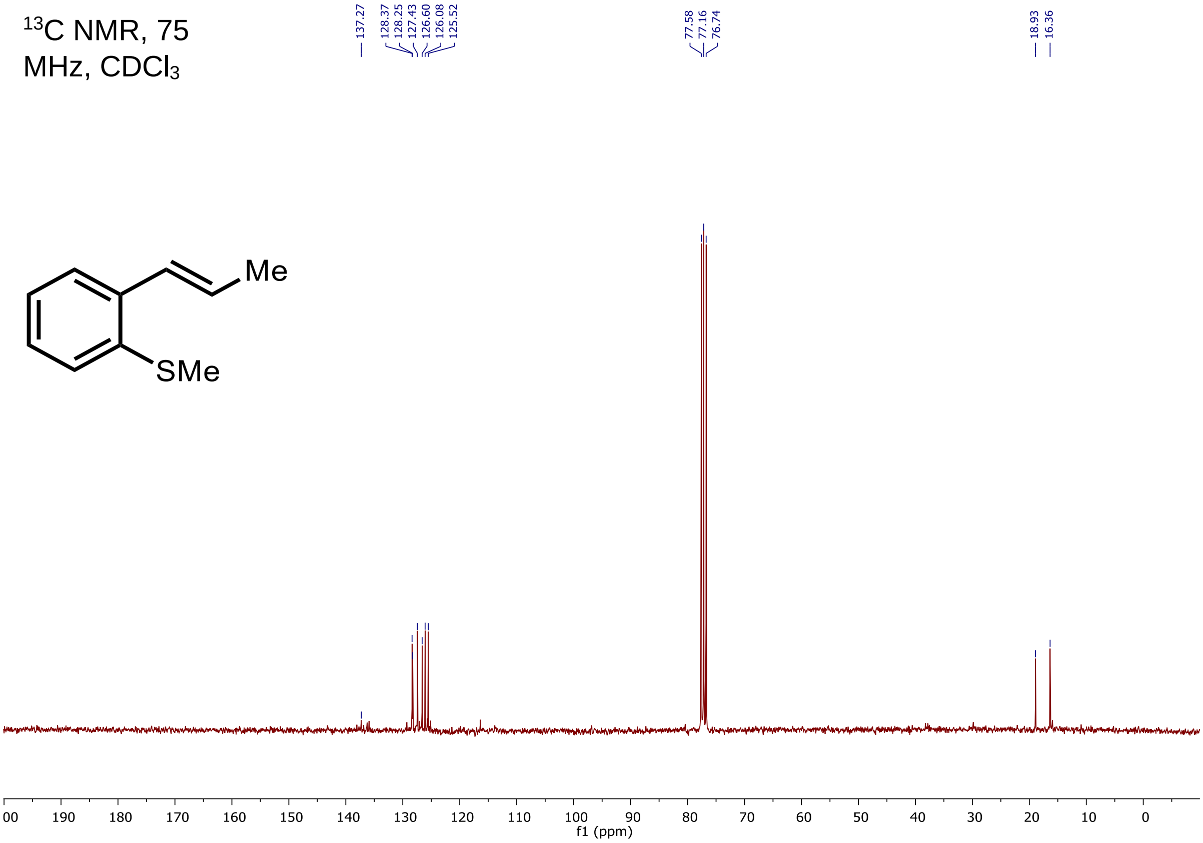

(*E*)-1-Methyl-2-(prop-1-en-1-yl)benzene (19)[26]

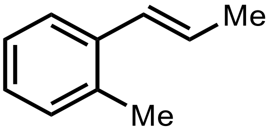

The title compound was prepared according to general procedure 3 using 2-allyl-methylbenzene (0.2 mmol). Yield determined by crude ^{1}H NMR using 1,3,5-trimethylbenzene as internal standard: 98% (*E*:*Z* = 86:14).

Resolved signals of the major isomer (*E*)-1-methyl-2-(prop-1-en-1-yl)benzene:

^{1}H NMR (500 MHz, Chloroform-*d*) δ 6.93 (dq, *J* = 15.6, 1.8 Hz, 1H), 6.41 (dq, *J* = 15.6, 6.6 Hz, 1H), 2.22 (dd, *J* = 6.6, 1.8 Hz, 3H).

Resolved signals of the minor isomer (*Z*)-1-methyl-2-(prop-1-en-1-yl)benzene:[28]

^{1}H NMR (500 MHz, Chloroform-*d*) δ 6.80 (dq, *J* = 11.5, 1.9 Hz, 1H), 6.14 (dq, *J* = 11.4, 7.0 Hz, 1H), 2.07 (dd, *J* = 7.0, 1.8 Hz, 3H).

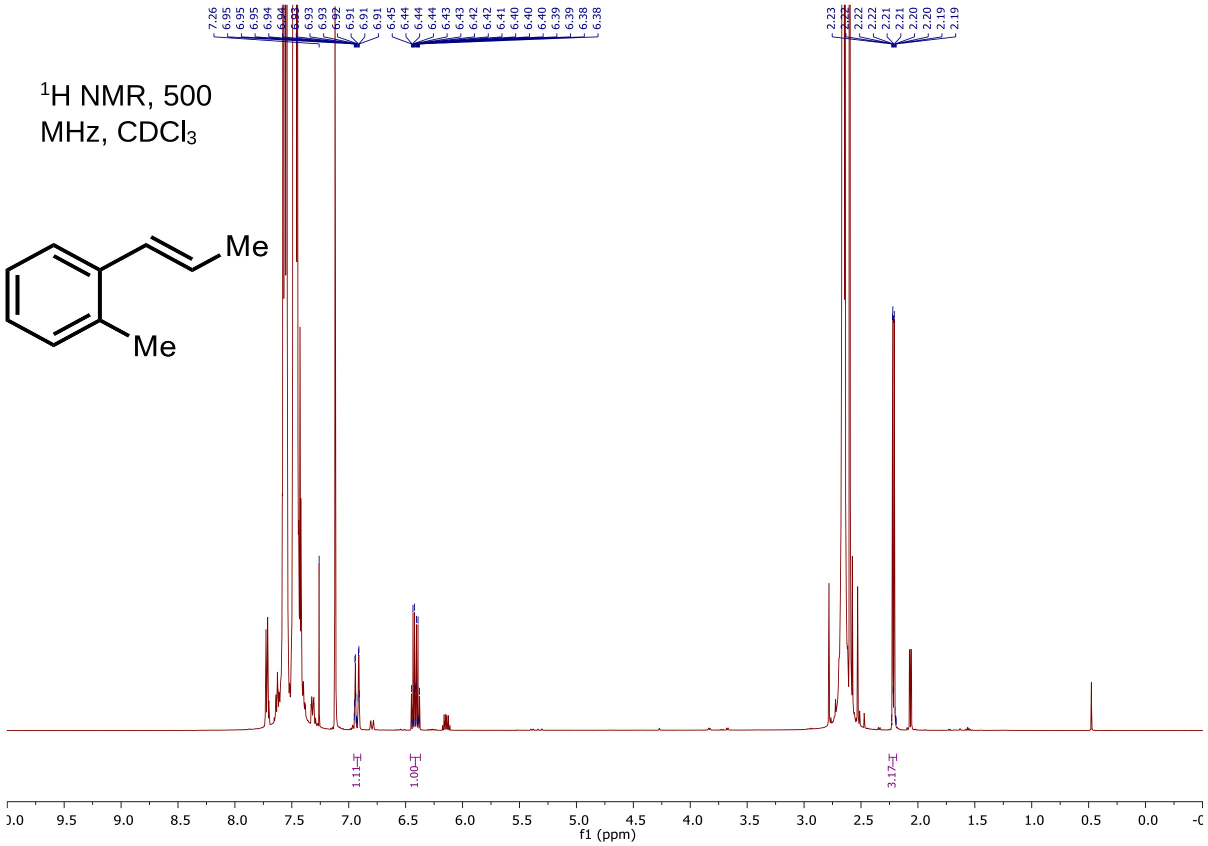

2-(1*E*)-1-Propen-1-yl-1,1’-biphenyl (20)[38]

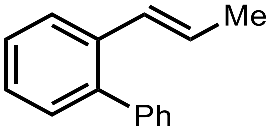

The title compound was prepared according to general procedure 3 using 3-(2-biphenyl)-1-propene (0.2 mmol). Purification by flash silica chromatography (eluent = 10% EtOAc in hexanes) gave the title compound as yellow liquid (31.8 mg, 82%) as a mixture of *E*/*Z* products; R_f = 0.70 (eluent = 10% EtOAc in hexane). NMR yield = >98% (*E*:*Z* = 93:7).

^{1}H NMR (500 MHz, Chloroform-*d*) δ 7.59 (dt, *J* = 8.1, 0.9 Hz, 1H), 7.46 – 7.42 (m, 2H), 7.40 – 7.37 (m, 3H), 7.35 – 7.30 (m, 1H), 7.30 – 7.27 (m, 2H), 6.44 – 6.38 (m, 1H), 6.20 (dq, *J* = 15.7, 6.6 Hz, 1H), 1.82 (dd, *J* = 6.6, 1.7 Hz, 3H).

^{13}C NMR (126 MHz, Chloroform-*d*) δ 141.4, 140.3, 136.1, 130.3, 129.9, 128.1, 127.5, 126.9, 126.8, 126.6, 125.9, 18.8.

<u>Resolved signals of the minor isomer 2-(1*Z*)-1-Propen-1-yl-1,1’-biphenyl:</u>

^{1}H NMR (500 MHz, Chloroform-*d*) δ 6.35 – 6.28 (m, 1H), 5.73 (dd, *J* = 11.5, 7.1 Hz, 1H), 1.89 – 1.83 (m, 3H).

^{13}C NMR (126 MHz, Chloroform-*d*) δ 129.8, 127.9, 14.6.

^{1}H NMR, 500 MHz, $CDCl_3$

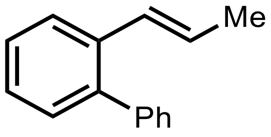

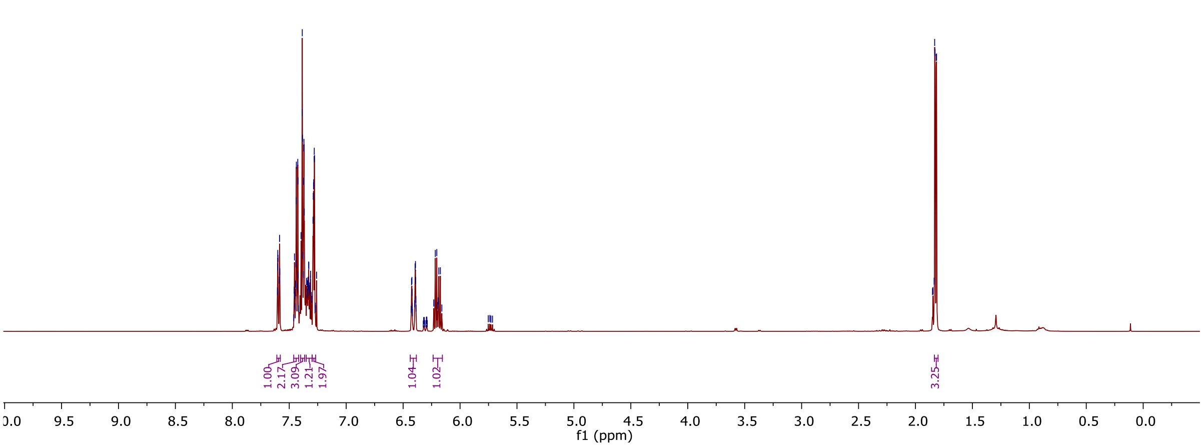

^{13}C NMR, 126 MHz, $CDCl_3$

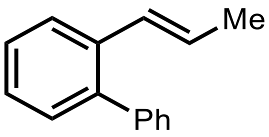

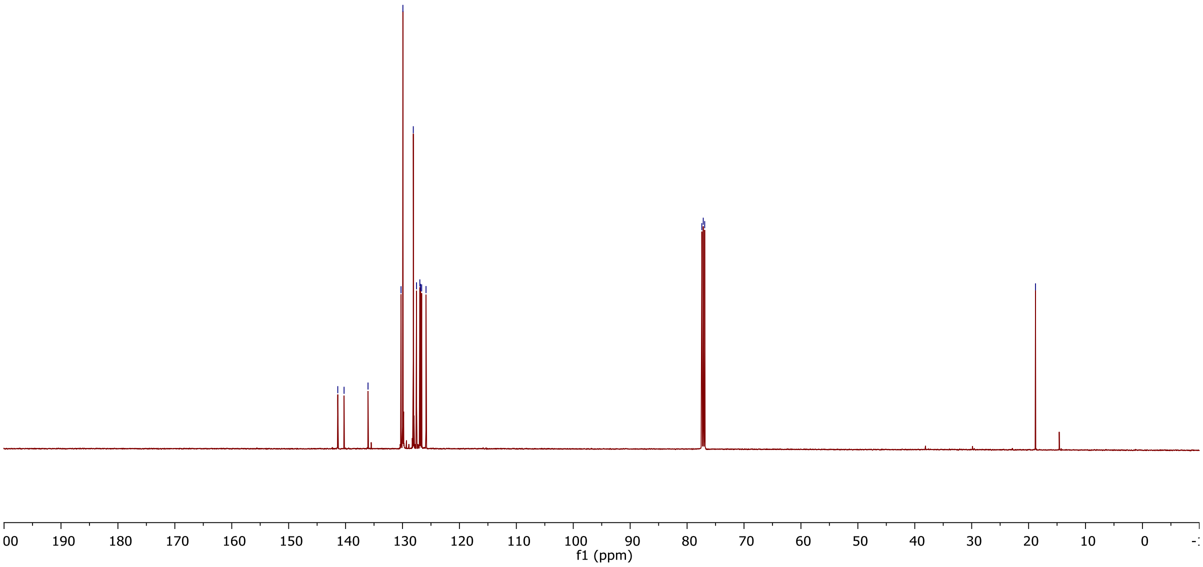

(*E*)-1,3-dimethyl-2-(prop-1-en-1-yl)benzene (21)[26]

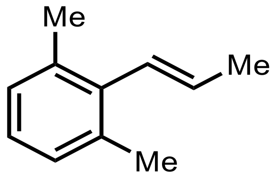

The title compound was prepared according to general procedure 3 using 2-allyl-1,3-dimethylbenzene (0.2 mmol). Yield determined by crude ^{1}H NMR using 1,3,5-trimethylbenzene as internal standard: 98% (*E*:*Z* = 96:4).

Resolved signals of the major isomer (*E*)-1,3-dimethyl-2-(prop-1-en-1-yl)benzene:

^{1}H NMR (500 MHz, Chloroform-*d*) δ 6.72 (dt, *J* = 16.1, 1.8 Hz, 1H), 6.11 – 6.01 (m, 1H), 2.27 (dt, *J* = 6.5, 1.7 Hz, 3H).

Resolved signals of the minor isomer (*Z*)-1,3-dimethyl-2-(prop-1-en-1-yl)benzene:[26]

^{1}H NMR (500 MHz, Chloroform-*d*) δ 6.47 – 6.41 (m, 1H), 6.32 – 6.17 (m, 1H), 2.23 (dt, *J* = 6.5, 1.6 Hz, 3H).

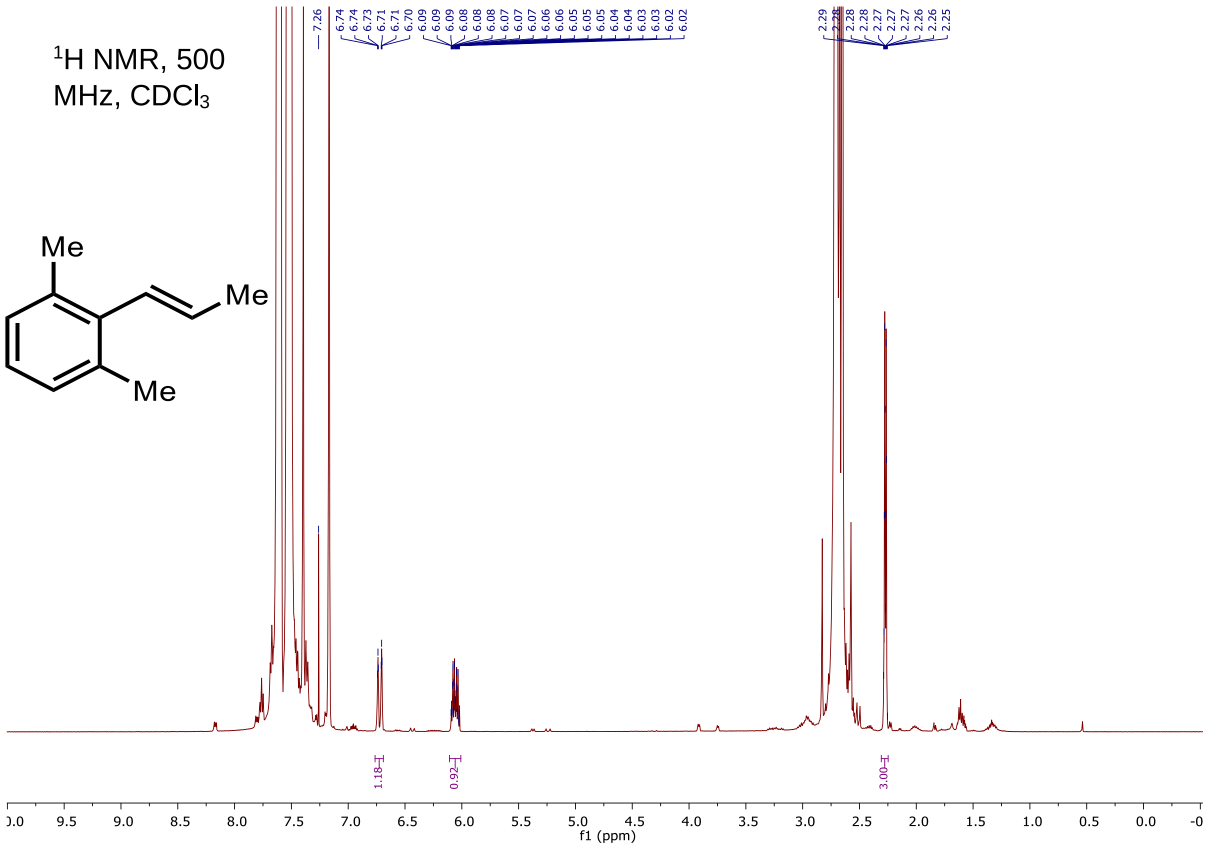

(*E*)-1,3-diisopropyl-2-(prop-1-en-1-yl)benzene (22)

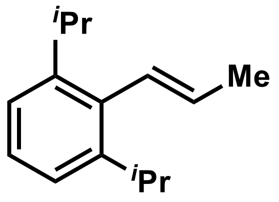

The title compound was prepared according to general procedure 3 using 2-allyl-1,3-diisopropylbenzene (0.2 mmol). Yield determined by crude ^{1}H NMR using 1,3,5-trimethylbenzene as internal standard: 60% (*E*:*Z* = >98:<2).

<u>Resolved signals of the isomer (*E*)-1,3-diisopropyl-2-(prop-1-en-1-yl)benzene:</u>

^{1}H NMR (500 MHz, Chloroform-*d*) δ 6.66 (dq, *J* = 16.0, 1.7 Hz, 1H), 5.79 (dqd, *J* = 16.0, 6.5, 1.4 Hz, 1H), 3.49 (heptd, *J* = 6.9, 1.3 Hz, 2H), 2.12 (dt, *J* = 6.5, 1.5 Hz, 3H), 1.41 (dd, *J* = 6.9, 1.4 Hz, 11H).

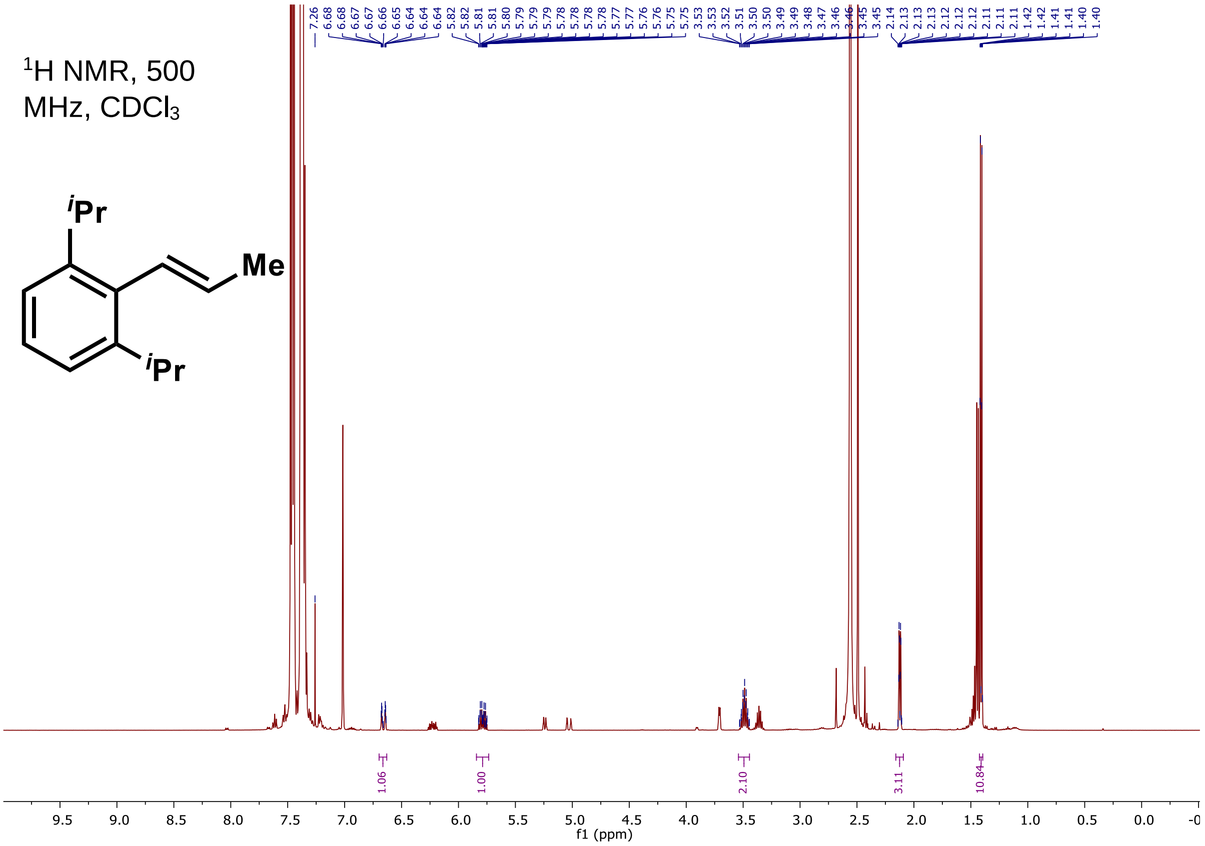

(*E*)-1-(Benzyloxy)-4-(prop-1-en-1-yl)benzene (23)[39]

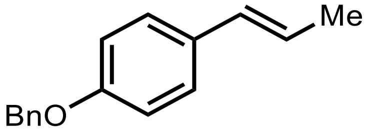

The title compound was prepared according to general procedure 3 using 1-allyl-4-(benzyloxy)benzene (0.2 mmol). Yield determined by crude ^{1}H NMR using 1,3,5-trimethylbenzene as internal standard: 21% (*E*:*Z* = 91:9).

Resolved signals of the major isomer (*E*)-1-(benzyloxy)-4-(prop-1-en-1-yl)benzene:

^{1}H NMR (500 MHz, Chloroform-*d*) δ 6.71 (dq, *J* = 15.7, 1.8 Hz, 1H), 6.44 (dqd, *J* = 14.5, 6.6, 1.4 Hz, 1H), 2.22 (dt, *J* = 6.6, 1.7 Hz, 3H).

Resolved signals of the minor isomer (*Z*)-1-(benzyloxy)-4-(prop-1-en-1-yl)benzene:[40]

^{1}H NMR (500 MHz, Chloroform-*d*) δ 6.79 (dd, *J* = 11.5, 1.9 Hz, 1H), 6.10 (dq, *J* = 11.5, 7.1 Hz, 1H), 2.29 (dt, *J* = 7.2, 1.7 Hz, 3H).

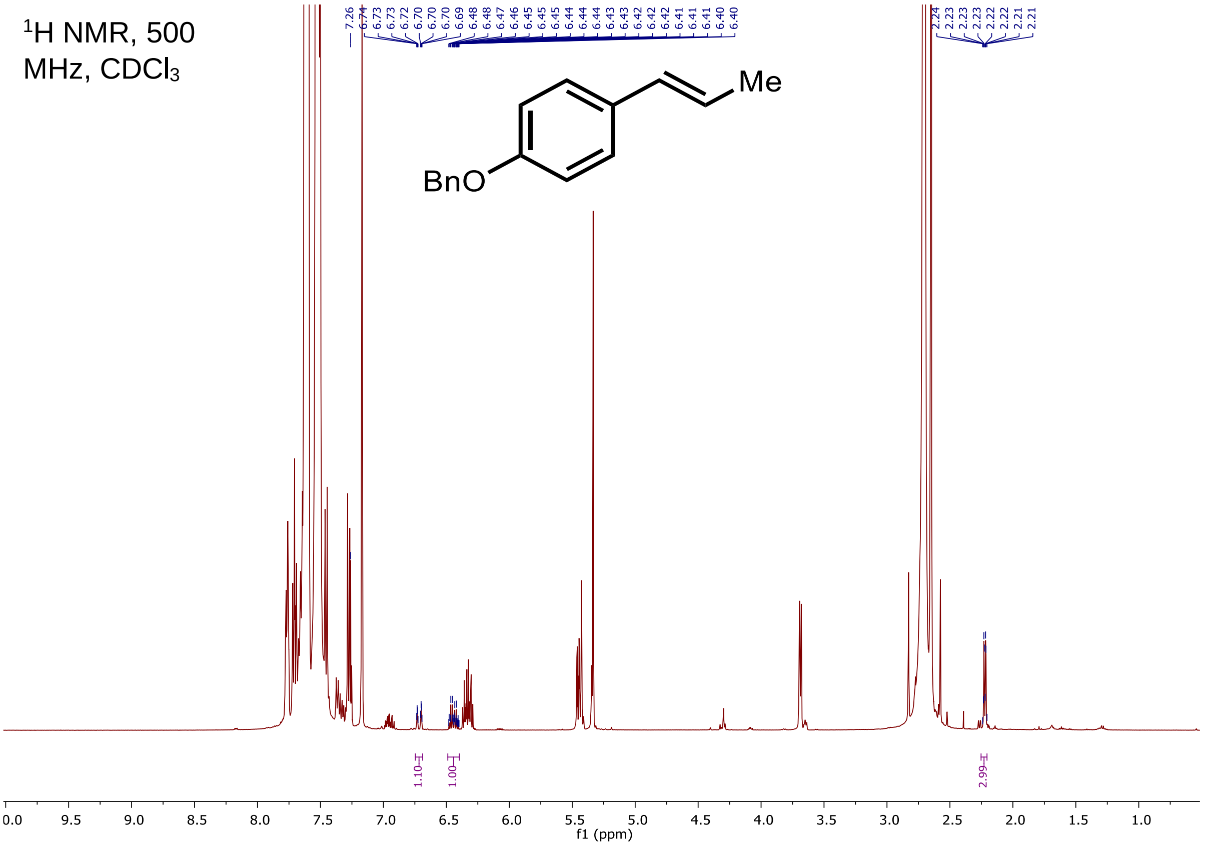

1-(1*E*)-1-Propen-1-yl-naphthalene (29)[38]

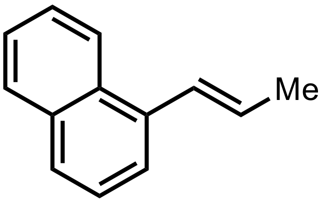

The title compound was prepared according to general procedure 3 using 1-allylnaphthalene (0.2 mmol). Purification by flash silica chromatography (eluent = 10% EtOAc in hexanes) gave the title compound as a yellow liquid (28 mg, 84%) as a mixture of *E*/*Z* products with grease impurity; R_f = 0.75 (eluent = 10% EtOAc in hexanes). NMR yield = 97% (*E*:*Z* = 84:16).

Signals of the major isomer 1-(1*E*)-1-propen-1-yl-naphthalene:

^{1}H NMR (300 MHz, Chloroform-*d*) δ 8.25 – 8.15 (m, 1H), 7.92 – 7.88 (m, 1H), 7.80 (dd, *J* = 8.1, 1.2 Hz, 1H), 7.64 – 7.48 (m, 4H), 7.21 (dq, *J* = 15.5, 1.9 Hz, 1H), 6.31 (dq, *J* = 15.5, 6.6 Hz, 1H), 2.06 (dd, *J* = 6.6, 1.8 Hz, 3H).

^{13}C NMR (75 MHz, Chloroform-*d*) δ 135.9, 133.7, 131.2, 129.1, 128.6, 128.3, 127.3, 125.9, 125.8, 125.7, 124.1, 123.6, 19.1.

<u>Resolved signals of the minor isomer 1-(1*Z*)-1-propen-1-yl-naphthalene:</u>[24]

^{1}H NMR (300 MHz, Chloroform-*d*) δ 7.05 – 6.93 (m, 1H), 6.11 (dq, *J* = 11.4, 7.0 Hz, 1H), 1.82 (dd, *J* = 6.9, 1.9 Hz, 3H).

^{13}C NMR (75 MHz, Chloroform-*d*) δ 134.7, 132.0, 128.7, 128.5, 128.0, 127.2, 126.6, 125.3, 125.2, 14.8.

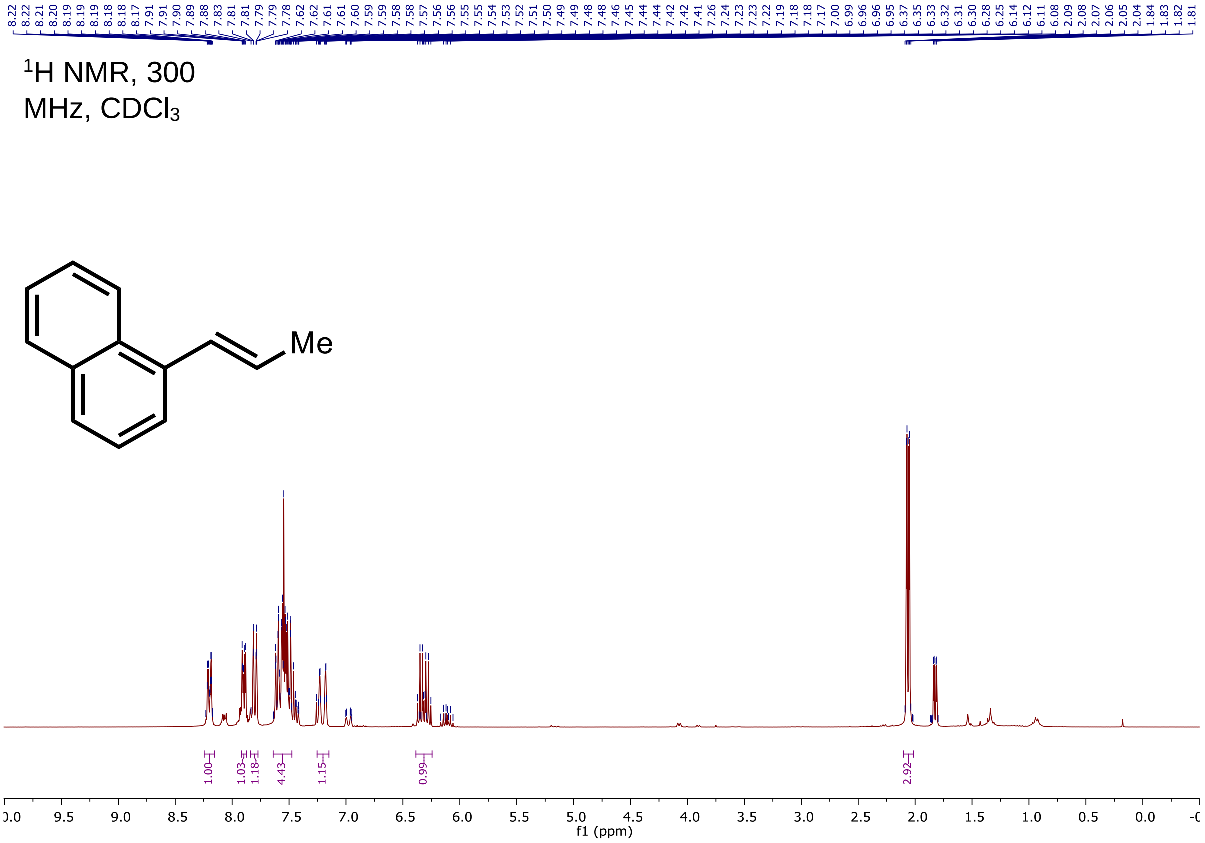

^{13}C NMR, 75 MHz, $CDCl_3$

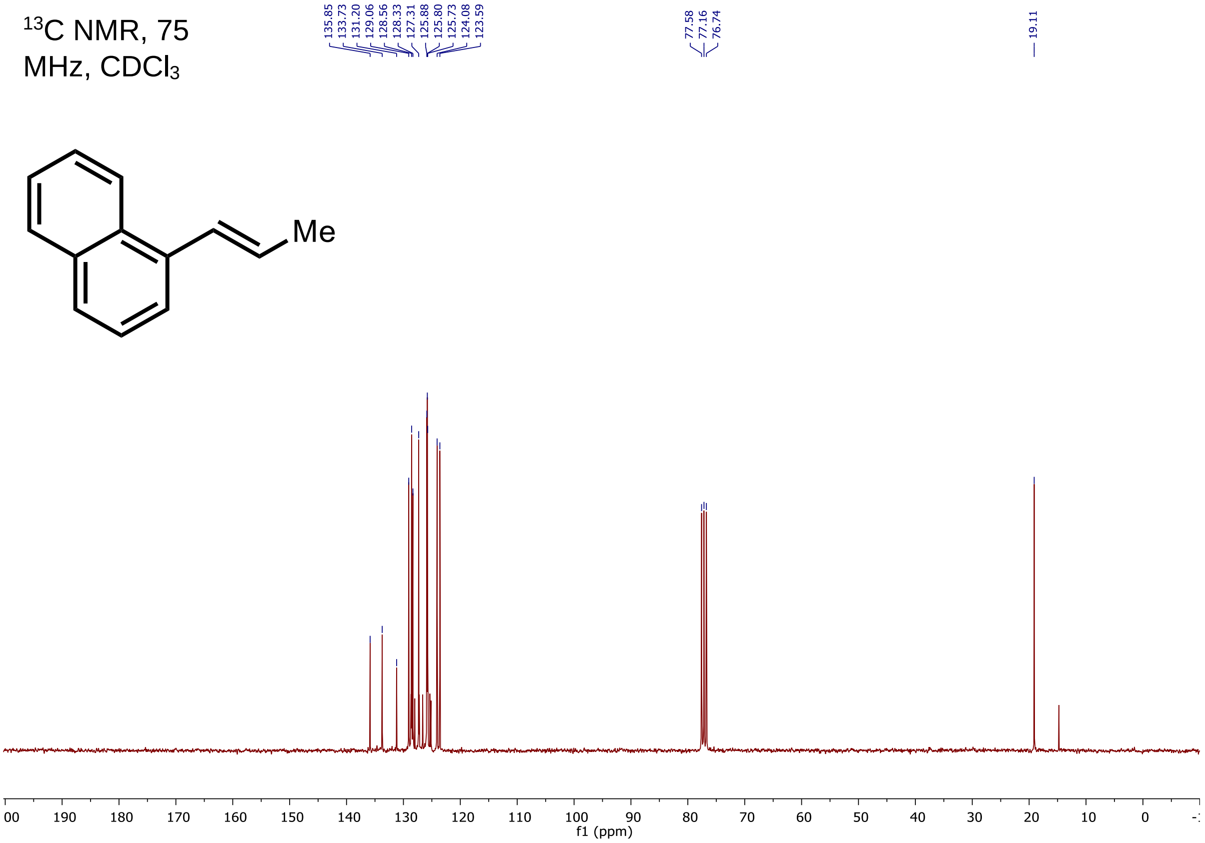

2-(1*E*)-1-Propen-1-yl-naphthalene (30)[29]

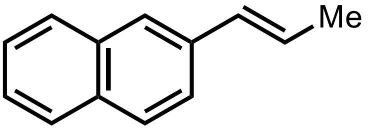

The title compound was prepared according to general procedure 3 using 2-allylnaphthalene (0.2 mmol). Purification by flash silica chromatography (eluent = 10% EtOAc in hexanes) gave the title compound as a white solid (23.5 mg, 70%) as a mixture of *E*/*Z* products with grease impurity; R_f = 0.76 (eluent = 10% EtOAc in hexanes). NMR yield = 95% (*E*:*Z* = 94:6).

^{1}H NMR (500 MHz, Chloroform-*d*) δ 7.80 – 7.75 (m, 3H), 7.69 – 7.65 (m, 1H), 7.58 (dd, *J* = 8.5, 1.8 Hz, 1H), 7.47 – 7.39 (m, 2H), 6.60 – 6.55 (m, 1H), 6.38 (dq, *J* = 15.7, 6.6 Hz, 1H), 1.95 (dd, *J* = 6.6, 1.7 Hz, 3H).

^{13}C NMR (126 MHz, Chloroform-*d*) δ 135.5, 133.8, 132.7, 131.3, 128.2, 127.9, 127.8, 126.3, 126.2, 125.5, 125.3, 123.6, 18.8.

<u>Resolved signals of the minor isomer 2-(1*Z*)-1-Propen-1-yl-naphthalene:</u>[25]

^{1}H NMR (500 MHz, Chloroform-*d*) δ 7.84 – 7.81 (m, 3H), 6.61 (d, 1H), 5.89 (dq, *J* = 11.6, 7.2 Hz, 1H), 1.99 (dd, *J* = 7.2, 1.8 Hz, 3H).

^{13}C NMR (126 MHz, Chloroform-*d*) δ 132.2, 130.0, 127.6, 126.1, 125.9, 125.8, 14.9.

^{1}H NMR, 500 MHz, $CDCl_3$

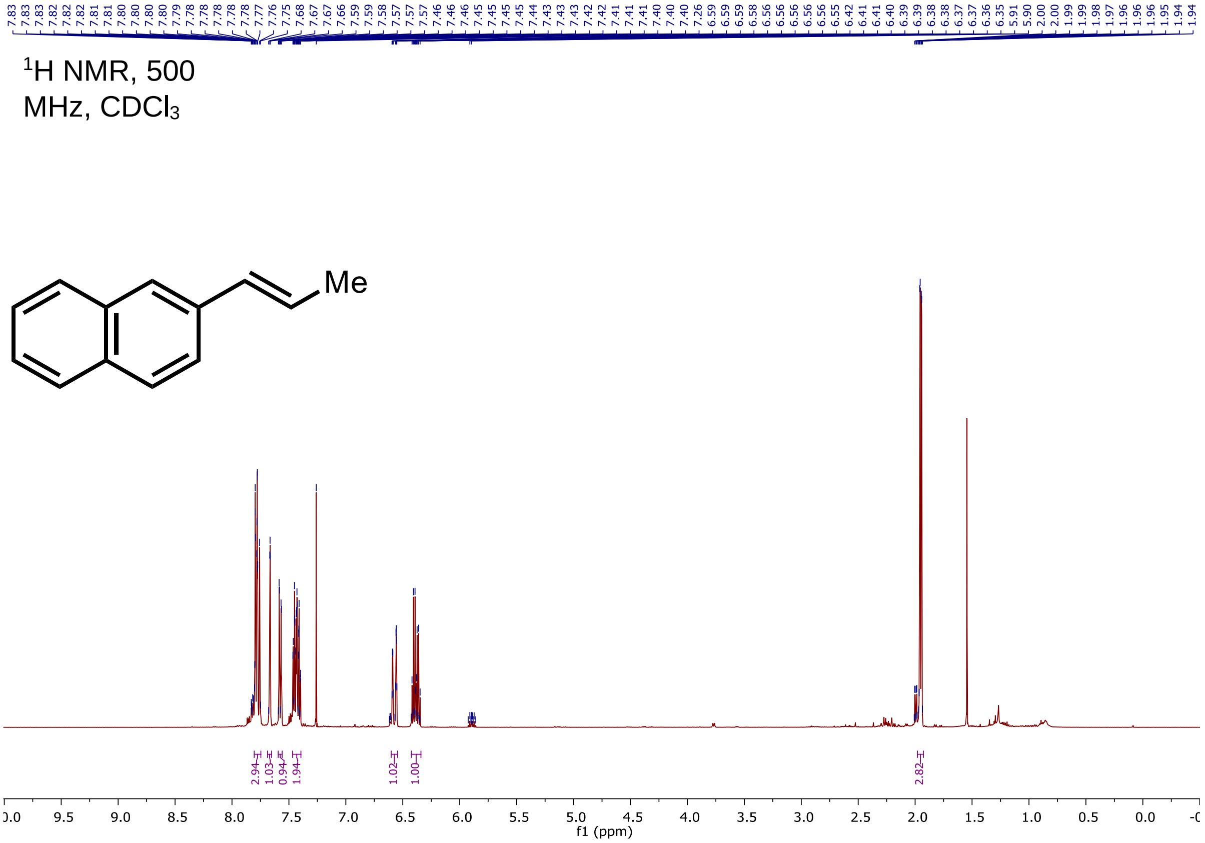

^{13}C NMR, 126 MHz, $CDCl_3$

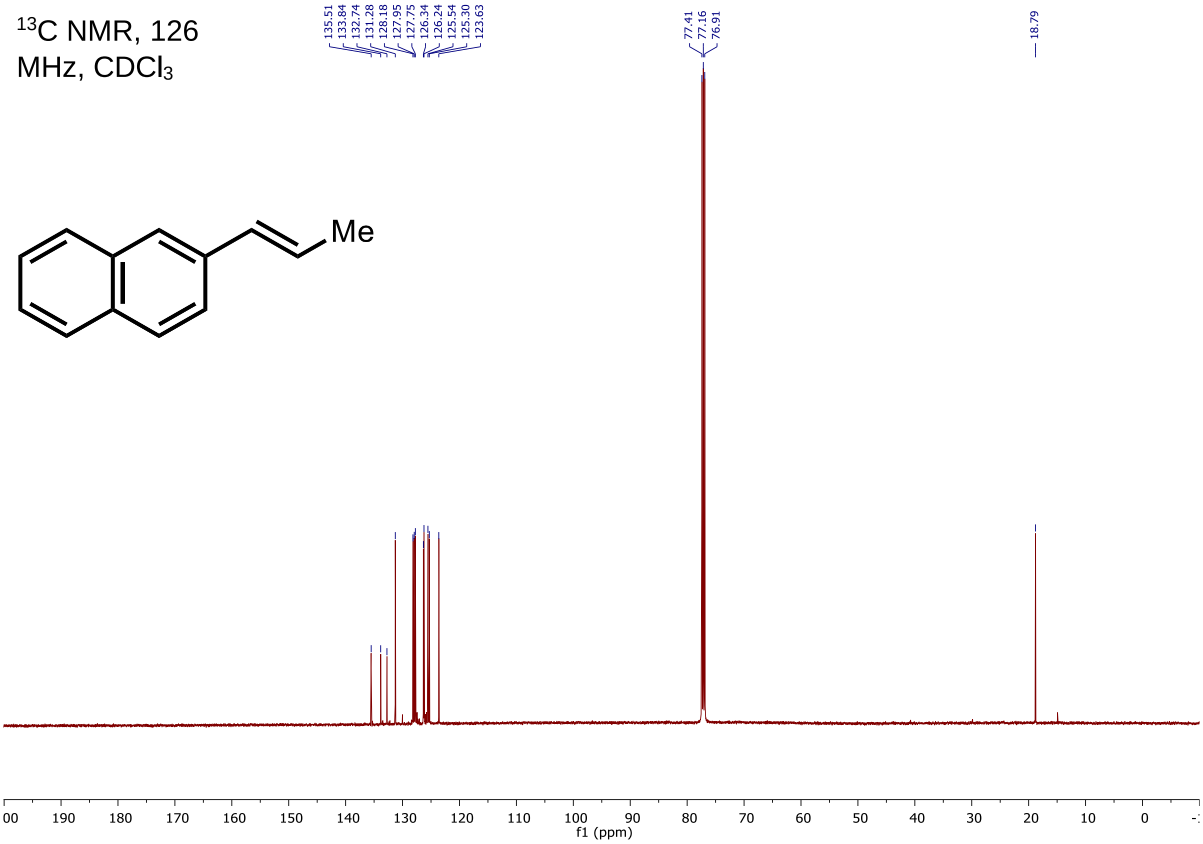

(*E*)-*tert*-butyldimethyl((6-(prop-1-en-1-yl)naphthalen-2-yl)oxy)silane (31)

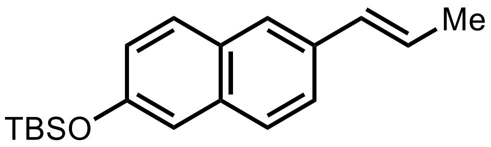

The title compound was prepared according to general procedure 3 using ((6-allylnaphthalen-2-yl)oxy)(*tert*-butyl)dimethylsilane (0.2 mmol). Purification by deactivated flash silica chromatography (eluent = 100% PE) gave the title compound as colourless liquid (45 mg, 76%) as a mixture of *E*/*Z* products. NMR yield = 87% (*E*:*Z* = 97:3).

Signals of the major isomer (*E*)-*tert*-butyldimethyl((6-(prop-1-en-1-yl)naphthalen-2-yl)oxy) silane:

^{1}H NMR (300 MHz, Chloroform-*d*) δ 7.71 – 7.65 (m, 2H), 7.62 – 7.60 (m, 1H), 7.54 (dd, *J* = 8.5, 1.8 Hz, 1H), 7.18 (d, *J* = 2.4 Hz, 1H), 7.07 (dd, *J* = 8.8, 2.4 Hz, 1H), 6.59 – 6.50 (m, 1H), 6.33 (dq, *J* = 15.7, 6.5 Hz, 1H), 1.95 (dd, *J* = 6.5, 1.6 Hz, 3H), 1.05 (s, 9H), 0.28 (s, 6H).

^{13}C NMR (75 MHz, Chloroform-*d*) δ 153.4, 133.9, 133.6, 131.3, 129.6, 129.4, 126.9, 125.3, 125.1, 123.9, 122.4, 115.0, 25.9, 18.8, 18.4, -4.2.

IR (film, ν_{max} / cm^{-1}) 2951, 2928, 2857, 1628, 1599, 1477, 1258, 1244, 1179, 1152.

Resolved signals of the minor isomer (*Z*)-*tert*-butyldimethyl((6-(prop-1-en-1-yl)naphthalen-2-yl)oxy)silane:

^{1}H NMR (300 MHz, Chloroform-*d*) δ 7.45 – 7.41 (m, 1H), 7.21 (d, *J* = 2.6 Hz, 1H), 7.12 (dd, *J* = 4.6, 2.2 Hz, 1H), 5.86 (dq, *J* = 11.5, 7.2 Hz, 1H), 2.01 (dd, *J* = 7.2, 1.8 Hz, 3H).

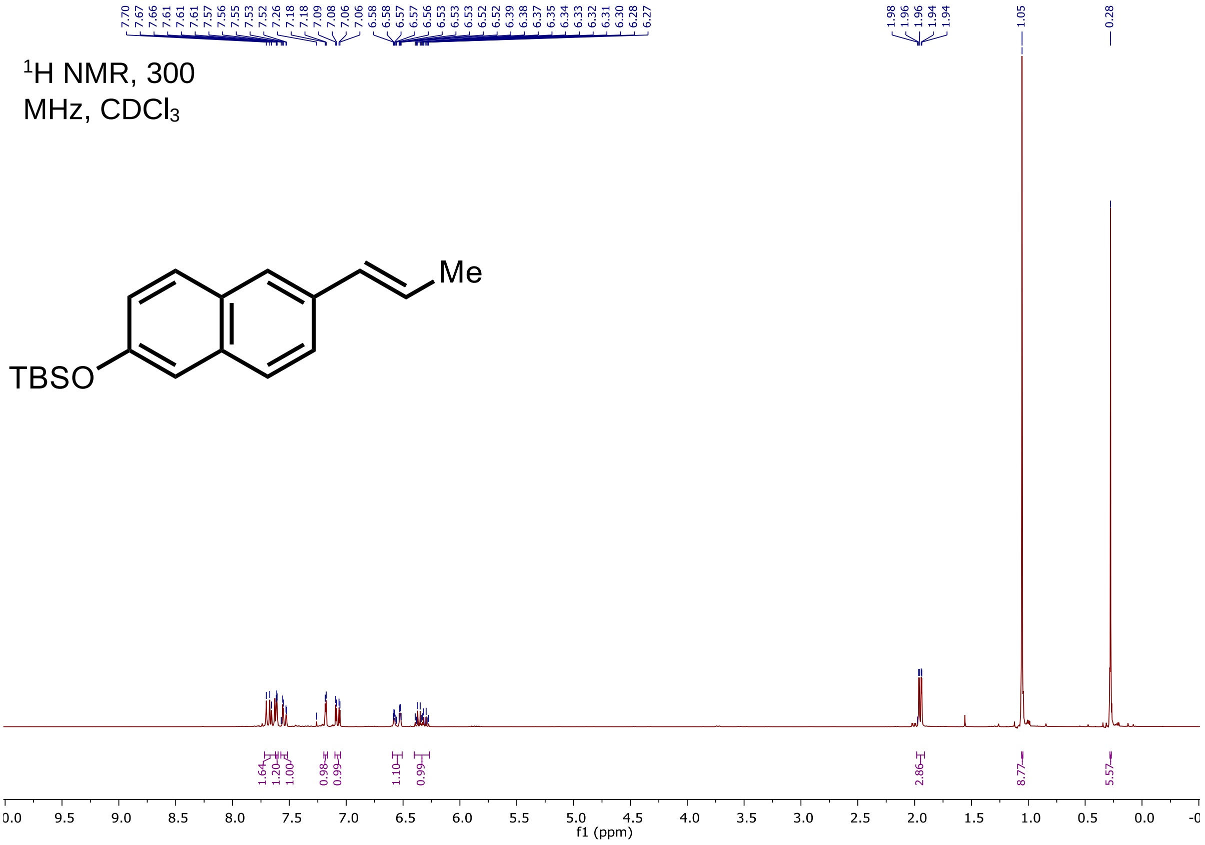

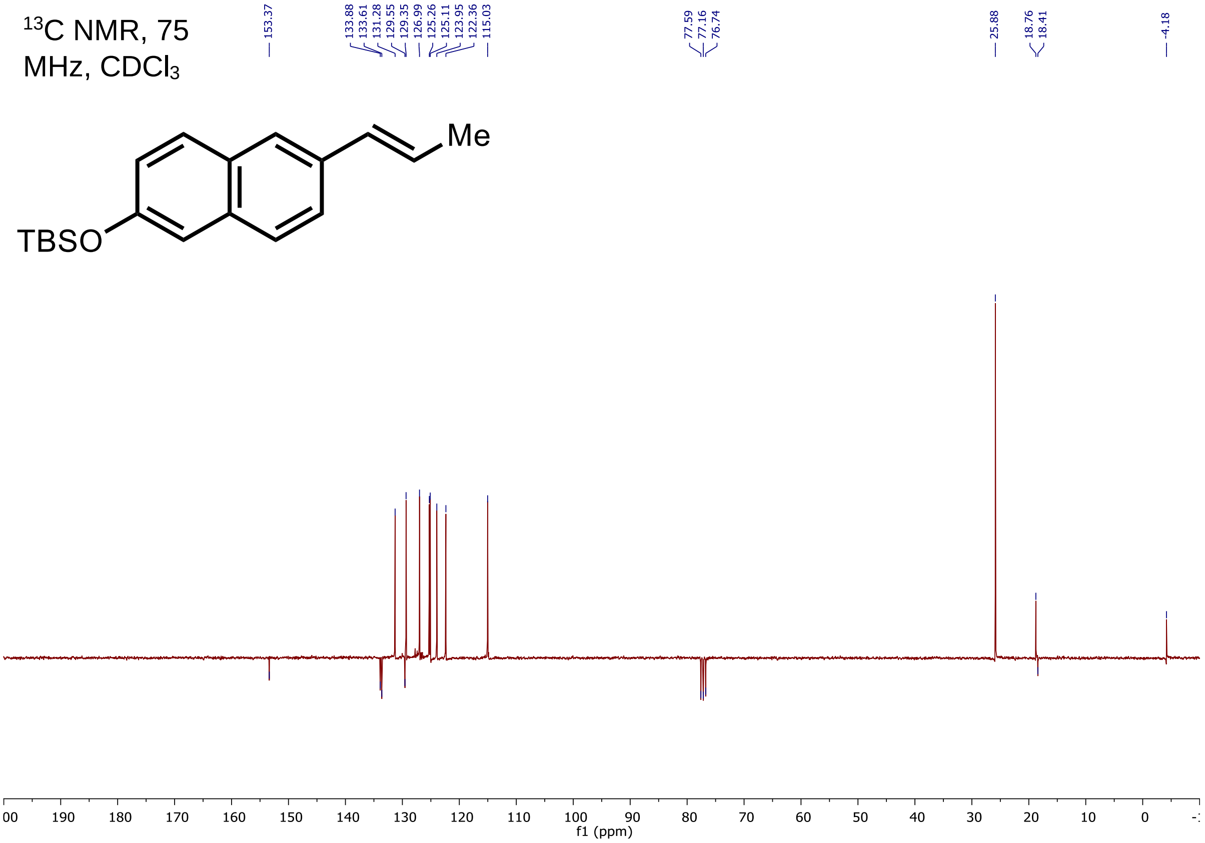

(*E*)-6-methoxy-1-(prop-1-en-1-yl)naphthalene (32)[29]

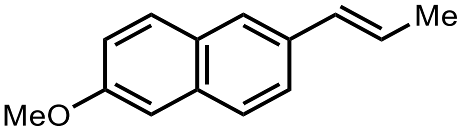

The title compound was prepared according to general procedure 3 using 2-allyl-6-methoxynaphthalene (0.2 mmol). Purification by flash silica chromatography (eluent = 10% EtOAc in hexanes) gave the title compound as white solid (34 mg, 86%) as a mixture of *E*/*Z* products with grease impurity. NMR yield = 91% (*E*:*Z* = 94:6).

<u>Signals of the major isomer (*E*)-6-methoxy-1-(prop-1-en-1-yl)naphthalene:</u>

1H NMR (500 MHz, Chloroform-d) δ 7.69 – 7.64 (m, 2H), 7.59 (s, 1H), 7.53 (dd, *J* = 8.5, 1.8 Hz, 1H), 7.12 – 7.09 (m, 2H), 6.55 – 6.50 (m, 1H), 6.31 (dq, *J* = 15.7, 6.6 Hz, 1H), 3.92 – 3.91 (m, 3H), 1.94 – 1.91 (m, 3H).

^{13}C NMR (126 MHz, Chloroform-*d*) δ 157.5, 133.8, 133.5, 131.2, 129.5, 129.3, 127.1, 125.2, 125.1, 124.2, 118.9, 105.9, 55.5, 18.8.

<u>Resolved signals of the minor isomer (*Z*)-6-methoxy-1-(prop-1-en-1-yl)naphthalene:</u>

^{1}H NMR (500 MHz, Chloroform-*d*) δ 5.83 (dd, *J* = 11.5, 7.2 Hz, 1H), 3.93 (d, *J* = 1.9 Hz, 3H), 1.98 (dd, J = 7.2, 1.8 Hz, 3H).

^{13}C NMR (126 MHz, Chloroform-*d*) δ 14.3.

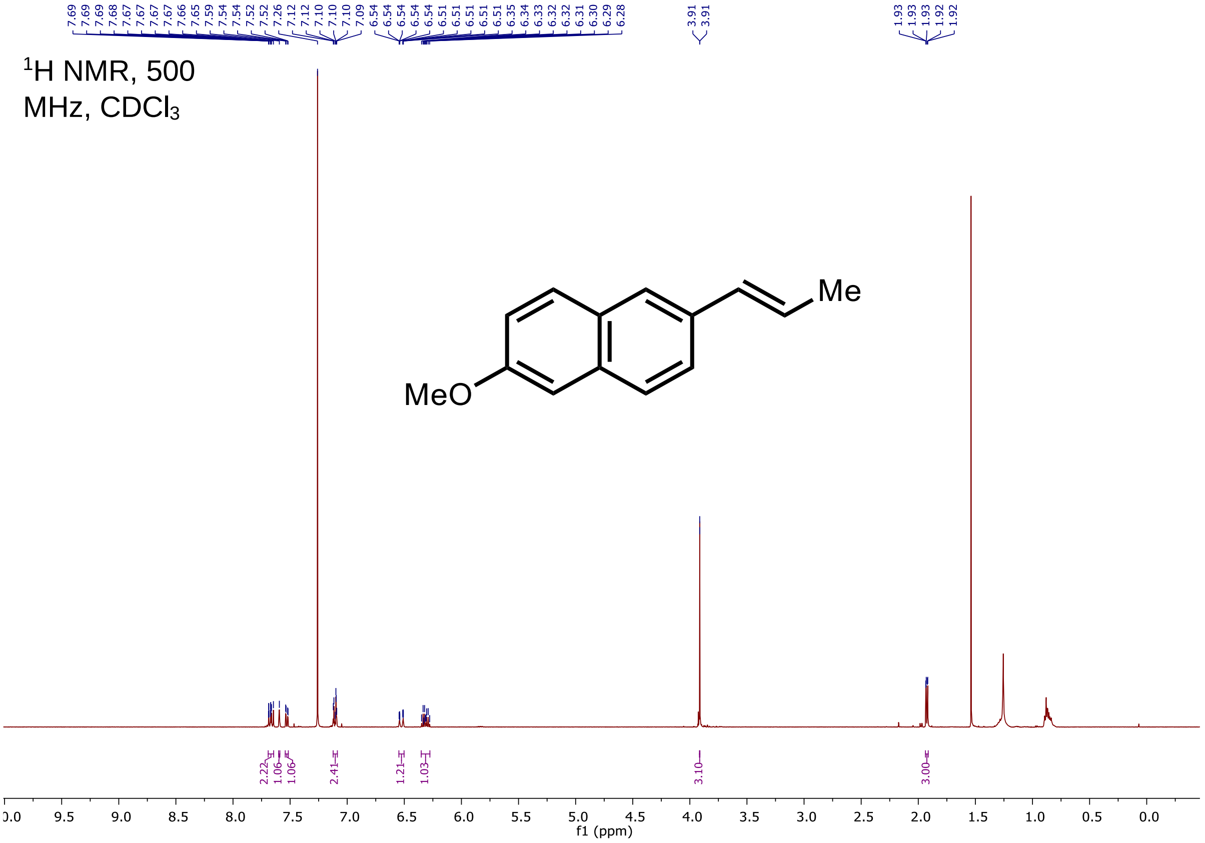

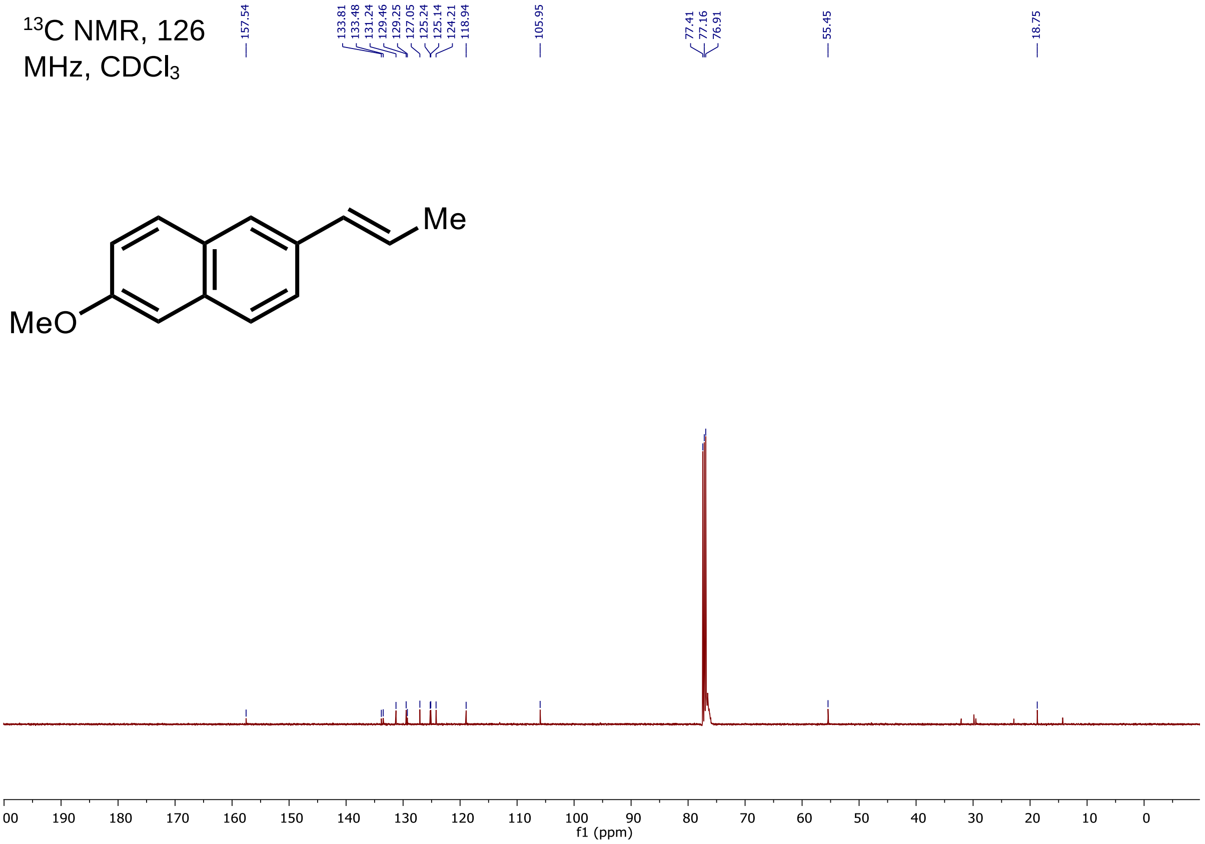

(*E*)-5-(prop-1-en-1-yl)benzofuran (33)[41]

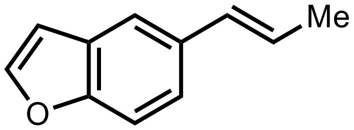

The title compound was prepared according to general procedure 3 using 5-allylbenzofuran (0.2 mmol). Purification by flash silica chromatography (eluent = 10% EtOAc in hexanes) gave the title compound as a colourless oil with grease impurity (26.5 mg, 83%). NMR yield = 75% (*E*:*Z* = 93:7).

Signals of the major isomer (*E*)-5-(prop-1-en-1-yl)benzofuran:

^{1}H NMR (500 MHz, Chloroform-d) δ 7.59 (d, *J* = 2.2 Hz, 1H), 7.52 (d, *J* = 1.8 Hz, 1H), 7.41 (ddt, *J* = 8.5, 1.0, 0.4 Hz, 1H), 7.31 (dd, *J* = 8.6, 1.8 Hz, 1H), 6.73 (dd, *J* = 2.2, 0.9 Hz, 1H), 6.51 – 6.46 (m, 1H), 6.21 (dq, *J* = 15.7, 6.6 Hz, 1H), 1.90 (dd, *J* = 6.6, 1.7 Hz, 3H).

^{13}C NMR (126 MHz, Chloroform-d) δ 154.4, 145.4, 133.2, 131.2, 127.8, 124.7, 122.5, 118.4, 111.4, 106.8, 18.7.

Resolved signals of the minor isomer (*Z*)-5-(prop-1-en-1-yl)benzofuran:

^{1}H NMR (500 MHz, Chloroform-d) δ 5.82 – 5.76 (m, 1H), 1.92 (dd, *J* = 7.2, 1.9 Hz, 3H).

^{13}C NMR (126 MHz, Chloroform-d) δ 14.3.

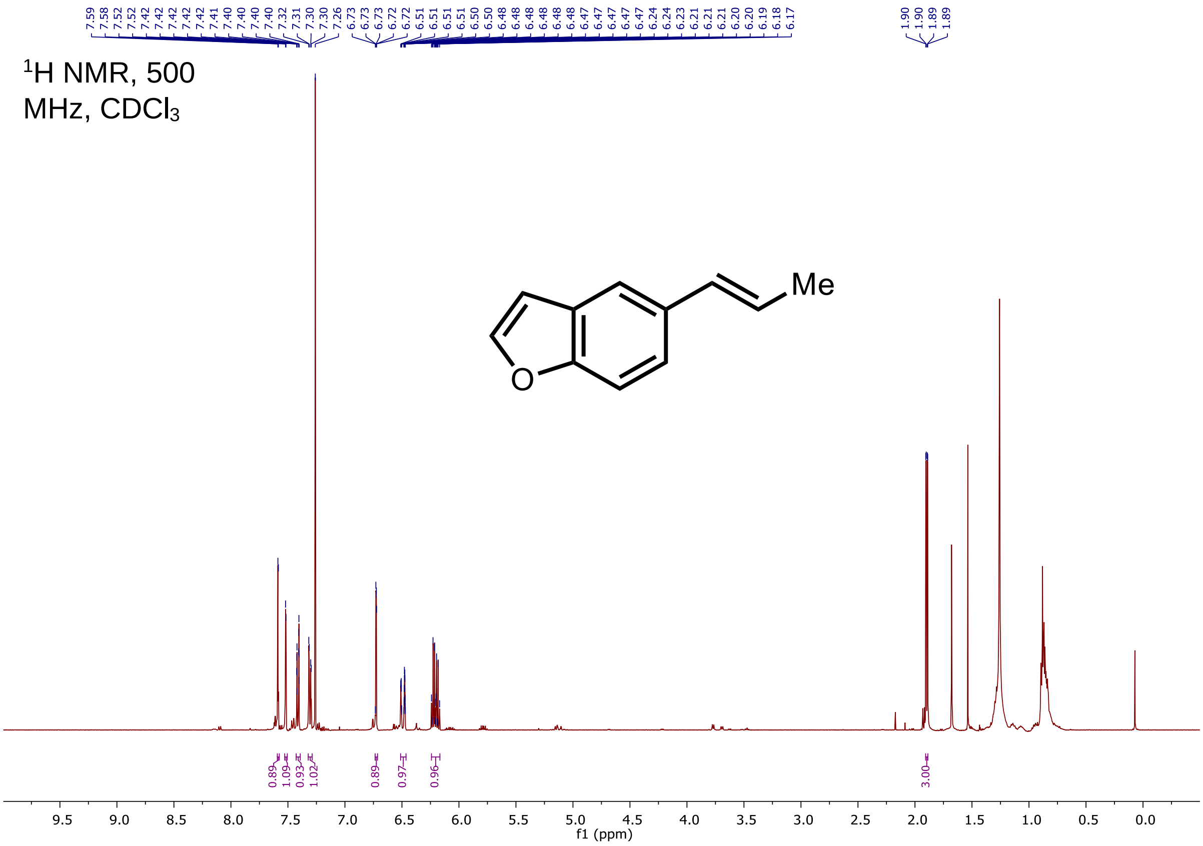

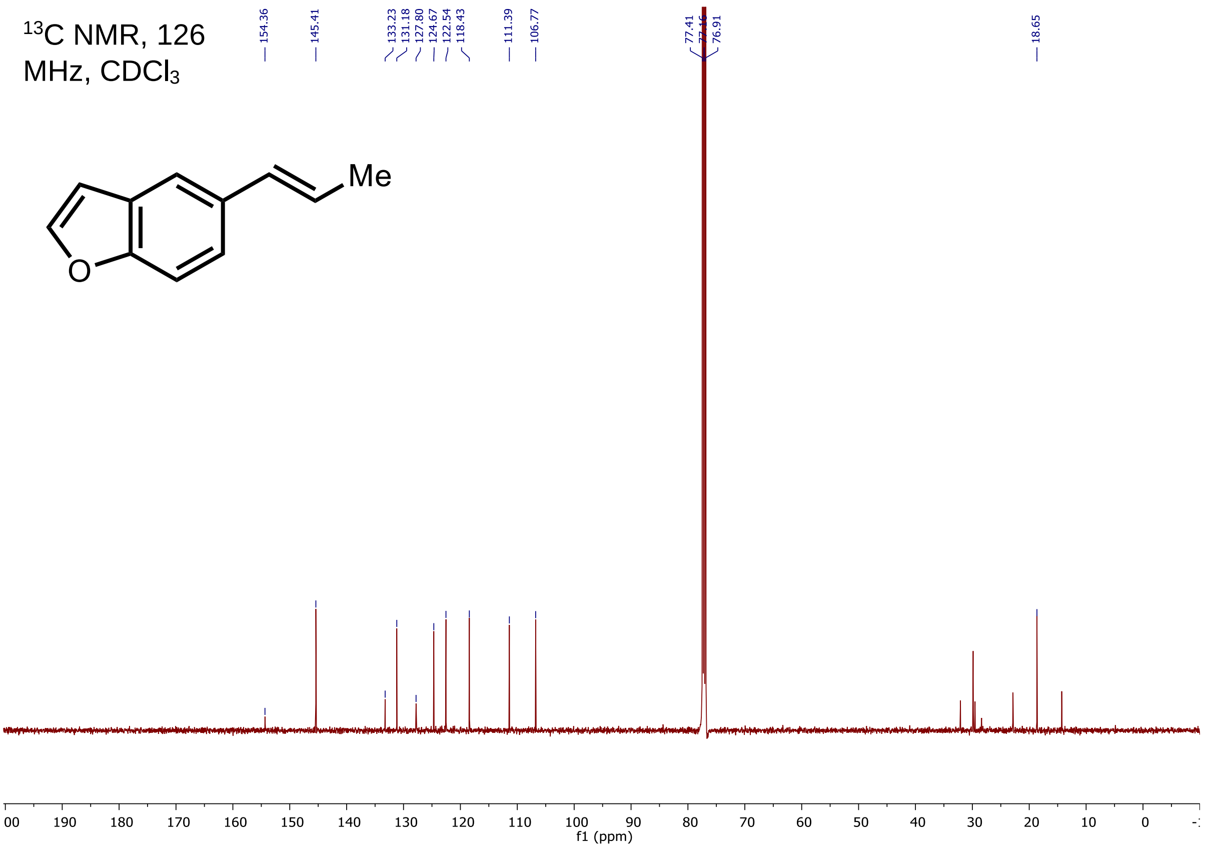

(2-Methylprop-1-en-1-yl)benzene (34)[42]

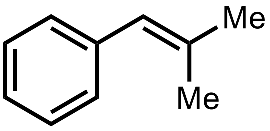

The title compound was prepared according to general procedure 3 using (2-methylallyl)benzene (0.2 mmol). Yield determined by crude ^{1}H NMR using 1,3,5-trimethylbenzene as internal standard: 62%.

<u>Resolved signals of (2-methylprop-1-en-1-yl)benzene:</u>

^{1}H NMR (300 MHz, Chloroform-*d*) δ 6.62 (s, 1H), 2.23 (d, *J* = 1.4 Hz, 3H), 2.19 (d, *J* = 1.3 Hz, 3H).

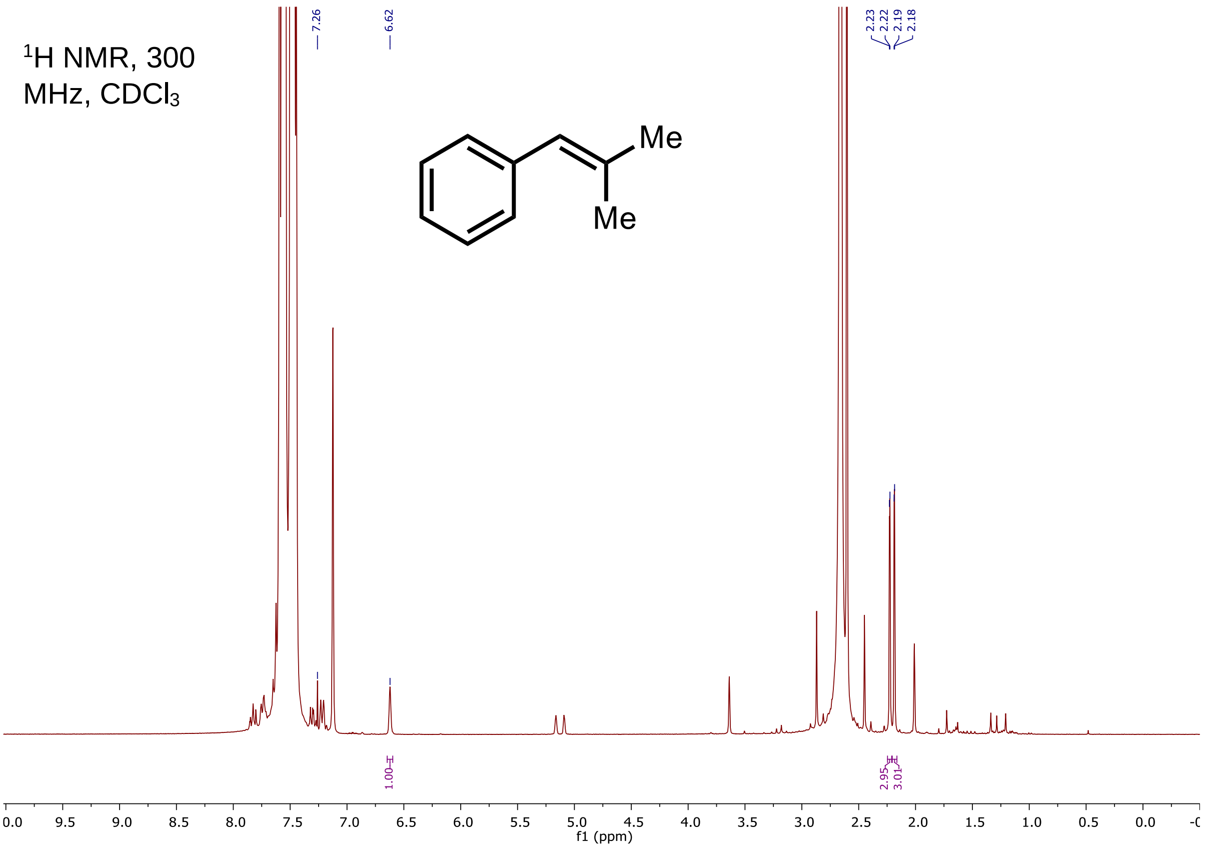

α-Pinene (35)[43]

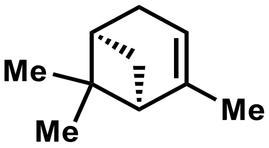

The title compound was prepared according to general procedure 3 using β-pinene (0.2 mmol). Yield determined by crude ^{1}H NMR using 1,3,5-trimethylbenzene as internal standard: 69%.

<u>Resolved signals of α-pinene:</u>

^{1}H NMR (500 MHz, Chloroform-*d*) δ 5.75 (s, 1H).

LRMS (CI) [$C_{10}H_{16}$] (M): m/z 136.12.

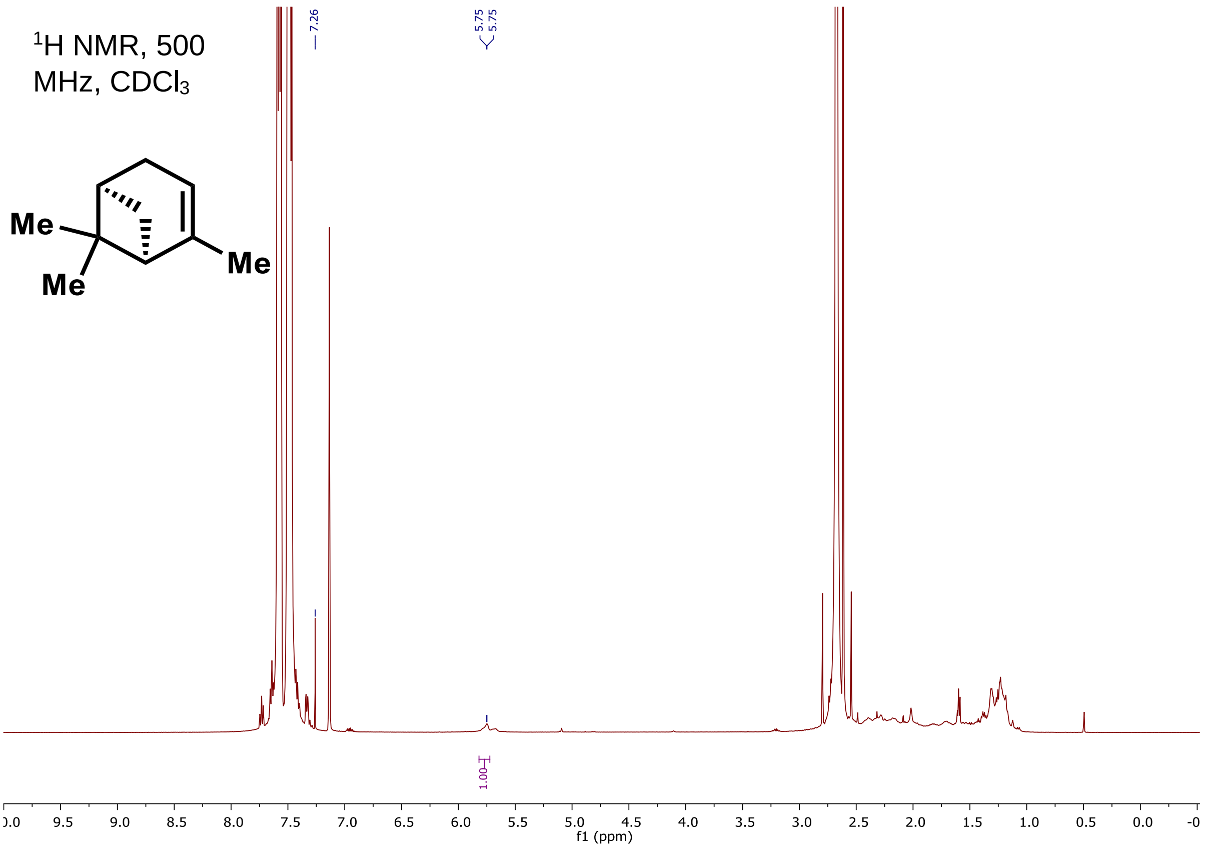

1-Ethylcyclohex-1-ene (36)[44]

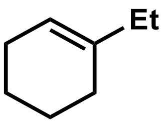

The title compound was prepared according to general procedure 3 using vinylcyclohexane (0.2 mmol) for 48 h. Yield determined by crude ^{1}H NMR using 1,3,5-trimethylbenzene as internal standard: 64%.

Resolved signals of 1-ethylcyclohex-1-ene:

^{1}H NMR (300 MHz, Chloroform-*d*) δ 5.73 (tt, *J* = 3.6, 1.7 Hz, 1H), 2.36 – 2.29 (m, 1H), 2.31 – 2.19 (m, 4H), 1.91 (dddd, *J* = 16.2, 11.7, 4.4, 2.8 Hz, 4H), 1.32 (t, *J* = 7.4 Hz, 3H).

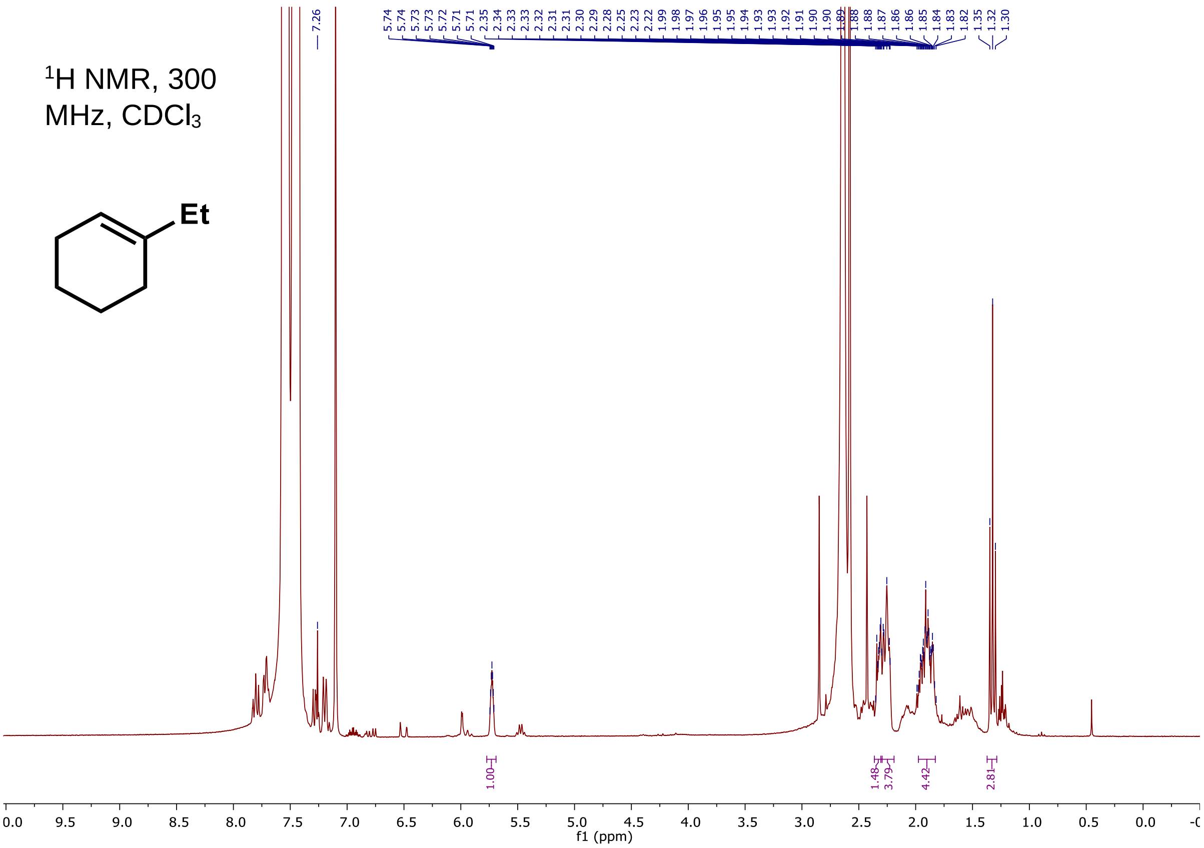

(*E*)-But-1-en-1-ylbenzene (37)[45]

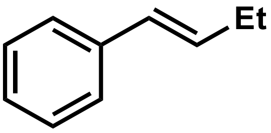

The title compound was prepared according to general procedure 3 using but-3-en-1-yl-benzene (0.2 mmol). Yield determined by crude ^{1}H NMR using 1,3,5-trimethylbenzene as internal standard: 83% (*E*:*Z* = 96:4).

Resolved signals of the major isomer (*E*)-but-1-en-1-ylbenzene:

^{1}H NMR (400 MHz, Chloroform-*d*) δ 6.58 (dt, *J* = 15.8, 1.5 Hz, 1H), 6.51 – 6.41 (m, 1H), 1.31 (td, *J* = 7.5, 1.3 Hz, 3H).

Resolved signals of the minor isomer (*Z*)-but-1-en-1-ylbenzene:[45]

^{1}H NMR (400 MHz, Chloroform-*d*) δ 6.85 – 6.73 (m, 1H), 5.79 – 5.68 (m, 1H).

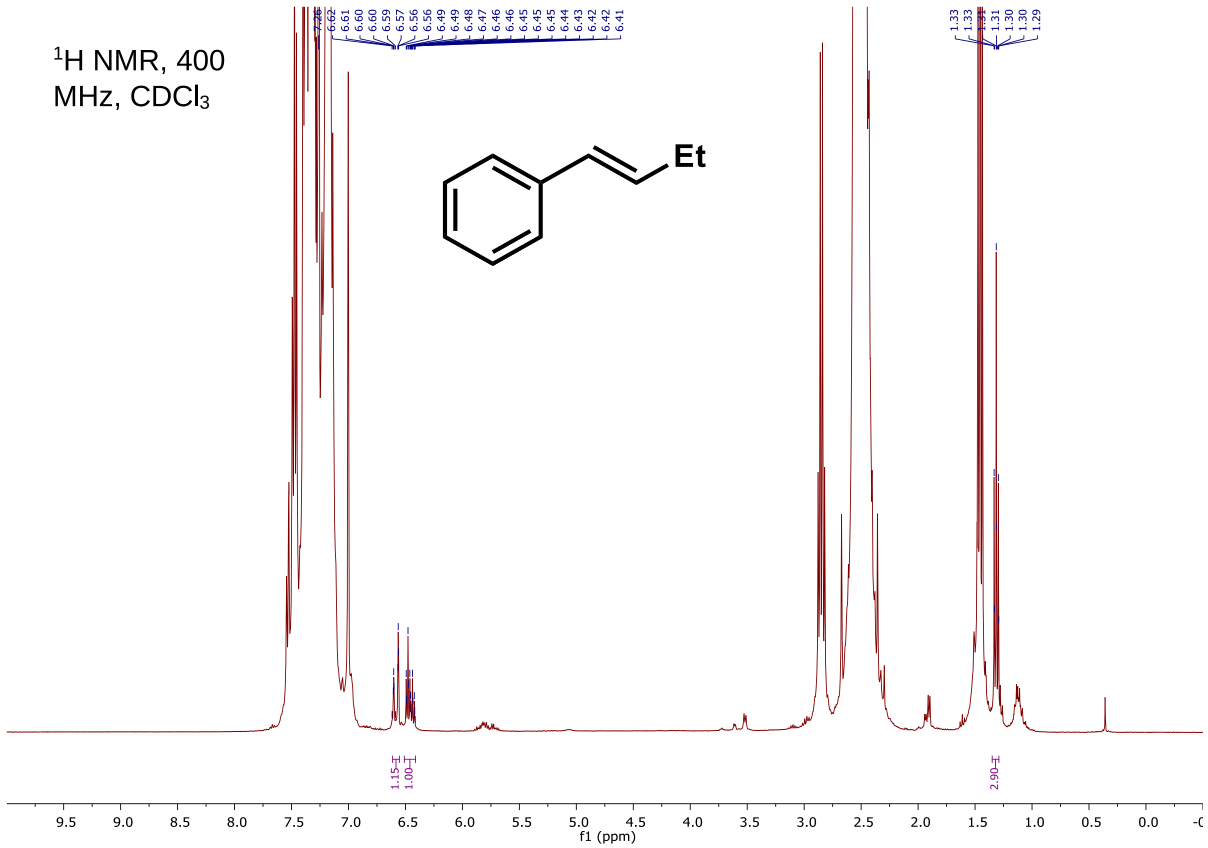

(1Z,3Z)-Cycloocta-1,3-diene (38)[46]

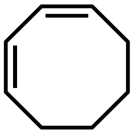

The title compound was prepared according to general procedure 3 using (1Z,5Z)-Cycloocta-1,5-diene (0.2 mmol) for 48 h. Yield determined by crude ^{1}H NMR using 1,3,5-trimethylbenzene as internal standard: >98%.

^{1}H NMR (300 MHz, Chloroform-*d*) δ 6.24 – 6.16 (m, 2H), 6.06 – 5.92 (m, 2H), 1.94 – 1.81 (m, 4H).

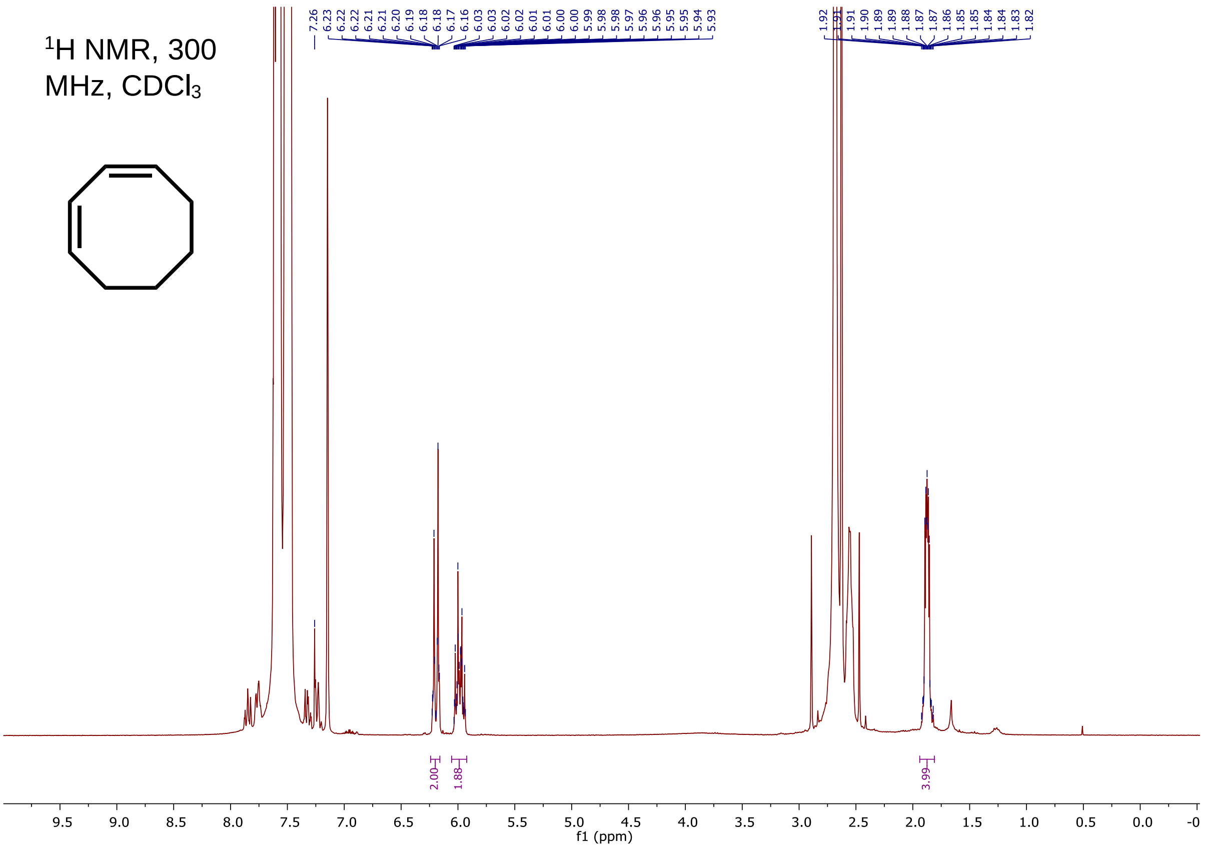

1-Propylcyclopent-1-ene (39)

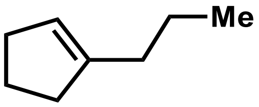

The title compound was prepared according to general procedure 3 using allylcyclopentane (0.2 mmol). Yield determined by crude ^{1}H NMR using 1,3,5-trimethylbenzene as internal standard: 74%.

<u>Resolved signals of 1-propylcyclopent-1-ene:</u>

^{1}H NMR (300 MHz, Chloroform-*d*) δ 5.77 (h, *J* = 1.8 Hz, 1H), 2.47 (t, *J* = 7.8 Hz, 2H), 2.35 – 2.22 (m, 2H), 1.90 (m, 2H), 1.34 (td, *J* = 7.4, 1.7 Hz, 3H).

^{13}C NMR (75 MHz, Chloroform-*d*) δ 35.1, 33.5, 32.6, 23.6, 21.1, 14.1.

^{1}H-^{1}H COSY NMR of crude 1-propylcyclopent-1-ene:

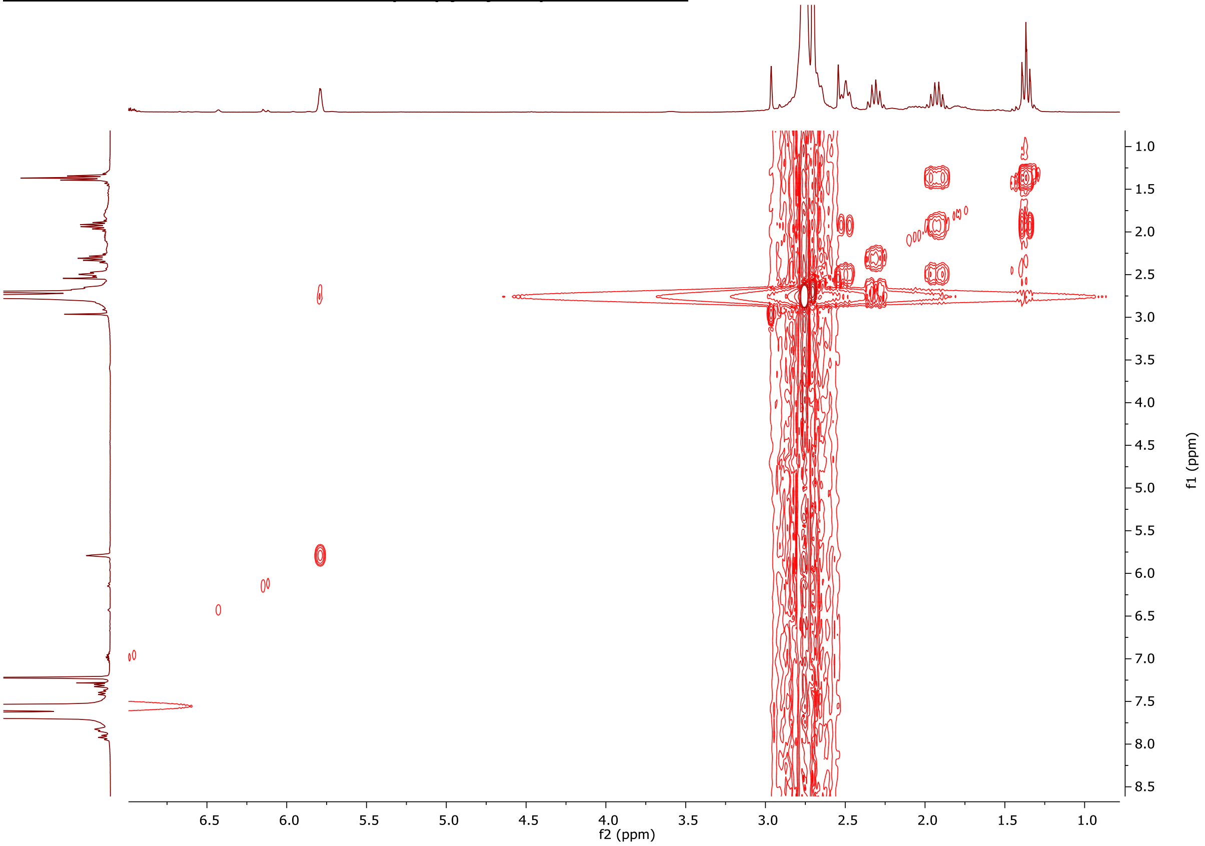

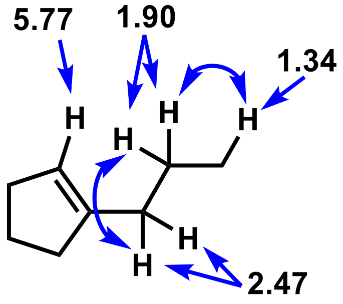

2.4. Mechanistic study

2.4.1. Isotopic solvent effect

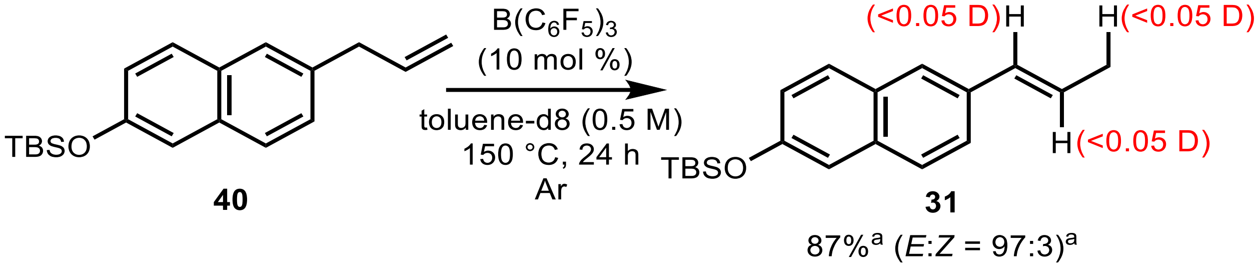

The experiment was prepared according to general procedure 3 using ((6-allylnaphthalen-2-yl)oxy)(*tert*-butyl)dimethylsilane (**40**). Yield determined by crude ^{1}H NMR using 1,3,5-trimethylbenzene as internal standard. Deuterium incorporation was determined by ^{1}H NMR after purification by a preparative TLC.

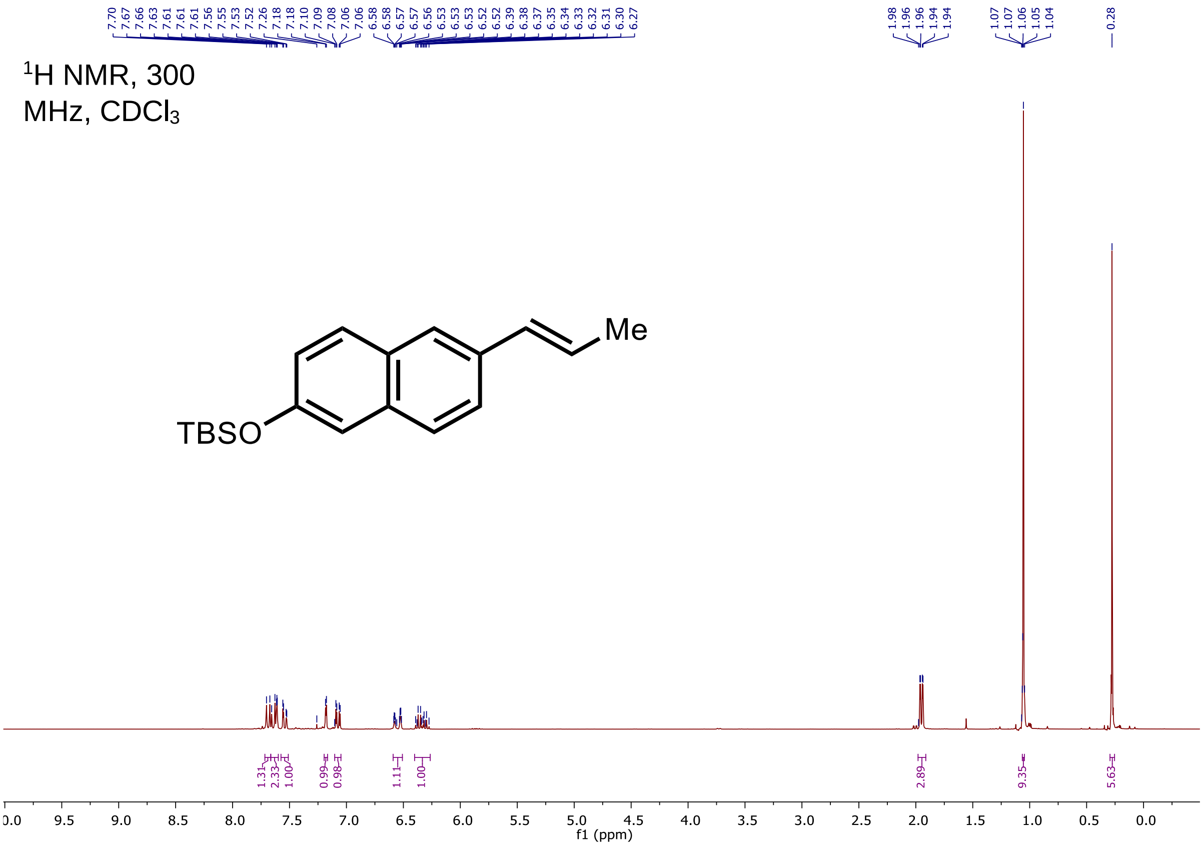

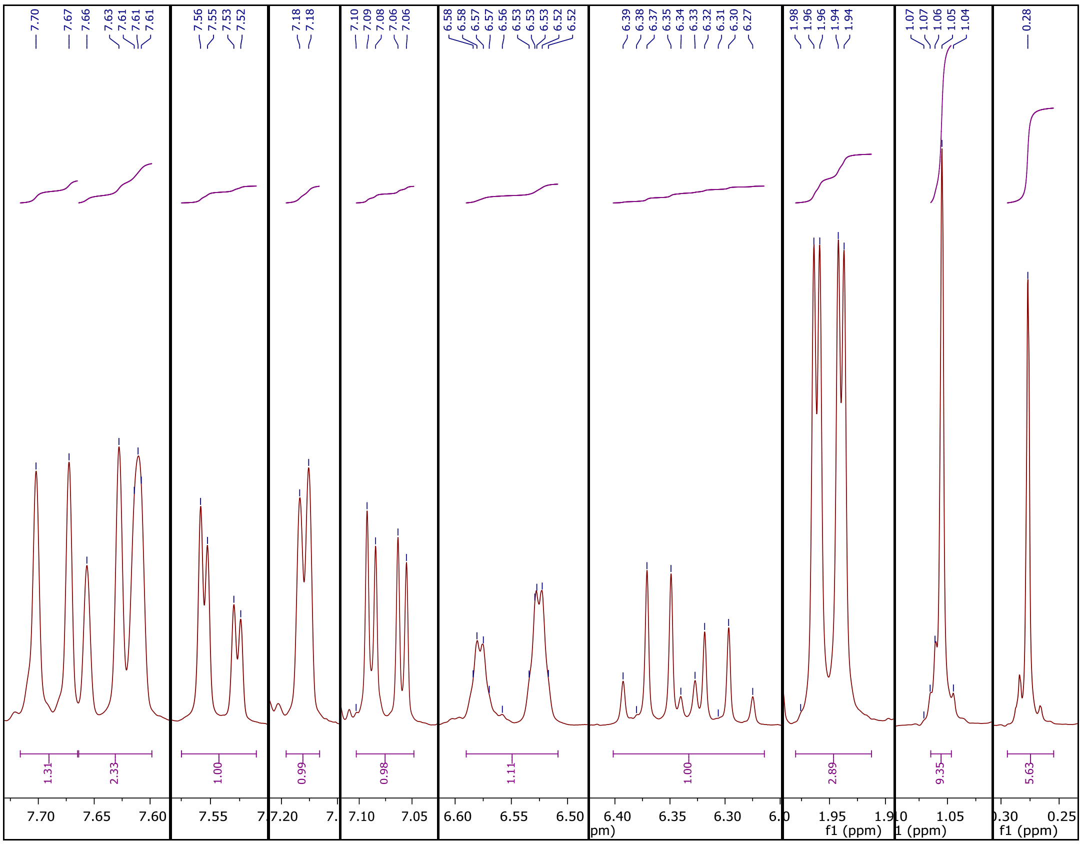

Figure S1. ^{1}H NMR ($CDCl_3$, 300 MHz) of (*E*)-*tert*-butyldimethyl((6-(prop-1-en-1-yl)naphthalen-2-yl)oxy)silane (**31**) after a preparative TLC separation.

2.4.2. Hydrogen isotope effect

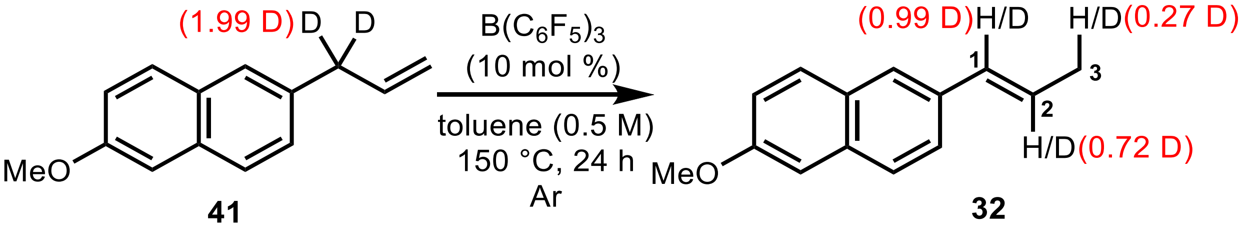

41 **32**

80% (1.98 D)

The experiment was prepared according to general procedure 3 using deuterated 2-allyl-6-methoxynaphthalene (**41**). Yield determined by crude ^{1}H NMR using 1,3,5-trimethylbenzene as internal standard. Deuterium incorporation was determined by ^{1}H NMR after purification by preparative TLC with the integration of aromatic signal (d, 1H) as internal standard.

$$D\ incorporation\ equation = \frac{\Sigma proton - \ integration}{\Sigma proton}$$

$$D\ incorporation\ at\ C1 = \frac{1 - 0.01}{1} = 0.99$$

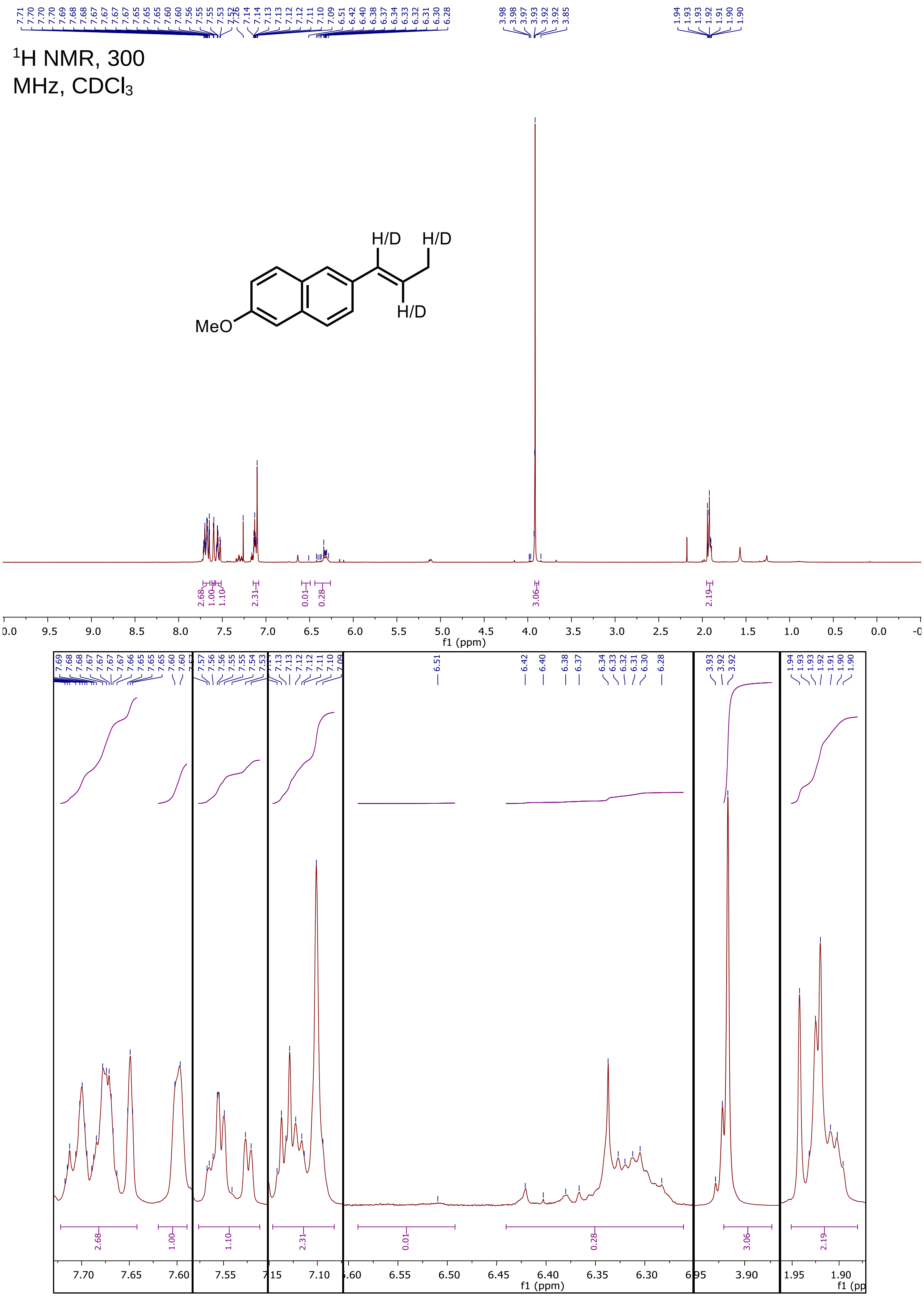

Figure S2. ^{1}H NMR ($CDCl_3$, 300 MHz) of *E*- product of deuterated 2-allyl-6-methoxynaphthalene (**32**).

2.4.3. *E-/Z-* products equilibrium

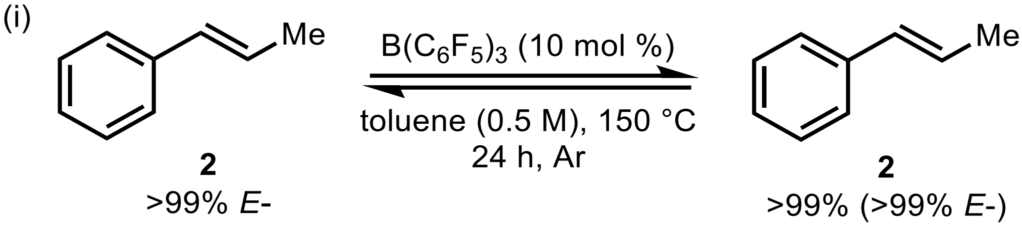

The experiment was prepared according to general procedure 3 using (*E*)-prop-1-en-1-ylbenzene (**2**). Yield determined by crude ^{1}H NMR using 1,3,5-trimethylbenzene as internal standard.

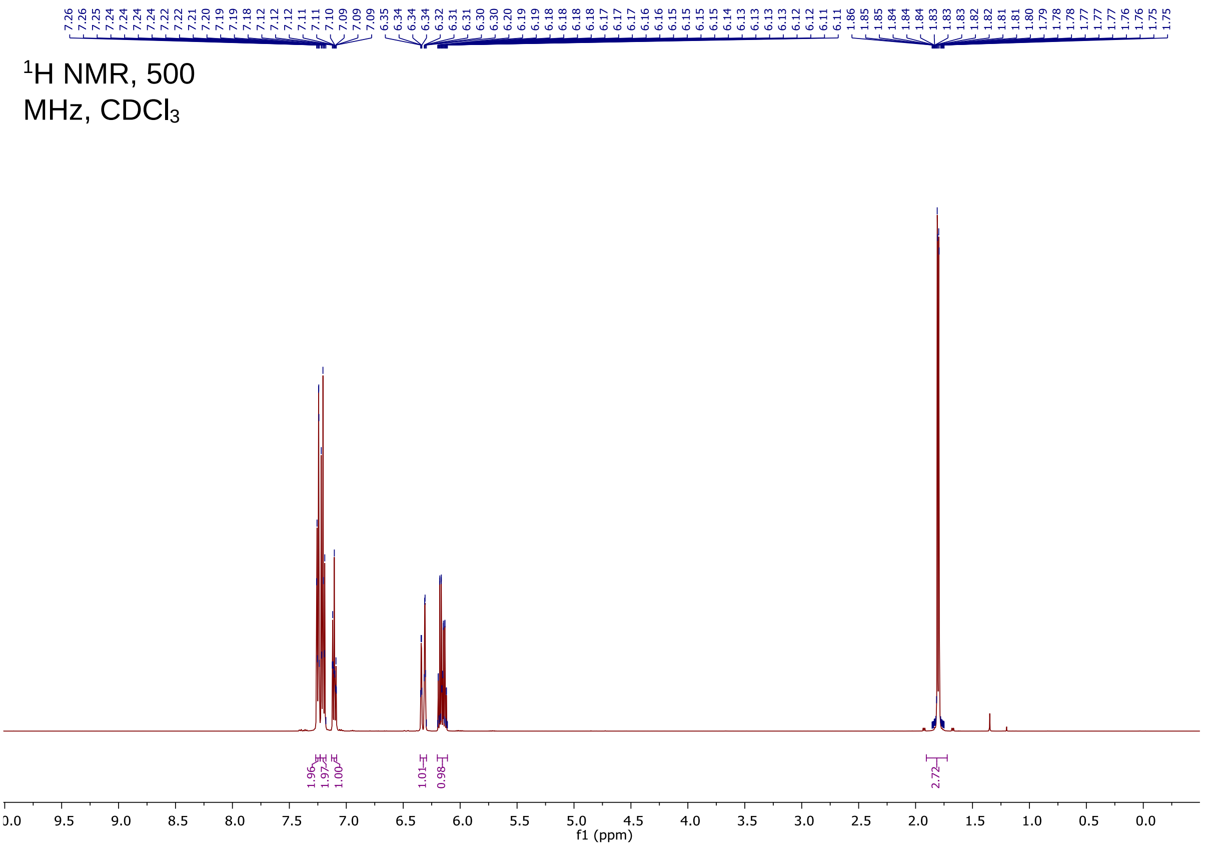

Figure S3. ^{1}H NMR ($CDCl_3$, 500 MHz) of the starting material ((*E*)-prop-1-en-1-ylbenzene) (**2**) used for the experiment.

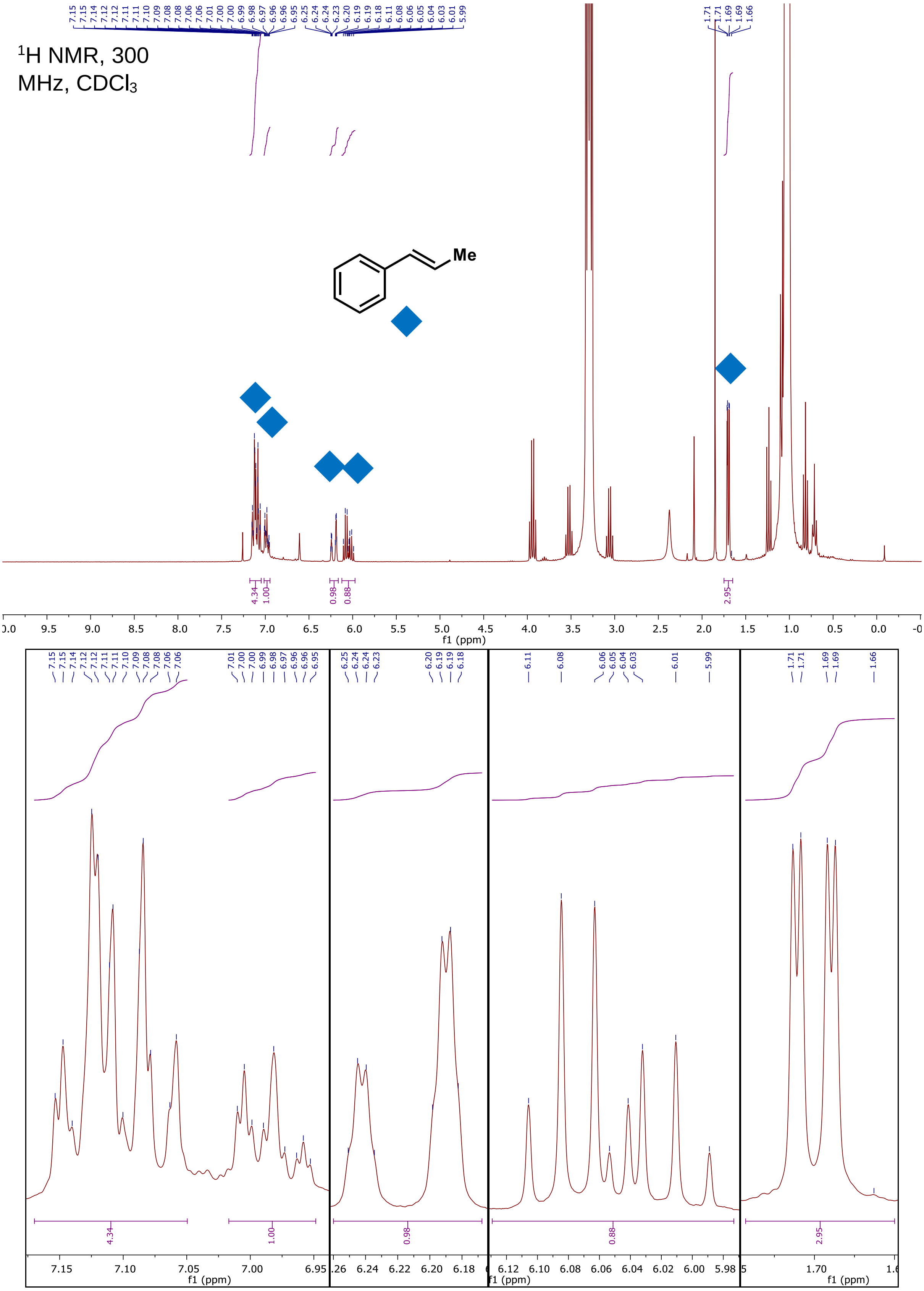

Figure S4. ^{1}H NMR ($CDCl_3$, 300 MHz) of (*E*)-prop-1-en-1-ylbenzene (**2**) equilibrium reaction after a preparative TLC separation. Solvent-free product could not be achieved due to product volatility.

(ii)

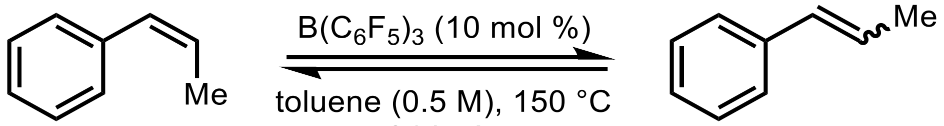

42 >99% *Z*- → **42** >99% (*E*:*Z* = 5:95)

The experiment was prepared according to general procedure 3 using (*Z*)-prop-1-en-1-ylbenzene (**42**). Yield determined by crude ^{1}H NMR using 1,3,5-trimethylbenzene as internal standard.

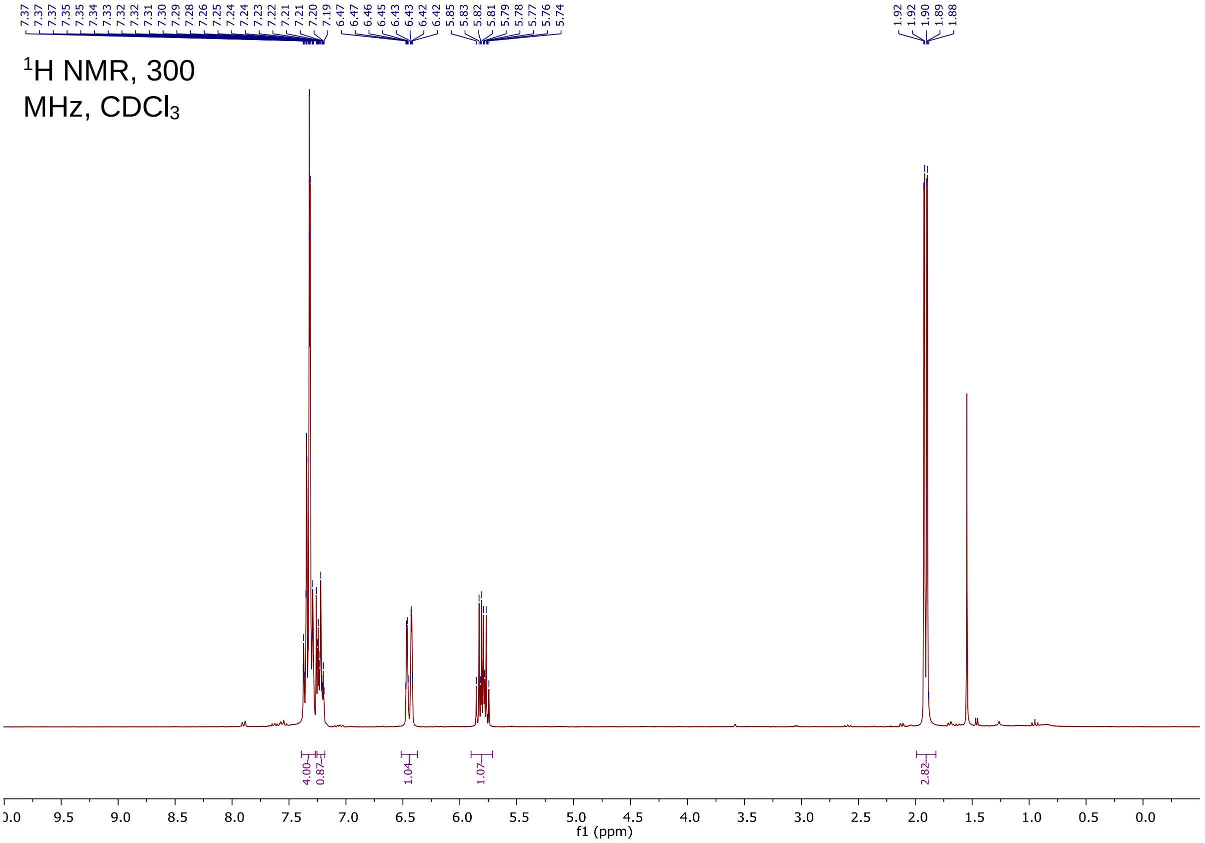

Figure S5. ^{1}H NMR ($CDCl_3$, 300 MHz) of starting material ((*Z*)-prop-1-en-1-ylbenzene) (**42**) used for the experiment.

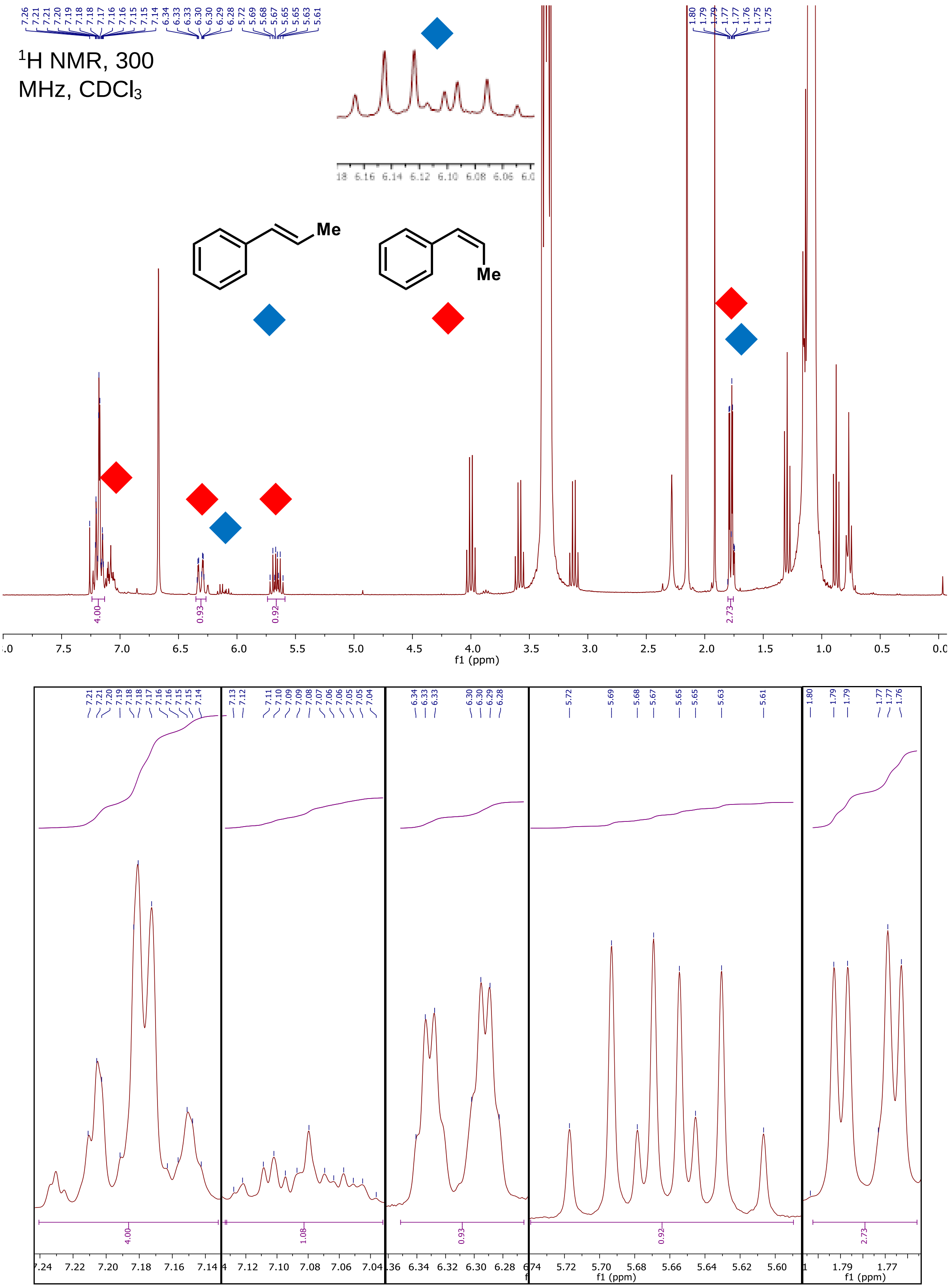

Figure S6. ^{1}H NMR ($CDCl_3$, 300 MHz) of (*Z*)-prop-1-en-1-ylbenzene (**42**) equilibrium reaction after a preparative TLC separation. Solvent-free product could not be achieved due to product volatility.

(iii)

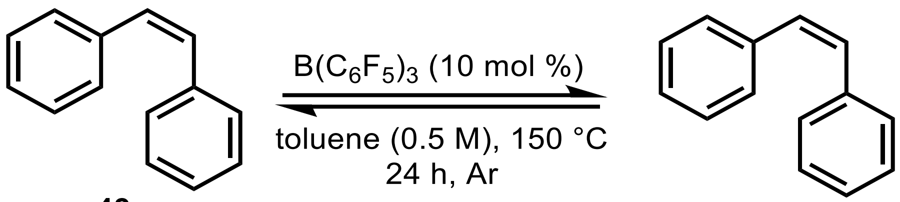

43 95% *Z*- **43** >99%[a] (95% *Z*-)

The experiment was prepared according to general procedure 3 using (*Z*)-stilbene (**43**). Yield determined by crude ^{1}H NMR using 1,3,5-trimethylbenzene as internal standard.

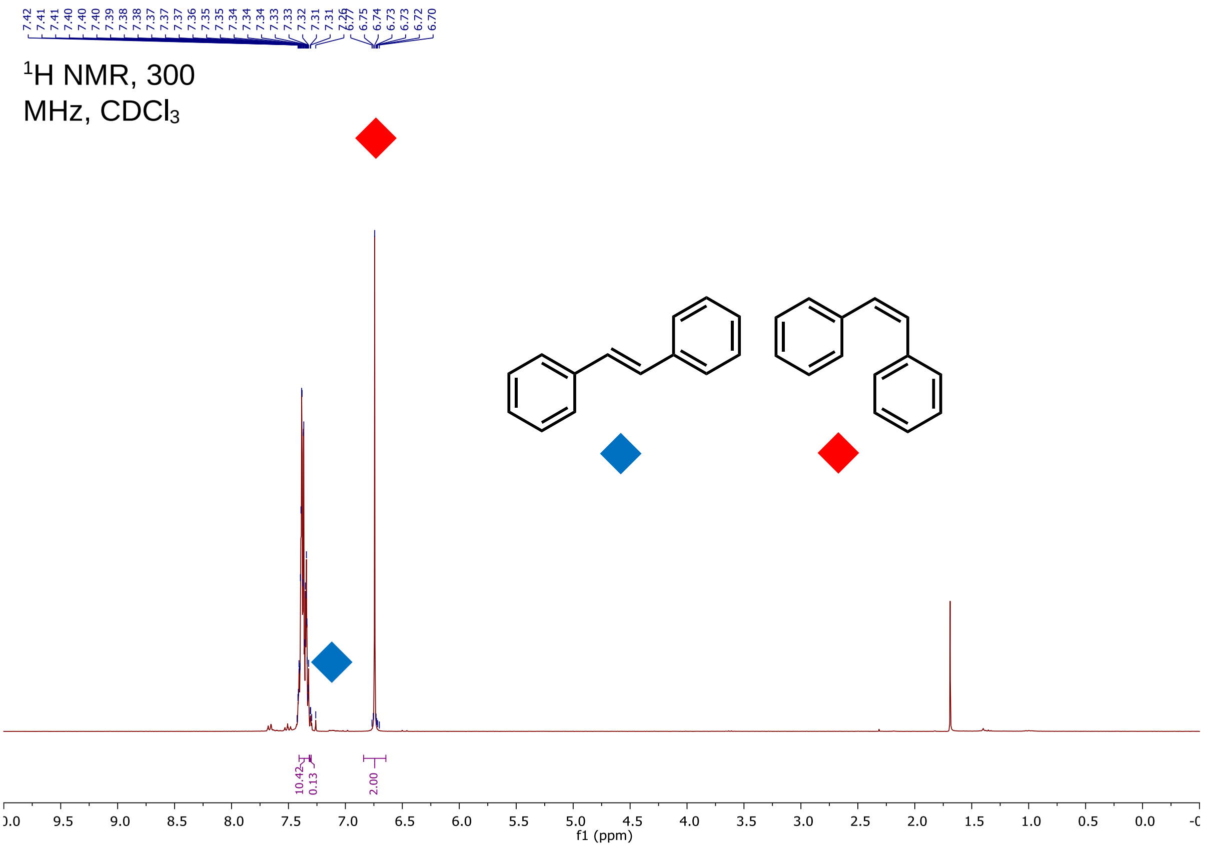

Figure S7. ^{1}H NMR ($CDCl_3$, 300 MHz) of starting material (*Z*)-stilbene (**43**) used for the experiment.

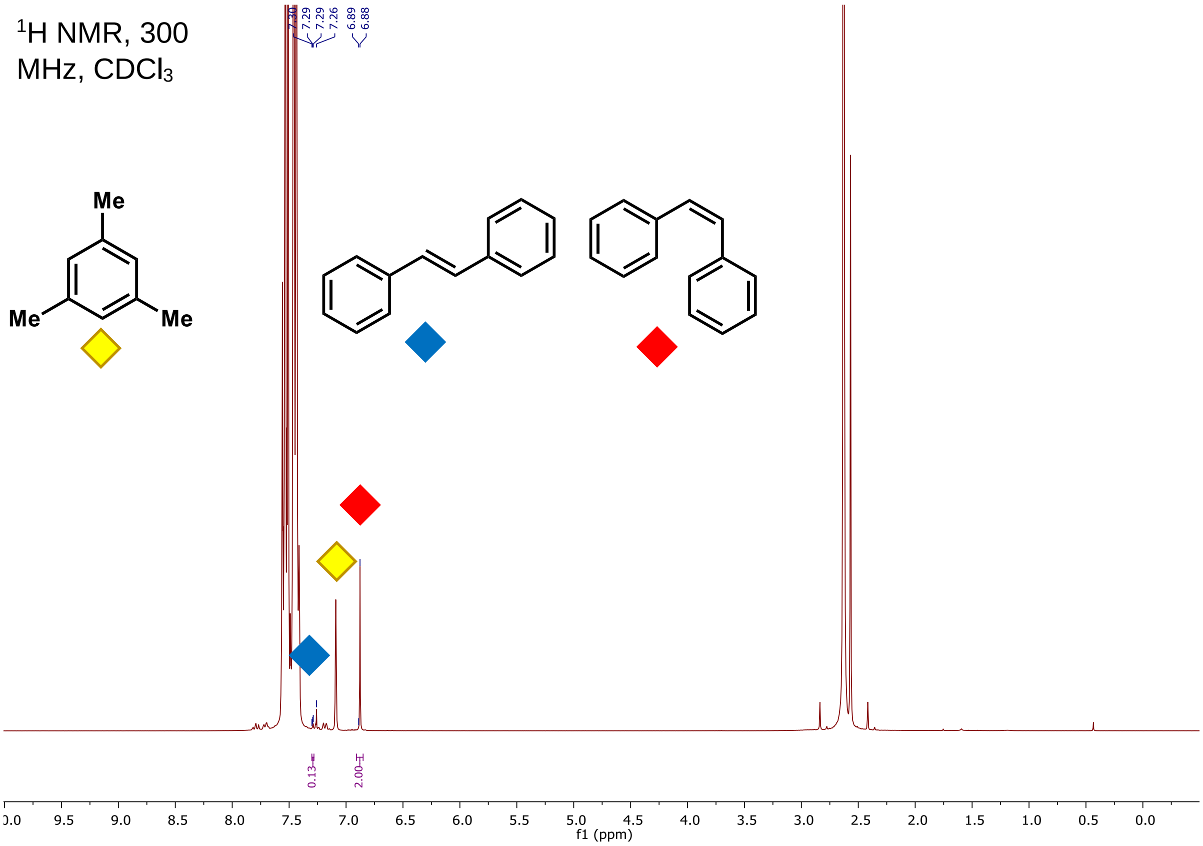

Figure S8. ^{1}H NMR (CDCl$_3$, 300 MHz) of crude *Z*-stilbene (**43**) equilibrium reaction.

2.4.4. Crossover experiment

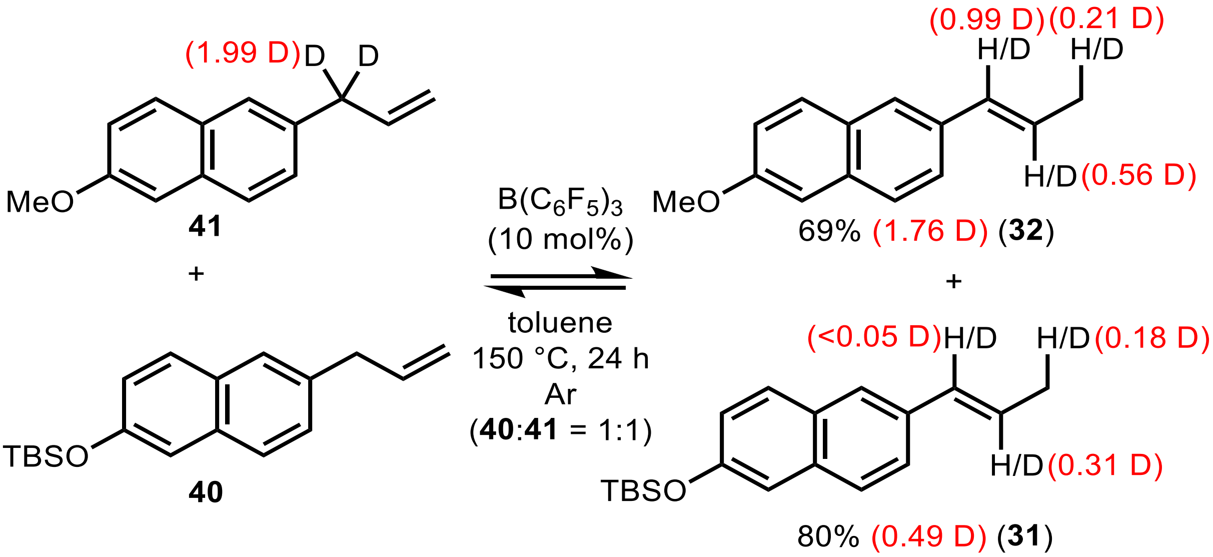

The experiment was prepared according to general procedure 3 using deuterated 2-allyl-6-methoxynaphthalene (**41**) and ((6-allylnaphthalen-2-yl)oxy)(*tert*-butyl)dimethylsilane (**40**). Yield determined by crude ^{1}H NMR using 1,3,5-trimethylbenzene as internal standard. Deuterium incorporation was determined by ^{1}H NMR after purification by preparative TLC.

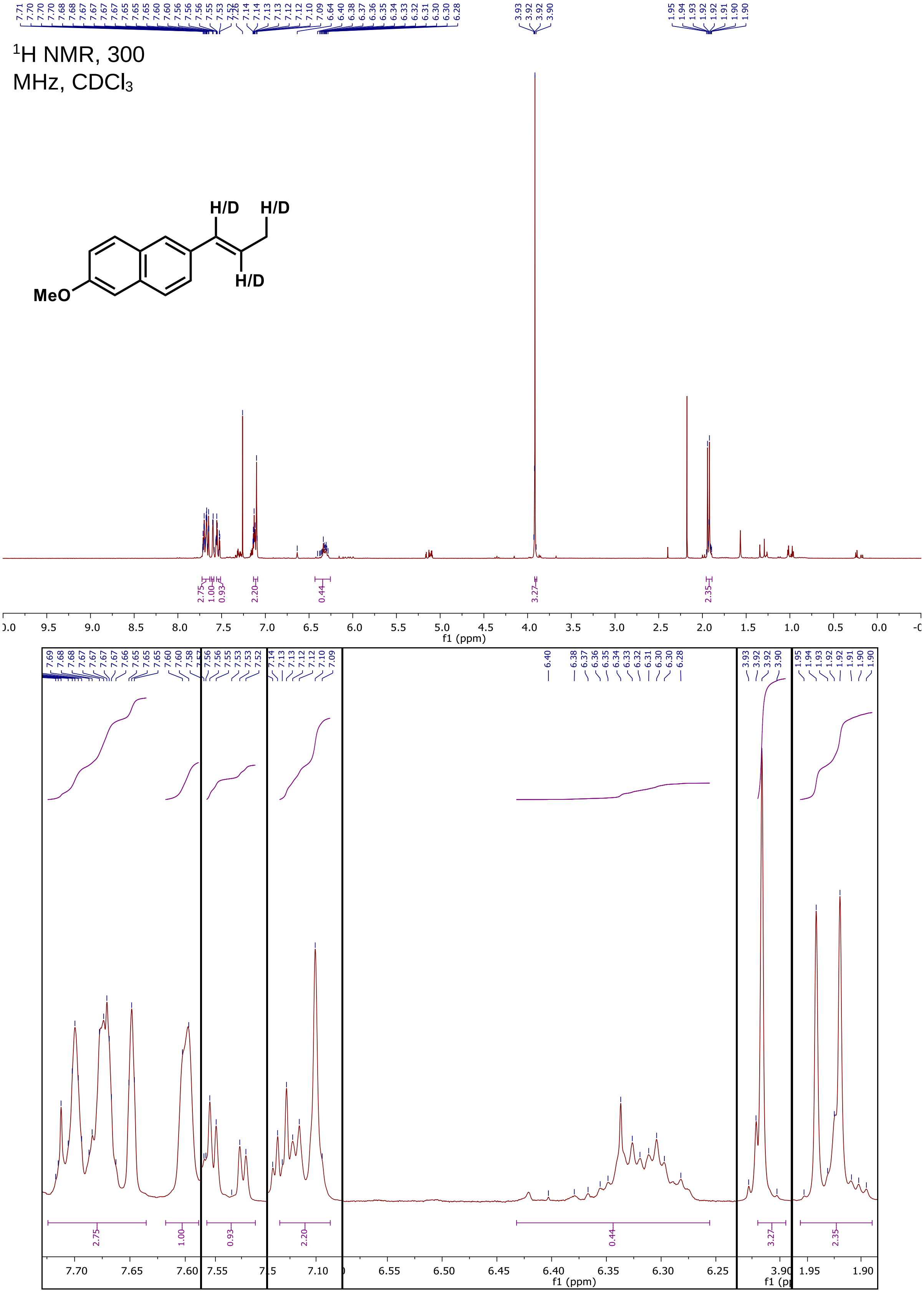

Figure S9. ^1H NMR ($CDCl_3$, 300 MHz) of the deuterated 6-methoxy-2-allylnaphthalene products (**32**).

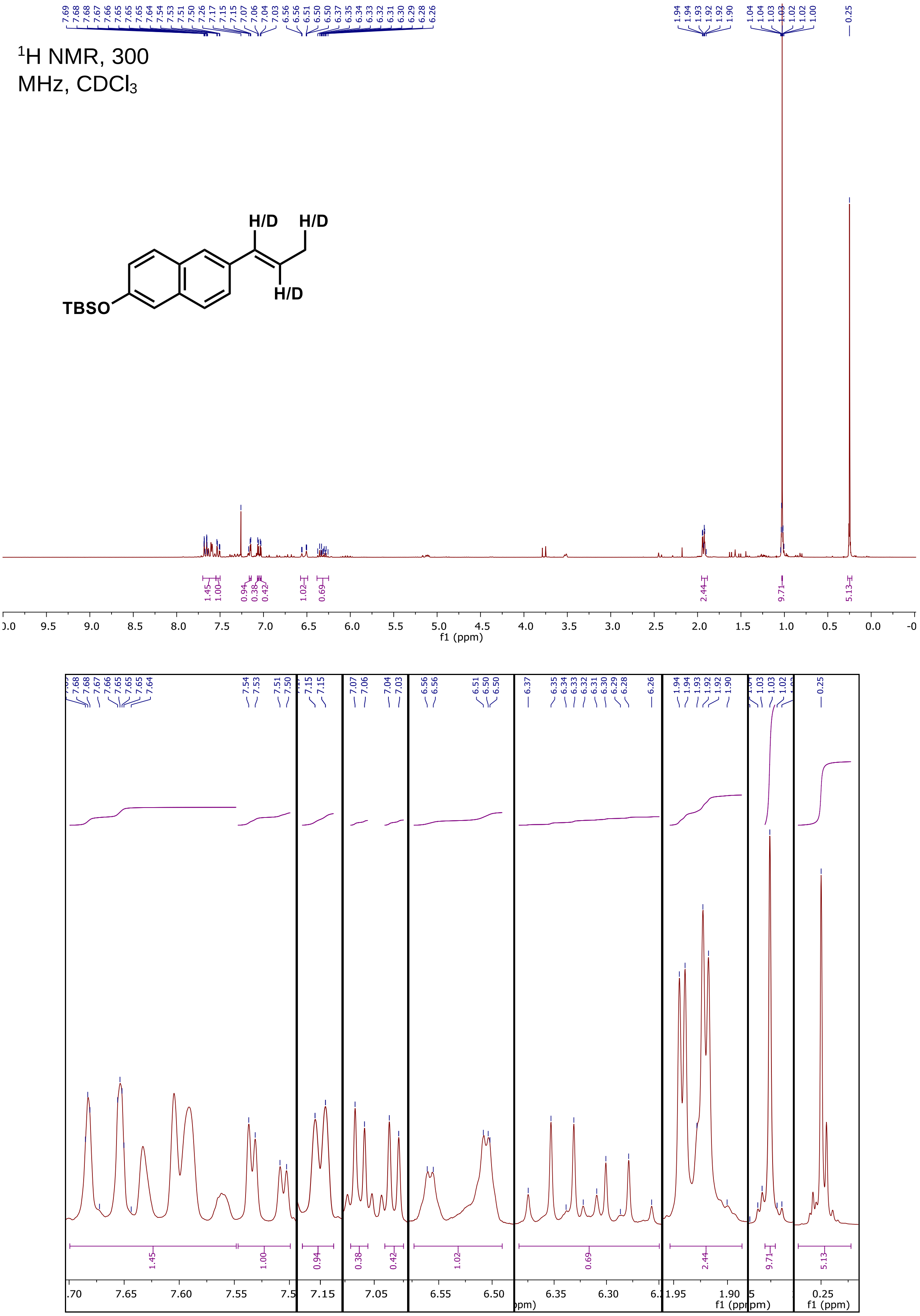

Figure S10. ^{1}H NMR ($CDCl_3$, 300 MHz) of the 6-OTBS-2-allylnaphthalene products (**31**).

2.4.5. $B(C_6F_5)_3$•H_2O-catalyzed isomerization attempt

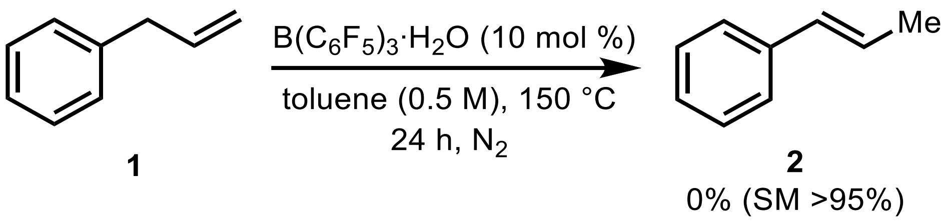

In an oven-dried J. Young flask equipped with stirrer bar was cycled *via* vacuum-N_2 gas backfills. $B(C_6F_5)_3$ (0.02 mmol) as received from the supplier was weighed on the bench and added to the J. Young flask. The atmosphere then was cycled three times again *via* vacuum-N_2 backfills. Anhydrous toluene (0.4 mL) was added using syringe-septa techniques. The mixture was stirred and Et_3SiH (6.4 µL, 0.04 mmol) was added resulting in an effervescence for less than 30 seconds. The mixture was stirred for further 10 min. Allylbenzene (**1**) then was added using syringe-septa techniques. The J. Young flask was sealed and the mixture was stirred at 150 ˚C in oil bath for 24 h. The reaction was cooled to rt, wet toluene (0.2 mL) and 1,3,5-trimethylbenzene (30 µL, 0.2 mmol) were added. The mixture was stirred for 5 minutes and the yield analysed using ^1H NMR.

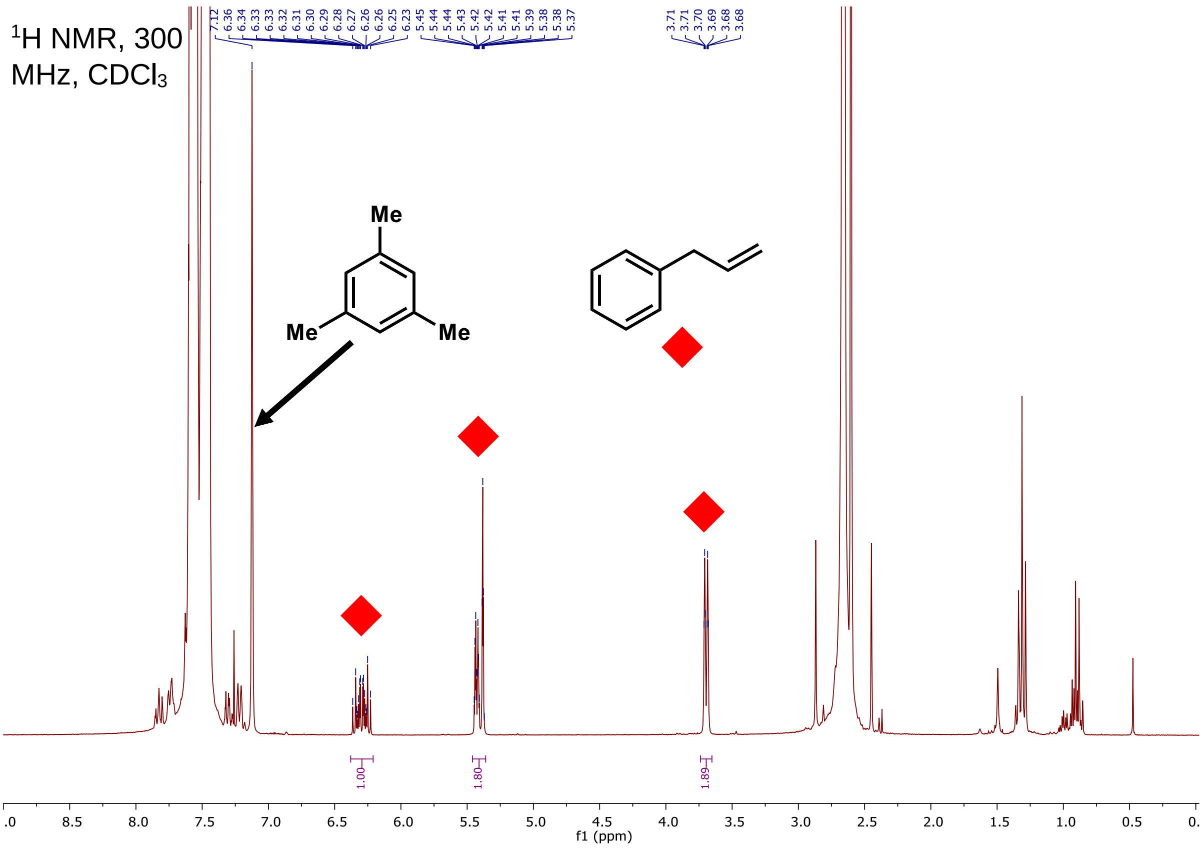

Figure S11. ^1H NMR ($CDCl_3$, 300 MHz) of the crude $B(C_6F_5)_3$·H_2O-catalysed allylbenzene (**1**) isomerization.

2.4.6. Computational method

All density functional theory and semi-empirical calculations were executed using Gaussian 16, A.03.[47] All geometry optimisations were computed with the AM1 semi-empirical method with IEF-PCM(Tol).[48] Frequency calculations at the same level of theory were used to generate free energies with energy minima (confirmed with no imaginary frequencies), corresponding to the relevant intermediate species along the reaction coordinate and saddle points (confirmed with one imaginary frequency), corresponding to the relevant transition states (TSs). Single point energy corrections were calculated at the M06-2X/def2-TZVPP/IEF-PCM(Tol) level, with Aldrichs triple-ζ basis set deployed on all atoms.[49–51]

The system proved challenging to model. TS geometry optimisations using the M06-2X, B3LYP and B97-D3BJ functionals did not converge, despite extensive efforts, for the borane-catalysed isomerisation. Semi-empirical methods yielded TSs for two possible borane-catalysed isomerisation pathways (**TS2** and **TS3**). **TS1** calculated for the uncatalysed process using the M06-2X/def2-TZVPP/IEF-PCM(Tol)//M06-2X/6-31G*/IEF-PCM(Tol) and M06-2X/def2-TZVPP/IEF-PCM(Tol)//AM1/IEF-PCM(Tol) levels of theory, yielded similar free energy barriers of +73.9 and +75.4 kcal mol^{-1} respectively, indicating geometry optimisation using AM1 and single point energy correction using M06-2X is an acceptable approximation to full DFT calculations. All corrections and scaling factors were applied using the GoodVibes programme, frequency cut-off 100.0 cm^{-1} (T = 423.15 K, C = 1.0 mol L^{-1}, vibrational scale factor = 1.0).[52,53]

All reported free energies are referenced to free starting material and catalyst, reported in kcal mol^{-1}. Computed structures are illustrated with CYLView.[54]

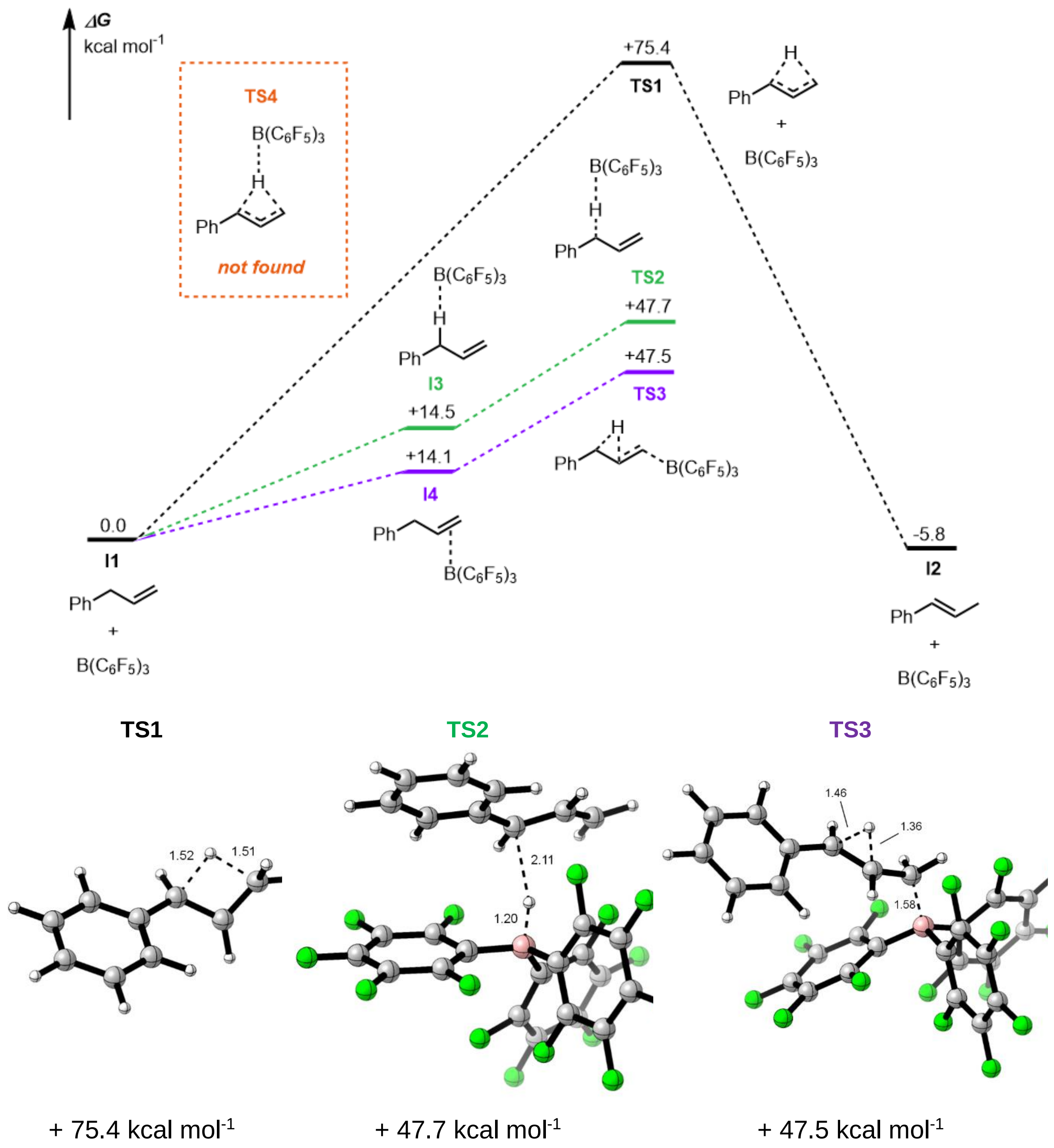

Figure S12: Free energy profile for uncatalysed 1,3-hydride shift, catalysed hydride abstraction and catalysed 1,2-hydride shift, TSs calculated at the M06-2X/def2-TZVPP/IEF-PCM(Tol)//AM1/IEF-PCM(Tol) level of theory.

Based on experimental isotope exchange investigations, three potential pathways were investigated, α-hydride abstraction, 1,2-hydride shift and 1,3-hydride shift. The uncatalysed 1,3-hydride shift proceeds with a $\Delta G^{\ddagger}$ of +75.4 kcal mol^{-1} (**TS1**). In the case of **TS2** (hydride abstraction) and **TS3** (1,2-hydride shift), introduction of the $B(C_6F_5)_3$ catalyst gives a $\Delta G^{\ddagger}$ of +47.7 and +47.5 kcal mol^{-1}, respectively. Although the barrier to isomerisation is still large, introduction of the borane makes the associated TSs more accessible. The similar energies

of **TS2** and **TS3** agree with experimentally determined deuterium scrambling from the allyl benzene α-position across the β- and γ-positions following isomerisation. The TS for the $B(C_6F_5)_3$-catalysed 1,3-hydride shift (**TS4**) could not be located using AM1 or DFT. Therefore, borane-catalysed 1,3-hydride shift cannot be discounted as a plausible mechanism.

Atomic Coordinates

Allyl benzene

M06-2X/def2-TZVPP/IEF-PCM(Tol)-derived Free Energy =

-348.803430

Number of imaginary frequencies = 0

Lowest frequency = 30.0488 cm^{-1}

AM1/IEF-PCM(Tol) Geometry

C	-1.65331	1.42397	-0.0782
C	-0.32649	1.00723	-0.16943
C	-0.00823	-0.35488	-0.11714
C	-1.03726	-1.29304	0.0285
C	-2.36354	-0.87401	0.11862
C	-2.67422	0.48459	0.06556
H	-1.8944	2.49623	-0.12008
H	0.47877	1.74891	-0.282
H	-0.79765	-2.36588	0.07247
H	-3.16591	-1.61753	0.23264
H	-3.72071	0.81405	0.13732
C	1.40322	-0.8137	-0.24431
H	1.50387	-1.85057	0.18416
H	1.65689	-0.88667	-1.33873
C	2.37008	0.07219	0.45336
H	2.0305	0.45681	1.42968
C	3.57238	0.37708	-0.02938
H	3.93464	0.01415	-1.00019
H	4.27818	1.01395	0.51934

***trans*-β-Methylstyrene**

M06-2X/def2-TZVPP/IEF-PCM(Tol)-derived Free Energy =

-348.812608

Number of imaginary frequencies = 0

Lowest frequency = 48.1630 cm^{-1}

AM1/IEF-PCM(Tol) Geometry

C	1.91402	1.32897	0.03236
C	0.54341	1.08749	0.10558
C	0.05045	-0.22498	0.08093
C	0.96223	-1.28993	-0.01035
C	2.33045	-1.04439	-0.08578
C	2.81033	0.26564	-0.06525
H	2.28826	2.36308	0.05306
H	-0.15418	1.93395	0.1904
H	0.58922	-2.32495	-0.0256
H	3.03458	-1.88603	-0.16101
H	3.89123	0.45801	-0.1239
C	-1.37168	-0.51675	0.15762
H	-1.61772	-1.54568	0.47727
H	-3.95266	-1.03473	0.29403
C	-2.35106	0.34827	-0.13813
H	-2.12954	1.37463	-0.47544
C	-3.78561	0.01525	-0.05075
H	-4.29573	0.70657	0.66684
H	-4.26874	0.13773	-1.05305

Tris(pentafluorophenyl)borane ($B(C_6F_5)_3$)

M06-2X/def2-TZVPP/IEF-PCM(Tol)-derived Free Energy =

-2208.347449

Number of imaginary frequencies = 0

Lowest frequency = 16.8020 cm^{-1}

AM1/IEF-PCM(Tol) Geometry

C	-2.15299	3.17338	-0.65463
C	-1.69277	1.86632	-0.63598
C	-0.5079	1.48297	0.00043
C	0.19262	2.51288	0.6363
C	-0.24537	3.82757	0.6537
C	-1.42454	4.15821	-0.00081
B	0.0004	-0.00013	0.00058
C	-1.03033	-1.18153	0.00066
C	-2.2722	-1.08911	0.63713

C	-0.77093	-2.39929	-0.63616
C	-3.19239	-2.1252	0.65485
C	-1.67337	-3.45088	-0.65441
C	-2.89005	-3.31181	0.00013
C	1.53896	-0.30189	0.00031
C	2.08017	-1.42422	0.63539
C	2.46392	0.53308	-0.63469
C	3.43768	-1.70252	0.65309
C	3.82596	0.27789	-0.65304
C	4.31406	-0.84624	-0.00026
F	-2.43455	0.96513	-1.28242
F	-3.27934	3.49014	-1.28598
F	-1.8535	5.41077	-0.00158
F	0.44979	4.7692	1.2843
F	1.33121	2.25625	1.28288
F	2.05474	1.62679	-1.28019
F	1.28859	-2.28204	1.28172
F	3.9051	-2.77595	1.28306
F	5.61323	-1.10132	-0.00078
F	4.66396	1.09517	-1.28347
F	-2.61804	0.02514	1.2845
F	-4.35506	-1.9935	1.28611
F	-3.76073	-4.30922	-0.00022
F	-1.38584	-4.5848	-1.28614
F	0.37987	-2.59142	-1.28347

TS1

M06-2X/def2-TZVPP/IEF-PCM(Tol)-derived Free Energy =

-348.683297

Number of imaginary frequencies = 1

Lowest frequency = -2181.0210 cm^{-1}

AM1/IEF-PCM(Tol) Geometry

C	-1.75457	1.37301	-0.05721
C	-0.402	1.0518	0.03343
C	0.00948	-0.2888	0.08647
C	-0.96473	-1.29931	0.03606
C	-2.31478	-0.97424	-0.06135
C	-2.71375	0.36222	-0.1066
H	-2.06521	2.42753	-0.09284
H	0.35373	1.85124	0.0655
H	-0.65654	-2.35458	0.07527
H	-3.06932	-1.77355	-0.10069
H	-3.78059	0.61665	-0.18205
C	1.41714	-0.63366	0.15971
H	1.61265	-1.69959	0.41113
H	2.48291	-0.92756	-0.87741
C	2.42103	0.31467	0.50379
H	2.39802	1.01079	1.34246
C	3.45325	0.13407	-0.43288
H	3.31069	0.45011	-1.48531
H	4.50732	0.16038	-0.12461

I3

M06-2X/def2-TZVPP/IEF-PCM(Tol)-derived Free Energy =

-2557.127771

Number of imaginary frequencies = 0

Lowest frequency = 3.6220 cm^{-1}

AM1/IEF-PCM(Tol) Geometry

C	-5.31465	1.41911	-0.57929
C	-4.41514	0.43168	-0.97594
C	-4.82553	-0.90429	-1.06398
C	-6.14715	-1.23706	-0.74721
C	-7.04505	-0.24741	-0.34891
C	-6.63125	1.08147	-0.26488
H	-4.98542	2.46654	-0.5154
H	-3.37568	0.69988	-1.2215
H	-6.47968	-2.28369	-0.81379
H	-8.08224	-0.51722	-0.1017

H	-7.34044	1.86128	0.04788
C	-3.85965	-1.96031	-1.48068
H	-4.28237	-2.977	-1.25539
C	-3.50297	-1.85424	-2.91978
H	-3.30267	-0.82877	-3.27351
C	-3.41397	-2.89955	-3.73824
H	-3.61631	-3.92909	-3.41516
H	-3.13101	-2.79281	-4.79344
C	-0.14118	0.65178	3.87995
C	0.64674	0.81812	2.71034
C	0.43727	0.01939	1.56675
C	-0.58907	-0.94732	1.60535
C	-1.39787	-1.11868	2.7599
C	-1.17068	-0.31822	3.90415
B	1.27853	0.18974	0.35612
C	1.33193	1.50442	-0.33184
C	0.16584	2.08913	-0.86743
C	2.5515	2.20014	-0.46523
C	0.20885	3.34284	-1.53271
C	2.61209	3.46365	-1.11064
C	1.43648	4.03597	-1.6508
C	2.07005	-0.95179	-0.16618
C	1.88589	-1.41709	-1.48499
C	3.02718	-1.59821	0.64361
C	2.63198	-2.51463	-1.99034
C	3.79722	-2.68452	0.15012
C	3.59546	-3.1485	-1.17101
F	1.62128	1.7541	2.73911
F	0.08975	1.42112	4.96409
F	-1.92721	-0.47859	5.00672
F	-2.37677	-2.04692	2.76662
F	-0.85159	-1.72956	0.53446
F	3.25965	-1.19	1.91077
F	0.97604	-0.84694	-2.30556
F	2.42567	-2.94754	-3.25148
F	4.3144	-4.18425	-1.64458
F	4.71545	-3.2758	0.94248
F	-1.02977	1.46358	-0.78469
F	-0.92046	3.87337	-2.0464
F	1.4855	5.22991	-2.27221
F	3.79001	4.11363	-1.21258
F	3.70061	1.69624	0.03696
H	-2.91575	-1.85433	-0.87236

TS2

M06-2X/def2-TZVPP/IEF-PCM(Tol)-derived Free Energy =

-2557.074803

Number of imaginary frequencies = 1

Lowest frequency = -66.0365 cm^{-1}

AM1/IEF-PCM(Tol) Geometry

C	4.40046	2.12915	1.76469
C	3.14536	1.80165	2.26052
C	2.71456	0.45363	2.28698
C	3.57826	-0.54405	1.76507
C	4.83608	-0.20635	1.28641
C	5.25064	1.12927	1.2838
H	4.72238	3.18258	1.73982
H	2.46669	2.60216	2.60117
H	3.23886	-1.59551	1.73769
H	5.50436	-0.99024	0.89472
H	6.24638	1.39472	0.89168
C	1.46223	0.05652	2.82361
H	1.16266	-1.00806	2.69151
C	0.67451	0.87283	3.6805
H	0.92825	1.94654	3.75623
C	-0.38119	0.38316	4.36812
H	-0.70167	-0.66897	4.30386
H	-0.98721	1.02582	5.02571
C	2.53405	-0.82661	-2.50102

C	1.46527	-0.14972	-1.85045
C	0.83659	-0.67274	-0.6983
C	1.37005	-1.88136	-0.18879
C	2.43425	-2.57599	-0.81941
C	3.01853	-2.05136	-1.99212
B	-0.30524	-0.00968	0.06868
C	-0.78662	1.34413	-0.45927
C	-0.47342	2.53732	0.22224
C	-1.61759	1.46615	-1.59843
C	-0.97566	3.80283	-0.1847
C	-2.13698	2.71797	-2.02334
C	-1.81819	3.89679	-1.31158
C	-1.52485	-0.92128	0.26722
C	-2.33554	-0.74633	1.40836
C	-1.91934	-1.93094	-0.63988
C	-3.46753	-1.56087	1.67327
C	-3.04836	-2.76058	-0.39866
C	-3.82644	-2.58106	0.76681
F	1.08721	1.02552	-2.40299
F	3.09104	-0.29191	-3.60949
F	4.03012	-2.70135	-2.60638
F	2.89582	-3.73006	-0.28639
F	0.88411	-2.43619	0.95322
F	-1.24713	-2.15076	-1.79292
F	-2.06634	0.22514	2.31776
F	-4.19719	-1.35768	2.79364
F	-4.89634	-3.36829	1.00748
F	-3.37995	-3.72172	-1.2878
F	0.33097	2.54	1.31557
F	-0.64361	4.9134	0.51223
F	-2.30819	5.09133	-1.70597
F	-2.93445	2.78373	-3.11166
F	-1.94408	0.38989	-2.35049
H	0.17317	0.16695	1.15204

I4

M06-2X/def2-TZVPP/IEF-PCM(Tol)-derived Free Energy =

-2557.128404

Number of imaginary frequencies = 0

Lowest frequency = 2.1306 cm^{-1}

AM1/IEF-PCM(Tol) Geometry

C	-8.91096	-0.47478	1.07762
C	-7.77467	-0.98996	0.45744
C	-6.86776	-0.13436	-0.18018
C	-7.11467	1.24274	-0.18778
C	-8.25282	1.75606	0.43316
C	-9.15225	0.89932	1.06651
H	-9.61858	-1.15323	1.57615
H	-7.58534	-2.07365	0.46604
H	-6.40763	1.92369	-0.68456
H	-8.43973	2.83986	0.4229
H	-10.04929	1.30517	1.55586
C	-5.65996	-0.68994	-0.8541
H	-5.97806	-1.52276	-1.54328
H	-5.17946	0.09989	-1.49317
C	-4.67829	-1.2373	0.11866
H	-5.11702	-1.84063	0.93133
C	-3.36535	-1.03991	0.03399
H	-2.89975	-0.43677	-0.75779
H	-2.65936	-1.47042	0.7574
C	2.33769	-2.64087	-2.73341
C	2.51473	-1.80726	-1.59746
C	1.54405	-0.85108	-1.23247
C	0.37861	-0.74724	-2.02005
C	0.17481	-1.5841	-3.14906
C	1.16056	-2.53226	-3.51051
B	1.74679	0.02464	-0.05124
C	1.94448	-0.57488	1.29224
C	0.96286	-1.40996	1.86503

C	3.12108	-0.32323	2.02839
C	1.14084	-1.98042	3.15322
C	3.32617	-0.89975	3.30982
C	2.32978	-1.72837	3.87728
C	1.75611	1.49975	-0.21397
C	0.84276	2.31138	0.49079
C	2.67979	2.12586	-1.07683
C	0.83786	3.72281	0.33396
C	2.70389	3.53718	-1.23184
C	1.77562	4.33931	-0.52736
F	3.65792	-1.94637	-0.89019
F	3.28876	-3.53791	-3.06699
F	0.97998	-3.32426	-4.58476
F	-0.95486	-1.47054	-3.87812
F	-0.59538	0.13842	-1.71289
F	3.58895	1.40383	-1.76863
F	-0.07404	1.77004	1.32296
F	-0.0562	4.47391	1.00995
F	1.78466	5.67796	-0.67534
F	3.60725	4.11055	-2.05391
F	-0.1919	-1.67749	1.21529
F	0.17941	-2.76545	3.68229
F	2.51147	-2.27242	5.09587
F	4.46718	-0.65271	3.98625
F	4.10353	0.45835	1.52789

TS3

M06-2X/def2-TZVPP/IEF-PCM(Tol)-derived Free Energy =

-2557.075254

Number of imaginary frequencies = 1

Lowest frequency = -1046.6600 cm^{-1}

AM1/IEF-PCM(Tol) Geometry

C	-5.72527	1.29414	-0.34345
C	-4.40419	1.01445	-0.68253
C	-4.11844	0.20471	-1.79643
C	-5.18075	-0.32493	-2.55498
C	-6.49683	-0.03657	-2.21165
C	-6.77155	0.77387	-1.107
H	-5.94176	1.92242	0.53553
H	-3.58717	1.41012	-0.0544
H	-4.9681	-0.97077	-3.4218
H	-7.3236	-0.45221	-2.80883
H	-7.81579	0.99708	-0.83479
C	-2.76363	-0.13258	-2.16536
H	-2.63196	-1.07286	-2.74814
H	-2.11336	0.94075	-2.91507
C	-1.60932	0.5506	-1.71642
H	-1.71054	1.4133	-1.01385
C	-0.24625	0.01357	-1.84521
H	-0.23924	-1.03082	-2.24142
H	0.34929	0.66392	-2.53378
C	-2.2988	-0.84205	2.19572
C	-1.3861	-0.126	1.37261
C	-0.53182	-0.76792	0.44903
C	-0.74307	-2.15997	0.2791
C	-1.6557	-2.89763	1.07831
C	-2.42803	-2.23976	2.06161
B	0.4873	-0.01252	-0.45063
C	0.87976	1.38382	0.10163
C	0.76511	2.60234	-0.6011
C	1.49563	1.4703	1.37893
C	1.26546	3.835	-0.09472
C	2.00449	2.68614	1.90637
C	1.8968	3.88296	1.16325
C	1.84716	-0.74763	-0.63447
C	2.67952	-0.4969	-1.75092
C	2.36598	-1.65281	0.32355
C	3.91809	-1.16615	-1.95192
C	3.6027	-2.33229	0.14741

C 4.3843 -2.09947 -1.00379
F -1.40765 1.22451 1.51069
F -3.05689 -0.17381 3.09292
F -3.28724 -2.93134 2.83934
F -1.79261 -4.22821 0.89084
F -0.08077 -2.86396 -0.67177
F 1.71597 -1.92297 1.47997
F 2.36192 0.41422 -2.70536
F 4.65043 -0.89731 -3.05594
F 5.55437 -2.74612 -1.18794
F 4.02961 -3.20074 1.09009
F 0.15947 2.69326 -1.81516
F 1.1261 4.96102 -0.82962
F 2.38062 5.04446 1.65084
F 2.59001 2.69877 3.12332
F 1.62047 0.38449 2.17713

I1’

M06-2X/def2-TZVPP/IEF-PCM(Tol)-derived Free Energy =

-348.792055

Number of imaginary frequencies = 0

Lowest frequency = 30.4902 cm^{-1}

M06-2X/6-31G*/IEF-PCM(Tol) Geometry

C 1.60119 1.43929 0.05905
C 0.29641 0.98809 0.23035
C 0.00381 -0.37886 0.20543
C 1.04614 -1.28262 -0.00358
C 2.35468 -0.83522 -0.17734
C 2.63594 0.52755 -0.1458
H 1.81181 2.50437 0.08282
H -0.51252 1.69911 0.38022
H 0.83098 -2.34814 -0.0296
H 3.15327 -1.55296 -0.33955
H 3.65411 0.87874 -0.28222
C -1.42093 -0.858 0.41117
H -1.45665 -1.9419 0.24602
H -1.72442 -0.68478 1.45129
C -2.39724 -0.17153 -0.50532
H -2.21336 -0.2929 -1.57256
C -3.4232 0.56748 -0.09241
H -3.6253 0.71193 0.96649
H -4.09865 1.04953 -0.7922

TS1’

M06-2X/def2-TZVPP/IEF-PCM(Tol)-derived Free Energy =

-348.674266

Number of imaginary frequencies = 1

Lowest frequency = -1866.6607 cm^{-1}

M06-2X/6-31G*/IEF-PCM(Tol) Geometry

C -1.782 1.37041 -0.0563
C -0.42824 1.04882 -0.05733
C -0.00944 -0.28663 -0.00421
C -0.98298 -1.29441 0.01354
C -2.3358 -0.97603 -0.00126
C -2.73973 0.35881 -0.03054
H -2.09087 2.41082 -0.09046
H 0.32165 1.83438 -0.11135
H -0.66891 -2.33498 0.04676
H -3.07818 -1.76824 0.01504
H -3.7963 0.60788 -0.04217
C 1.42894 -0.6114 0.03067
H 1.6447 -1.66997 0.17639
H 2.58651 -0.83527 -0.89417
C 2.42452 0.26917 0.53179
H 2.33274 0.89508 1.41999
C 3.5466 0.14375 -0.30264
H 3.44402 0.49703 -1.32754
H 4.57342 0.22834 0.0652

3. References

[1] D. C. Gerbino, S. D. Mandolesi, H. G. Schmalz, J. C. Podestá, *Eur. J. Org. Chem.* **2009**, 3964–3972.

[2] S. E. Denmark, N. S. Werner, *J. Am. Chem. Soc.* **2008**, *130*, 16382–16393.

[3] M. J. Gresser, S. M. Wales, P. A. Keller, *Tetrahedron* **2010**, *66*, 6965–6976.

[4] J. Leister, D. Chao, K. L. Billingsley, *Tetrahedron Lett.* **2021**, *66*, DOI 10.1016/j.tetlet.2020.152800.

[5] T. Furayama, M. Yonehara, S. Arimoto, M. Kobayashi, Y. Matsumoto, M. Uchiyama, *Chem. Eur. J.* **2008**, *14*, 10348–10356.

[6] H. Kwart, D. Drayer, *J. Org. Chem* **1974**, *39*, 2157.

[7] J. Cao, G. Li, G. Wang, L. Gao, S. Li, *Org. Biomol. Chem.* **2022**, *20*, 2857–2862.

[8] S. Lazzaroni, D. Dondi, M. Fagnoni, A. Albini, *Eur. J. Org. Chem.* **2007**, 4360–4365.

[9] S. Lin, C. X. Song, G. X. Cai, W. H. Wang, Z. J. Shi, *J. Am. Chem. Soc.* **2008**, *130*, 12901–12903.

[10] M. Warsitz, S. Doye, *Chem. Eur. J.* **2020**, *26*, 15121–15125.

[11] W. H. Roark, B. D. Roth, *Phosphonamide ACAT Inhibitors*, **2000**.

[12] D. S. Ziegler, K. Karaghiosoff, P. Knochel, *Angew. Chem.* **2018**, *130*, 6811–6815.

[13] A. M. Echavarren, J. K. Stille, *J. Am. Chem. Soc* **1987**, *109*, 5478–5486.

[14] A. Inoue, K. Kitagawa, H. Shinokubo, K. Oshima, *J. Org. Chem.* **2001**, *66*, 4333–4339.

[15] D. Phillips, G. Brodie, S. Memarzadeh, G. L. Tang, D. J. France, *RSC Adv.* **2020**, *10*, 30624–30630.

[16] J. Albarrán-Velo, V. Gotor-Fernández, I. Lavandera, *Adv. Synth. Catal.* **2021**, *363*, 4096–4108.

[17] L. Pretali, F. Doria, D. Verga, A. Profumo, M. Freccero, *J. Org. Chem.* **2009**, *74*, 1034–1041.

[18] Z. Liu, A. A. Yasseri, R. S. Loewe, A. B. Lysenko, V. L. Malinovskii, Q. Zhao, S. Surthi, Q. Li, V. Misra, J. S. Lindsey, D. F. Bocian, *J. Org. Chem.* **2004**, *69*, 5568–5577.

[19] M. O. Akram, P. S. Mali, N. T. Patil, *Org. Lett.* **2017**, *19*, 3075–3078.

[20] T. Dao-Huy, S. Latkolik, J. Bräuer, A. Pfeil, H. Stuppner, M. Schnürch, V. M. Dirsch, M. D. Mihovilovic, *Biomolecules* **2020**, *10*, 1–20.

[21] X. Yu, H. Zhao, P. Li, M. J. Koh, *J. Am. Chem. Soc.* **2020**, *142*, 18223–18230.

[22] M. B. Andrus, K. C. Harper, M. A. Christiansen, M. A. Binkley, *Tetrahedron Lett.* **2009**, *50*, 4541–4544.

[23] P. Miiller, J. Seres, K. Steiner, S. E. Helali, E. Hardegger, *Helv. Chim. Acta.* **1974**, *57*.

[24] X. L. Lu, M. Shannon, X. S. Peng, H. N. C. Wong, *Org. Lett.* **2019**, *21*, 2546–2549.

[25] H. Liu, M. Xu, C. Cai, J. Chen, Y. Gu, Y. Xia, *Org. Lett.* **2020**, *22*, 1193–1198.

[26] K. Nakayama, N. Maeta, G. Horiguchi, H. Kamiya, Y. Okada, *Org. Lett.* **2019**, *21*, 2246–2250.

[27] N. P. R. Onuska, M. E. Schutzbach-Horton, J. L. Rosario Collazo, D. A. Nicewicz, *Synlett* **2020**, *31*, 55–59.

[28] K. E. Kawamura, A. S. M. Chang, D. J. Martin, H. M. Smith, P. T. Morris, A. K. Cook, *Organometallics* **2022**, *41*, 486–496.

[29] H. Albright, H. L. Vonesh, C. S. Schindler, *Org. Lett.* **2020**, *22*, 3155–3160.

[30] M. R. Tiddens, R. J. M. Klein Gebbink, M. Otte, *Org. Lett.* **2016**, *18*, 3714–3717.

[31] G. Rong, D. Liu, L. Lu, H. Yan, Y. Zheng, J. Chen, J. Mao, *Tetrahedron* **2014**, *70*, 5033–5037.

[32] M. R. Swart, L. Twigge, E. Erasmus, C. Marais, B. C. B. Bezuidenhoudt, *Eur. J. Inorg Chem.* **2021**, *2021*, 1752–1762.

[33] C. Y. Liu, P. Knochel, *Org. Lett.* **2005**, *7*, 2543–2546.

[34] C. Zhang, P. X. Liu, L. Y. Huang, S. P. Wei, L. Wang, S. Y. Yang, X. Q. Yu, L. Pu, Q. Wang, *Chem. Eur. J.* **2016**, *22*, 10969–10975.

[35] Z. M. Wang, X. L. Sang, C. M. Che, J. Chen, *Tetrahedron Lett.* **2014**, *55*, 1736–1739.

[36] J. Zhao, B. Cheng, C. Chen, Z. Lu, *Org. Lett.* **2020**, *22*, 837–841.

[37] Tiecco M, Tingoli M, Wenkert E, *J. Org. Chem.* **1985**, *50*, 3828–3831.

[38] B. Yang, Z. X. Wang, *J. Org. Chem.* **2020**, *85*, 4772–4784.

[39] N. Huber, R. Li, C. T. J. Ferguson, D. W. Gehrig, C. Ramanan, P. W. M. Blom, K. Landfester, K. A. I. Zhang, *Catal. Sci. Technol.* **2020**, *10*, 2092–2099.

[40] J. H. Shin, E. Y. Seong, H. J. Mun, Y. J. Jang, E. J. Kang, *Org. Lett.* **2018**, *20*, 5872–5876.

[41] M. Roche, S. M. Salim, J. Bignon, H. Levaique, J. D. Brion, M. Alami, A. Hamze, *J. Org. Chem.* **2015**, *80*, 6715–6727.

[42] K. A. Bahou, D. C. Braddock, A. G. Meyer, G. P. Savage, Z. Shi, T. He, *J. Org. Chem.* **2020**, *85*, 4906–4917.

[43] Q. Ye, M. A. Upshur, E. S. Robinson, F. M. Geiger, R. C. Sullivan, R. J. Thomson, N. M. Donahue, *Chem* **2018**, *4*, 318–333.

[44] C. Chen, T. R. Dugan, W. W. Brennessel, D. J. Weix, P. L. Holland, *J. Am. Chem. Soc.* **2014**, *136*, 945–955.

[45] W. Yu, J. Han, D. Fang, M. Wang, J. Liao, *Org. Lett.* **2021**, *23*, 2482–2487.

[46] H. W. Suh, L. M. Guard, N. Hazari, *Polyhedron* **2014**, *84*, 37–43.

[47] G. W. T. M. J. Frisch, H. B. Schlegel, G. E. Scuseria, J. R. C. M. A. Robb, G. Scalmani, V. Barone, H. N. G. A. Petersson, X. Li, M. Caricato, A. v. Marenich, B. G. J. J. Bloino, R. Gomperts, B. Mennucci, H. P. Hratchian, A. F. I. J. v. Ortiz, J. L. Sonnenberg, D.

Williams-Young, F. L. F. Ding, F. Egidi, J. Goings, B. Peng, A. Petrone, D. R. T. Henderson, V. G. Zakrzewski, J. Gao, N. Rega, W. L. G. Zheng, M. Hada, M. Ehara, K. Toyota, R. Fukuda, M. I. J. Hasegawa, T. Nakajima, Y. Honda, O. Kitao, H. Nakai, K. T. T. Vreven, J. A. Montgomery Jr., J. E. Peralta, M. J. B. F. Ogliaro, J. J. Heyd, E. N. Brothers, K. N. Kudin, T. A. K. V. N. Staroverov, R. Kobayashi, J. Normand, A. P. R. K. Raghavachari, J. C. Burant, S. S. Iyengar, M. C. J. Tomasi, J. M. Millam, M. Klene, C. Adamo, R. Cammi, R. L. M. J. W. Ochterski, K. Morokuma, O. Farkas, A. D. J. F. J. B. Foresman, **2016**.

[48] M. J. S. Dewar, E. G. Zoebisch, E. F. Healy, J. J. P. Stewart, *J. Am. Chem. Soc.* **1985**, *107*, 3902–3909.

[49] Y. Zhao, D. G. Truhlar, *Theor. Chem. Acc.* **2008**, *120*, 215–241.

[50] F. Weigend, R. Ahlrichs, *Phys. Chem. Chem. Phys.* **2005**, *7*, 3297–3305.

[51] J. Tomasi, B. Mennucci, R. Cammi, *Chem. Rev.* **2005**, *105*, 2999–3093.

[52] G. Luchini, J. Alegre-Requena, I. Funes-Ardoiz, J. Rodriguez-Guerra, jingTao Chen, R. Paton, **n.d.**

[53] S. Grimme, *Chem. Eur. J.* **2012**, *18*, 9955–9964.

[54] C. Y. Legault, **2020**.
